# The Convergent Synthetic Efforts toward Knightol and a Simplified Analog

**DOI:** 10.1021/acs.joc.6c01838

**Published:** 2026-09-30

**Authors:** Learnmore Jeremia, Elise L. Bezold, Kate Bright, William M. Wuest

**Affiliations:** Department of Chemistry, 1371 Emory University, Atlanta, Georgia 30322, United States

## Abstract

We report our efforts toward the convergent total synthesis of the cembranoid diterpene (−)-knightol, a marine natural product reported to exhibit antibiofilm activity against *Staphylococcus aureus*, and a rationally designed aryl analog, *m*knightol. Our strategy was guided by a two-phase approach involving construction of the macrocyclic framework, followed by selective epoxidation and further diversification. Both targets were designed to arise from common western fragment building blocks and an eastern fragment accessible on a gram scale. A scalable cross-electrophile coupling provided efficient access to an advanced intermediate en route to the cyclophane and enabled evaluation of key late-stage transformations.

## Graphical abstract

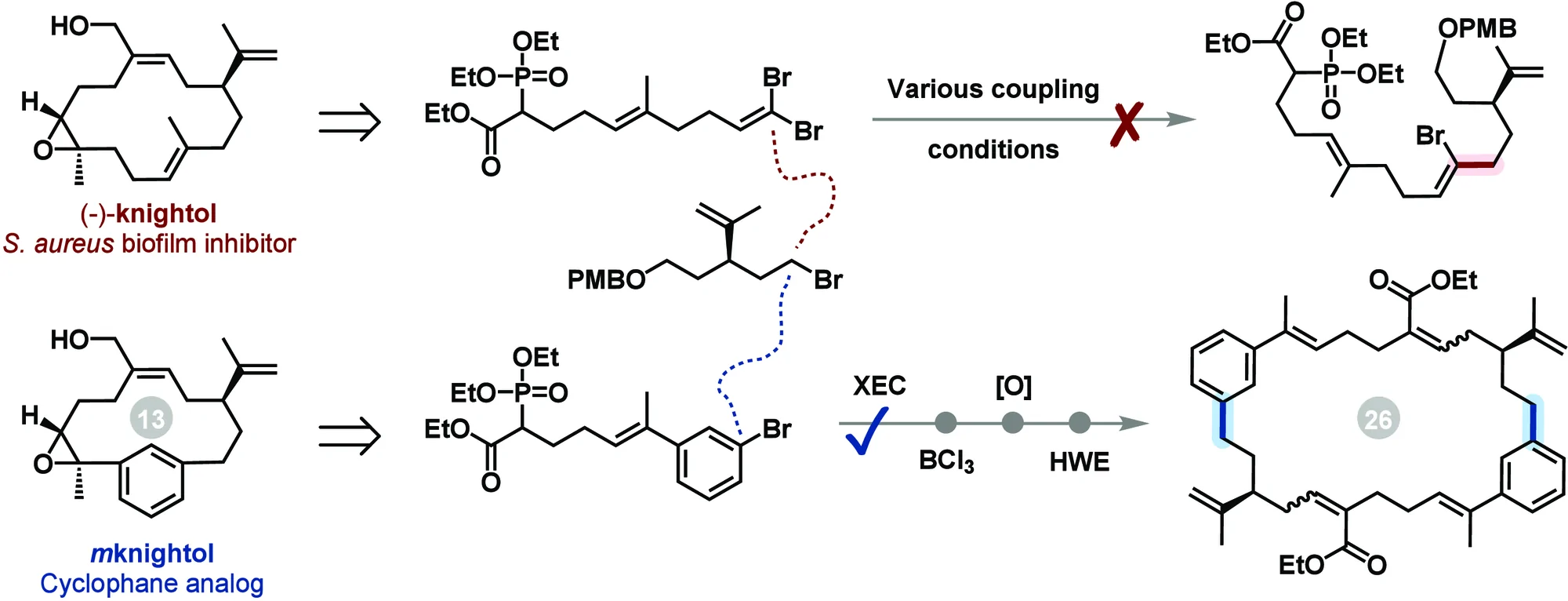

The cembranoid diterpenes (−)-knightol (**1**) and (−)-knightol acetate (**2**) were first isolated in 2009 from the Caribbean Sea Whip, *Eunicea knighti* (Figure 1).^1^ In 2012, these natural products were reisolated by the same group and found to have potent antibiofilm activity against the clinically relevant pathogen *Staphylococcus aureus,* exhibiting MBIC_50_ (Minimum Biofilm Inhibitory Concentrations) values of 0.16 μM and 1.96 μM, respectively.^2^ The known coisolates (−)-asperdiol (**3**) and (−)-asperdiol acetate (**4**), each with a C2 hydroxyl, were found to have comparable activity of 1.56 μM and 0.08 μM, respectively. Notably, the monohydroxylated compounds (−)-knightol (**1**) and (−)-asperdiol acetate (**4**) exhibited submicromolar activity,^2^ making them attractive targets for synthetic, structure–activity relationship, and biological target-identification studies.

**Figure 1:**
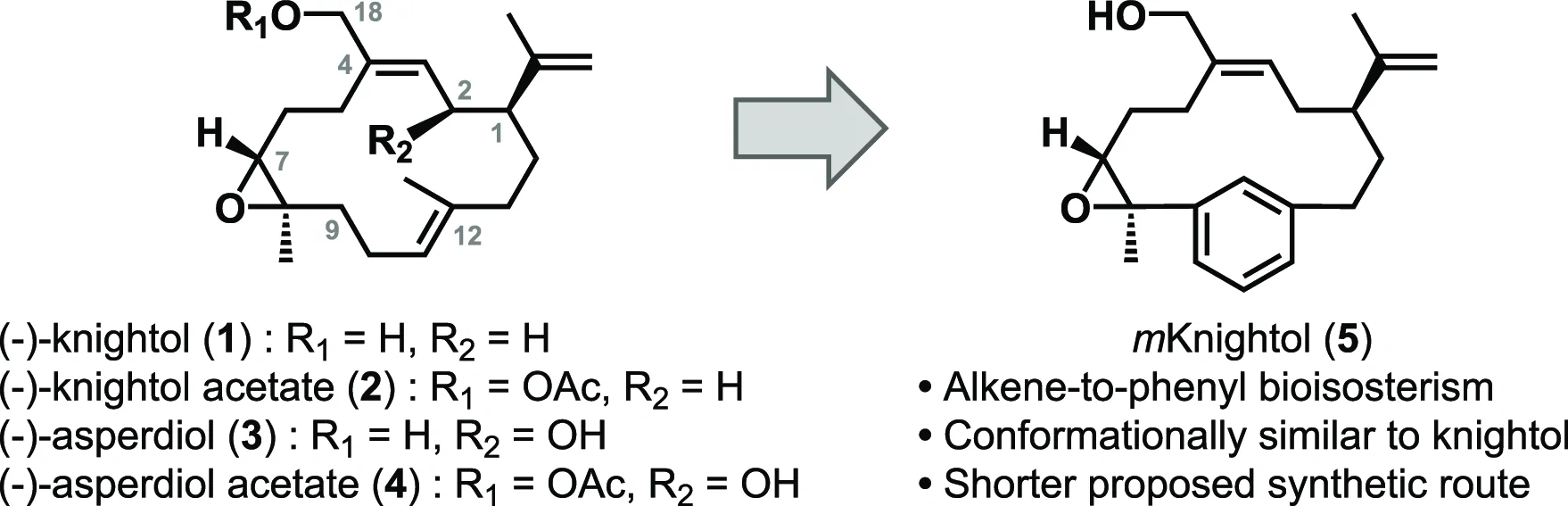
Structures of cembranoid diterpenes and the proposed aryl analog.

Our efforts focused on the novel cembranoid (−)-knightol (**1**), and in our validation studies of the compound, we found that it exhibited moderate activity against laboratory strains of methicillin-susceptible *S. aureus*.^3^ Because the C7–C8 epoxide is conserved across all members of the knightine family,^1,2^ we hypothesized that this functionality serves as a covalent-inhibition warhead and is therefore important for biological activity. In parallel, building on our successful natural product simplification efforts,^4–7^ we sought to enable rapid exploration of the chemical space surrounding (−)-knightol (**1**) through the design of the rigid [10]-metacyclophane analog *m*knightol (**5**). This scaffold was conceived to retain the structural features we propose are critical for activity while providing a synthetically accessible platform for analog development. Additionally, the close structural resemblance of **5** to strained 13-membered natural products, for example furanocembranoids,^8^ inspired the development of a generalized approach to the synthesis of such heterophanes.

Three total syntheses of asperdiol have been reported, highlighting approaches to C7–C8 epoxide installation and macrocyclization.^9–11^ Kato and co-workers first achieved the synthesis of racemic **3** and remain the only group to perform epoxidation after cyclization (Figure 2A).^9^ Central to their strategy was exploitation of the C2 hydroxyl group to mask the C11–C12 alkene of benzoate desepoxyasperdiol (**6**) as a bromoether, thereby enabling selective epoxidation of the C7–C8 double bond to afford **7** in a 3:1 diastereomeric ratio.^9^ In contrast, the Still and Tius groups installed the C7–C8 epoxide at earlier stages of their syntheses using alcohol-directed metal-catalyzed epoxidation and Sharpless asymmetric epoxidation, respectively, while macrocyclization was achieved through Nozaki–Hiyama–Kishi and Horner–Wadsworth–Emmons (HWE) reactions.^10,11^ Similarly, in our completed linear total synthesis of (−)-**1**, we accessed the 14-membered ring after epoxidation, with the C7–C8 oxirane introduced with poor regioselectivity (Figure 2B), thereby reducing overall synthetic efficiency.^3^

**Figure 2:**
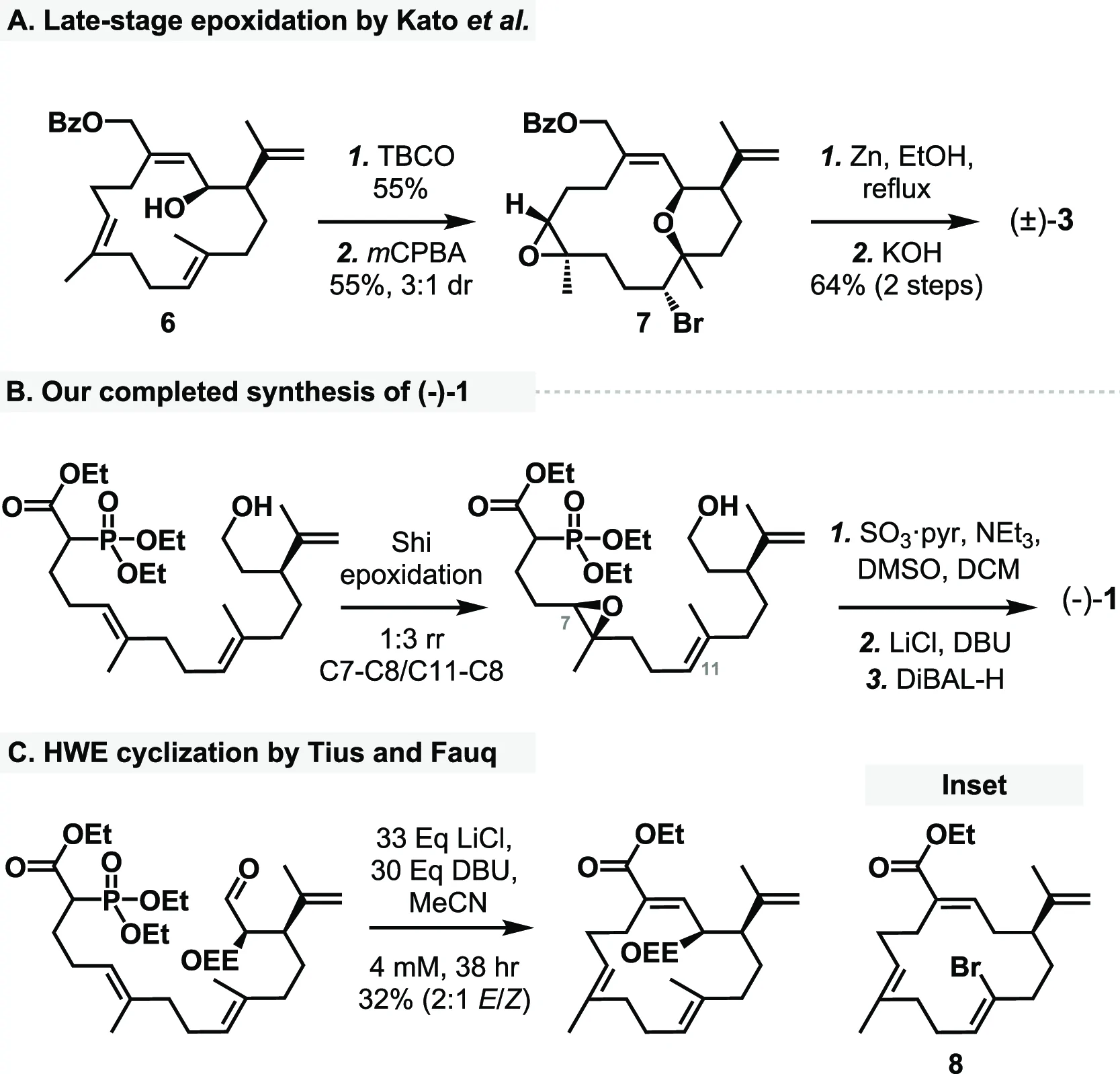
Previous work on cembranoid diterpenes.

Of note, Kato’s late-stage epoxidation strategy inspired three formal syntheses of asperdiol (**3**), in which the desepoxyasperdiol carbocyclic framework was accessed through sulfone-stabilized anion alkylation,^12^ [2,3]-Wittig contraction,^13^ and HWE reaction (Figure 2C).^14^ For the convergent synthesis of (−)-knightol (**1**), we identified alkenyl bromide **8** (Figure 2C, inset) as a key intermediate that could enable selective epoxidation of the C7–C8 alkene following macrocyclization without requiring a C2 hydroxyl group. This intermediate was expected to be accessible through the established HWE macrocyclization strategy, and the absence of C2 substitution in the knightol scaffold was anticipated to facilitate cyclization by reducing steric congestion around the reacting centers. Importantly, beyond deactivating the C11–C12 alkene toward epoxidation, the alkenyl bromide would provide a versatile handle for late-stage diversification via C12 manipulation.

Our retrosynthetic strategy toward (−)-knightol (**1**) and *m*knightol (**5**) is outlined in Figure 3. We envisioned accessing both targets via HWE macrocyclization of the advanced phosphonate–aldehyde intermediate **9**, followed by Shi epoxidation. The macrocyclization precursor would be assembled through coupling of the common eastern fragment (−)-**10** with western fragments. The gem-dibromo alkene **11** and aryl bromide **12** can be traced back to the commercially available dioxolane **13** and 1,3-dibromobenzene **14**, respectively, as well as the common building blocks **15** and **16**.

**Figure 3:**
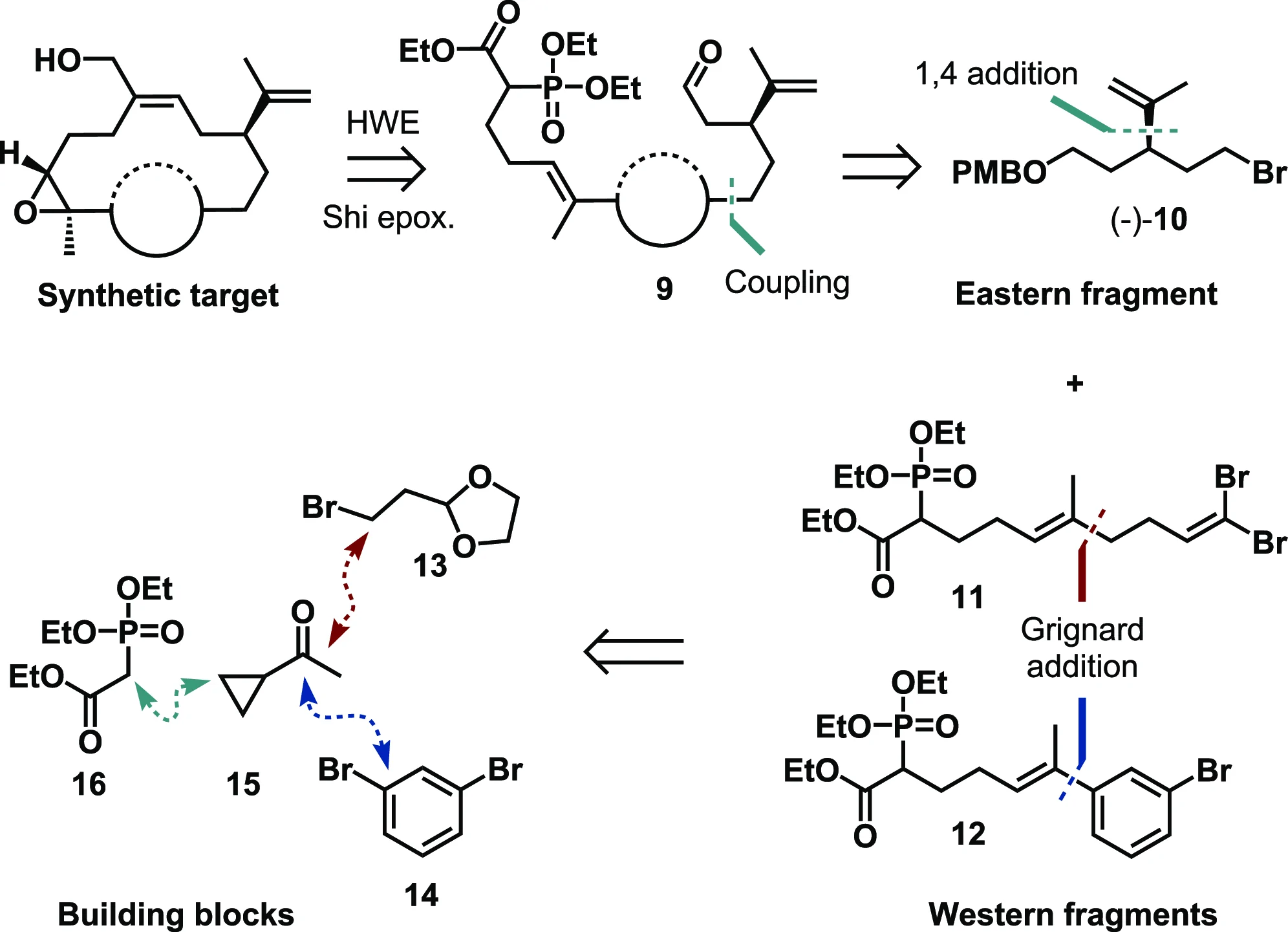
Retrosynthetic analysis.

The synthesis of the eastern fragment commenced from a known four-step sequence to carboxylic acid **18**, starting from 1,3-propanediol **17**, affording the acid in 75% overall yield (Scheme 1).^15^ Installation of the chiral auxiliary **19** under amide coupling conditions provided the α,β-unsaturated oxazolidinone (+)-**20** in 90% yield.^16^ Copper-mediated conjugate addition of isopropenylmagnesium bromide delivered alkene (+)-**21** in 96% yield.^17^ Reduction of (+)-**21** with lithium aluminum hydride proceeded in low yield, attributed to competitive reduction of the oxazolidinone carbonyl, which is more accessible to the hydride reagent than the sterically encumbered amide carbonyl shielded by the phenyl substituent and the newly installed isopropenyl group. In contrast, the use of sodium borohydride furnished (+)-**22** in 78% yield. Subsequent Appel halogenation using N-halosuccinimides provided the eastern fragment as the alkyl bromide (−)-**10** and iodide (−)-**23**.

**Scheme 1:**
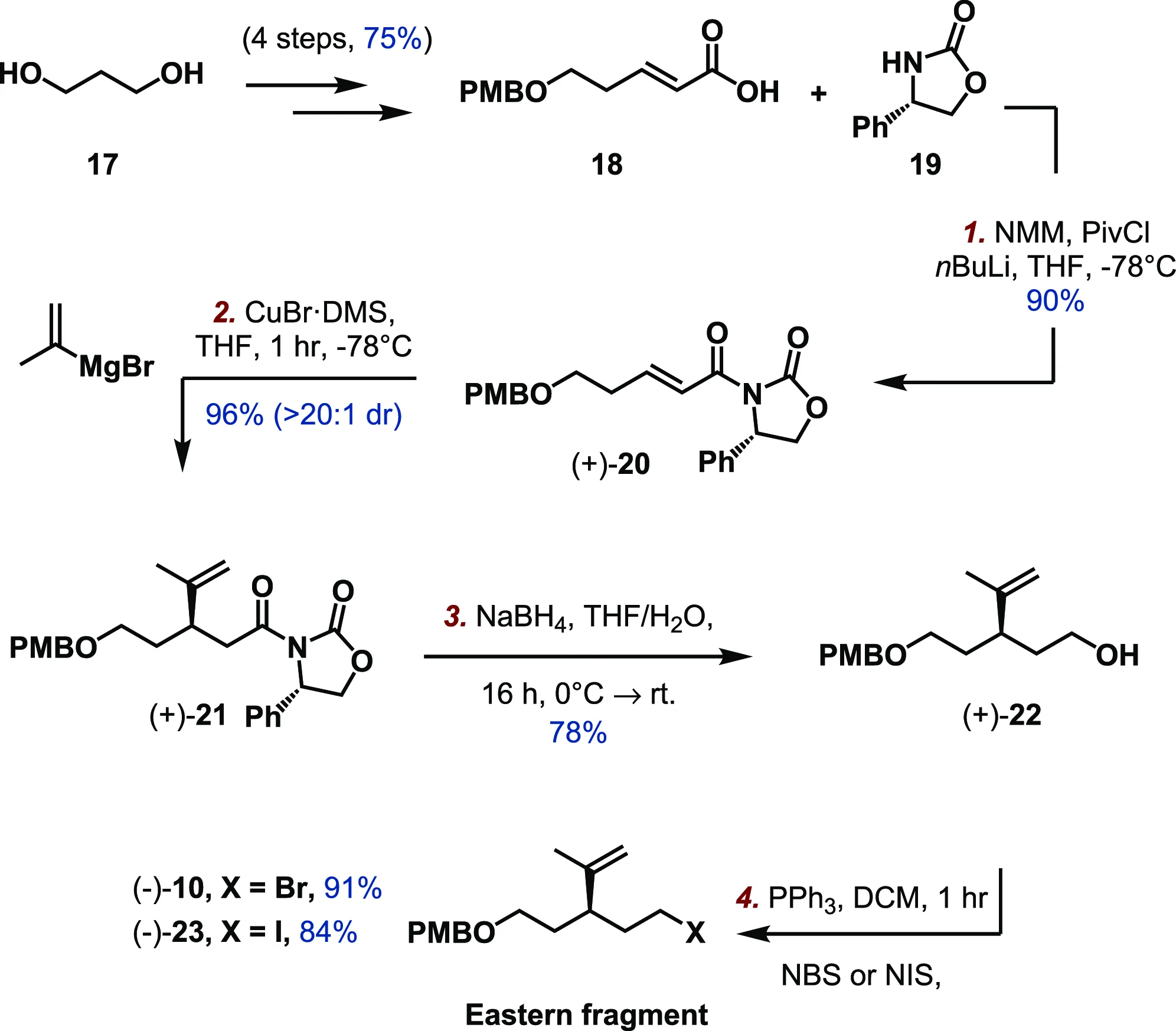
Synthesis of the Eastern Fragment

The elaboration of the western fragment **11** toward (−)-knightol (**1**) began with the formation of a Grignard reagent from **13**, followed by addition to ketone **15**, to afford tertiary alcohol **24** in 68% yield (Scheme 2). Treatment of **24** with ethereal magnesium bromide under reflux afforded the dioxolane **25** in 90% yield, with a 3:1 *E/Z* ratio.^18^ Alkylation of the phosphonate ester **16** was achieved by treatment with potassium tert-butoxide, followed by the addition of **25**. The crude product was stirred with 1 M hydrochloric acid to effect the dioxolane deprotection, affording aldehyde **26**. Finally, Ramirez’s gem-dibromo olefination conditions completed the western fragment in 28% yield over the three transformations.^19,20^

**Scheme 2:**
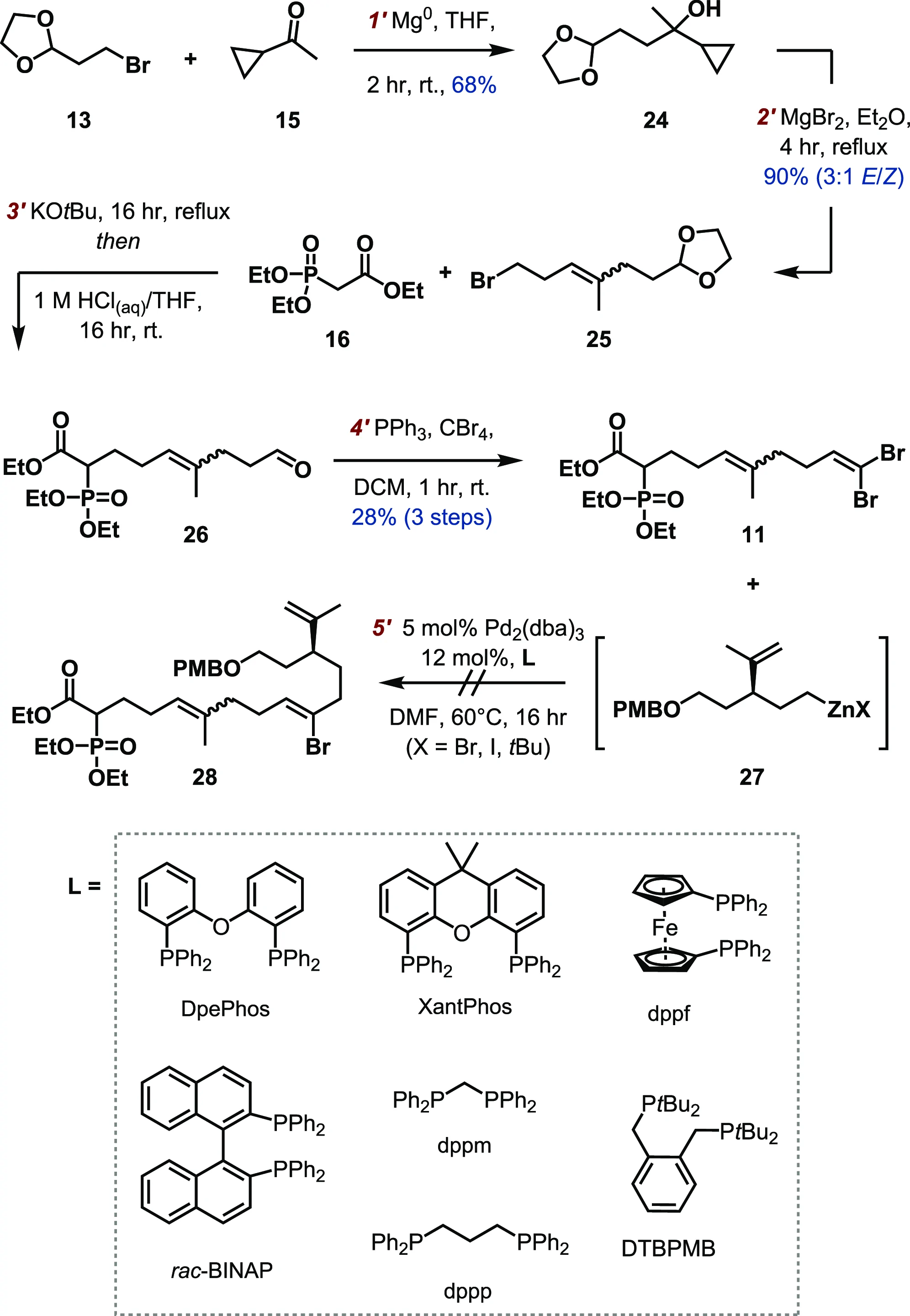
Synthesis of the Western Fragment and Negishi Coupling Studies

With both fragments in hand, we set out to investigate the key fragment coupling step. Two general strategies for alkyl C­(sp^3^)–gem-dichloro C­(sp^2^) bond formation have been reported based on Negishi and Suzuki–Miyaura cross-coupling methodologies.^21,22^ In the case of gem-dichloro substrates, monoalkylation proceeds selectively to give the *Z*-alkene, attributed to steric effects, with bidentate ligands featuring wide bite angles (e.g., DPEPhos and XantPhos) identified as optimal.^21,22^ In contrast, gem-dibromo substrates typically undergo dialkylation due to facile oxidative addition of the second C–Br bond following the initial catalytic cycle.^21^ A notable exception was reported by Uenishi and co-workers, who employed a gem-dibromo substrate under nickel catalysis with dppp and a Grignard reagent as nucleophile.^23^ Given the lower basicity and nucleophilicity of alkylzinc reagents, which would be expected to improve compatibility with phosphonate esters, the Negishi coupling was selected as the most suitable strategy for mild fragment union.

To this end, we first examined the direct formation of organozinc reagents from bromide (−)-**10** or iodide (−)-**23**. Using Huo’s procedure, conversion of (−)-**10** to alkylzinc bromide **27** was sluggish, with incomplete consumption after 6 h in dimethylacetamide (DMA).^24^ In contrast, iodide (−)-**23** underwent efficient metalation to the alkylzinc species in THF, DMF, and DMA. Following a reported Negishi protocol, seven bidentate ligands spanning a broad range of bite angles were evaluated. In all cases, neither the desired product **27** nor the dialkylated byproduct was detected. ^1^H NMR analysis of the crude mixtures revealed only recovered starting material **11** and the proto-dehalogenated alkyl fragment. Under otherwise identical conditions, replacement of Pd_2_(dba)_3_ with NiBr_2_·DME in both DMA and DMF led to the same outcome.

Polar aprotic amide solvents are known to stabilize alkylzinc species, thereby enhancing functional-group tolerance and facilitating their formation.^24^ In this reaction, we hypothesized that such stabilization was counterproductive, as it may attenuate reactivity even at elevated temperatures. Coupling attempts in THF at ambient temperature, 40 °C, and reflux did not afford the desired adduct. We next considered that coordination of the alkylzinc halide to the phosphonate ester could increase the acidity of the α-carbon proton and promote alkylzinc quenching. To address this, dialkylzinc reagents were evaluated, as these are expected to exhibit reduced Lewis acidity relative to alkylzinc halides.^25,26^ The dialkylzinc species was prepared via iodide–lithium (I–Li) exchange followed by transmetalation; however, screening of the same bidentate ligand set in THF at 60 °C again resulted only in recovery of starting material **11**. We then explored whether monodentate ligands might be more effective. Evaluation at room temperature and 60 °C with a ligand set including PPh_3_, tBuBrettPhos, tBuXPhos, JohnPhos, PCy_3_, P­(2-furyl)_3_, BrettPhos, XPhos, CyJohnPhos, and RuPhos, representing a range of electron-rich and sterically demanding Buchwald-type ligands, did not yield the desired product. In all cases, no β-hydride elimination product was detected, suggesting that inefficient transmetalation is the primary limitation in this system.

To improve this step of the catalytic cycle, we next evaluated a more reactive nucleophile, a Grignard reagent, for Kumada coupling. Construction of a compatible western coupling partner began with deprotection of dioxolane **25** to the corresponding aldehyde (Scheme 3A), followed by dibromo olefination to afford **29** in 54% yield over two steps. Preparation of alkylmagnesium reagent **30** was carried out via I–Li exchange of (−)-**23**, followed by transmetalation. Application of the conditions reported by Uenishi and co-workers to the coupling of **29** and **30** resulted in the recovery of starting material **29** and the proto-dehalogenated alkyl fragment.^23^

**Scheme 3:**
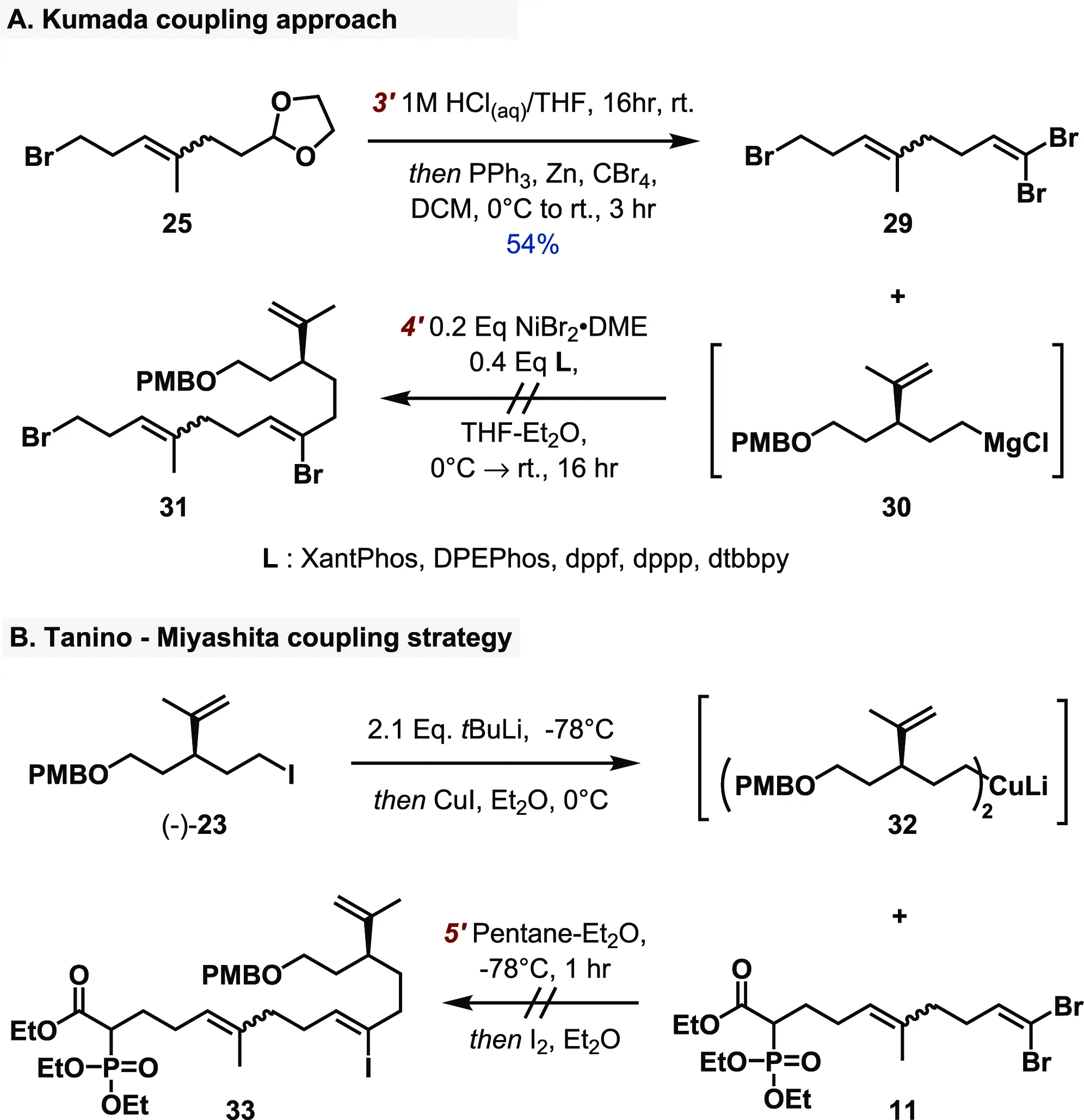
Kumada and Tanino–Miyashita Coupling

To bypass the transmetalation step under catalytic conditions, we sought to expand the scope of the methodology developed by Tanino and co-workers, which couples 1,1-dibromoalkenes with Gilman reagents.^27,28^ Toward (−)-knightol (**1**), the organocuprate **32** was treated with alkene **11**, followed by iodine.^27,28^ Under these conditions, the desired reaction to yield **33** did not occur; however, trace side products resulting from alkylation of the phosphonate-ester were recovered. Given the lack of coupling reactivity between **11** and the chiral alkyl-metal partners, and the functional-group incompatibility, we diverted our attention toward the aryl analog to continue our studies.

Our choice to prioritize *m*knightol (**5**) over the 14-member aryl analogs **34** and **35** (Figure 4) was guided by the high conformational similarity and our ability to establish and preserve the stereochemical integrity of the C7–C8 alkene.^18^ To access the metacyclophane carbocycle, our synthesis began with the construction of the western coupling partner **12** via a three-step sequence shown in Scheme 4. Treatment of 1,3-dibromobenzene **14** with a turbo Grignard reagent, followed by addition to cyclopropyl methyl ketone **15**, and subsequent hydrobromic acid/lithium bromide treatment of the corresponding crude tertiary alcohol, gave the intermediate alkyl bromide in 70% yield as only the *E*-alkene across the eventual C7–C8 position. An analogous sequence en route to **34** gave an 8:1 *E*/*Z* ratio of inseparable diastereomers (see SI scheme S1).^29^ Completion of phosphonate ester **12** was accomplished via alkylation of **16** in 74% yield.

**Figure 4:**
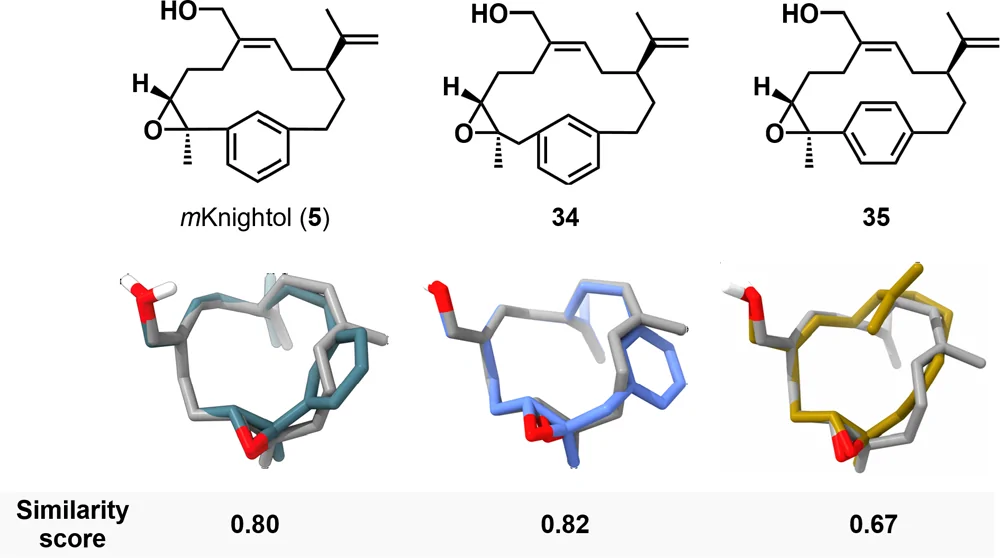
Flexible ligand alignment of (−)-knightol (gray structure) and arylogs **5**, **34**, and **35** giving similarity scores (0 = no match, 1 = identical), performed using default settings after macrocycle conformational search on Maestro interface of Schrödinger Release 2025-2.

**Scheme 4:**
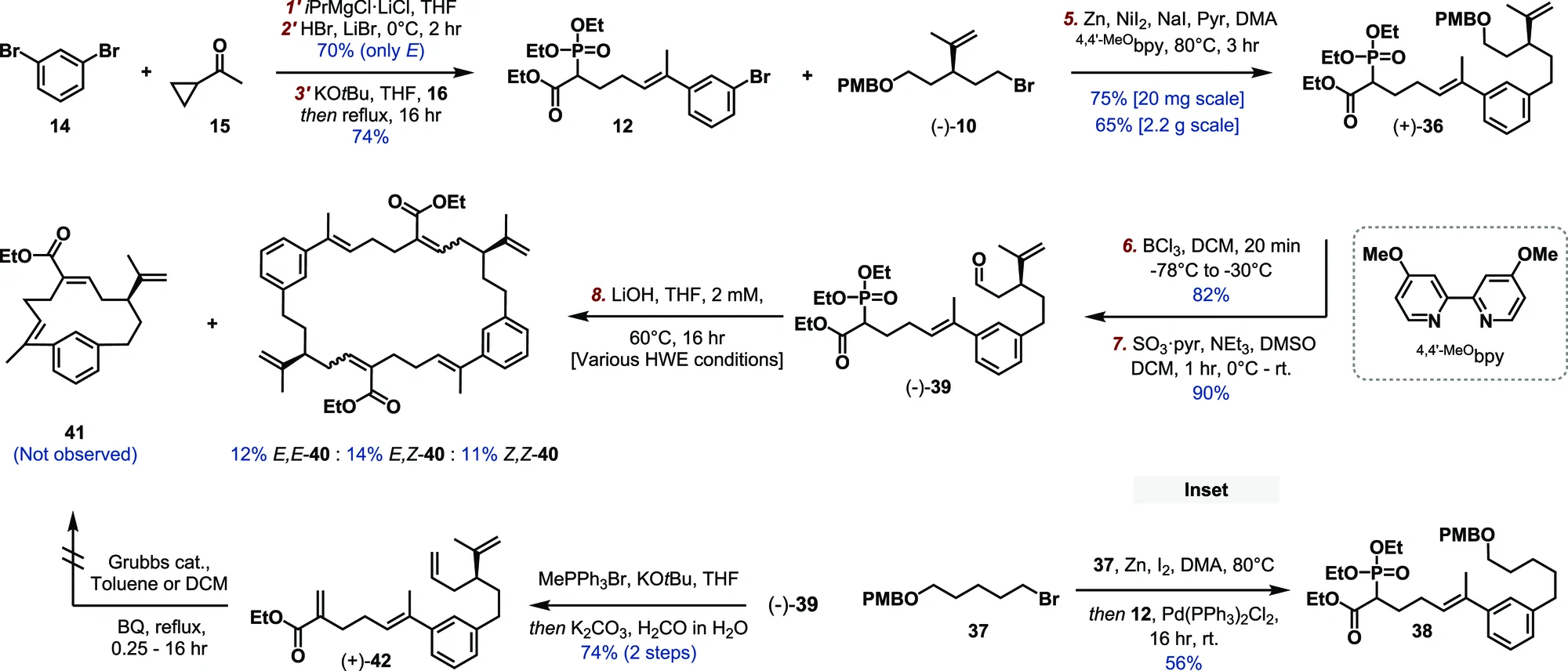
Sequence toward the *m*Knightol Carbocycle

With both fragments in hand, we resumed our coupling studies. Attempts to effect Negishi coupling with this new partner failed to yield the adduct (+)-**36** under multiple conditions. The coupling of the unbranched eastern fragment **37** and **12** proceeded in 56% yield under unoptimized Negishi coupling conditions, affording **38** (Scheme 4, inset).^24^ This result suggests that the isopropenyl substituent impedes coupling with alkyl-metal partners derived from (−)-**10** or (−)-**23**. Gratifyingly, scalable cross-electrophile coupling (XEC) of **12** and (−)-**10** delivered (+)-**36** in 65% yield, along with the homocoupled side products.^30–32^ Attempts to perform XEC on **11** and (−)-**10** resulted in complex mixtures of products, most likely driven by homocoupling of the fragments.

Efforts to remove the PMB protecting group from the model system **38** using traditional oxidative conditions (DDQ or CAN in H_2_O) resulted in decomposition under a range of reaction parameters (see SI Table S1).^33,34^ Successful deprotection was ultimately achieved using BCl_3_, which proved general: application of the same conditions to (+)-**36** afforded the corresponding alcohol in 82% yield (Scheme 4). The HWE precursor (−)-**39** was obtained in 90% yield via Parikh-Doering oxidation, setting the stage for macrocyclization. Attempts to forge the C3–C4 double bond under Masamune-Roush and Helquist HWE conditions resulted in recovery of the starting material (−)-**39** at both room temperature and 60 °C (see SI Table S2).^35,36^ Use of potassium carbonate in refluxing toluene gave trace amounts of the dimer **40**, whereas lithium hydroxide in heated THF furnished the dimeric products exclusively at 37% combined yield. Extensive efforts to promote monocyclization to **41**, including increased dilution (0.2 mM), slow substrate addition over 12 or 24 h, Lewis acid additives (Zn­(OTf)_2_, MgCl_2_, Ti­(OEt)_4_, LiCl), and alternative bases and ethereal solvents (dioxane, DME, DIPE), failed to provide the desired macrocycle **41** (see SI Table S2).

In light of the unsuccessful HWE monomacrocyclization, we next sought to leverage ring-closing metathesis (RCM) as a macrocyclization strategy. A Wittig olefination of (−)-**39** followed by an HWE methenylation with formaldehyde in the same pot furnished tetraene (+)-**42** in 74% yield over two steps. Screening of Grubbs and Hoveyda-Grubbs first- and second-generation catalysts in heated toluene and DCM at reaction times of 0.25, 6, and 16 h failed to yield either **40** or **41**. Instead, transposition of the monosubstituted alkene was consistently observed, despite the inclusion of benzoquinone (BQ).^37^ Additionally, we investigated HWE cyclization of the more flexible phosphonate-aldehyde precursor of aryl analog **34** (see SI Scheme S1), and this approach did not deliver the expected macrocycles. Following these unproductive attempts, further macrocyclization efforts along this pathway were discontinued.

Lastly, the structure of *E,E*-**40** was unambiguously established by X-ray crystallographic analysis (Scheme 5). The C_*2*_-symmetric structure provided an opportunity to investigate the proposed endgame sequence. Exposure of *E,E*-**40** to Shi epoxidation conditions afforded diepoxide **43** and monoepoxide **44** in yields of 21% and 14%, respectively, with 11% recovered starting material (RSM).^38^ Attempts to drive the reaction to completion using excess Oxone resulted in a diminished yield due to overepoxidation. Subsequent treatment of the diepoxide with DIBAL afforded trace amounts of the expected diol as a result of the sensitivity of **43** to the reductant.

**Scheme 5:**
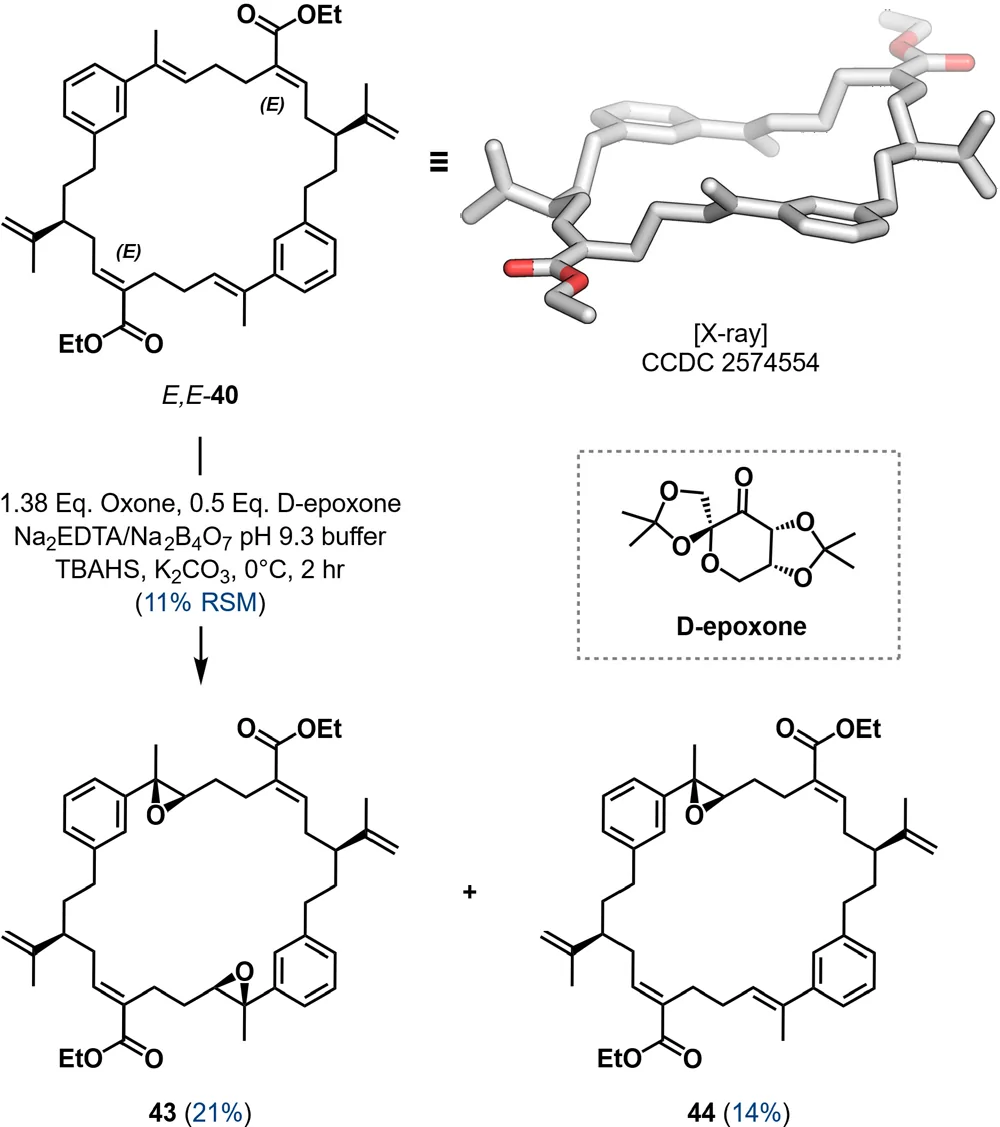
Epoxidation Postcyclization

In summary, key fragments of the cembranoid diterpene (−)-knightol (**1**) and its cyclophane analog, *m*knightol (**5**), were successfully prepared. Attempts to couple the eastern fragment with 1,1-dibromoalkene partners using traditional cross-coupling methodologies were unsuccessful. In contrast, cross-electrophile coupling of the alkyl fragment with a meta-substituted aryl bromide provided access to the advanced cyclization precursor (−)-**39**. Subjecting (−)-**39** to HWE macrocyclization conditions afforded only dimer **40**, despite extensive optimization efforts. These results suggest that the highly strained scaffold **41**, a precursor to *m*knightol (**5**), is not readily accessible through an HWE-based macrocyclization strategy.

## Supplementary Material



## Data Availability

The data underlying this study are available in the published article and its Supporting Information

## Notes

Experimental procedures, characterization data (^1^H, ^13^C­{^1^H}, and ^31^P NMR, HRMS, OR), and X-ray crystallography report (PDF)
