# Supplementary material for: The Convergent Synthetic Efforts toward Knightol and a Simplified Analog
Source: J Org Chem. 2026 Sep 30;91(41):13793–9. doi: 10.1021/acs.joc.6c01838 (PMC13682205; doi:10.1021/acs.joc.6c01838)
Supplement: Supplementary file 1 [file jo6c01838_si_001.pdf]

Supporting Information

The Convergent Synthetic Efforts Toward Knightol and a Simplified Analog

Learnmore Jeremia, Elise L. Bezold, Kate Bright, William M. Wuest*

Department of Chemistry, Emory University, Atlanta, GA 30322, United States.

Email: wwuest@emory.edu

Contents

Instrumentation and General Notes S3
Table S1 PMB deprotection studies S4
Table S2 HWE monocyclization efforts S5
Scheme S1 Efforts towards aryl analog **34** S6
Mono-protection of diol **17** S7
Oxidation of PMB-protected alcohol S8
Horner-Wadsworth-Emmons (HWE) reaction to ester **S3** S9
Saponification of ester **S3** S10
Chiral auxiliary installation S11
Conjugate addition of isopropenyl unit S12
Reduction of oxazolidinone S13
Appel to the alkyl bromide (-)-**10** S14
Appel to alkyl Iodide (-)-**23** S15
Synthesis of tertiary alcohol **24** S16
Preparation of alkyl bromide **25** S17
Synthesis of gem-dibromo alkene **11** S18
Synthesis of gem-dibromo alkene **29** S20
Synthesis of alkylbromide **S4** S21
Synthesis of styrene **12** S22
Cross-electrophile coupling S23
Synthesis of **38** via Negishi coupling S25
BCl_3-mediated PMB Deprotection S26
Parikh-Doering oxidation to access (-)-**39** S27
LiOH promoted HWE macrocyclization S28
Synthesis of Ring Closing Metathesis (RCM) precursor (+)-**42** S29
Epoxidation of E,E-**40** S30
Crystal Data and Experimental S31
References for Supplementary Information S45
NMR spectra of compounds S46

Instrumentation and General Notes

All glassware used was flame-dried and cooled under vacuum. Reactions conducted in non-aqueous solvents were carried out in an Argon atmosphere. Reagents purchased from commercial suppliers (Oakwood Chemical, Sigma-Aldrich, Combi Blocks, AK Scientific, TCI America, Alfa Aesar, or Fisher Scientific) were used without further purification. The following solvents were dried *via* passage through alumina: Tetrahydrofuran (THF) and dichloromethane (DCM). Saturated aqueous solutions of sodium chloride, sodium carbonate, sodium bicarbonate, and ammonium chloride are referred to as brine, sat. Na_2CO_3, sat. $NaHCO_3$, and sat. NH_4Cl respectively. Reaction progress was monitored by analytical thin-layer chromatography (TLC) on EMD Millipore silica gel 60 F254 precoated plates and visualized under UV and/or with ninhydrin or vanillin stains. Automated flash chromatography was performed on Biotage Isolera One instruments using Siliaflash silica gel (40-63 μm). Optical rotations were measured using an Autopol IV (Rudolph Research Analytical) polarimeter (concentration, *c*, in g/100mL). Accurate mass spectra were recorded on a Thermo Scientific™ Exactive Plus Orbitrap LTQ-FTMS using atmospheric pressure chemical ionization (APCI) and Electrospray Ionization (ESI).

^{1}H, ^{13}C, and ^{31}P spectra were acquired using the following spectrometers: Bruker Avance NEO800 (800 MHz), Bruker Avance III HD 600, Varian INOVA 400, or ADVANCE III 400. The NMR samples were prepared in deuterated solvent chloroform ($CDCl_3$) using residual solvent peaks ($CDCl_3$: ^{1}H = 7.26 ppm, ^{13}C = 77.16 ppm or TMS: ^{1}H = 0.00 ppm, ^{13}C = 0.00 ppm) as internal references. The proton NMR chemical shifts (δ) are reported in ppm, multiplicities are reported as s (singlet), d (doublet), t (triplet), q (quartet), p (quintet), m (multiplet or unresolved), and the corresponding coupling constant (J) in Hz.

Table S1 *PMB deprotection studies*

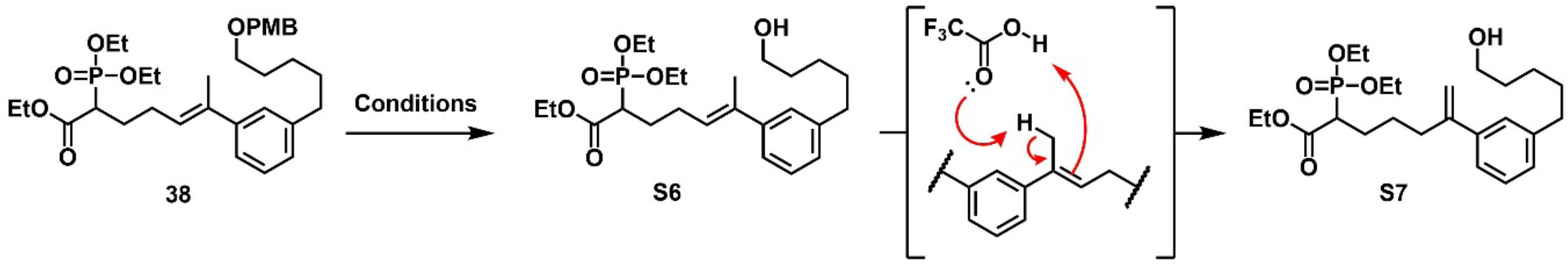

Entry	Conditions	Yield (%)
1	DDQ, MeCN, pH 7 Buffer or Water, 0°C → rt., 0.25 - 2 hr	**decomp.**
2	CAN, MeCN/Water, 0°C → rt., 0.25 - 2 hr	**decomp.**
3	$FeCl_3$, DCM, rt. 2 hr	**NR**
4	$ZrCl_4$, MeCN, rt., 2 hr	**NR**
5	$POCl_3$, DCE, rt., 16 hr	**Trace**
6	Oxalyl chloride, DCE, rt., 2 hr	**NR**
7	tButylBr, MeCN, reflux, 16 hr	**decomp.**
8	TFA (10% v/v), DCM, rt., 2 hr	**83% (1:1.7 | S6/S7)**
9	BCl_3, DCM, -78 → -30°C, 20 min	**84**

Table S2 *HWE monocyclization efforts*

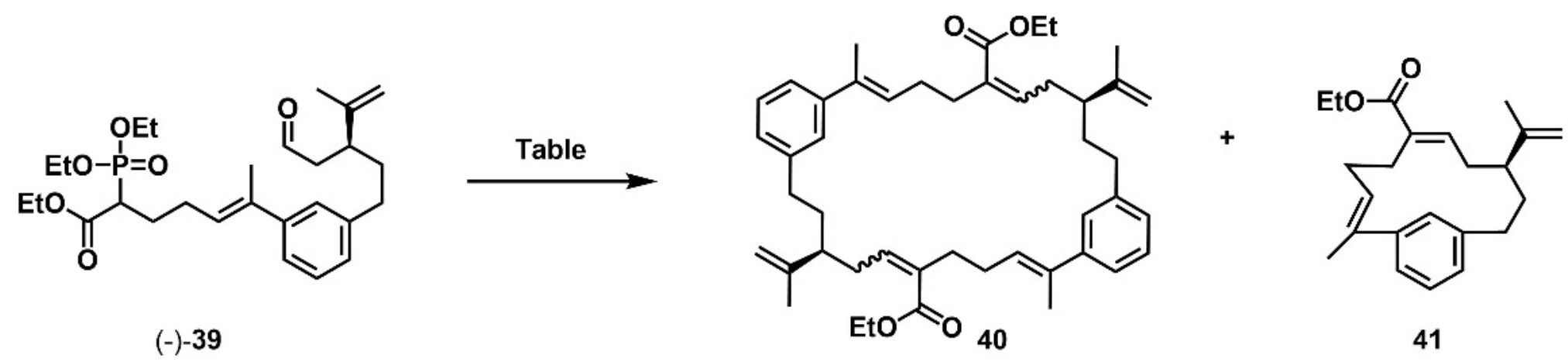

(-)-**39** **40** **41**

	Entry	Conditions	Result
Initial screen	**1**	5 Eq DBU, 10 Eq LiCl, MeCN, 2 mM, 16 hr, rt.	NR
	**2**	5 Eq DBU, 10 Eq LiCl, THF, 2 mM, 16 hr, rt.	NR
	**3**	5 Eq DBU, 10 Eq LiCl, MeCN, 0.5 mM, 16 hr, 60°C	NR
	**4**	5 Eq TEA, 1.5 Eq TMEDA, 1.5 Eq $Zn(OTf)_2$, THF, 2 mM, 16 hr, 60°C	NR
	**5**	5 Eq K_2CO_3, 10 Eq 18-Crown-6, Tol, 2 mM, 16 hr, reflux	**39** + **40** (trace)
	**6**	5 Eq LiOH, THF, 2 mM, 16 hr, 60°C	**40**
Slow addition	**7**	200 mM LiOH, THF, 2 mM, 16 hr, 60°C	**40**
	**8**	200 mM LiOH, THF, 2 mM, 12 hr add'n time, 16 hr, 60°C	**40**
	**9**	200 mM LiOH, THF, 2 mM, 24 hr add'n time, 16 hr, 60°C	**40**
	**10**	200 mM LiOH, THF, 0.2 mM, 24 hr add'n time, 16 hr, 60°C	NR
Lewis acid additives	**11**	200 mM LiOH, 200 mM $Zn(OTf)_2$, THF, 2 mM, 16 hr, 60°C	**40**
	**12**	200 mM LiOH, 200 mM $MgCl_2$, THF, 2 mM, 16 hr, 60°C	**40**
	**13**	200 mM LiOH, 200 mM $Ti(OEt)_4$, THF, 2 mM, 16 hr, 60°C	**40**
	**14**	200 mM LiOH, 200 mM LiCl, THF, 2 mM, 16 hr, 60°C	**40**
Hydroxide bases	**15**	200 mM KOH, THF, 2 mM, 16 hr, 60°C	decomp
	**16**	200 mM NaOH, THF, 2 mM, 16 hr, 60°C	**40** (trace)
	**17**	200 mM NaOH, 200 mM LiCl, THF, 2 mM, 16 hr, 60°C	**40**
Alkoxide bases	**18**	200 mM NaOMe, 200 mM LiCl, THF, 2 mM, 16 hr, 60°C	**39** + **40** (trace)
	**19**	200 mM NaOEt, 200 mM LiCl, THF, 2 mM, 16 hr, 60°C	complex mixture
	**20**	200 mM NaOtBu, 200 mM LiCl, THF, 2 mM, 16 hr, 60°C	NR
	**21**	200 mM LiOMe, THF, 2 mM, 16 hr, 60°C	**40**
DBU & solvent	**22**	200 mM DBU, 200 mM LiCl, MeCN, 2 mM, 16 hr, 60°C	**39** + **40** (trace)
	**23**	200 mM DBU, 200 mM LiCl, THF, 2 mM, 16 hr, 60°C	**40**
Ethereal solvents	**24**	200 mM LiOH, 200 mM LiCl, Dioxane, 2 mM, 16 hr, 60°C	decomp + **40** (trace)
	**25**	200 mM LiOH, 200 mM LiCl, DME, 2 mM, 16 hr, 60°C	**S8**
	**26**	200 mM LiOH, 200 mM LiCl, DIPE, 2 mM, 16 hr, 60°C	**40**

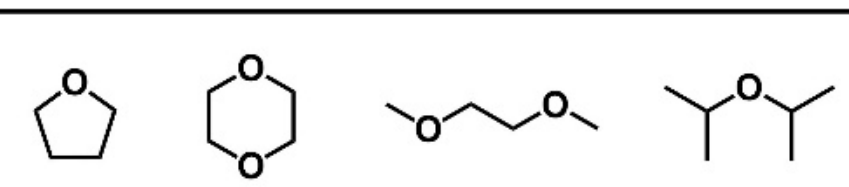

THF **Dioxane** **DME** **DIPE**

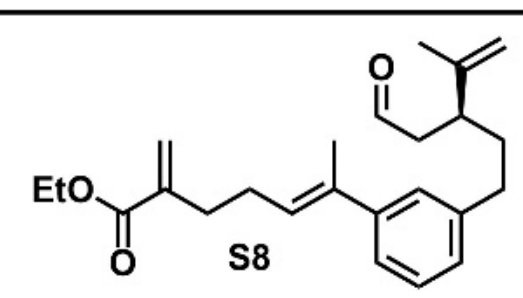

S8

Scheme S1** Efforts towards aryl analog **34

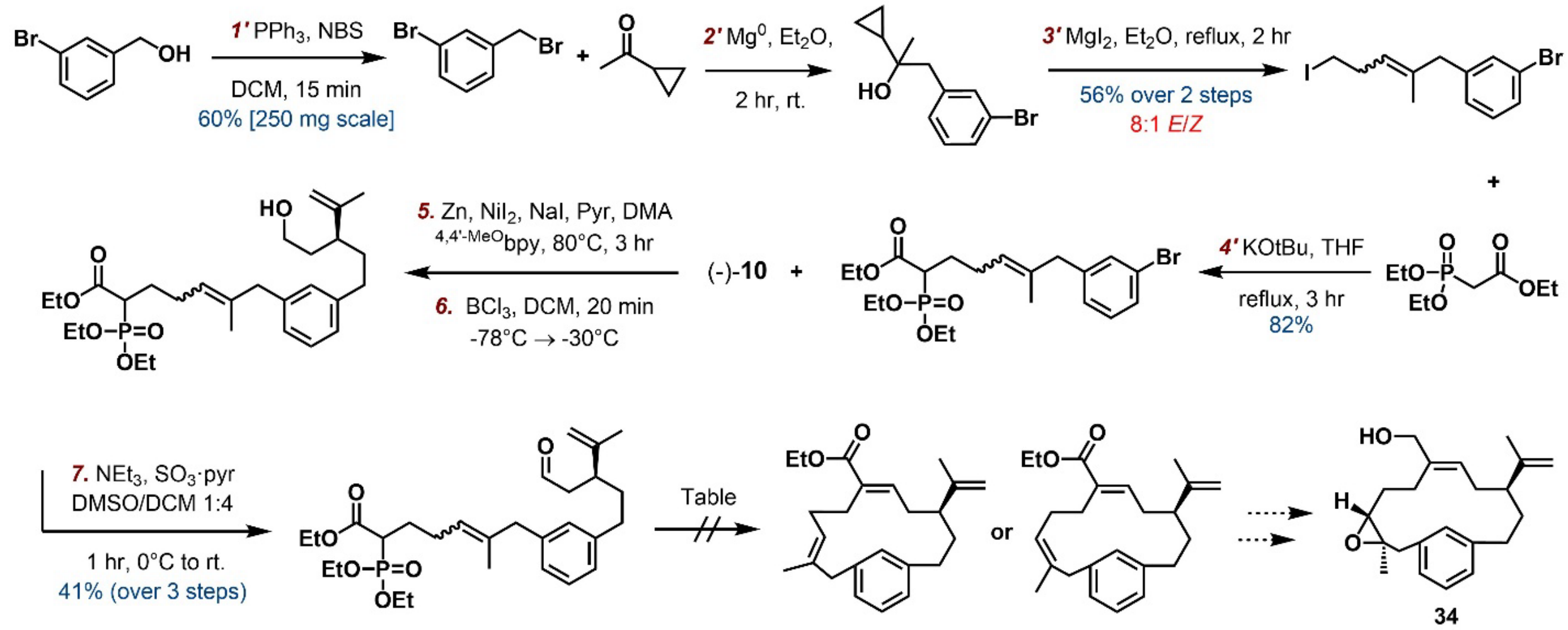

Entry	Conditions	Result
1	10 Eq. LiCl, 5 Eq. DBU, 1 mM, MeCN, rt., 48 hr	NR
2	10 Eq. LiCl, 5 Eq. DBU, 10 mM, MeCN, rt., 16 hr	NR
3	10 Eq. LiCl, 5 Eq. DBU, 10 mM, MeCN, 60°C, 16 hr	decomp.
4	100 Eq. LiOH, 1 mM, THF, 60°C, 16 hr	decomp.

*Mono-protection of diol **17***

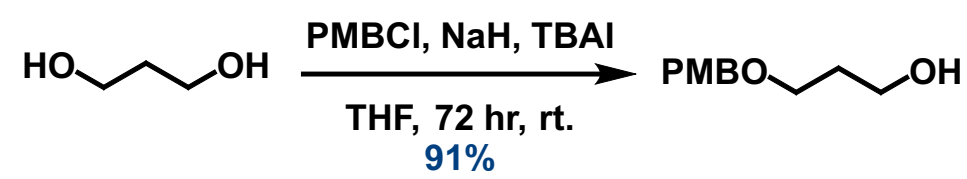

3-((4-methoxybenzyl)oxy)propan-1-ol (**S1**): To a suspension of Sodium hydride (4.29 g, 60% Wt, 3 Eq, 107.1 mmol) in dry THF (257 mL) was added TBAI (1.32 g, 0.10 Eq, 3.57 mmol) at 0°C, and the mixture was stirred for 5 min. A solution of 1,3-propanediol **17** (10.9 g, 10 mL, 4 Eq, 142.8 mmol) in THF (32 mL) was then added at 0°C, and the mixture was warmed to room temperature for 30 min. After 30 min, the reaction mixture was again cooled to 0°C, and PMBCl (5.59 g, 4.8 mL, 1 Eq, 35.7 mmol) was added slowly with additional stirring for 72 hr. Finally, the reaction was quenched by the addition of ice water. The aqueous phase was separated and extracted with EtOAc (3 × 100 mL). The combined organic layers were washed with water (100 mL) and brine (100 mL), dried over anhydrous Na_2SO_4, and concentrated *in vacuo*. The residue was purified by flash chromatography (silica gel, 33% EtOAc in hexanes) to give the alcohol **S1** (6.34 g, 32.3 mmol, yield 91%) as a colorless liquid

[**Caution!** Sodium hydride (NaH) is a highly reactive, flammable solid that violently releases explosive hydrogen gas upon contact with water or moisture. Avoid contact with skin as it causes severe burns and irritation.]

^{1}H NMR (600 MHz, $CDCl_3$) δ 7.27 – 7.23 (m, 2H), 6.90 – 6.85 (m, 2H), 4.45 (s, 2H), 3.80 (s, 3H), 3.77 (t, J = 5.7 Hz, 2H), 3.63 (t, J = 5.8 Hz, 2H), 1.85 (p, J = 5.7 Hz, 2H).

^{13}C{^{1}H} NMR (151 MHz, $CDCl_3$) δ 159.4, 130.3, 129.4, 114.0, 73.1, 69.3, 62.1, 55.4, 32.2.

R_f: 0.33 (1:1 EtOAc/Hex, $KMnO_4$ stain)

Spectral data are consistent with the reported literature.[1]

Oxidation of PMB-protected alcohol

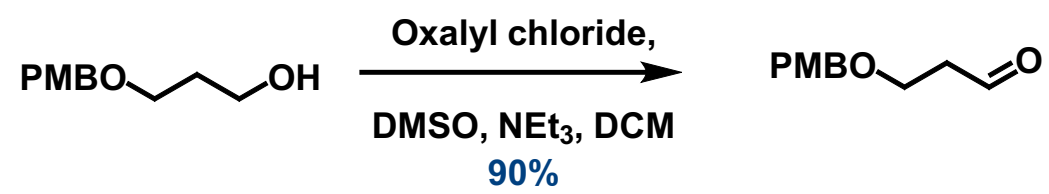

3-((4-methoxybenzyl)oxy)propanal (**S2**): A solution of DMSO (7.17 g, 6.5 mL, 3.0 Eq, 91.7 mmol) in DCM (96 mL) was added to a solution of Oxalyl chloride (5.82 g, 4.0 mL, 1.5 Eq, 45.9 mmol) in DCM (96 mL) at -78°C. After the mixture was stirred for 30 min, a solution of **S1** (6.00 g, 1 Eq, 30.6 mmol) in DCM (96 mL) was added to the above solution at -78°C, and the mixture was stirred at the same temperature for 1 hr. Triethylamine (17.0 mL, 4.0 Eq, 122.3 mmol) was then added to the reaction mixture, which was gradually warmed to room temperature and stirred for 2 hr. The reaction mixture was washed with water and brine, dried over anhydrous Na_2SO_4, and concentrated *in vacuo*. The crude product was subjected to flash column chromatography (silica gel, 20% EtOAc in hexanes) to afford the title compound (5.36 g, 27.6 mmol, yield 90%) as a colorless oil.

[**Caution!** Oxalyl chloride is a severely corrosive and toxic chemical that reacts violently with water to release flammable and toxic gases.]

^{1}H NMR (600 MHz, $CDCl_3$) δ 9.78 (t, J = 1.8 Hz, 1H), 7.27 – 7.22 (m, 2H), 6.89 – 6.86 (m, 2H), 4.46 (s, 2H), 3.80 (s, 3H), 3.78 (t, J = 6.1 Hz, 2H), 2.68 (td, J = 6.1, 1.9 Hz, 2H).

^{13}C{^{1}H} NMR (151 MHz, $CDCl_3$) δ 201.4, 159.4, 130.1, 129.5, 114.0, 73.1, 63.7, 55.4, 44.0.

R_f: 0.65 (1:1 EtOAc/Hex, $KMnO_4$ stain)

Spectral data are consistent with the reported literature.[1]

*Horner-Wadsworth-Emmons (HWE) reaction to ester **S3***

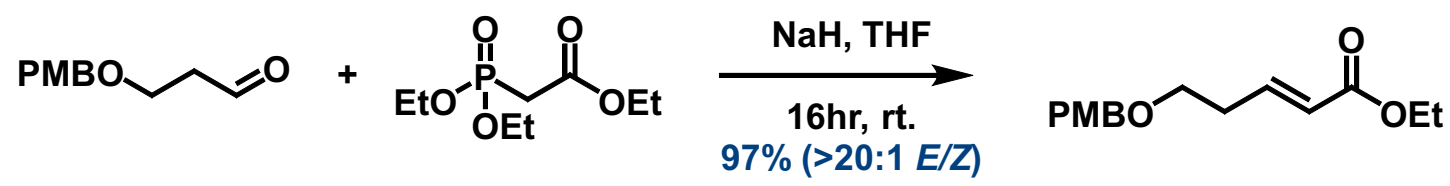

ethyl (E)-5-((4-methoxybenzyl)oxy)pent-2-enoate (**S3**): To a suspension of Sodium hydride (726 mg, 60% Wt, 1.5 Eq, 18.2 mmol) in THF (57 mL) was added phosphonate ester **16** (3.84 mL, 1.6 Eq, 19.4 mmol). The mixture was stirred for 30 min at room temperature, then cooled to 0°C, and aldehyde **S2** (2.35 g, 1 Eq, 12.1 mmol) in THF (19 mL) was added. The reaction mixture was allowed to stir for 30 min and then quenched with sat. $NH_4Cl_{(aq)}$ (50 mL), extracted with EtOAc (100 mL), washed with brine, and dried over anhydrous Na_2SO_4. The solvent was removed *in vacuo* and the crude was purified by flash chromatography (silica gel, 0 – 25% EtOAc in hexanes) to afford **S3** (3.09 g, 11.7 mmol, yield 97 %, >20:1 *E/Z*).

^{1}H NMR (600 MHz, $CDCl_3$) δ 7.27 – 7.23 (m, 2H), 6.96 (dt, *J* = 15.7, 6.9 Hz, 1H), 6.90 – 6.86 (m, 2H), 5.88 (dt, *J* = 15.7, 1.6 Hz, 1H), 4.45 (s, 2H), 4.18 (q, *J* = 7.1 Hz, 2H), 3.80 (s, 3H), 3.55 (t, *J* = 6.5 Hz, 2H), 2.49 (qd, *J* = 6.6, 1.6 Hz, 2H), 1.28 (t, *J* = 7.1 Hz, 3H).

^{13}C{^{1}H} NMR (151 MHz, $CDCl_3$) δ 166.6, 159.4, 145.8, 130.3, 129.5, 123.0, 113.9, 72.8, 68.1, 60.3, 55.4, 32.8, 14.4.

R_f: 0.63 (1:2 EtOAc/Hex, $KMnO_4$ stain)

Spectral data are consistent with the reported literature.[1]

*Saponification of ester **S3***

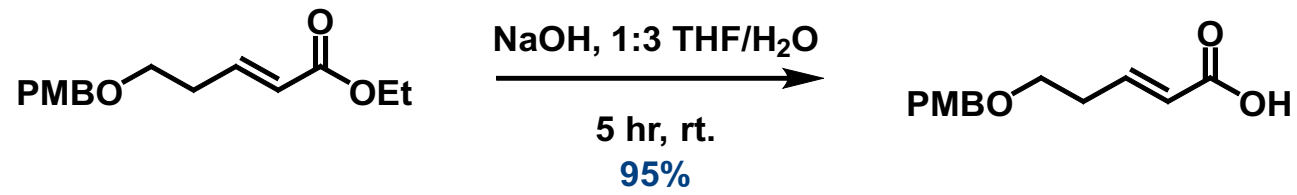

(E)-5-((4-methoxybenzyl)oxy)pent-2-enoic acid (**18**): A solution of ester **S3** (2.70 g, 1 Eq, 10.2 mmol) in THF (4.8 mL) and Water (15.3 mL) was treated with Sodium hydroxide (613 mg, 1.5 Eq, 15.3 mmol). The mixture was stirred at room temperature overnight and diluted with Et_2O (50 mL). The layers were separated, and the aqueous layer was acidified with 6 M HCl and extracted again with Et_2O (6 × 20 mL). The combined extracts were dried with anhydrous Na_2SO_4, filtered, and concentrated. The crude product was washed with diisopropyl ether to give the carboxylic acid **18** (2.29 g, 9.7 mmol, yield 95%) as white crystals.

^{1}H NMR (600 MHz, $CDCl_3$) δ 7.28 – 7.23 (m, 2H), 7.09 (dt, J = 15.7, 6.9 Hz, 1H), 6.90 – 6.87 (m, 2H), 5.90 (dt, J = 15.7, 1.6 Hz, 1H), 4.46 (s, 2H), 3.81 (s, 3H), 3.57 (t, J = 6.4 Hz, 2H), 2.53 (qd, J = 6.5, 1.6 Hz, 2H).

^{13}C{^{1}H} NMR (151 MHz, $CDCl_3$) δ 171.5, 159.4, 148.8, 130.2, 129.5, 122.3, 114.0, 72.9, 67.9, 55.4, 32.9.

Spectral data are consistent with the reported literature.[1]

Chiral auxiliary installation

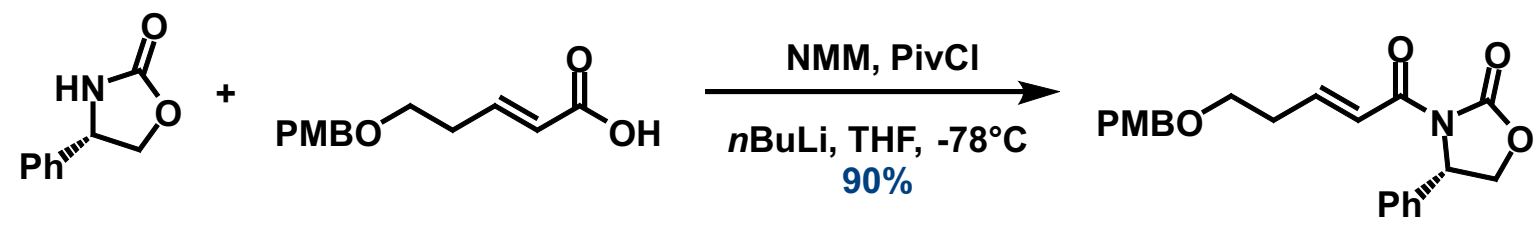

(*S*,*E*)-3-(5-((4-methoxybenzyl)oxy)pent-2-enoyl)-4-phenyloxazolidin-2-one ((+)**-20**): To a solution of the carboxylic acid **18** (1.91 g, 1 Eq, 8.07 mmol) in anhydrous THF (22 mL) containing N-methylmorfoline (0.9 mL, 1 Eq, 8.07 mmol), was added pivaloyl chloride (1.0 mL, 1 Eq, 8.07 mmol) at 0 ˚C under argon, and the mixture was stirred at –78 ˚C for 1 hr. To this was added by cannula, a cooled solution of lithium (*S*)-4-phenyl-2-oxazolidinone, prepared through addition of n-BuLi (3.9 mL, 2.5 molar, 1.2 Eq, 9.68 mmol) to a suspension of (*S*)-(+)-4-phenyl-2-oxazolidinone (**19**) (1.58 g, 1.2 Eq, 9.68 mmol) in anhydrous THF (22 mL) at –78 ˚C under argon with stirring for 30 min in a separate flask. After stirring at –78 ˚C for 2 hr, the solution was warmed to room temperature and stirred overnight. To the mixture was added 100 mL of saturated NH_4Cl; THF was evaporated under reduced pressure, and the residue was extracted with ethyl acetate, washed with H_2O and saturated brine, and dried over Na_2SO_4. Concentration and purification by silica gel chromatography (0 – 25% EtOAc in hexanes, gradient) afforded oxazolidinone (+)**-20** (2.77 g, 7.26 mmol, yield 90%) as a colorless viscous oil.

[**Caution!** n-Butyllithium (n-BuLi) is an extremely reactive, pyrophoric, and corrosive chemical that catches fire spontaneously in air and reacts violently with water.]

^{1}H NMR (600 MHz, $CDCl_3$) δ 7.40 – 7.36 (m, 2H), 7.35 – 7.30 (m, 4H), 7.27 – 7.22 (m, 2H), 7.10 (dt, *J* = 15.4, 6.9 Hz, 1H), 6.88 – 6.85 (m, 2H), 5.48 (dd, *J* = 8.7, 3.8 Hz, 1H), 4.70 (t, *J* = 8.8 Hz, 1H), 4.44 (s, 2H), 4.28 (dd, *J* = 8.9, 3.9 Hz, 1H), 3.80 (s, 3H), 3.56 (t, *J* = 6.5 Hz, 2H), 2.56 (qd, *J* = 6.6, 1.6 Hz, 2H).

^{13}C{^{1}H} NMR (151 MHz, $CDCl_3$) δ 164.5, 159.4, 153.8, 148.6, 139.2, 130.3, 129.5, 129.3, 128.8, 126.1, 121.7, 113.9, 72.9, 70.1, 68.1, 57.9, 55.4, 33.2.

$[\alpha]_D^{22}$: + 101.8 (*c* 0.50, MeOH)

R_f : 0.68 (1:1 EtOAc/Hex, $KMnO_4$ stain)

HRMS (ESI+): calc. 404.1474 for $C_{22}H_{23}O_5N^{23}Na$ $[M + Na]^+$, found 404.1475

Conjugate addition of isopropenyl unit

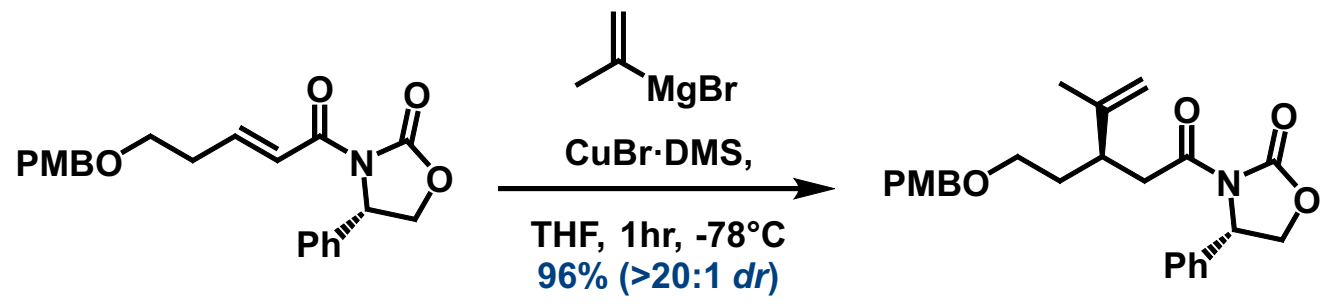

(*S*)-3-((*R*)-3-(2-((4-methoxybenzyl)oxy)ethyl)-4-methylpent-4-enoyl)-4-phenyloxazolidin-2-one ((+)-**21**): Recrystallized copper(I) bromide dimethyl sulfide complex (70 mg, 1.3 Eq, 341 µmol) was dispersed in THF (1.4 mL), and the mixture was cooled to –78 °C. Isopropenyl magnesium bromide (680 µL, 0.5 molar, 1.3 Eq, 341 µmol) was added dropwise, and the dark red solution was stirred at –78 °C for 70 min. Then, the oxazolidinone (+)-**20** (100 mg, 1 Eq, 262 µmol) dissolved in THF (0.9 mL) was added dropwise, and the reaction mixture was stirred for 1 hr. Saturated $NH_4Cl_{(aq)}$ (10 mL) solution was added, and the mixture was allowed to warm to room temperature. The aqueous layer was extracted with EtOAc (3 × 20 mL). The combined organic layers were dried over $MgSO_4$ and concentrated under reduced pressure, then purified via flash column chromatography on silica gel (0 – 60% EtOAc in hexanes, gradient) to obtain alkene (+)**-21** (107 mg, 252 µmol, yield 96%, >20:1 dr) as a colorless viscous oil.

[**Caution!** Isopropenyl magnesium bromide is a highly dangerous, flammable chemical. It reacts with water to release flammable gases that ignite spontaneously]

^{1}H NMR (600 MHz, $CDCl_3$) δ 7.37 – 7.29 (m, 3H), 7.27 – 7.22 (m, 4H), 6.90 – 6.84 (m, 2H), 5.36 (dd, *J* = 8.7, 3.7 Hz, 1H), 4.71 – 4.66 (m, 2H), 4.58 (t, *J* = 8.8 Hz, 1H), 4.41 – 4.34 (m, 3H), 4.21 (dd, *J* = 8.9, 3.7 Hz, 1H), 3.80 (s, 3H), 3.43 – 3.34 (m, 2H), 3.08 (dd, *J* = 16.0, 7.5 Hz, 1H), 3.02 (dd, *J* = 16.0, 7.1 Hz, 1H), 2.79 (dtd, *J* = 8.9, 7.3, 5.5 Hz, 1H), 1.75 – 1.65 (m, 2H), 1.64 (s, 3H).

^{13}C{^{1}H} NMR (151 MHz, $CDCl_3$) δ 171.5, 159.2, 153.8, 146.3, 139.2, 130.8, 129.3, 129.2, 128.7, 126.0, 113.8, 112.2, 72.6, 70.0, 68.2, 57.8, 55.4, 40.1, 39.6, 32.9, 19.4.

$[\alpha]_D^{21}$: + 90.9 (*c* 0.50, MeOH)

R_f : 0.73 (1:1 EtOAc/Hex, $KMnO_4$ stain)

HRMS (APCI+): calc. 424.2118 for $C_{25}H_{30}NO_5$ $[M + H]^+$, found 424.2125

Reduction of oxazolidinone

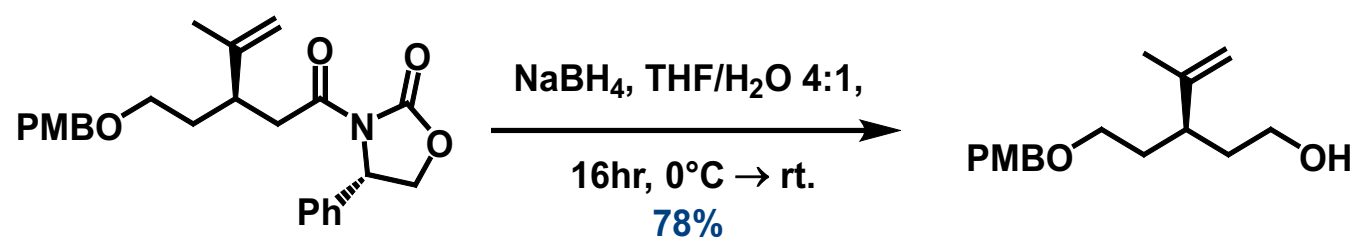

(S)-3-(2-((4-methoxybenzyl)oxy)ethyl)-4-methylpent-4-en-1-ol ((+)-**22**): Oxazolidinone (+)-**21** (105 mg, 1 Eq, 248 µmol) in 3:1 THF/H_2O co-solvent was treated with Sodium borohyhdride (38 mg, 4 Eq, 992 µmol) at 0°C then stirred at room temperature until TLC indicated full conversion (~72 hr). The resulting reaction mixture was quenched with sat. $NH_4Cl_{(aq)}$ until effervescence ceased, extracted with ethyl acetate (3 x 10 mL), washed with $NaHCO_3$ solution, and brine, then dried over Na_2SO_4. The crude product was purified using silica gel chromatography (0% – 25% EtOAc in hexanes, gradient) to give the alcohol (+)-**22** (51 mg, 0.19 mmol, yield 78%) as a colorless oil. (76% yield on a 6.7 g scale of (+)-**21**)

[**Caution!** Sodium borohydride ($NaBH_4$) is highly reactive, corrosive, and flammable. When it contacts water, acids, or oxidizing agents, it can react violently and release hazardous hydrogen gas.]

^{1}H NMR (600 MHz, $CDCl_3$) δ 7.27 – 7.22 (m, 2H), 6.89 – 6.85 (m, 2H), 4.79 – 4.74 (m, 2H), 4.40 (d, *J* = 1.6 Hz, 2H), 3.80 (s, 3H), 3.64 – 3.54 (m, 2H), 3.44 – 3.34 (m, 2H), 2.40 (tt, *J* = 9.1, 5.7 Hz, 1H), 1.72 – 1.58 (m, 7H).

^{13}C{^{1}H} NMR (151 MHz, $CDCl_3$) δ 159.3, 147.2, 130.8, 129.4, 113.9, 112.5, 72.8, 68.3, 61.6, 55.4, 41.2, 36.2, 33.4, 18.0.

$[\alpha]_D^{23}$: +3.76 (*c* 0.50, MeOH)

R_f : 0.43 (1:1 EtOAc/Hex, $KMnO_4$ stain)

HRMS (ESI+): calc. 287.1623 for $C_{16}H_{24}O_3{}^{23}Na$ $[M + Na]^+$, found 287.1621

*Appel to the alkyl bromide (-)-**10***

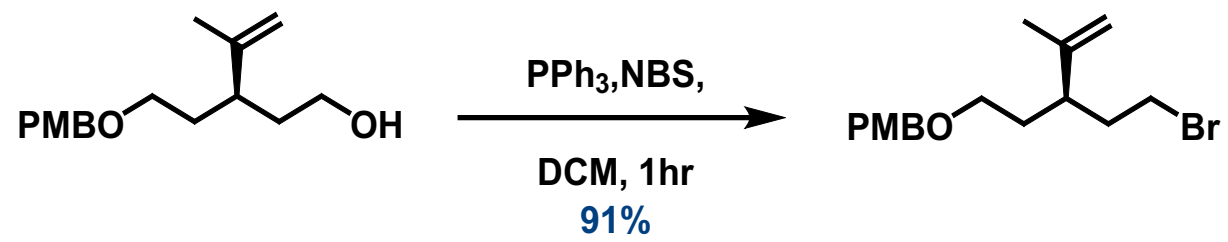

(*R*)-1-(((3-(2-bromoethyl)-4-methylpent-4-en-1-yl)oxy)methyl)-4-methoxybenzene ((-)-**10**): A solution of N-Bromosuccinimide (1.14 g, 1.2 Eq, 6.400 mmol) in dry DCM (2.7 mL) was added dropwise to a solution of alcohol (+)-**22** (1.41 g, 1 Eq, 5.33 mmol) and Triphenylphosphine (1.68 g, 1.2 Eq, 6.40 mmol) in dry DCM (21 mL) at 0 °C. After stirring for 1 hr at room temperature, the reaction mixture was concentrated under reduced pressure. The crude was loaded on silica gel, then purified by flash column chromatography (0 – 25% EtOAc in hexanes) to afford the title compound (1.59 g, 4.87 mmol, yield 91%) as a colorless oil.

^{1}H NMR (600 MHz, $CDCl_3$) δ 7.25 (m, 2H), 6.90 – 6.85 (m, 2H), 4.82 (dt, *J* = 3.0, 1.4 Hz, 1H), 4.78 (dt, *J* = 2.2, 0.8 Hz, 1H), 4.40 (s, 2H), 3.81 (s, 3H), 3.44 – 3.33 (m, 3H), 3.25 (dt, *J* = 9.9, 7.9 Hz, 1H), 2.45 (tt, *J* = 8.6, 6.4 Hz, 1H), 1.91 – 1.86 (m, 2H), 1.69 – 1.62 (m, 2H), 1.61 (dd, *J* = 1.5, 0.8 Hz, 3H).

^{13}C{^{1}H} NMR (151 MHz, $CDCl_3$) δ 159.3, 145.0, 130.8, 129.4, 113.9, 113.6, 72.8, 68.2, 55.4, 42.8, 36.3, 33.1, 31.8, 17.9.

$[\alpha]_D^{23}$: - 19.6 (*c* 0.5, MeOH)

R_f : 0.68 (1:4 EtOAc/Hex, $KMnO_4$ stain)

HRMS (ESI+): calc. 349.0779 for $C_{16}H_{23}O_2{}^{79}Br^{23}Na$ $[M + Na]^+$, found 349.0779

*Appel to alkyl Iodide (-)-**23***

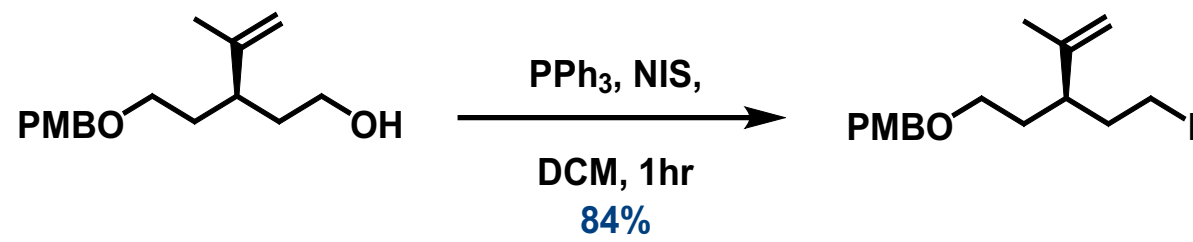

(*R*)-1-(((3-(2-iodoethyl)-4-methylpent-4-en-1-yl)oxy)methyl)-4-methoxybenzene ((-)-**23**): A solution of N-Iodosuccinimide (317 mg, 1.2 Eq, 1.41 mmol) in dry DCM (602 μL) was added dropwise to a solution of alcohol (+)-**22** (310 mg, 1 Eq, 1.17 mmol) and Triphenylphosphine (369 mg, 1.2 Eq, 1.41 mmol) in dry DCM (4.5 mL) at 0 °C. After stirring for 1 hr at room temperature, the solvent was evaporated from the reaction mixture. The crude was purified by silica gel flash column chromatography (0 – 25% EtOAc in hexanes) to afford the title compound (369 mg, 987 μmol, yield 84%) as a colorless oil.

^{1}H NMR (600 MHz, $CDCl_3$) δ 7.30 – 7.21 (m, 2H), 6.90 – 6.86 (m, 2H), 4.82 (dd, *J* = 2.3, 1.4 Hz, 1H), 4.79 (dp, *J* = 2.2, 0.8 Hz, 1H), 4.40 (d, *J* = 0.9 Hz, 2H), 3.81 (s, 3H), 3.44 – 3.33 (m, 2H), 3.20 – 3.12 (m, 1H), 2.99 (dt, *J* = 9.6, 8.1 Hz, 1H), 2.41 – 2.33 (m, 1H), 1.92 – 1.79 (m, 2H), 1.68 – 1.63 (m, 2H), 1.60 (dd, *J* = 1.5, 0.8 Hz, 3H).

^{13}C{^{1}H} NMR (151 MHz, $CDCl_3$) δ 159.3, 144.9, 130.7, 129.4, 113.9, 113.7, 72.8, 68.2, 55.4, 44.9, 37.1, 32.9, 17.8, 4.8.

$[\alpha]_D^{23}$: - 24.6 (*c* 1.0, MeOH)

R_f : 0.70 (1:4 EtOAc/Hex, $KMnO_4$ stain)

HRMS (ESI+): calc. 397.0635 for $C_{16}H_{23}O_2{}^{127}I^{23}Na$ $[M + Na]^+$, found 397.0638

*Synthesis of tertiary alcohol **24***

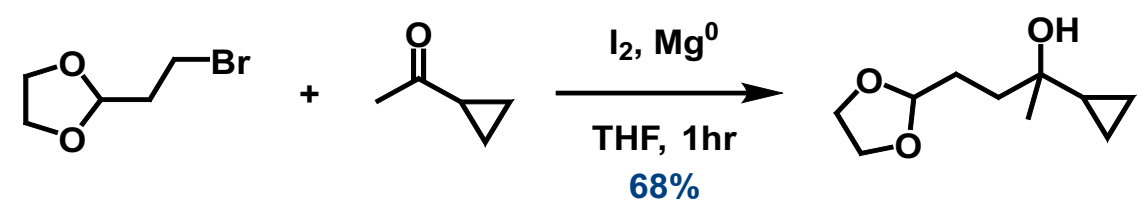

2-cyclopropyl-4-(1,3-dioxolan-2-yl)butan-2-ol (**24**): To an oven-dried reaction vial equipped with a magnetic stir bar was added Magnesium turnings (242 mg, 3 Eq, 9.94 mmol) and catalytic Iodine (8 mg, 0.01 Eq, 33.1 µmol). The vial was cooled to 0°C, and a solution of bromide **13** (390 µL, 1 Eq, 3.31 mmol) in dry THF (6.6 mL) was added dropwise. The mixture was stirred at room temperature for 2 hr. At 0°C, freshly distilled cyclopropyl methyl ketone **15** (348 µL, 1.1 Eq, 3.65 mmol) was added and stirred at room temperature for an additional 1 hr (the mixture turned milky white upon addition of the ketone). The reaction was quenched using water (5 mL), and the mixture was extracted with ethyl acetate (3 x 5 mL). The organic layer was dried using brine, then anhydrous Na_2SO_4, filtered, and concentrated under reduced pressure. The crude product was purified using Silica gel column chromatography (0 – 15% EtOAc in hexanes) to afford the title compound (423 mg, 2.27 mmol, yield 68%) as a colorless oil.

[**Caution!** Elemental iodine (I_2) sublimes at room temperature, releasing vapors that severely irritate the respiratory tract and are corrosive to skin and eyes. If ingested or absorbed, I_2 is toxic and can be fatal.]

^{1}H NMR (400 MHz, $CDCl_3$) δ 4.89 (t, J = 4.6 Hz, 1H), 4.03 – 3.93 (m, 2H), 3.92 – 3.82 (m, 2H), 1.95 – 1.78 (m, 2H), 1.73 – 1.64 (m, 2H), 1.62 (s, 1H), 1.11 (s, 3H), 0.86 (tt, J = 8.4, 5.6 Hz, 1H), 0.44 – 0.31 (m, 3H), 0.31 – 0.23 (m, 1H).

^{13}C{^{1}H} NMR (101 MHz, $CDCl_3$) δ 104.8, 70.4, 65.1, 36.9, 28.4, 26.4, 21.1, 0.8, 0.5.

HRMS (APCI+): Calcd. 187.1329 for $C_{10}H_{19}O_3$ $[M+H]^+$, found 187.1326

*Preparation of alkyl bromide **25***

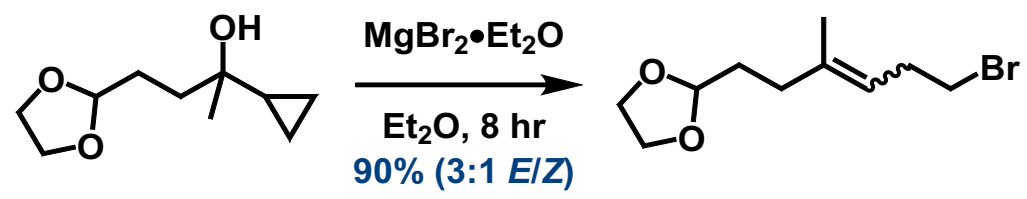

2-(6-bromo-3-methylhex-3-en-1-yl)-1,3-dioxolane (**25**): Prepared according to literature procedure.[2] In a flame-dried two-neck round-bottomed flask, equipped with a condenser and a magnetic stir bar, a mixture of alcohol **24** (1.00 g, 1 Eq, 5.37 mmol) and magnesium bromide-diethyl ether (1.73 g, 1.25 Eq, 6.71 mmol) in Et_2O (21 mL) was stirred at 40 ºC in an oil bath for 8 hr. After completion of the reaction, the mixture was diluted with water (20 mL) and extracted with ethyl acetate (3 x 50 mL). The combined organic layers were dried over anhydrous Na_2SO_4, filtered, and concentrated under reduced pressure. The crude product was purified via flash column chromatography (silica gel, isocratic hexanes) to afford the title compound (1.20 g, 4.83 mmol, yield 90%), as a colorless oil. 3:1 *E*/*Z* determined by ^{1}H NMR of crude material.

Note: a small portion of the first column chromatography fraction was dried under low pressure and used for characterization. This sample was found to be enriched with the major isomer (8:1 *E/Z*). However, a 3:1 *E/Z* mixture of **25** (from this and other batches) was used for subsequent reactions.

^{1}H NMR (400 MHz, $CDCl_3$) δ 5.18 (tp, *J* = 7.1, 1.4 Hz, 1H), 4.86 (t, *J* = 4.7 Hz, 1H), 4.03 – 3.91 (m, 2H), 3.90 – 3.77 (m, 2H), 3.34 (t, *J* = 7.3 Hz, 2H), 2.64 – 2.50 (m, 2H), 2.13 (dd, *J* = 9.9, 6.3 Hz, 2H), 1.82 – 1.69 (m, 2H), 1.67 – 1.62 (m, 3H).

^{13}C{^{1}H} NMR (151 MHz, $CDCl_3$) δ 137.9, 121.2, 104.3, 65.0, 33.9, 32.9, 32.4, 31.8, 16.5.

R_f : 0.54 (1:9 EtOAc/Hex, $KMnO_4$ stain)

HRMS (APCI+): Calcd. 249.0485 for $C_{10}H_{18}O_2{}^{79}Br$ $[M+H]^+$, found 249.0485

*Synthesis of gem-dibromo alkene **11***

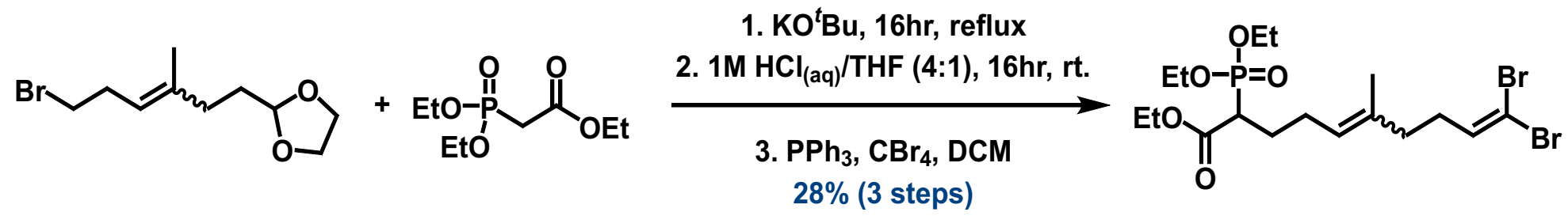

ethyl 10,10-dibromo-2-(diethoxyphosphoryl)-6-methyldeca-5,9-dienoate (11): To a suspension of KOtBu (820 mg, 1.3 Eq, 7.31 mmol) in THF (26 mL) was added phosphonate ester **16** (1.76 g, 1.6 mL, 1.4 Eq, 7.87 mmol). The mixture was stirred for 30 min at room temperature, then cooled to 0°C, and alkyl bromide **25** (1.40 g, 1 Eq, 5.62 mmol) in THF (8.8 mL) was added. The reaction mixture was refluxed in an oil bath overnight and then quenched with sat. $NH_4Cl_{(aq)}$, extracted with EtOAc (3 × 20 mL), washed with brine, and dried over anhydrous Na_2SO_4. The solvent was removed *in vacuo*, and the crude mixture was purified by flash chromatography (silica gel, 0 – 80% EtOAc in hexanes).

To a round-bottomed flask charged with a magnetic stir bar, the dioxolane intermediate was added as a solution in 16 mL of THF, followed by 67 mL of 1 M $HCl_{(aq)}$. The reaction mixture was stirred for 16 hr at room temperature, and then extracted using EtOAc (3 × 100 mL) washed with brine, and dried over anhydrous Na_2SO_4. The solvent was removed in vacuo, and the crude aldehyde **26** was dried under high vacuum and used without further purification.

To a stirred solution of Triphenylphosphine (3.31 g, 3 Eq, 12.6 mmol) in DCM (5.3 mL) at 0 °C under argon, a solution of CBr_4 (2.09 g, 1.5 Eq, 6.31 mmol) in DCM (2.1 mL) was added. The mixture was stirred at room temperature for 30 min before being cooled to 0 °C. A solution of the crude aldehyde **26** (1.47 g, 1 Eq, 4.21 mmol) in DCM (1.0 mL) was added dropwise. The reaction mixture was warmed to room temperature, and stirring continued for a further 30 min. Hexanes were added to the reaction mixture, and the resulting precipitate was filtered. The filtrate was concentrated under reduced pressure. The crude was purified using column chromatography (silica gel, 0 – 80%, EtOAc in hexanes, gradient) to afford **11** (800 mg, 1.59 mmol, yield 28%) as a pale-yellow oil.

^{1}H NMR (400 MHz, $CDCl_3$) δ 6.34 (td, J = 6.9, 4.3 Hz, 1H), 5.19 – 5.04 (m, 1H), 4.32 – 4.07 (m, 6H), 3.01 – 2.87 (m, 1H), 2.26 – 2.12 (m, 2H), 2.11 – 1.93 (m, 5H), 1.96 – 1.80 (m, 1H), 1.71 – 1.65 (m, 2H), 1.58 (d, J = 1.3 Hz, 2H), 1.35 – 1.26 (m, 9H).

^{13}C{^{1}H} NMR (101 MHz, $CDCl_3$) δ 169.3 (d, J = 4.9 Hz), 138.3, 138.1, 135.5, 135.4, 124.8, 123.9, 89.2, 88.9, 62.8 (t, J = 6.4 Hz), 61.5 (d, J = 4.6 Hz), 46.1, 46.0, 44.8, 44.7, 37.5, 31.6, 31.5, 29.8, 27.3 – 26.5 (m), 23.3, 16.5 (dd, J = 6.1, 4.1 Hz), 16.0, 14.3.

$^{31}P\{^{1}H\}$ NMR (162 MHz, $CDCl_3$) δ 22.87.

R_f : 0.68 (4:1 ethyl acetate/hexanes, $KMnO_4$ stain)

HRMS (APCI+): Calcd. 503.0192 for $C_{17}H_{30}O_5{}^{79}Br_2P$ $[M+H]^+$, found 503.0195

*Synthesis of gem-dibromo alkene **29***

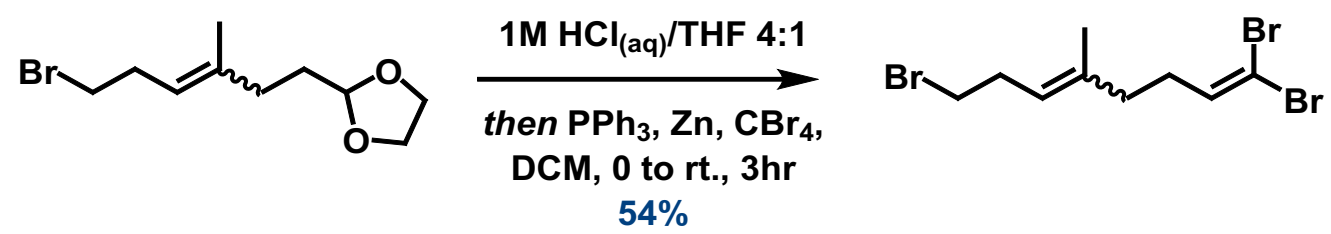

(*E*)-1,1,8-tribromo-5-methylocta-1,5-diene (**29**): To a round-bottomed flask charged with a magnetic stir-bar was added alkyl bromide **25** (610 mg, 1 Eq, 2.45 mmol) as a solution dissolved in 10 mL of THF, followed by 37 mL of 1 M $HCl_{(aq)}$. The reaction mixture was stirred for 16 hr at room temperature and then extracted using EtOAc (3 × 20 mL), washed with brine, and dried over anhydrous Na_2SO_4. The solvent was removed *in vacuo*, and the crude aldehyde (369 mg, 1.80 mmol) was dried under high vacuum and used without further purification.

To a stirred solution of Triphenylphosphine (892 mg, 2 Eq, 3.40 mmol) in DCM (2.1 mL) at 0 °C under argon, was added zinc (222 mg, 2 Eq, 3.40 mmol) followed by a solution of CBr_4 (1.13 g, 2 Eq, 3.40 mmol) in DCM (850 µL). The reaction mixture was stirred at room temperature for 1 hr, then cooled to 0 °C. A solution of the crude aldehyde (349 mg, 1 Eq, 1.70 mmol) in DCM (425 µL) was added dropwise. The reaction mixture was warmed to room temperature, and stirring continued for a further 2 hr. Hexanes were added to the reaction mixture, and the resulting precipitate was collected by filtration. The filtrate was then concentrated under reduced pressure and subjected to column chromatography to afford **29** (480 mg, 1.33 mmol, yield 54%) as a colorless oil.

^{1}H NMR (400 MHz, $CDCl_3$) δ 6.40 – 6.32 (m, 1H), 5.21 – 5.12 (m, 1H), 3.36 (td, J = 7.2, 0.8 Hz, 2H), 2.59 (q, J = 7.1 Hz, 2H), 2.22 (tdd, J = 7.8, 6.5, 1.1 Hz, 2H), 2.12 (t, J = 7.4 Hz, 2H), 1.64 (t, J = 1.2 Hz, 3H).

^{13}C{^{1}H} NMR (101 MHz, $CDCl_3$) δ 138.2, 137.0, 122.5, 89.0, 37.5, 32.9, 31.7, 31.4, 16.3.

R_f : 0.53 (hexanes, $KMNO_4$ stain)

HRMS (APCI+): Calcd. 356.8484 for $C_9H_{12}{}^{79}Br_3$ $[M-H]^+$, found 356.8483

*Synthesis of alkylbromide **S4***

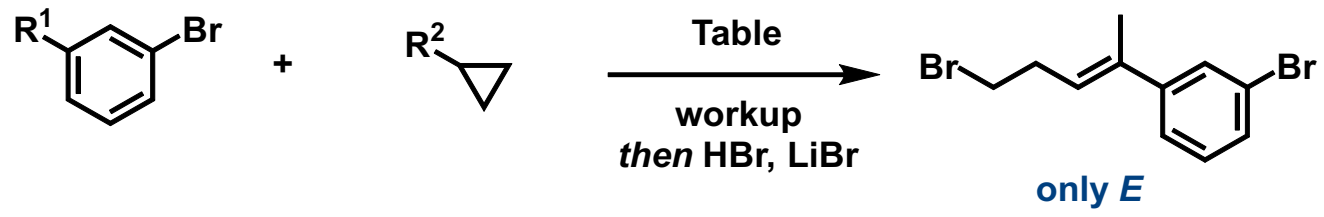

Entry	R_1 | R_2	Grignard formation	2-step yield (%)
1	-Br | -$COCH_3$	Mg^0, THF, 1 hr	54
2	-$COCH_3$ | -Br	Mg^0, THF, 1 hr	49
3	-Br | -$COCH_3$	iPrMgCl·LiCl, THF, 1 hr	70

(*E*)-1-bromo-3-(5-bromopent-2-en-2-yl)benzene (**S4**): To an oven-dried round-bottomed flask equipped with a magnetic stir bar was added a solution of freshly distilled 1,3-dibromobenzene **14** (673 mg, 345 µL, 1.2 Eq, 2.85 mmol) in dry THF (5 mL), and the round-bottomed flask was cooled to 0°C. The turbo Grignard, iPrMgCl·LiCl complex (380 mg, 2.0 mL, 1.3 M, 1.1 Eq, 2.62 mmol), was added dropwise, and the mixture was stirred at 40 °C for 3 hr. After cooling to 0 °C, freshly distilled cyclopropyl methyl ketone **15** (0.20 g, 227 µL, 1 Eq, 2.38 mmol) was added, and the mixture was stirred at room temperature for another 2 hr. The reaction was quenched using water (5 mL), and the mixture was extracted with ethyl acetate (3 x 5 mL). The organic layer was dried using brine, then anhydrous Na_2SO_4, filtered, and concentrated under reduced pressure. To the crude mixture, hydrogen bromide (601 mg, 408 µL, 48% Wt, 1.5 Eq, 3.56 mmol) and Lithium bromide (206 mg, 1 Eq, 2.38 mmol) were stirred at 0 ºC for 2 hr. After completion of the reaction, the reaction mixture was diluted with water (5 mL) and extracted with ethyl acetate (3 x 5 mL). The combined organic layers were dried over anhydrous Na_2SO_4, filtered, and concentrated under reduced pressure. The crude product was purified via flash column chromatography (silica gel, isocratic hexanes) to afford the title compound (507 mg, 1.67 mmol, 70%) as a colorless oil.

^{1}H NMR (400 MHz, $CDCl_3$) δ 7.52 (t, *J* = 1.9 Hz, 1H), 7.37 (ddd, *J* = 7.8, 2.0, 1.1 Hz, 1H), 7.30 (ddd, *J* = 7.9, 1.8, 1.1 Hz, 1H), 7.18 (t, *J* = 7.9 Hz, 1H), 5.76 (tq, *J* = 7.1, 1.4 Hz, 1H), 3.46 (t, *J* = 7.1 Hz, 2H), 2.84 – 2.74 (m, 2H), 2.03 (dt, *J* = 1.5, 0.9 Hz, 3H).

^{13}C{^{1}H} NMR (151 MHz, $CDCl_3$) δ 145.6, 136.7, 130.1, 129.9, 129.0, 125.9, 124.5, 122.6, 32.3, 32.3, 16.2.

R_f : 0.29 (hexanes, UV and $KMNO_4$ stain)

HRMS (APCI+): Calcd. 302.9379 for $C_{11}H_{13}{}^{79}Br_2$ $[M+H]^+$, found 302.9385

*Synthesis of styrene **12***

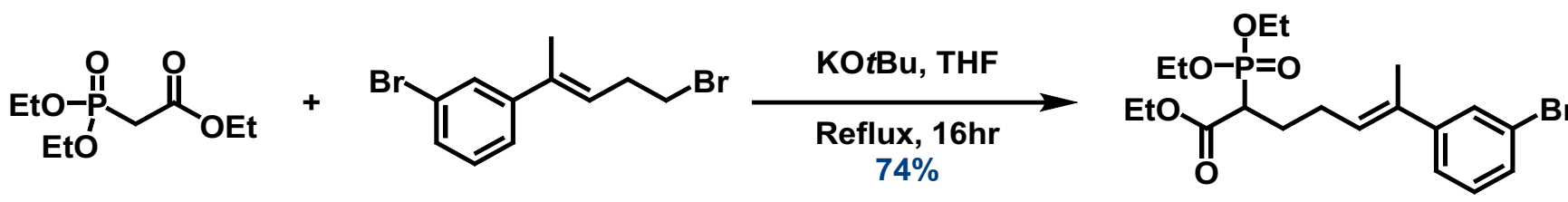

ethyl (*E*)-6-(3-bromophenyl)-2-(diethoxyphosphoryl)hept-5-enoate (**12**): To a suspension of KOtBu (3.87 g, 1.2 Eq, 35 mmol) in THF (135 mL) was added phosphonate-ester **16** (7.73 g, 7 mL, 1.2 Eq, 35 mmol). The mixture was stirred for 30 min at room temperature, then cooled to 0°C, and **S4** (8.74 g, 1 Eq, 28.7 mmol) in THF (45 mL) was added. The reaction mixture was refluxed in an oil bath overnight and then quenched with sat. $NH_4Cl_{(aq)}$, extracted with EtOAc (3 × 100 mL), washed with brine, and dried over anhydrous Na_2SO_4. The solvent was removed *in vacuo*, and the crude was purified by flash chromatography (silica gel, 20 – 80% EtOAc in hexanes) to afford the title compound (9.57 g, 21.4 mmol, yield 74%) as a colorless oil.

^{1}H NMR (600 MHz, $CDCl_3$) δ 7.48 (dd, *J* = 2.1, 1.3 Hz, 1H), 7.34 (ddq, *J* = 7.9, 2.2, 1.1 Hz, 1H), 7.29 – 7.24 (m, 1H), 7.16 (tt, *J* = 7.9, 1.0 Hz, 1H), 5.70 (tt, *J* = 7.4, 1.6 Hz, 1H), 4.28 – 4.08 (m, 6H), 2.97 (dddt, *J* = 22.8, 10.8, 3.5, 0.8 Hz, 1H), 2.33 – 2.11 (m, 3H), 2.05 – 1.95 (m, 4H), 1.36 – 1.30 (m, 6H), 1.30 – 1.25 (m, 3H).

^{13}C{^{1}H} NMR (151 MHz, $CDCl_3$) δ 169.3 (d, *J* = 5.0 Hz), 145.9, 135.4, 129.82, 129.78, 128.9, 127.6, 124.4, 122.6, 62.9 (dd, *J* = 11.3, 6.5 Hz), 61.6, 45.4 (d, *J* = 130.7 Hz), 27.6 (d, *J* = 15.4 Hz), 26.8 (d, *J* = 4.7 Hz), 16.5 (t, *J* = 6.1 Hz), 15.9, 14.3.

^{31}P{^{1}H} NMR (243 MHz, $CDCl_3$) δ 22.60.

R_f : 0.68 (4:1 ethyl acetate/hexanes, UV and $KMNO_4$ stain)

HRMS (APCI+): Calcd. for $C_{19}H_{29}BrO_5P$ $[M+H]^+$ 447.0931, found 447.0924

Cross-electrophile coupling

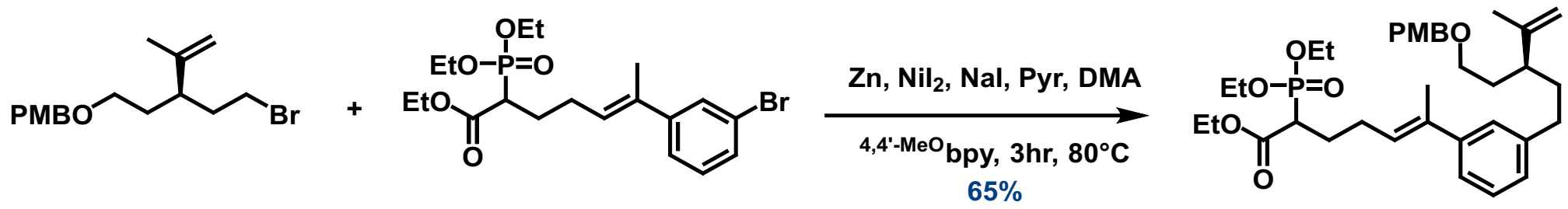

ethyl (E)-2-(diethoxyphosphoryl)-6-(3-((S)-3-(2-((4-methoxybenzyl)oxy)ethyl)-4-methylpent-4-en-1-yl)phenyl)hept-5-enoate ((+)-**36**): To an oven-dried 100 mL round-bottomed flask, equipped with a magnetic stir bar was added Sodium iodide (252 mg, 0.25 Eq, 1.68 mmol) and zinc (1.32 g, 3 Eq, 20.2 mmol) and the reagents were flame-dried under vacuum then allowed to cool to room temperature. A solution of alkyl bromide (-)-**10** (2.20 g, 1 Eq, 6.72 mmol) and aryl bromide **12** (3.61 g, 1.2 Eq, 8.07 mmol), pre-mixed in dry DMA (60 mL), was added, followed by 1 drop (~ 50 µL) of Pyridine. To the mixture, a solution of Nickel(II)iodide (231 mg, 0.11 Eq, 740 µmol) and 4-Methoxy-2-(4-methoxypyridin-2-yl)pyridine (145 mg, 0.10 Eq, 672 µmol) pre-ligated (stirred together at 40°C on a heating block for 1 hr then allowed to cool) in 1 mL of DMA was added (final DMA volume 61 mL, 110 mM), and the reaction mixture was stirred in an oil bath at 80 °C for 3 hr. The reaction was quenched using sat. NH_4Cl, diluted with ethyl acetate (3 × 200 mL), and filtered through a pad of celite, then washed with brine, and the combined organics were dried using anhydrous Na_2SO_4. The solvent was removed under reduced pressure, and the crude was purified by column chromatography on silica gel (20 – 65% EtOAc in hexanes, gradient) to afford adduct (+)-**36** (2.70 g, 4.38 mmol, yield 65%).

[**Caution!** Sodium iodide is an irritant that can cause skin and serious eye irritation, and may affect the thyroid or an unborn child upon prolonged exposure. Pyridine is a highly flammable liquid that causes skin and eye irritation and is harmful if swallowed, inhaled, or absorbed through the skin.]

^{1}H NMR (600 MHz, $CDCl_3$) δ 7.26 – 7.12 (m, 5H), 7.02 (dt, *J* = 7.4, 1.5 Hz, 1H), 6.89 – 6.85 (m, 2H), 5.68 (tt, *J* = 7.3, 1.4 Hz, 1H), 4.82 (dp, *J* = 3.4, 1.7 Hz, 1H), 4.74 (dt, *J* = 2.4, 0.7 Hz, 1H), 4.39 (d, *J* = 1.5 Hz, 2H), 4.26 – 4.10 (m, 6H), 3.80 (s, 3H), 3.44 – 3.32 (m, 2H), 3.06 – 2.95 (m, 1H), 2.54 (dt, *J* = 13.7, 8.0 Hz, 1H), 2.46 (dt, *J* = 13.8, 8.0 Hz, 1H), 2.34 – 2.11 (m, 4H), 2.04 – 1.94 (m, 4H), 1.75 – 1.58 (m, 6H), 1.35 – 1.31 (m, 6H), 1.28 (t, *J* = 7.1 Hz, 3H).

^{13}C{^{1}H} NMR (151 MHz, $CDCl_3$) δ 169.3 (d, *J* = 4.9 Hz), 159.2, 146.6, 143.7, 142.7, 136.7, 130.8, 129.4, 128.3 (d, *J* = 10.8 Hz), 126.9, 126.0, 125.9, 125.7, 123.18, 113.8, 112.6, 72.8, 68.5, 62.8 (dd, *J* = 11.6, 6.6 Hz), 61.5, 55.4, 45.4 (d, *J* = 130.6 Hz), 44.0, 35.5, 33.7 (d, *J* = 89.1 Hz), 27.6 (d, *J* = 15.5 Hz), 26.9 (d, *J* = 4.4 Hz), 17.9, 16.5 (t, *J* = 6.0 Hz), 16.0, 14.3.

^{31}P NMR (243 MHz, $CDCl_3$) δ 23.07 – 22.59 (m).

$[\alpha]_D^{23}$: +2.59 (*c* 1.0, $CHCl_3$)

R_f : 0.56 (4:1 EtOAc/Hex, UV and $KMnO_4$ stain)

HRMS (APCI+): calc. 615.3445 for $C_{35}H_{52}O_7P$ $[M + H]^+$, found 615.3443

*Synthesis of **38** via Negishi coupling*

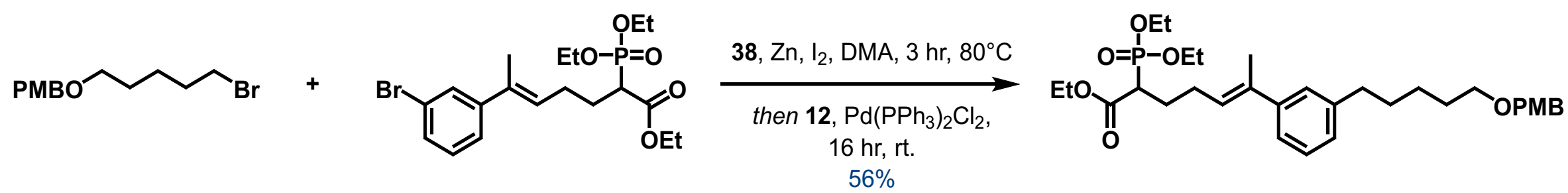

ethyl (E)-2-(diethoxyphosphoryl)-6-(3-(5-((4-methoxybenzyl)oxy)pentyl)phenyl)hept-5-enoate (**38**): Prepared according to literature procedure.[3] To a flame-dried round-bottomed flask equipped with a stir bar was added dry DMA (5 mL), Iodine (66 mg, 0.05 Eq, 261 μmol), and zinc dust (512 mg, 1.5 Eq, 7.83 mmol). The mixture was stirred at 23 °C until the red color of I_2 disappeared (ca. 2 min). Alkyl bromide **37** (1.50 g, 1 Eq, 5.22 mmol) [**37** was prepared according to a literature procedure[4]] was added, and the mixture was stirred at 80 °C in an oil bath for 3 hr. The mixture was cooled to room temperature, and arylbromide **12** (1.87 g, 0.8 Eq, 4.178 mmol) and $Pd(PPh_3)_2Cl_2$ (550 mg, 0.15 Eq, 783.4 μmol) were added successively at 23 °C, then stirred at the temperature overnight. The reaction was quenched with NH_4Cl, and the mixture was extracted with ethyl acetate (4 × 20 mL). The combined organic phases were washed with brine, dried over Na_2SO_4, filtered, and evaporated. The crude was purified using flash chromatography on silica gel (20 – 60% EtOAc in hexanes, gradient) to give the title compound (1.34 g, 2.33 mmol, yield 56%) as a clear, colorless oil.

[**Caution!** Elemental iodine (I_2) sublimes at room temperature, releasing vapors that severely irritate the respiratory tract and are corrosive to skin and eyes. If ingested or absorbed, I_2 is toxic and can be fatal.]

^{1}H NMR (600 MHz, $CDCl_3$) δ 7.28 – 7.22 (m, 2H), 7.18 – 7.14 (m, 2H), 7.03 (dt, *J* = 7.4, 1.5 Hz, 1H), 6.89 – 6.85 (m, 2H), 5.73 – 5.66 (m, 1H), 4.42 (s, 2H), 4.26 – 4.09 (m, 6H), 3.80 (s, 3H), 3.44 (t, *J* = 6.7 Hz, 2H), 3.00 (ddd, *J* = 22.8, 10.9, 3.5 Hz, 1H), 2.62 – 2.57 (m, 2H), 2.33 – 2.20 (m, 2H), 2.16 (ddqd, *J* = 14.0, 8.2, 5.6, 2.9 Hz, 1H), 2.08 – 1.86 (m, 4H), 1.67 – 1.59 (m, 4H), 1.33 (tdd, *J* = 7.1, 3.8, 0.5 Hz, 8H), 1.28 (t, *J* = 7.1 Hz, 3H).

^{13}C{^{1}H} NMR (151 MHz, $CDCl_3$) δ 169.2 (d, *J* = 4.7 Hz), 159.1, 143.6, 142.6, 136.6, 130.8, 129.2, 128.1, 126.9, 125.9, 125.8, 123.1, 113.8, 72.5, 70.1, 62.7 (dd, *J* = 11.5, 6.5 Hz), 61.4, 55.3, 45.3 (dd, *J* = 130.5, 2.7 Hz), 36.1, 31.5, 29.7, 27.5 (d, *J* = 15.5 Hz), 26.8 (d, *J* = 4.7 Hz), 26.0, 16.4 (t, *J* = 6.0 Hz), 15.9, 14.2.

^{31}P{^{1}H} NMR (243 MHz, $CDCl_3$) δ 22.80.

R_f: 0.40 (1:4 Hex/EtOAc, $KMnO_4$ stain)

HRMS (APCI+): calc. 575.3132 for $C_{32}H_{48}O_7P$ $[M + H]^+$, found 575.3129

BCl_3-mediated PMB Deprotection

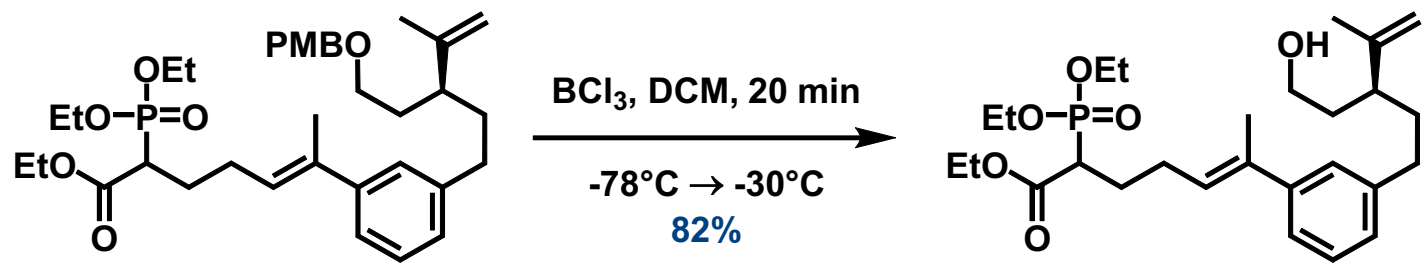

ethyl (E)-2-(diethoxyphosphoryl)-6-(3-((S)-3-(2-hydroxyethyl)-4-methylpent-4-en-1-yl)phenyl)hept-5-enoate (**S5**): To a solution of (+)-**36** (290 mg, 1 Eq, 472 µmol) in DCM (40 mL) at -78°C was added boron trichloride (710 µL, 1.0 molar, 1.5 Eq, 710 µmol) in DCM slowly then stirred at the temperature for 2 min. The solution was warmed to -30°C and stirred at the temperature for 15 min, then quenched using cold sat. $NaHCO_{3(aq)}$. The organic layer was separated, and the aqueous layer was extracted with DCM (2 × 10 mL). The combined organic phases were washed with brine, dried using Na_2SO_4, and concentrated under reduced pressure, and the residue was purified by column chromatography (silica gel, 20 – 75% EtOAc in hexanes, gradient) to afford the corresponding alcohol (192 mg, 389 µmol, yield 82%) as a colorless viscous oil.

[**Caution!** Boron trichloride (BCl_3) is an extremely corrosive and toxic gas that reacts violently with water to release dangerous hydrogen chloride fumes. Breathing the gas irritates the respiratory tract and can cause lung damage.]

^{1}H NMR (800 MHz, $CDCl_3$) δ 7.20 (t, *J* = 7.5 Hz, 1H), 7.17 – 7.14 (m, 2H), 7.03 (dt, *J* = 7.6, 1.4 Hz, 1H), 5.68 (tt, *J* = 7.2, 1.3 Hz, 1H), 4.84 (ddd, *J* = 2.3, 1.5, 0.8 Hz, 1H), 4.79 (d, *J* = 2.3 Hz, 1H), 4.22 (dq, *J* = 10.7, 7.1 Hz, 1H), 4.20 – 4.11 (m, 5H), 3.64 – 3.55 (m, 2H), 3.00 (ddd, *J* = 22.8, 11.0, 3.5 Hz, 1H), 2.58 – 2.52 (m, 1H), 2.50 – 2.43 (m, 1H), 2.33 – 2.12 (m, 4H), 2.03 – 1.85 (m, 6H), 1.69 – 1.62 (m, 7H), 1.32 (td, *J* = 7.1, 4.5 Hz, 6H), 1.27 (t, *J* = 7.1 Hz, 3H).

^{13}C{^{1}H} NMR (201 MHz, $CDCl_3$) δ 169.3 (d, *J* = 5.1 Hz), 147.2, 143.7 (d, *J* = 2.2 Hz), 142.5, 136.7, 128.2, 126.9, 126.1, 125.9, 123.2, 112.7, 62.9 (dd, *J* = 12.9, 6.5 Hz), 61.5 (d, *J* = 8.9 Hz), 45.7, 45.0, 44.1 (d, *J* = 2.9 Hz), 36.2, 35.4, 33.8, 27.5 (d, *J* = 15.3 Hz), 26.9 (d, *J* = 4.5 Hz), 17.9, 16.5 (dd, *J* = 8.3, 6.8 Hz), 16.0, 14.3.

^{31}P NMR (162 MHz, $CDCl_3$) δ 22.84.

R_f : 0.25 (4:1 EtOAc/Hex, UV and $KMnO_4$ stain)

HRMS (APCI+): calc. 495.2870 for $C_{27}H_{44}O_6P$ $[M + H]^+$, found 495.2873

*Parikh-Doering oxidation to access (-)-**39***

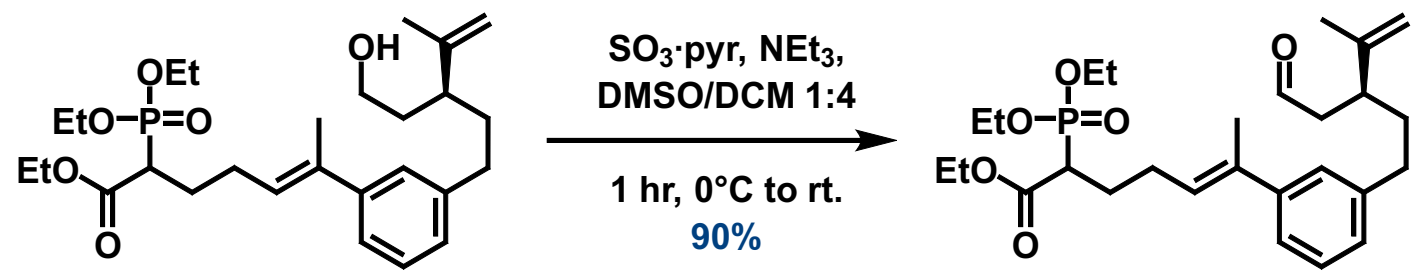

ethyl (E)-2-(diethoxyphosphoryl)-6-(3-((S)-4-methyl-3-(2-oxoethyl)pent-4-en-1-yl)phenyl)hept-5-enoate ((-)-**39**): A solution of **S5** (118 mg, 1 Eq, 239 µmol) and Triethylamine (133 µL, 4 Eq, 958 µmol) in 4:1 DCM/DMSO (2.4 mL) at 0 °C was treated with SO_3·pyr (95 mg, 2.5 Eq, 599 µmol) and stirred for 1 hr at rt. The reaction was diluted with EtOAc (5 mL), washed sequentially with water (3 × 5 mL), saturated $NaHCO_{3(aq)}$, and brine. The organic layer was dried using anhydrous Na_2SO_4 and concentrated under reduced pressure. The crude was purified via flash column chromatography (silica gel, 20 – 75% EtOAc in hexanes, gradient) to afford aldehyde (-)-**39** (107 mg, 216 µmol, yield 90%) as a viscous colorless oil.

[**Caution!** Sulfur trioxide pyridine complex (SO_3·pyr) is a highly reactive, moisture-sensitive, corrosive, and toxic chemical, capable of causing severe chemical burns, irreversible eye damage, and respiratory irritation.]

^{1}H NMR (800 MHz, $CDCl_3$) δ 9.66 (dd, *J* = 2.8, 2.1 Hz, 1H), 7.22 (t, *J* = 7.6 Hz, 1H), 7.18 (dt, *J* = 7.8, 1.5 Hz, 1H), 7.14 (t, *J* = 1.9 Hz, 1H), 7.03 (dt, *J* = 7.4, 1.6 Hz, 1H), 5.71 – 5.66 (m, 1H), 4.89 (p, *J* = 1.6 Hz, 1H), 4.84 (dt, *J* = 1.7, 0.9 Hz, 1H), 4.26 – 4.12 (m, 6H), 3.00 (ddd, *J* = 22.8, 11.0, 3.5 Hz, 1H), 2.73 (tt, *J* = 8.7, 5.9 Hz, 1H), 2.59 (ddd, *J* = 13.8, 10.0, 5.6 Hz, 1H), 2.54 – 2.41 (m, 3H), 2.29 (ddt, *J* = 14.0, 8.3, 4.3 Hz, 1H), 2.23 (dq, *J* = 14.7, 7.6 Hz, 1H), 2.20 – 2.12 (m, 1H), 2.02 – 1.96 (m, 4H), 1.78 – 1.69 (m, 3H), 1.33 (td, *J* = 7.1, 4.7 Hz, 6H), 1.28 (t, *J* = 7.1 Hz, 3H).

^{13}C{^{1}H} NMR (201 MHz, $CDCl_3$) δ 202.3, 169.3 (d, *J* = 5.1 Hz), 145.6, 143.9, 141.9, 136.6, 128.4, 126.9, 126.2, 125.9, 123.5, 113.2, 62.9 (dd, *J* = 13.3, 6.6 Hz), 61.5, 47.6, 45.8, 45.1, 41.4, 35.1, 33.6, 27.6 (d, *J* = 15.5 Hz), 26.9 (d, *J* = 4.5 Hz), 18.8, 16.6 – 16.5 (m), 16.0, 14.3.

^{31}P{^{1}H} NMR (162 MHz, $CDCl_3$) δ 22.78.

$[\alpha]_D^{24}$: -1.34 (*c* 2.0, $CHCl_3$)

R_f : 0.43 (4:1 EtOAc/Hex, UV and $KMnO_4$ stain)

HRMS (APCI+): calc. 493.2714 for $C_{27}H_{42}O_6P$ $[M + H]^+$, found 493.2718

LiOH promoted HWE macrocyclization

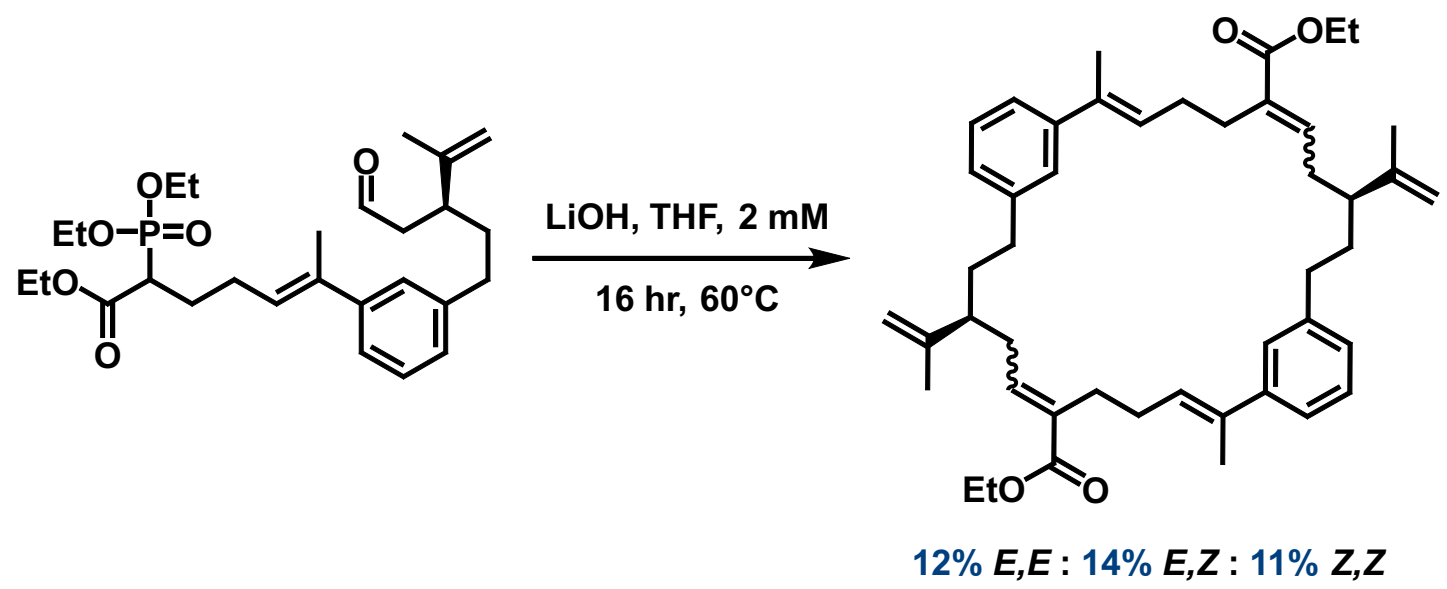

To a suspension of LiOH (778 mg, 100 Eq, 32.5 mmol) in THF (162 mL) at 60 °C in an oil bath, was added a solution of (-)-**39** (160 mg, 1 Eq, 325 µmol) dropwise using a motorized syringe-pump over 4 hr with vigorous stirring. After an additional 16 hr, the reaction was quenched with 6 M HCl(aq), concentrated, and extracted with EtOAc (3 × 10 mL). The combined organics were washed with brine (30 mL), dried over anhydrous Na_2SO_4, and concentrated *in vacuo*. The crude was purified via preparative TLC (5% EtOAc in Hex), and the dimer products were obtained as colorless oils.

E,E-**40** (13 mg, 19.8 µmol, yield 12%)

^{1}H NMR (400 MHz, $CDCl_3$) δ 7.23 – 7.12 (m, 7H), 6.98 (dt, *J* = 7.2, 1.7 Hz, 2H), 6.74 (dd, *J* = 8.1, 6.2 Hz, 2H), 5.80 (td, *J* = 7.5, 1.5 Hz, 2H), 4.84 (t, *J* = 1.8 Hz, 2H), 4.75 – 4.70 (m, 2H), 4.21 (q, *J* = 7.1 Hz, 4H), 2.53 – 2.27 (m, 12H), 2.28 – 2.08 (m, 4H), 2.00 (d, *J* = 1.3 Hz, 4H), 1.72 – 1.61 (m, 11H), 1.31 (t, *J* = 7.1 Hz, 6H).

^{13}C{^{1}H} NMR (151 MHz, $CDCl_3$) δ 167.95, 145.93, 143.96, 142.37, 141.55, 135.67, 132.09, 128.28, 127.31, 127.03, 125.24, 123.25, 112.95, 60.51, 46.89, 35.80, 33.77, 32.80, 28.49, 27.17, 18.57, 15.86, 14.46.

R_f : 0.41 (1:9 EtOAc/Hex, UV and $KMnO_4$ stain), 0.64 (1:4 EtOAc/Hex)

$[\alpha]_D^{23}$: +2.15 (*c* 0.35, $CHCl_3$)

HRMS (APCI+): calc. 677.4564 for $C_{46}H_{61}O_4$ $[M + H]^+$, found 677.4568

*Synthesis of Ring Closing Metathesis (RCM) precursor (+)-**42***

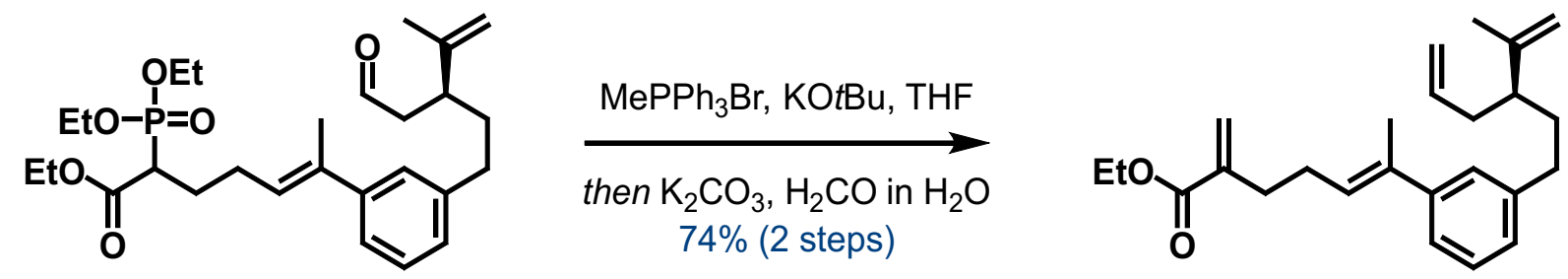

ethyl (*R,E*)-2-methylene-6-(3-(3-(prop-1-en-2-yl)hex-5-en-1-yl)phenyl)hept-5-enoate ((+)-**42**): To a suspension of $MePPh_3Br$ (143 mg, 3.3 Eq, 400 µmol) in dry THF (400 µL) was added KOtBu (42.2 mg, 3.1 Eq, 376 µmol) at 0 °C. After 30 min, aldehyde (-)-**39** (59.7 mg, 1 Eq, 121 µmol) in THF (200 µL) was added and allowed to stir at 0 °C for 1 hr. To the same pot, 1.0 mL of 30% formaldehyde in water and $K_2CO_{3(s)}$ (50 mg, 3 Eq, 363 µmol) were added, then stirred at room temperature overnight. The mixture was extracted with EtOAc (3 × 5 mL). The organic layer was washed with brine (10 mL), dried over Na_2SO_4, and the solvent was removed under reduced pressure. The crude was purified using flash chromatography (silica gel, gradient 0 – 20% EtOAc in hexanes) to afford the title compound (33 mg, 90.0 µmol, yield 74%) as a colorless oil.

^{1}H NMR (800 MHz, $CDCl_3$) δ 7.21 (t, *J* = 7.5 Hz, 1H), 7.17 (dt, *J* = 7.7, 1.5 Hz, 1H), 7.16 (t, *J* = 1.9 Hz, 1H), 7.03 (dt, *J* = 7.5, 1.5 Hz, 1H), 6.18 (d, *J* = 1.5 Hz, 1H), 5.77 – 5.68 (m, 2H), 5.57 (q, *J* = 1.4 Hz, 1H), 5.02 – 4.97 (m, 1H), 4.96 (ddt, *J* = 10.2, 2.2, 1.1 Hz, 1H), 4.83 (dq, *J* = 2.8, 1.5 Hz, 1H), 4.74 (dt, *J* = 2.4, 0.8 Hz, 1H), 4.22 (q, *J* = 7.1 Hz, 2H), 2.57 (ddd, *J* = 13.7, 10.5, 5.2 Hz, 1H), 2.50 – 2.44 (m, 3H), 2.39 (dtt, *J* = 9.1, 7.3, 0.9 Hz, 2H), 2.20 – 2.12 (m, 3H), 2.02 (q, *J* = 1.0 Hz, 3H), 1.71 (dddd, *J* = 13.8, 10.9, 6.5, 4.5 Hz, 1H), 1.67 (dd, *J* = 1.5, 0.8 Hz, 3H), 1.66 – 1.61 (m, 1H), 1.31 (t, *J* = 7.1 Hz, 3H).

^{13}C{^{1}H} NMR (201 MHz, $CDCl_3$) δ 167.4, 147.0, 144.0, 142.7, 140.5, 137.4, 135.8, 128.2, 127.1, 126.8, 125.9, 125.1, 123.2, 115.5, 112.1, 60.7, 47.0, 38.3, 34.7, 33.9, 32.0, 27.9, 18.6, 16.1, 14.4.

$[\alpha]_D^{22}$: + 4.32 (*c* 0.5, DCM)

R_f : 0.79 (1:4 EtOAc/Hex, UV and $KMnO_4$ stain)

HRMS (APCI+): calc. 367.2632 for $C_{25}H_{35}O_2$ $[M + H]^+$, found 367.2634

*Epoxidation of E,E-**40***

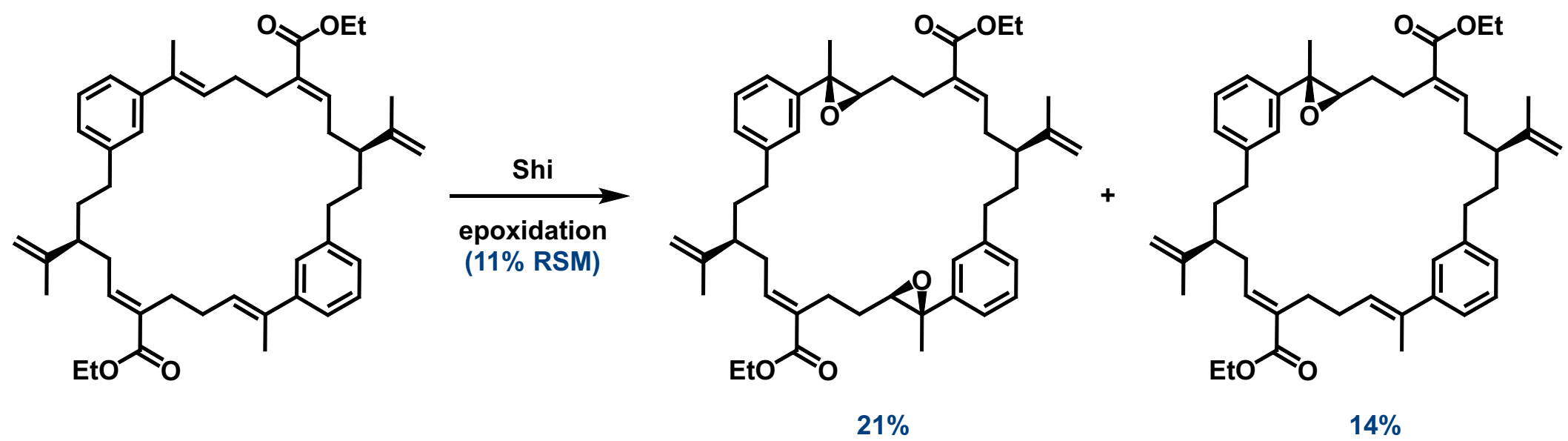

Prepared according to a reported literature procedure.[5] To an oven-dried 15 mL reaction tube equipped with a magnetic stir-bar was added diester *E,E*-**40** (25 mg, 1 Eq, 36.9 μmol) in DME/MeCN (555 μL, 2:1 v/v) followed by buffer (0.05 M solution of $Na_2B_4O_7 \cdot 10H_2O$ in 4 × 10^{-4} M $Na_2EDTA_{(aq)}$, 369 μL), TBAHS (0.5 mg, 0.04 Eq, 1.48 μmol), and D-Epoxone (10.0 mg, 1.05 Eq, 38.8 μmol) were added with stirring. The mixture was cooled to 0 °C via an ice bath. A solution of Oxone® (31 mg, 1.38 Eq, 51 μmol) in 0.4 mM $Na_2EDTA_{(aq)}$ (240 μL) and a solution of $K_2CO_{3(aq)}$ (30 mg, 240 μL, 0.89 M) in water were added dropwise separately & simultaneously over a 2 hr period via a syringe pump. The reaction mixture was quenched by the addition of EtOAc (1 mL) and water (1 mL). The mixture was extracted with EtOAc (3 × 2 mL), washed with brine, dried over anhydrous Na_2SO_4, and purified by preparative TLC to afford the epoxides.

(-)-**43 | Double Epoxidation** (5.4 mg, 7.57 μmol, yield 21%), viscous colorless oil:

^{1}H NMR (600 MHz, $CDCl_3$) δ 7.22 (t, *J* = 7.6 Hz, 1H), 7.17 – 7.13 (m, 1H), 7.05 (d, *J* = 1.8 Hz, 1H), 7.04 – 7.01 (m, 1H), 6.75 (t, *J* = 7.0 Hz, 1H), 4.83 (dq, *J* = 2.9, 1.5 Hz, 1H), 4.73 – 4.71 (m, 1H), 4.19 (qd, *J* = 7.1, 2.0 Hz, 2H), 2.74 (dd, *J* = 7.1, 5.9 Hz, 1H), 2.56 (dq, *J* = 14.0, 7.1 Hz, 1H), 2.52 – 2.39 (m, 2H), 2.35 (ddd, *J* = 13.8, 11.4, 5.5 Hz, 1H), 2.16 – 2.11 (m, 2H), 2.06 (tt, *J* = 9.0, 5.5 Hz, 1H), 1.97 – 1.88 (m, 1H), 1.83 (ddt, *J* = 13.8, 9.2, 6.9 Hz, 1H), 1.60 (s, 3H), 1.59 (dd, *J* = 1.5, 0.8 Hz, 3H), 1.51 – 1.43 (m, 1H), 1.29 (t, *J* = 7.1 Hz, 3H).

^{13}C{^{1}H} NMR (151 MHz, $CDCl_3$) δ 167.6, 145.8, 143.2, 142.9, 142.3, 131.3, 128.4, 127.5, 124.2, 122.9, 113.2, 67.0, 60.8, 60.6, 47.1, 35.9, 34.0, 32.5, 28.2, 23.9, 18.3, 17.4, 14.4.

$[\alpha]_D^{23}$: - 22.2 (*c* 0.36, $CHCl_3$)

R_f : 0.45 (1:4 EtOAc/Hex, $KMnO_4$ stain)

HRMS (APCI+): calc. 709.4463 for $C_{46}H_{61}O_6$ $[M + H]^+$, found 709.4466

E,E-**40**

X-ray Crystallography Center

R_1=5.85%

Crystal Data and Experimental

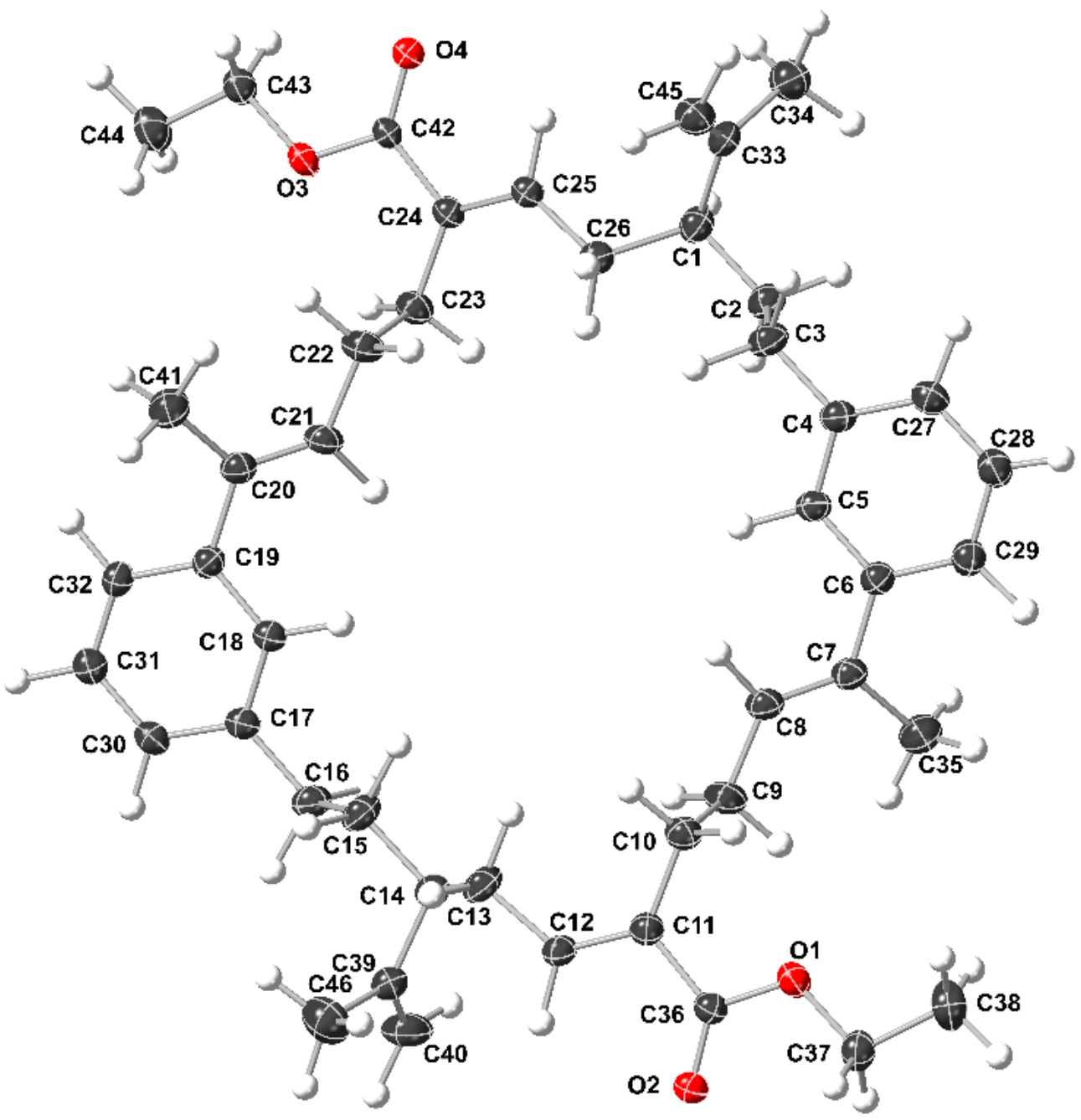

Experimental.[6–8] Single colorless prism-shaped crystals of *E,E*-**40** were recrystallised from methanol by slow evaporation. A suitable crystal with dimensions 0.19 × 0.10 × 0.03 mm was selected and mounted on a nylon loop with NVH oil on a XtaLAB Synergy, Dualflex, HyPix diffractometer. The crystal was kept at a constant T = 100.0(1) K during data collection. The structure was solved with the ShelXT (Sheldrick, 2015) and Olex2 1.5-dev (Dolomanov et al., 2009). The model was refined with olex2.refine 1.5-dev (Bourhis et al., 2015) using full matrix least squares minimisation on $|F|^2$.

Crystal Data. $C_{46}H_{60}O_4$, M_r = 676.987, monoclinic, $P2_1$ (No. 4), a = 15.9751(5) Å, b = 6.71509(19) Å, c = 18.8950(5) Å, β = 100.108(3)°, α = γ = 90°, V = 1995.49(10) Å³, T = 100.0(1) K, Z = 2, Z' = 1, μ(Cu K_α) = 0.541, 34758 reflections measured, 8163 unique (R_{int} = 0.0748) which were used in all calculations. The final wR_2 was 0.1238 (all data) and R_1 was 0.0585 (I≥2σ(I)).

**Compound**	*E,E*-**40**
Formula	$C_{46}H_{60}O_4$
$D_{calc.}$/ g cm^{-3}	1.127
μ/mm^{-1}	0.541
Formula Weight	676.987
Colour	colourless
Shape	prism-shaped
Size/mm	0.19×0.10×0.03
T/K	100.00(10)
Crystal System	monoclinic
Flack Parameter	0.09(16)
Hooft Parameter	0.17(14)
Space Group	$P2_1$
a/Å	15.9751(5)
b/Å	6.71509(19)
c/Å	18.8950(5)
α/°	90
β/°	100.108(3)
γ/°	90
V/Å³	1995.49(10)
Z	2
Z'	1
Wavelength/Å	1.54184
Radiation type	Cu K_α
Θ_{min}/°	2.81
Θ_{max}/°	77.30
Index range h	-20 ≤ h ≤ 20
Index range k	-8 ≤ k ≤ 8
Index range l	-23 ≤ l ≤ 22
Measured Refl's.	34758
Indep't Refl's	8163
Refl's I≥2σ(I)	6772
R_{int}	0.0748
Parameters	884
Restraints	901
Largest Peak/eÅ³	0.3950
Deepest Hole/eÅ³	-0.2983
GooF	1.0868
R_1 (I≥2σ(I) / all)	0.0585 / 0.0741
wR_2 (I≥2σ(I) / all)	0.1167 / 0.1238

Structure Quality Indicators

**Reflections:**	d min (CuKα) 2Θ=154.6° 0.79	I/σ(I) 17.6	Rint m=4.25 7.48%	Full 135.4° 97% to 154.6° 99.3
**Refinement:**	Shift -0.002	Max Peak 0.4	Min Peak -0.3	GooF 1.087

A colourless prism-shaped crystal with dimensions 0.19 × 0.10 × 0.03 mm was mounted on a nylon loop with NVH oil. Data were collected using a XtaLAB Synergy, Dualflex, HyPix diffractometer operating at T = 100.0(1) K.

Data were measured using ω scans with Cu K_{α} radiation. The diffraction pattern was indexed and the total number of runs and images was based on the strategy calculation from the program CrysAlis^{Pro} system (CCD 44.57a 64-bit (release 20-06-2024)). The maximum resolution achieved was Θ = 77.30° (0.79 Å).

The unit cell was refined using CrysAlis^{Pro} on 8674 reflections, 25% of the observed reflections.

Data reduction, scaling and absorption corrections were performed using CrysAlis^{Pro}. The final completeness is 99.54 % out to 77.30° in Θ. A numerical absorption correction based on gaussian integration over a multifaceted crystal model was performed using CrysAlisPro 1.171.44.57a (Rigaku Oxford Diffraction, 2022). An empirical absorption correction using spherical harmonics, implemented in SCALE3 ABSPACK scaling algorithm was also applied. The absorption coefficient μ of this material is 0.541 mm^{-1} at this wavelength (λ = 1.54184Å) and the minimum and maximum transmissions are 0.812 and 1.000.

The structure was solved in the space group $P2_1$ (# 4) by ShelXT 2018/2 (Sheldrick, 2018). It was refined by full matrix least squares minimisation on $|F|^2$ using version of olex2.refine 1.5-dev (Bourhis et al., 2015). All non-hydrogen atoms were refined anisotropically.

All hydrogen atoms were located in difference Fourier maps and refined, including anisotropic displacement parameters, using Hirshfeld atom refinement (HAR). Most hydrogen atom positions were calculated geometrically and refined using the riding model, but some hydrogen atoms were refined freely.

SOFTWARE: LJ-04-341PRP-A.tscb PARTITIONING: NoSpherA2 INT ACCURACY: Normal METHOD: PBE BASIS SET: def2-SVP CHARGE: 0 MULTIPLICITY: 1 SOLVATION: None DATE: 2026-06-29_16-44-59

There is a single formula unit in the asymmetric unit, which is represented by the reported sum formula. In other words: Z is 2 and Z' is 1. The moiety formula is $C_{46}H_{60}O_4$.

The molecule is a large carbocyclic macrocycle and contains no nitrogen. A 26-membered all-carbon ring (C1 to C26) incorporates two 1,3-disubstituted (meta) benzene rings, defined by C4/C5/C6/C27/C28/C29 and C17/C18/C19/C30/C31/C32, which act as rigid meta-phenylene bridges. The two aromatic units are linked by two unsaturated aliphatic chains.

Each bridging chain carries one α,β-unsaturated ethyl ester and one aryl-conjugated trisubstituted alkene. The ester groups are at C36 (O1, O2; conjugated to the C11=C12 double bond) and C42 (O3, O4; conjugated to the C24=C25 double bond), each present as an ethyl ester (O1-C37-C38 and O3-C43-C44). The aryl-conjugated alkenes are C7=C8 and C20=C21, each bearing a vinylic methyl group (C35 and C41, respectively). The four ring C=C bond lengths lie between 1.344(3) and 1.354(3) Å, consistent with localised double bonds.

The ring contains two sp^3 stereocentres, C1 and C14, each bearing an isopropenyl group (C1-C33(=C45)(C34) and C14-C39(=C40)(C46)). C1 and C14 have the same relative configuration, so the molecule possesses

approximate non-crystallographic twofold (C2) symmetry relating its two halves. The macrocycle adopts an open, oval conformation around an empty cavity, with the two ester groups projecting outward. There are no classical hydrogen-bond donors in the molecule (the only acceptors are the four ester oxygen atoms), so the crystal packing is governed by van der Waals interactions and no solvent is present.

After removal of the inversion-twin parameter, the absolute structure was reassessed using an independent refinement (SHELXL) together with a Bijvoet-pair analysis (PLATON). The Flack parameter is 0.09(16) (Parsons z = 0.11(15)) and the Hooft y parameter is 0.17(14), evaluated over 3650 Bijvoet pairs. These central values lie close to zero, rather than near the racemic value of 0.5 obtained from the earlier twinned refinement, indicating that the absolute structure of the present model is not inverted. The two stereocentres C1 and C14 are of the same configuration. Because the structure contains only the light atoms C, H and O, the anomalous-dispersion signal at Cu Kα is weak (Friedif = 25) and the standard uncertainties remain large; the diffraction data are therefore consistent with, but do not independently prove, the absolute configuration, which is assigned from the synthesis.

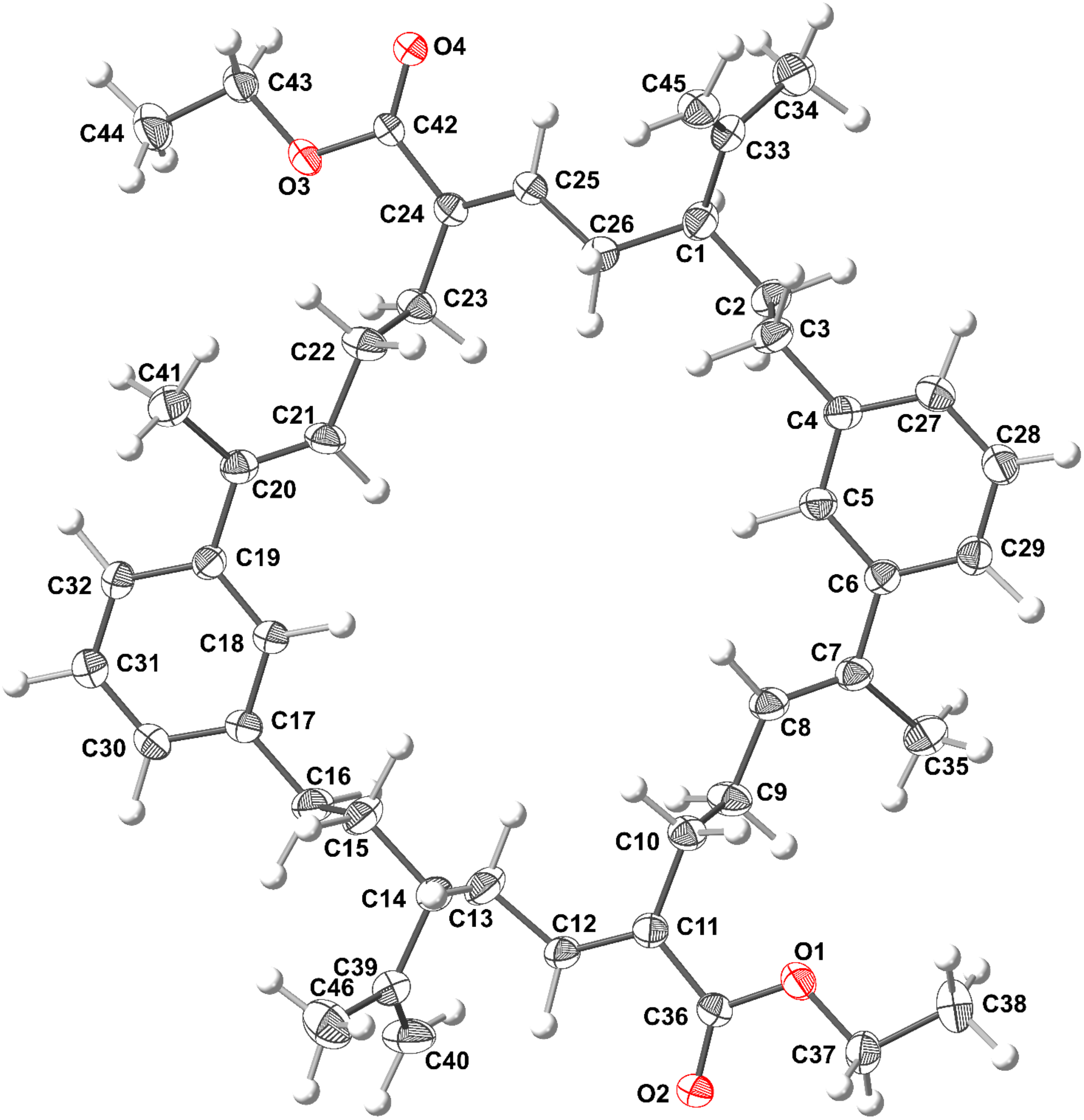

Figure 1 Thermal ellipsoid representation of the asymmetric unit at the 50% probability level, excluding the H atoms, which are shown as spheres.

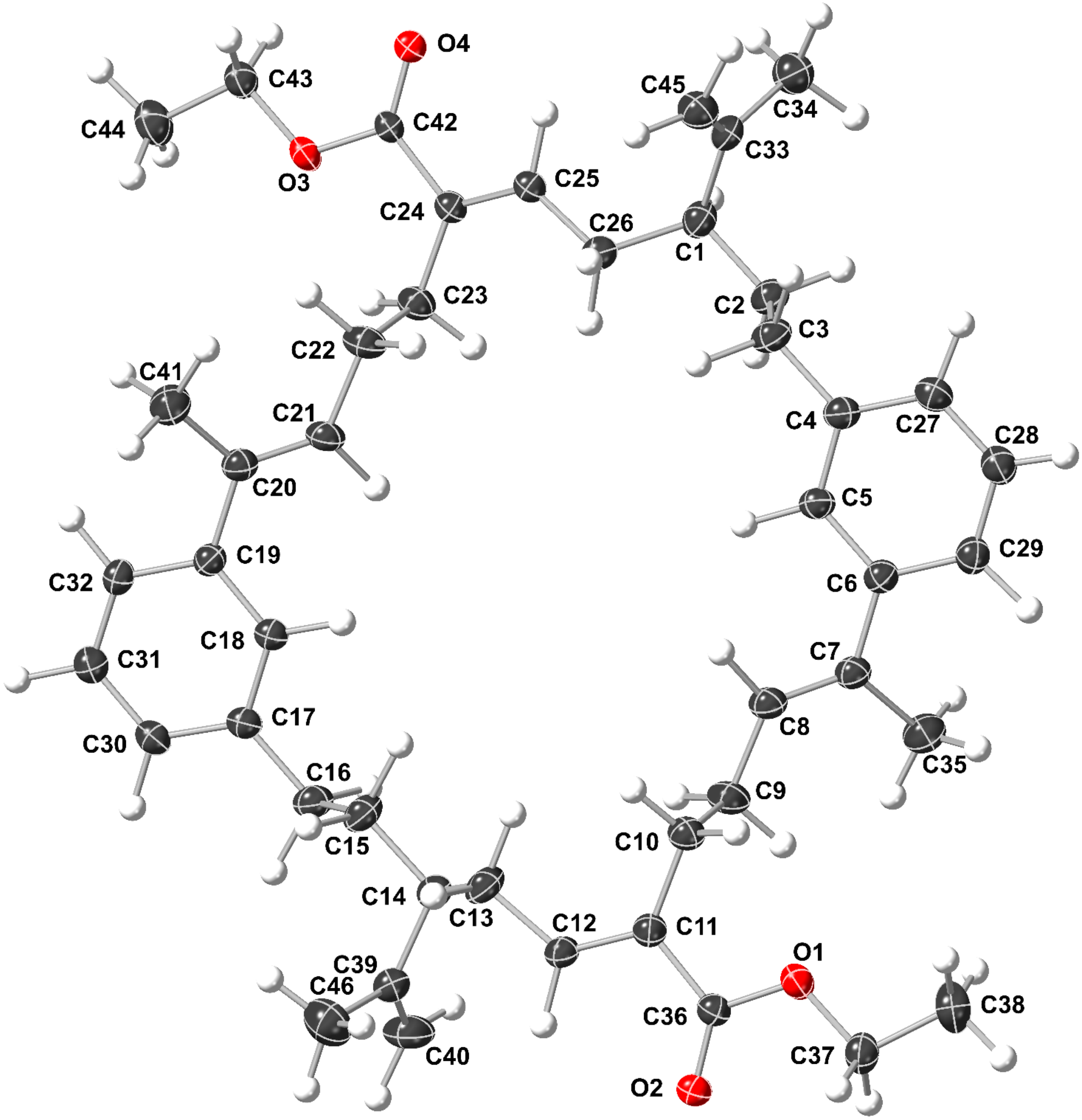

Figure 2 Thermal ellipsoid representation of the asymmetric unit at the 50% probability level, excluding the H atoms, which are shown as spheres.

Data Plots: Diffraction Data

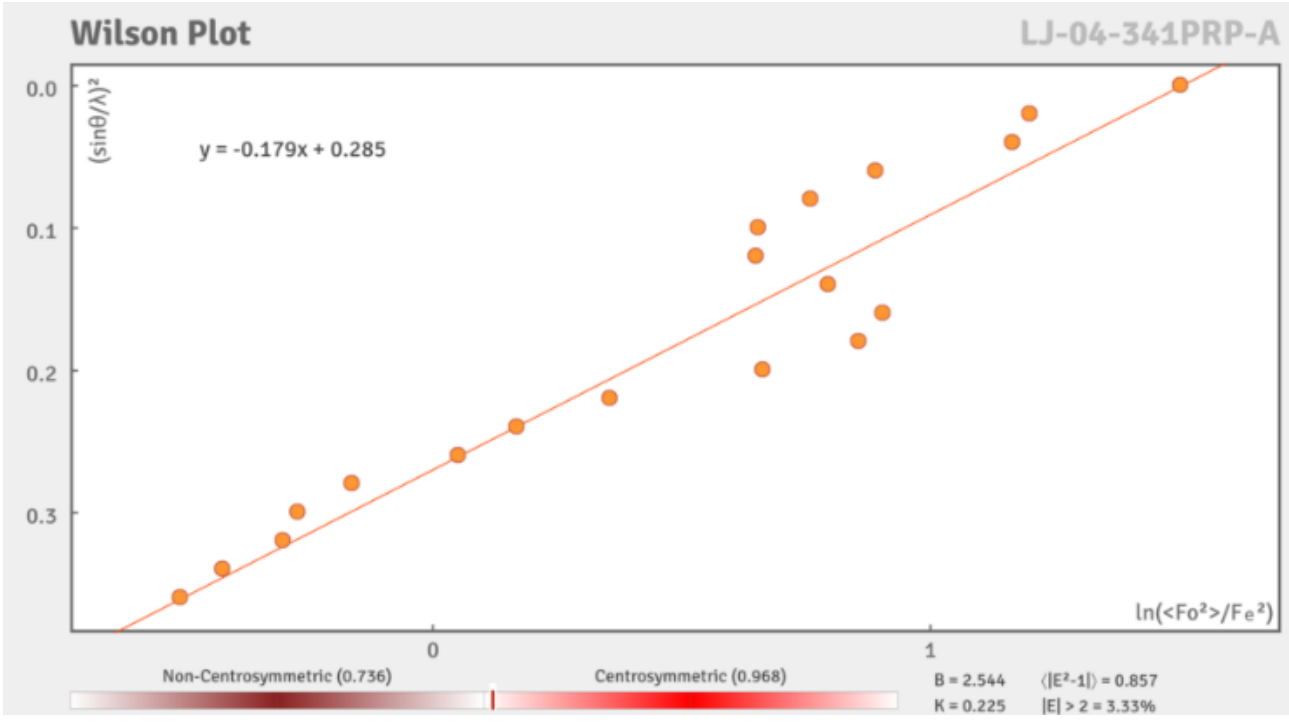

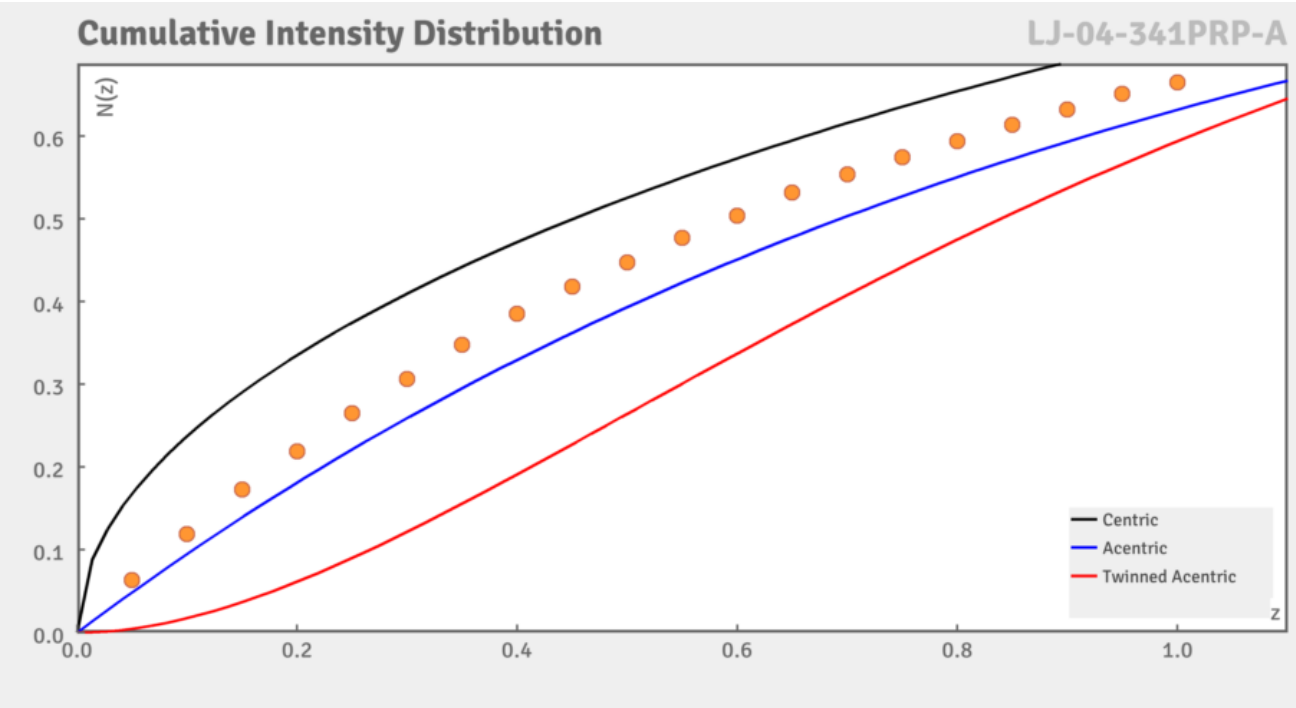

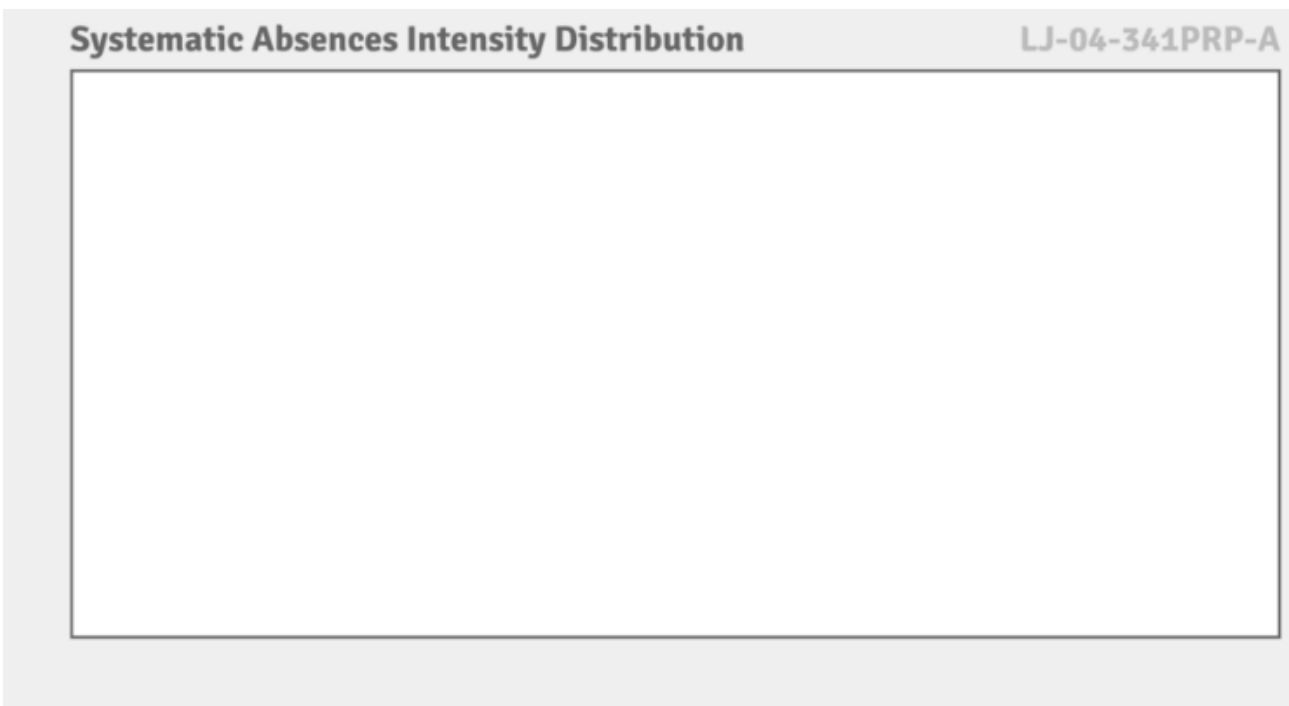

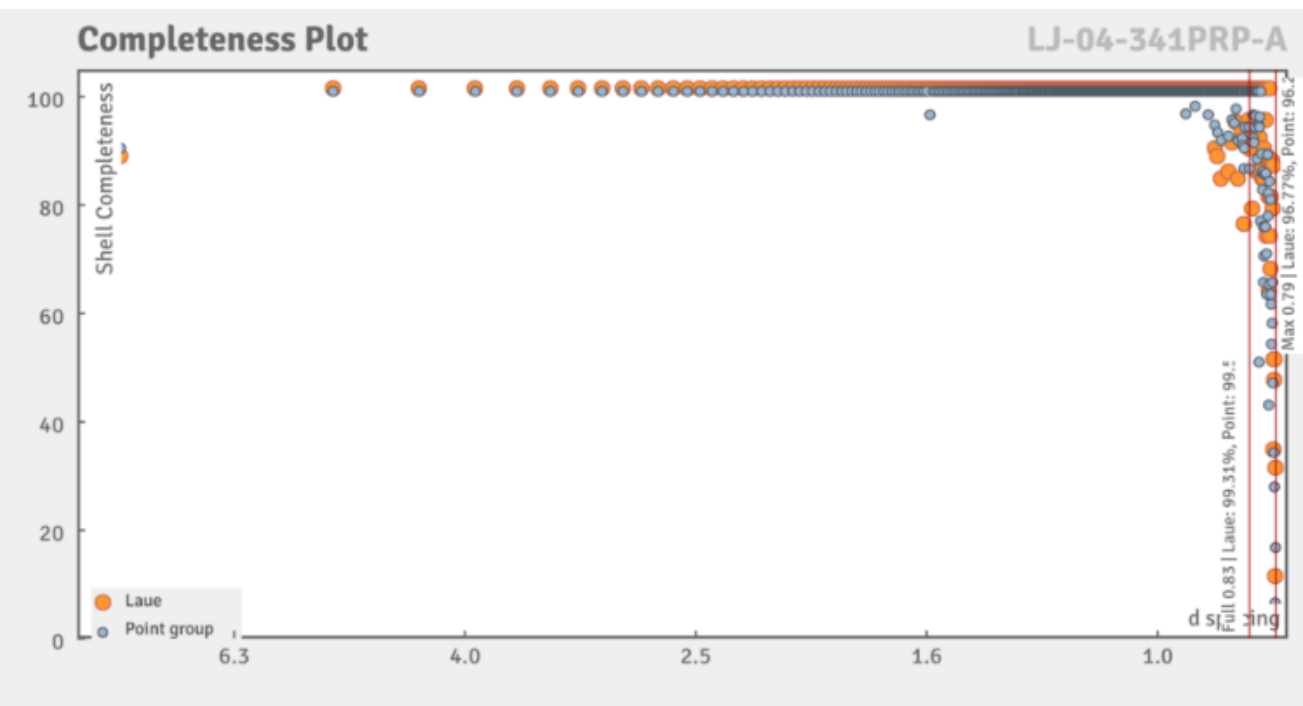

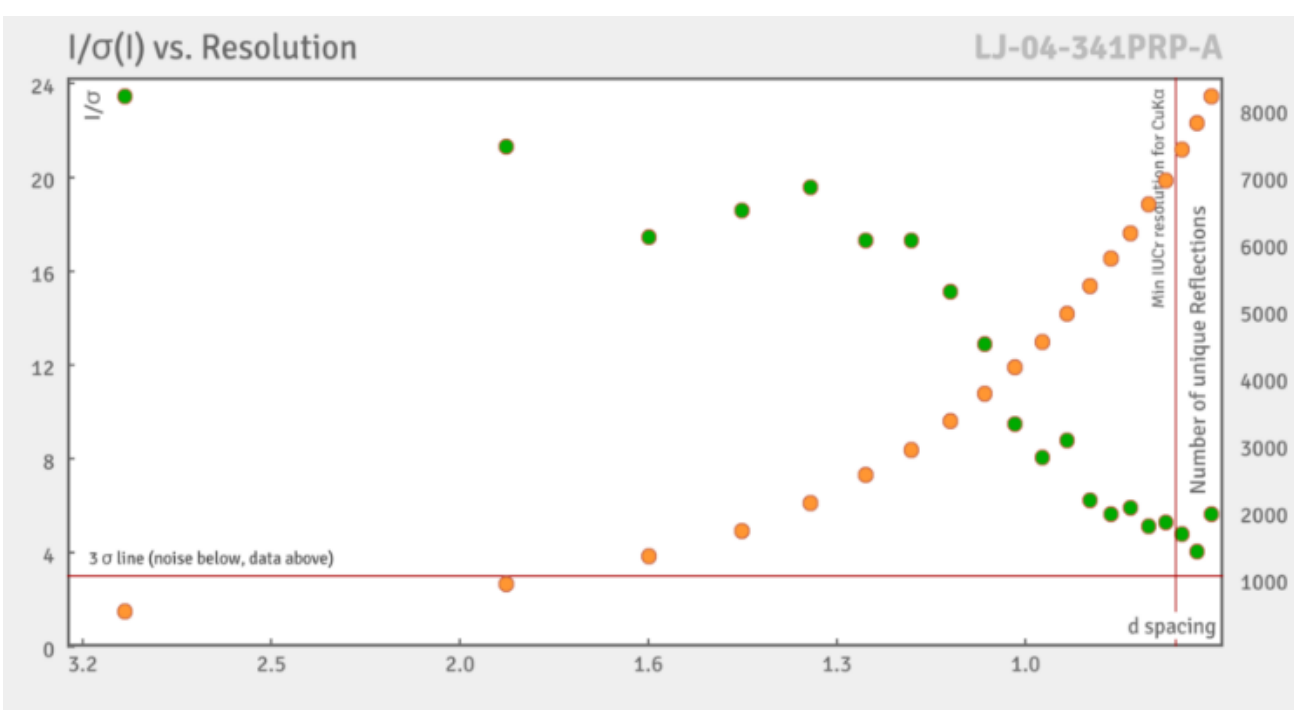

Data Plots: Refinement and Data

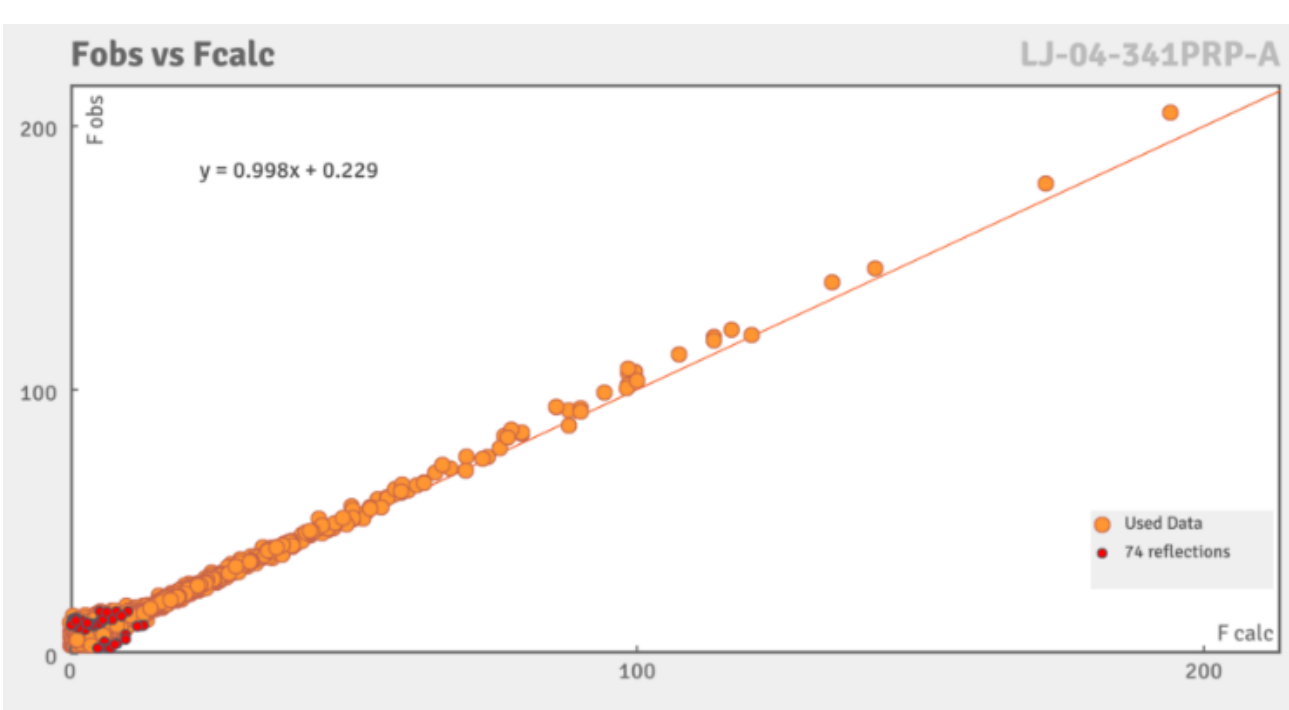

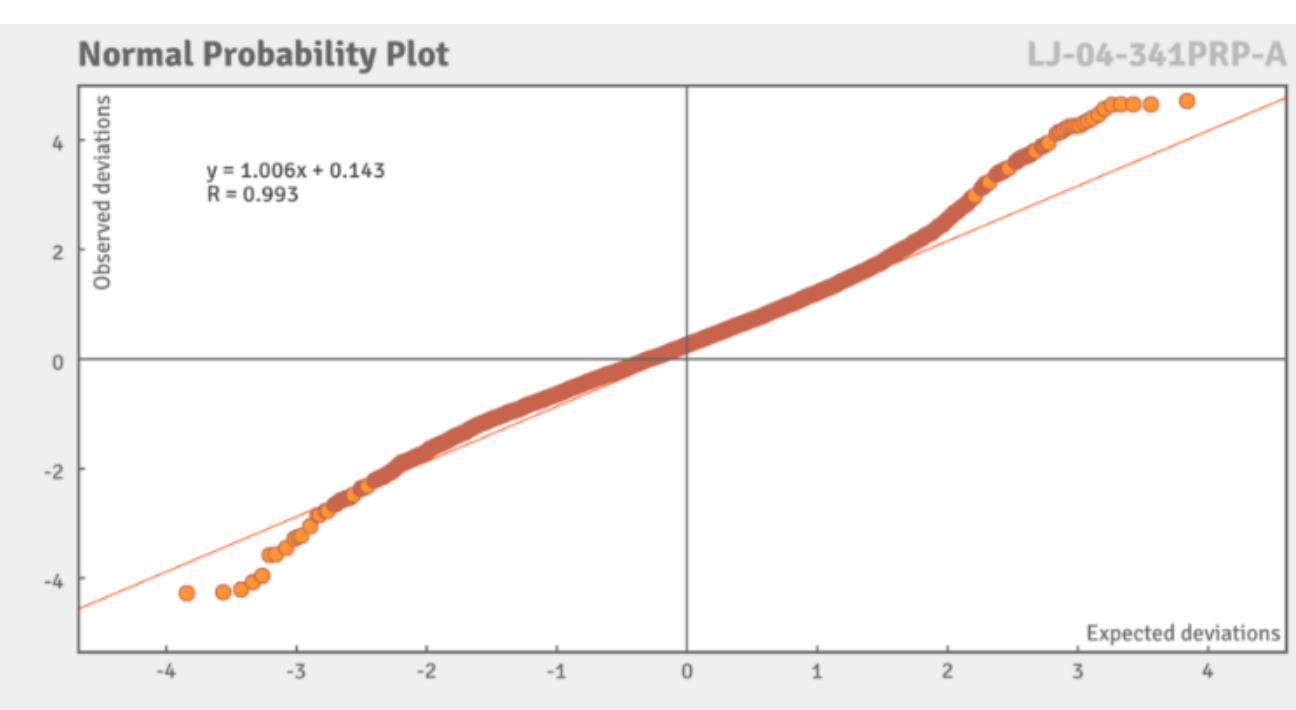

Reflection Statistics

Total reflections (after filtering)	34664	Unique reflections	8163
Completeness	0.962	Mean I/σ	11.42

hkl_{max} collected	(20, 8, 22)	hkl_{min} collected	(-20, -8, -23)
hkl_{max} used	(19, 8, 23)	hkl_{min} used	(-20, -8, 0)
Lim d_{max} collected	999.0	Lim d_{min} collected	0.77
d_{max} used	15.73	d_{min} used	0.79
Friedel pairs	4172	Friedel pairs merged	0
Inconsistent equivalents	122	R_{int}	0.0748
R_{sigma}	0.0569	Intensity transformed	0
Omitted reflections	0	Omitted by user (OMIT hkl)	94
Multiplicity	(4000, 3009, 2071, 1124, 654, 501, 346, 216, 139, 94, 65, 34, 18, 3, 1)	Maximum multiplicity	16
Removed systematic absences	0	Filtered off (Shel/OMIT)	0

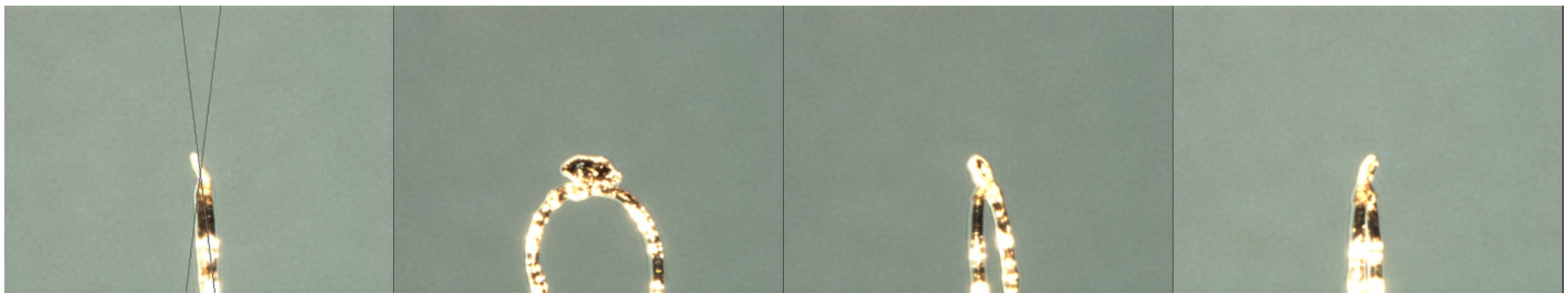

Table 1: Fractional Atomic Coordinates (×10⁴) and Equivalent Isotropic Displacement Parameters (Å²×10³) for *E,E*-**41** . U_{eq} is defined as 1/3 of the trace of the orthogonalised U_{ij}.

Atom	x	y	z	U_{eq}
O1	8709.1(11)	-1511(3)	2201.0(9)	29.3(4)
O3	1442.4(11)	11652(3)	2690.4(9)	30.6(4)
O2	9596.5(11)	-597(3)	3198.4(9)	33.0(4)
O4	545.4(11)	10666(3)	1711.5(9)	32.1(4)
C2	2392.6(16)	2638(4)	826.7(14)	31.6(6)
C5	4337.6(15)	811(4)	967.2(13)	27.5(5)
C4	3652.9(16)	1150(4)	411.9(13)	29.5(5)
C31	6081.3(17)	12290(4)	5169.1(15)	32.1(6)
C33	1268.9(16)	4875(3)	175.0(13)	27.2(5)
C23	2595.8(15)	8644(4)	2690.7(13)	29.0(5)
C14	7851.5(16)	4887(4)	4138.2(13)	30.0(5)
C22	3342.8(16)	9940(4)	2537.4(14)	33.0(6)
C9	6826.4(15)	295(4)	2447.1(13)	32.3(6)
C36	8955.3(15)	-314(4)	2766.6(12)	26.1(5)
C19	5261.8(15)	11155(4)	4042.3(12)	24.9(5)
C32	5437.4(16)	12586(4)	4585.5(14)	28.6(5)
C7	5575.1(16)	-1231(4)	1582.7(13)	27.6(5)
C11	8356.6(16)	1394(4)	2771.6(12)	26.6(5)
C12	8576.8(16)	2750(4)	3302.0(13)	27.6(5)
C27	3448.5(16)	-315(4)	-118.2(14)	31.6(5)
C20	4548.0(16)	11452(4)	3430.7(13)	27.5(5)
C6	4835.9(15)	-928(4)	1000.4(13)	25.8(5)
C38	8852(2)	-4362(4)	1487.2(16)	44.1(7)
C42	1199.5(15)	10437(4)	2133.7(12)	24.9(5)
C29	4613.0(16)	-2366(4)	464.6(14)	29.9(5)
C21	4091.3(16)	9877(4)	3138.6(13)	30.0(5)
C8	6045.7(16)	316(4)	1880.4(14)	30.0(5)
C39	8600.6(15)	4891(4)	4756.0(14)	30.2(5)
C24	1841.2(15)	8831(4)	2090.3(12)	25.6(5)
C10	7556.9(15)	1471(4)	2213.8(13)	28.9(5)
C1	1805.3(17)	4425(4)	888.3(13)	30.3(5)
C3	3110.4(17)	3013(4)	407.7(15)	32.9(6)
C16	6931.3(17)	7211(4)	4763.7(14)	32.9(6)

Atom	x	y	z	U_{eq}
C35	5765(2)	-3353(4)	1820.7(17)	43.2(7)
C25	1732.1(16)	7774(4)	1476.8(13)	28.1(5)
C26	2309.8(16)	6215(4)	1268.9(14)	33.0(6)
C13	8105.8(18)	4625(4)	3395.9(14)	36.6(6)
C37	9290.1(17)	-3092(4)	2097.7(15)	34.0(6)
C45	1489.0(18)	6202(4)	-306.3(15)	34.1(6)
C46	9291.5(19)	6319(5)	4723.1(18)	51.7(8)
C28	3928.2(17)	-2055(4)	-93.3(14)	32.5(6)
C43	858.7(16)	13271(4)	2757.7(14)	32.9(6)
C18	5763.9(15)	9430(4)	4110.9(13)	28.4(5)
C41	4375(2)	13555(4)	3174.0(16)	42.1(7)
C17	6411.0(15)	9110(4)	4701.1(13)	28.8(5)
C30	6573.5(16)	10568(4)	5226.5(14)	31.1(6)
C15	7325.1(18)	6814(4)	4103.4(14)	34.7(6)
C40	8628.3(19)	3675(5)	5316.8(17)	44.6(7)
C44	1207(2)	14392(5)	3426.8(17)	50.7(8)
C34	476.4(18)	3707(4)	-3.2(17)	42.1(7)

Table 2: Anisotropic Displacement Parameters (×10[4]) for *E,E*-**40** . The anisotropic displacement factor exponent takes the form: $-2\pi^2[h^2a^{*2} \times U_{11} + ... + 2hka^* \times b^* \times U_{12}]$

Atom	U_{11}	U_{22}	U_{33}	U_{23}	U_{13}	U_{12}
O1	28.3(9)	30.3(9)	28.0(8)	-2.8(6)	1.5(6)	-4.7(6)
O3	26.2(9)	31.8(9)	31.1(9)	-2.6(7)	-2.5(6)	-9.9(7)
O2	31.3(9)	32.8(10)	30.8(9)	6.5(7)	-6.1(7)	-5.4(7)
O4	30.2(9)	28.4(9)	33.3(9)	5.5(7)	-6.2(7)	-7.4(7)
C2	33.2(13)	26.9(12)	34.3(13)	4.0(9)	5.0(9)	5.4(9)
H2a	39(8)	39(9)	37(3)	5(4)	3.9(19)	9(2)
H2b	42(7)	32(5)	44(8)	-1(3)	5(3)	2(3)
C5	23.2(11)	30.6(13)	28.0(11)	3.9(8)	2.4(8)	-0.7(8)
H5	47(13)	40(8)	39(9)	8(5)	-5(6)	-10(4)
C4	26.1(11)	29.3(12)	31.9(12)	2.7(8)	1.6(8)	1.9(8)
C31	33.9(13)	29.9(13)	31.3(12)	-0.3(8)	2.1(9)	-3.3(9)
H31	32(12)	38(8)	38(7)	-3(5)	5(5)	-10(4)
C33	30.5(11)	21.1(11)	30.1(11)	2.4(8)	6.0(8)	0.6(8)
C23	29.9(12)	32.6(13)	22.9(11)	-0.8(9)	0.0(8)	3.7(9)
H23a	38(9)	34(3)	34(9)	1.6(18)	3(4)	4.9(17)
H23b	25(8)	33(8)	23(3)	-2(4)	-1(2)	3(2)
C14	30.2(12)	26.3(12)	31.9(11)	5.0(8)	0.9(8)	-0.2(8)
H14	41(7)	34(5)	44(8)	-2(2)	1(3)	5(2)
C22	27.3(12)	40.4(15)	28.9(13)	-1.4(10)	-1.8(9)	8.5(11)
H22a	40(12)	57(10)	34(6)	2(5)	4(4)	6(3)
H22b	39(11)	43(3)	62(13)	3(2)	15(6)	16(2)
C9	29.1(12)	37.0(15)	28.6(12)	-1.2(9)	-1.0(8)	10.4(10)
H9a	24(8)	48(8)	32(4)	-3(4)	-1(2)	4(2)
H9b	30(8)	38(3)	36(9)	-0.5(18)	-1(4)	11.5(19)
C36	25.7(11)	27.0(11)	24.6(10)	0.0(8)	1.1(7)	0.0(7)
C19	26.2(11)	23.4(11)	25.0(10)	2.5(8)	4.2(8)	0.1(7)
C32	31.6(12)	24.8(12)	29.0(11)	3.2(9)	4.5(8)	-4.4(8)
H32	77(16)	47(8)	52(13)	35(6)	-26(7)	-26(5)
C7	27.9(11)	25.5(12)	28.4(11)	1.7(7)	2.6(8)	5.0(8)
C11	29.6(12)	25.7(12)	23.4(11)	1.2(8)	2.0(8)	1.5(8)
C12	31.0(12)	26.8(11)	23.2(11)	6.8(9)	-0.6(9)	0.4(8)
C27	23.5(11)	34.9(13)	35.3(13)	-2.5(8)	2.5(9)	-1.3(9)
H27	30(8)	43(11)	42(9)	1(4)	-4(4)	-4(5)

Atom	U_{11}	U_{22}	U_{33}	U_{23}	U_{13}	U_{12}
C20	27.2(11)	28.7(12)	26.0(10)	3.0(8)	2.9(8)	2.2(8)
C6	24.1(10)	25.3(12)	28.4(11)	0.3(7)	6.1(8)	1.4(7)
C38	54.7(18)	35.9(15)	42.3(14)	-9.9(12)	9.9(12)	-11.3(11)
H38a	64(10)	42(8)	46(4)	-11(4)	9(3)	-8(3)
H38b	69(7)	43(5)	63(10)	-2(3)	17(4)	-12(3)
H38c	59(4)	48(9)	56(10)	-13(3)	14(3)	-9(4)
C42	23.9(10)	22.9(11)	25.4(10)	-1.2(7)	-2.3(7)	-4.1(7)
C29	31.3(12)	26.0(12)	32.4(12)	-1.2(9)	5.8(8)	-1.5(8)
H29	49(12)	36(7)	42(12)	11(4)	0(6)	-7(4)
C21	26.8(12)	33.2(13)	26.7(12)	1.4(9)	-4.5(9)	5.5(9)
C8	28.1(11)	26.7(13)	33.0(12)	1.4(8)	-0.9(9)	7.4(9)
C39	27.5(11)	28.9(12)	33.7(12)	3.9(8)	3.5(8)	-0.4(8)
C24	26.7(11)	26.2(12)	22.1(10)	0.4(8)	-0.6(7)	-1.6(7)
C10	31.4(12)	28.9(13)	24.7(12)	0.7(10)	0.4(9)	5.2(10)
H10a	54(11)	32(3)	51(11)	8(2)	17(5)	12(2)
H10b	34(11)	41(8)	28(5)	3(4)	1(3)	2(3)
C1	36.7(12)	25.1(11)	29.1(11)	3.6(8)	5.5(8)	0.5(8)
H1	41(6)	34(9)	35(7)	4(3)	10(3)	4(3)
C3	28.4(12)	26.2(12)	43.8(14)	3.2(8)	5.7(9)	8.5(9)
H3a	32(9)	38(10)	44(3)	5(4)	5.9(19)	11(2)
H3b	40(7)	31(5)	60(9)	-1(3)	2(3)	4(3)
C16	31.0(13)	35.3(14)	30.7(12)	8.0(9)	0.9(9)	2.5(9)
H16a	39(6)	54(11)	38(5)	6(3)	-5(2)	2(3)
H16b	42(7)	40(5)	41(10)	1(3)	5(4)	2(3)
C35	49.9(18)	31.4(14)	44.3(16)	5.1(11)	-2.8(13)	9.3(11)
H35a	56(4)	47(9)	67(10)	-1(3)	3(3)	13(4)
H35b	74(10)	45(8)	55(6)	12(4)	8(3)	5(3)
H35c	51(6)	47(10)	47(4)	8(3)	-3(3)	8(3)
C25	28.0(12)	28.1(12)	25.5(11)	3.0(9)	-2.8(8)	-3.6(8)
C26	31.1(13)	31.7(13)	33.8(13)	6.5(8)	-1.0(9)	-3.6(9)
H26a	37(7)	48(9)	34(4)	14(3)	0(2)	-2(3)
H26b	35(7)	33(8)	33(7)	3(3)	1(3)	-6(3)
C13	47.8(15)	28.2(12)	32.5(12)	15.4(10)	3.9(9)	3.6(9)
H13a	60(7)	34(5)	37(9)	8(3)	9(3)	1(3)
H13b	54(4)	40(10)	42(6)	17(3)	-3(2)	2(3)
C37	34.8(13)	29.6(12)	38.6(14)	-1.8(9)	9.1(10)	-7.6(9)
H37a	41(4)	48(9)	58(10)	-8(2)	16(3)	-5(4)
H37b	41(9)	44(7)	46(4)	1(3)	10(3)	2(3)
C45	37.1(14)	33.3(14)	30.4(12)	-2.3(10)	1.2(10)	7.6(10)
C46	40.6(16)	55.5(19)	57.7(19)	-15.3(12)	5.0(12)	-0.1(15)
H46a	55(9)	70(11)	62(4)	-12(4)	13(3)	0(3)
H46b	57(9)	58(3)	84(11)	-10(2)	12(4)	0(2)
H46c	39(6)	61(10)	56(5)	-11(3)	7(2)	-3(3)
C28	34.1(12)	31.6(13)	30.9(12)	-3.0(9)	3.1(9)	-2.7(9)
H28	94(18)	62(11)	64(11)	35(7)	-35(7)	-34(5)
C43	28.7(12)	35.1(14)	33.8(13)	-0.1(9)	2.2(9)	-11.0(9)
H43a	49(9)	40(7)	39(5)	1(3)	5(3)	-8(3)
H43b	32(3)	41(9)	54(10)	-2(2)	7(2)	-10(4)
C18	27.3(11)	28.9(12)	26.9(11)	3.5(8)	-1.4(8)	-2.1(8)
H18	45(15)	48(9)	42(8)	19(6)	-15(6)	-19(4)
C41	52.6(18)	32.2(14)	37.6(15)	4.4(11)	-3.3(13)	6.0(10)
H41a	62(9)	51(11)	41(3)	4(4)	-6(2)	7(2)
H41b	72(8)	48(9)	49(7)	15(3)	7(4)	5(3)
H41c	58(4)	44(8)	58(11)	-2(3)	0(3)	6(4)
C17	24.6(11)	31.4(12)	28.5(11)	2.4(8)	-0.4(8)	-1.8(8)
C30	26.6(12)	33.9(13)	30.6(12)	-2.2(8)	-1.2(9)	-4.1(8)
H30	33(11)	46(11)	36(6)	1(5)	-6(4)	-3(4)
C15	42.9(15)	28.4(12)	32.9(13)	12.6(10)	6.6(9)	3.2(9)

Atom	U_{11}	U_{22}	U_{33}	U_{23}	U_{13}	U_{12}
H15a	50(6)	39(10)	40(5)	13(3)	0(2)	2(3)
H15b	54(7)	35(5)	49(10)	5(3)	9(4)	6(3)
C40	33.2(15)	47.9(17)	48.7(16)	3.1(12)	-3.9(11)	17.3(12)
C44	47.9(17)	56(2)	45.4(16)	2.1(14)	-1.1(12)	-24.9(13)
H44a	51(3)	72(11)	73(11)	-2(2)	4(2)	-27(4)
H44b	60(8)	60(5)	63(11)	8(3)	5(4)	-24(3)
H44c	68(11)	66(8)	52(5)	3(4)	5(3)	-19(3)
C34	35.2(14)	35.4(15)	54.4(17)	-4.4(10)	4.8(11)	0.8(13)
H34a	47(10)	37(3)	78(11)	-2.4(18)	8(4)	4(2)
H34b	40(7)	47(10)	58(8)	-1(3)	8(3)	1(4)
H34c	49(9)	42(11)	55(3)	-1(4)	2(2)	0(2)
H8	50(13)	26(3)	49(15)	-1(2)	-24(7)	7(2)
H21	45(16)	35(4)	67(18)	0(3)	-23(10)	13(3)
H12	37(6)	36(11)	31(9)	11(4)	-7(4)	-4(5)
H25	41(8)	62(16)	33(7)	23(6)	-12(4)	-17(6)
H45a	42(12)	57(15)	33(4)	-9(7)	-2(3)	12(4)
H40a	36(8)	84(19)	50(8)	-4(6)	-6(4)	23(6)
H45b	110(20)	70(12)	46(11)	-54(8)	-33(6)	32(5)
H40b	113(15)	210(30)	129(18)	-110(10)	-77(8)	127(11)

Table 3: Bond Lengths in Å for *E,E*-**40** .

Atom	Atom	Length/Å
O1	C36	1.340(3)
O1	C37	1.446(3)
O3	C42	1.334(3)
O3	C43	1.453(3)
O2	C36	1.207(3)
O4	C42	1.208(3)
C2	C1	1.540(3)
C2	C3	1.524(4)
C5	C4	1.395(3)
C5	C6	1.409(3)
C4	C27	1.401(4)
C4	C3	1.522(3)
C31	C32	1.384(4)
C31	C30	1.392(4)
C33	C1	1.495(3)
C33	C45	1.363(4)
C33	C34	1.477(4)
C23	C22	1.545(3)
C23	C24	1.509(3)
C14	C39	1.518(3)
C14	C13	1.537(4)
C14	C15	1.539(3)
C22	C21	1.498(3)
C9	C8	1.494(3)
C9	C10	1.536(3)
C36	C11	1.495(3)

Atom	Atom	Length/Å
C19	C32	1.398(3)
C19	C20	1.487(3)
C19	C18	1.402(3)
C7	C6	1.480(3)
C7	C8	1.347(4)
C7	C35	1.508(4)
C11	C12	1.354(3)
C11	C10	1.508(3)
C12	C13	1.493(3)
C27	C28	1.394(4)
C20	C21	1.347(4)
C20	C41	1.503(4)
C6	C29	1.399(4)
C38	C37	1.504(4)
C42	C24	1.500(3)
C29	C28	1.395(4)
C39	C46	1.471(4)
C39	C40	1.332(4)
C24	C25	1.344(3)
C1	C26	1.551(3)
C16	C17	1.515(4)
C16	C15	1.516(4)
C25	C26	1.493(3)
C43	C44	1.493(4)
C18	C17	1.398(3)
C17	C30	1.386(4)

Table 4: Bond Angles in ° for *E,E*-**40** .

Atom	Atom	Atom	Angle/°
C37	O1	C36	116.28(19)
C43	O3	C42	115.12(19)
C3	C2	C1	115.7(2)
C6	C5	C4	122.1(2)
C27	C4	C5	118.5(2)
C3	C4	C5	120.6(2)
C3	C4	C27	120.8(2)
C30	C31	C32	120.8(2)
C45	C33	C1	124.0(2)
C34	C33	C1	115.5(2)
C34	C33	C45	120.4(2)
C24	C23	C22	110.7(2)
C13	C14	C39	113.8(2)
C15	C14	C39	112.5(2)
C15	C14	C13	106.7(2)
C21	C22	C23	112.5(2)
C10	C9	C8	111.7(2)
O2	C36	O1	122.7(2)
C11	C36	O1	111.5(2)
C11	C36	O2	125.8(2)
C20	C19	C32	120.4(2)
C18	C19	C32	117.5(2)
C18	C19	C20	122.1(2)
C19	C32	C31	120.8(2)
C8	C7	C6	121.2(2)
C35	C7	C6	116.4(2)
C35	C7	C8	122.4(2)
C12	C11	C36	115.9(2)
C10	C11	C36	119.1(2)
C10	C11	C12	124.9(2)
C13	C12	C11	125.7(2)
C28	C27	C4	120.4(2)
C21	C20	C19	120.0(2)
C41	C20	C19	116.7(2)

Atom	Atom	Atom	Angle/°
C41	C20	C21	123.3(2)
C7	C6	C5	121.2(2)
C29	C6	C5	118.0(2)
C29	C6	C7	120.8(2)
O4	C42	O3	122.8(2)
C24	C42	O3	112.06(19)
C24	C42	O4	125.2(2)
C28	C29	C6	120.6(2)
C20	C21	C22	126.3(2)
C7	C8	C9	128.8(2)
C46	C39	C14	117.7(2)
C40	C39	C14	121.5(3)
C40	C39	C46	120.8(3)
C42	C24	C23	118.8(2)
C25	C24	C23	124.8(2)
C25	C24	C42	116.1(2)
C11	C10	C9	112.02(19)
C33	C1	C2	110.5(2)
C26	C1	C2	111.4(2)
C26	C1	C33	115.5(2)
C4	C3	C2	109.9(2)
C15	C16	C17	112.6(2)
C26	C25	C24	127.2(2)
C25	C26	C1	111.7(2)
C12	C13	C14	115.1(2)
C38	C37	O1	107.2(2)
C29	C28	C27	120.3(3)
C44	C43	O3	107.6(2)
C17	C18	C19	122.1(2)
C18	C17	C16	120.7(2)
C30	C17	C16	120.5(2)
C30	C17	C18	118.8(2)
C17	C30	C31	119.9(2)
C16	C15	C14	114.5(2)

Table 5: Torsion Angles in ° for *E,E*-**40** .

Atom	Atom	Atom	Atom	Angle/°
O1	C36	C11	C12	-176.5(2)
O1	C36	C11	C10	5.0(2)
O3	C42	C24	C23	6.4(2)
O3	C42	C24	C25	-167.8(2)
O2	C36	O1	C37	-4.8(3)
O2	C36	C11	C12	1.7(3)
O2	C36	C11	C10	-176.8(2)
O4	C42	O3	C43	0.5(3)
O4	C42	C24	C23	-175.4(2)
O4	C42	C24	C25	10.5(3)
C2	C1	C33	C45	-92.4(2)
C2	C1	C33	C34	85.1(2)
C2	C1	C26	C25	-167.4(2)
C2	C3	C4	C5	-87.9(2)
C2	C3	C4	C27	88.5(2)
C5	C4	C27	C28	-0.9(3)
C5	C6	C7	C8	-32.0(3)

Atom	Atom	Atom	Atom	Angle/˚
C5	C6	C7	C35	147.8(3)
C5	C6	C29	C28	1.4(3)
C4	C5	C6	C7	178.2(2)
C4	C5	C6	C29	-1.7(3)
C4	C27	C28	C29	0.6(3)
C4	C3	C2	C1	178.2(2)
C31	C32	C19	C20	177.7(2)
C31	C32	C19	C18	-0.4(3)
C31	C30	C17	C16	-178.9(2)
C31	C30	C17	C18	1.6(3)
C33	C1	C2	C3	70.0(2)
C33	C1	C26	C25	65.6(2)
C23	C22	C21	C20	-134.3(2)
C23	C24	C25	C26	1.0(3)
C14	C13	C12	C11	125.7(2)
C14	C15	C16	C17	-179.3(2)
C22	C23	C24	C42	-89.5(2)
C22	C23	C24	C25	84.2(2)
C22	C21	C20	C19	178.2(3)
C22	C21	C20	C41	-1.4(3)
C9	C8	C7	C6	-177.3(3)
C9	C8	C7	C35	2.9(4)
C9	C10	C11	C36	86.0(2)
C9	C10	C11	C12	-92.3(2)
C36	O1	C37	C38	174.3(2)
C36	C11	C12	C13	176.8(2)
C19	C32	C31	C30	0.5(3)
C19	C18	C17	C16	178.9(2)
C19	C18	C17	C30	-1.6(3)
C32	C31	C30	C17	-1.1(3)
C32	C19	C20	C21	-145.0(2)
C32	C19	C20	C41	34.6(3)
C32	C19	C18	C17	0.9(3)
C7	C6	C29	C28	-178.5(2)
C7	C8	C9	C10	127.6(3)
C11	C36	O1	C37	173.47(19)
C11	C10	C9	C8	-177.9(2)
C12	C13	C14	C39	59.3(3)
C12	C13	C14	C15	-176.1(2)
C27	C4	C5	C6	1.4(3)
C27	C28	C29	C6	-0.9(3)
C20	C19	C18	C17	-177.2(2)
C6	C5	C4	C3	177.9(2)
C42	O3	C43	C44	176.8(2)
C42	C24	C25	C26	174.7(2)
C29	C6	C7	C8	147.9(2)
C29	C6	C7	C35	-32.3(3)
C21	C22	C23	C24	177.1(2)
C21	C20	C19	C18	33.0(3)
C39	C14	C15	C16	-61.1(3)
C24	C42	O3	C43	178.8(2)
C24	C25	C26	C1	139.6(3)
C10	C11	C12	C13	-4.9(3)
C3	C2	C1	C26	-59.8(2)
C3	C4	C27	C28	-177.3(2)
C16	C15	C14	C13	173.6(2)
C26	C1	C33	C45	35.2(3)
C26	C1	C33	C34	-147.4(2)

Atom	Atom	Atom	Atom	Angle/°
C13	C14	C39	C46	54.4(3)
C13	C14	C39	C40	-126.8(3)
C46	C39	C14	C15	-67.0(3)
C18	C19	C20	C41	-147.4(3)
C18	C17	C16	C15	53.7(3)
C30	C17	C16	C15	-125.8(3)
C15	C14	C39	C40	111.7(3)

Table 6: Hydrogen Fractional Atomic Coordinates (×10[4]) and Equivalent Isotropic Displacement Parameters (Å[2]×10[3]) for *E,E*-**40**. U_{eq} is defined as 1/3 of the trace of the orthogonalised U_{ij}.

Atom	x	y	z	U_{eq}
H2a	2683(5)	2137(9)	1390(10)	39(4)
H2b	1989(7)	1360(20)	560(5)	40(4)
H5	4536(19)	1920(50)	1421(16)	44(8)
H31	6217(18)	13420(50)	5633(15)	36(7)
H23a	2790(4)	7140(30)	2744.4(17)	36(4)
H23b	2420(3)	9098(9)	3180(9)	28(4)
H14	7498(17)	3680(40)	4257(14)	41(4)
H22a	3541(4)	9416(11)	2040(10)	44(6)
H22b	3126(4)	11490(30)	2451(2)	47(6)
H9a	6680(3)	934(12)	2936(9)	36(4)
H9b	7026(4)	-1230(30)	2560(3)	36(4)
H32	5040(20)	13920(50)	4530(17)	63(11)
H27	2894(18)	-230(50)	-562(15)	40(7)
H38a	8712(3)	-3481(15)	1010(8)	51(5)
H38b	9258(7)	-5570(20)	1401(2)	58(5)
H38c	8274(10)	-4937(11)	1617(3)	54(5)
H29	4990(20)	-3790(50)	430(16)	43(8)
H10a	7371(4)	2950(30)	2123(2)	45(6)
H10b	7682(3)	888(11)	1732(9)	35(5)
H1	1301(18)	3830(40)	1215(14)	36(4)
H3a	2825(5)	3450(8)	-185(10)	38(5)
H3b	3532(7)	4340(20)	669(5)	45(5)
H16a	7483(10)	7324(4)	5278(9)	45(5)
H16b	6485(8)	5840(20)	4854(2)	42(5)
H35a	5196(9)	-4048(12)	1920(2)	58(5)
H35b	6003(4)	-4147(14)	1409(7)	58(5)
H35c	6229(8)	-3363(4)	2301(8)	50(5)
H26a	2748(8)	5670(10)	1766(8)	40(4)
H26b	2714(7)	6889(12)	898(7)	34(5)
H13a	8508(8)	5910(20)	3297(2)	43(5)
H13b	7520(11)	4668(4)	2980(8)	47(4)
H37a	9867(11)	-2482(12)	1970(3)	48(5)
H37b	9446(4)	-3968(17)	2579(9)	44(4)
H46a	9532(5)	6095(6)	4246(8)	62(5)
H46b	9056(4)	7780(30)	4732.1(18)	66(5)
H46c	9780(9)	6103(6)	5168(8)	52(5)
H28	3830(20)	-3110(60)	-487(18)	79(12)
H43a	803(2)	14237(18)	2299(9)	43(4)
H43b	240(12)	12692(11)	2789.5(15)	43(5)
H18	5661(19)	8350(50)	3661(15)	48(9)
H41a	4019(6)	13539(4)	2615(9)	53(5)
H41b	3993(6)	14324(13)	3527(6)	57(5)
H41c	4985(10)	14351(13)	3190.4(16)	55(5)

Atom	x	y	z	U_{eq}
H30	7068(18)	10290(50)	5702(15)	40(7)
H15a	6786(10)	6737(4)	3605(9)	44(5)
H15b	7760(8)	8150(20)	4021(2)	46(5)
H44a	1876(12)	14756(8)	3428.5(17)	66(5)
H44b	844(6)	15770(20)	3446.5(18)	62(5)
H44c	1157(2)	13473(16)	3897(8)	63(5)
H34a	629(3)	2030(30)	83(2)	54(5)
H34b	6(8)	4195(8)	365(6)	48(5)
H34c	173(5)	3981(6)	-598(9)	50(5)
H8	5834(19)	1800(50)	1675(16)	46(8)
H21	4290(20)	8300(50)	3343(18)	53(9)
H12	9168(17)	2530(40)	3679(14)	36(7)
H25	1164(18)	8100(50)	1085(15)	48(9)
H45a	1086(19)	6280(50)	-844(16)	45(8)
H40a	9154(19)	3700(60)	5755(17)	58(10)
H45b	1910(20)	7410(60)	-163(17)	80(12)
H40b	8160(30)	2710(90)	5340(30)	163(19)

References for Supplementary Information

1. Liang, Y. F. *et al.* Total Syntheses of (+)-α-Allokainic Acid and (-)-2- epi-α-Allokainic Acid Employing Ketopinic Amide as a Chiral Auxiliary. *Journal of Organic Chemistry* **83**, 10564–10572 (2018).

2. McCormick, J. P. & Barton, D. L. Synthetic applications of metal halides. Conversion of cyclopropylmethanols into homoallylic halides. *Journal of Organic Chemistry* **45**, 2566–2570 (2002).

3. Huo, S. Highly Efficient, General Procedure for the Preparation of Alkylzinc Reagents from Unactivated Alkyl Bromides and Chlorides. *Org. Lett.* **5**, 423–425 (2003).

4. Sheddan, N. A. & Mulzer, J. Access to isocarbacyclin derivatives via substrate-controlled enolate formation: Total synthesis of 15-deoxy-16-(m-tolyl)-17,18,19,20- tetranorisocarbacyclin. *Org. Lett.* **7**, 5115–5118 (2005).

5. Wang, Z. X., Tu, Y., Frohn, M., Zhang, J. R. & Shi, Y. An Efficient Catalytic Asymmetric Epoxidation Method. *J. Am. Chem. Soc.* **119**, 11224–11235 (1997).

6. CrysAlis^{Pro} (Rigaku Oxford Diffraction), Rigaku Oxford Diffraction, Poland .

7. Bourhis, L. J., Dolomanov, O. V., Gildea, R. J., Howard, J. A. K. & Puschmann, H. The anatomy of a comprehensive constrained, restrained refinement program for the modern computing environment – Olex2 dissected. *Acta. Crystallogr. A Found. Adv.* **71**, 59–75 (2015).

8. Dolomanov, O. V., Bourhis, L. J., Gildea, R. J., Howard, J. A. K. & Puschmann, H. OLEX2: a complete structure solution, refinement and analysis program. *J. Appl. Cryst.* **42**, 339–341 (2009).

NMR spectra of compounds

See next page

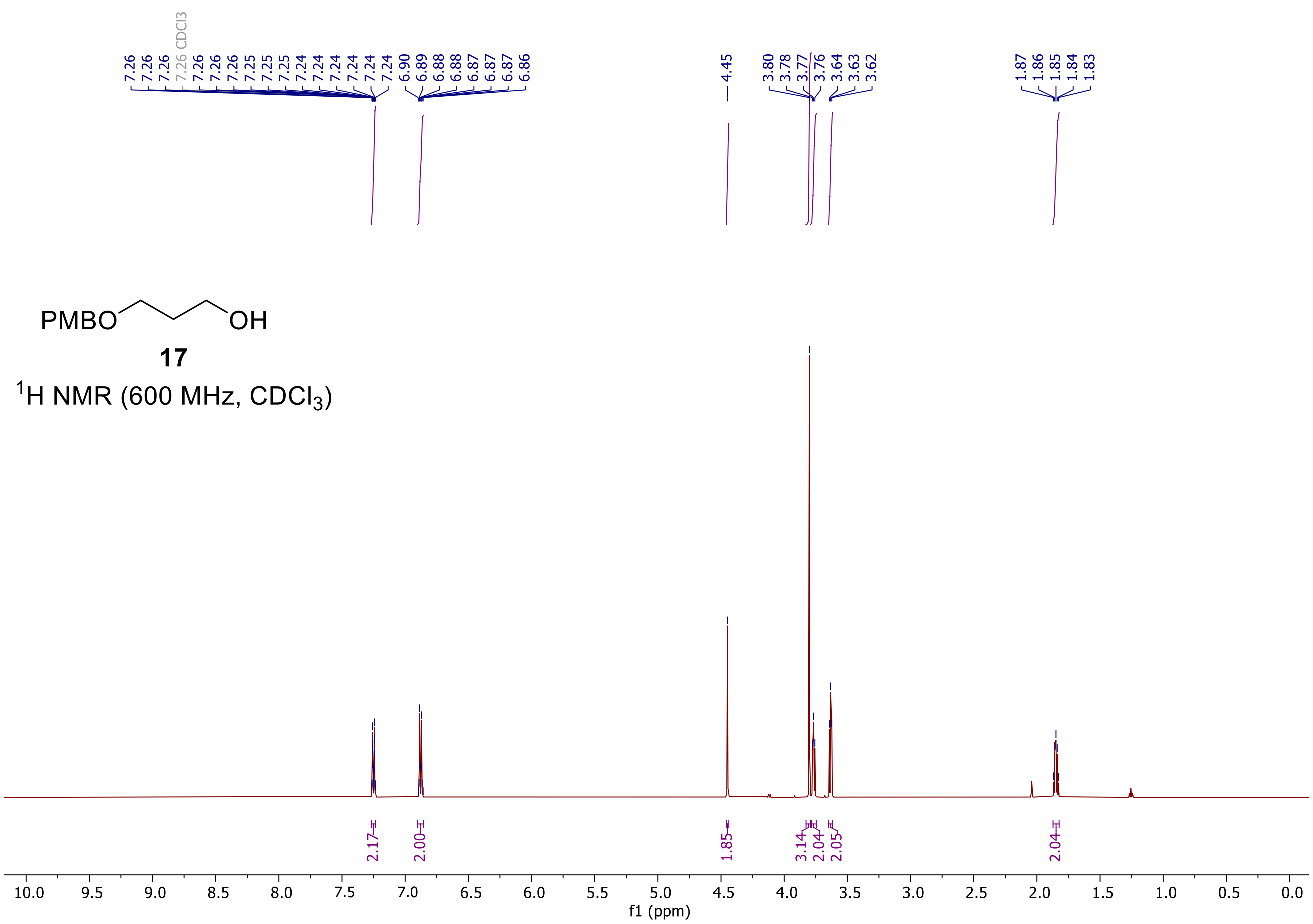

7.26
7.26
7.26
7.26 CDCl3
7.26
7.26
7.26
7.25
7.25
7.24
7.24
7.24
7.24
7.24
7.24
6.90
6.89
6.88
6.88
6.87
6.87
6.87
6.86
4.45
3.80
3.78
3.77
3.76
3.64
3.63
3.62
1.87
1.86
1.85
1.84
1.83
PMBO OH
17
1H NMR (600 MHz, CDCl3)
2.17
2.00
1.85
3.14
2.04
2.05
2.04
10.0 9.5 9.0 8.5 8.0 7.5 7.0 6.5 6.0 5.5 5.0 4.5 4.0 3.5 3.0 2.5 2.0 1.5 1.0 0.5 0.0
f1 (ppm)

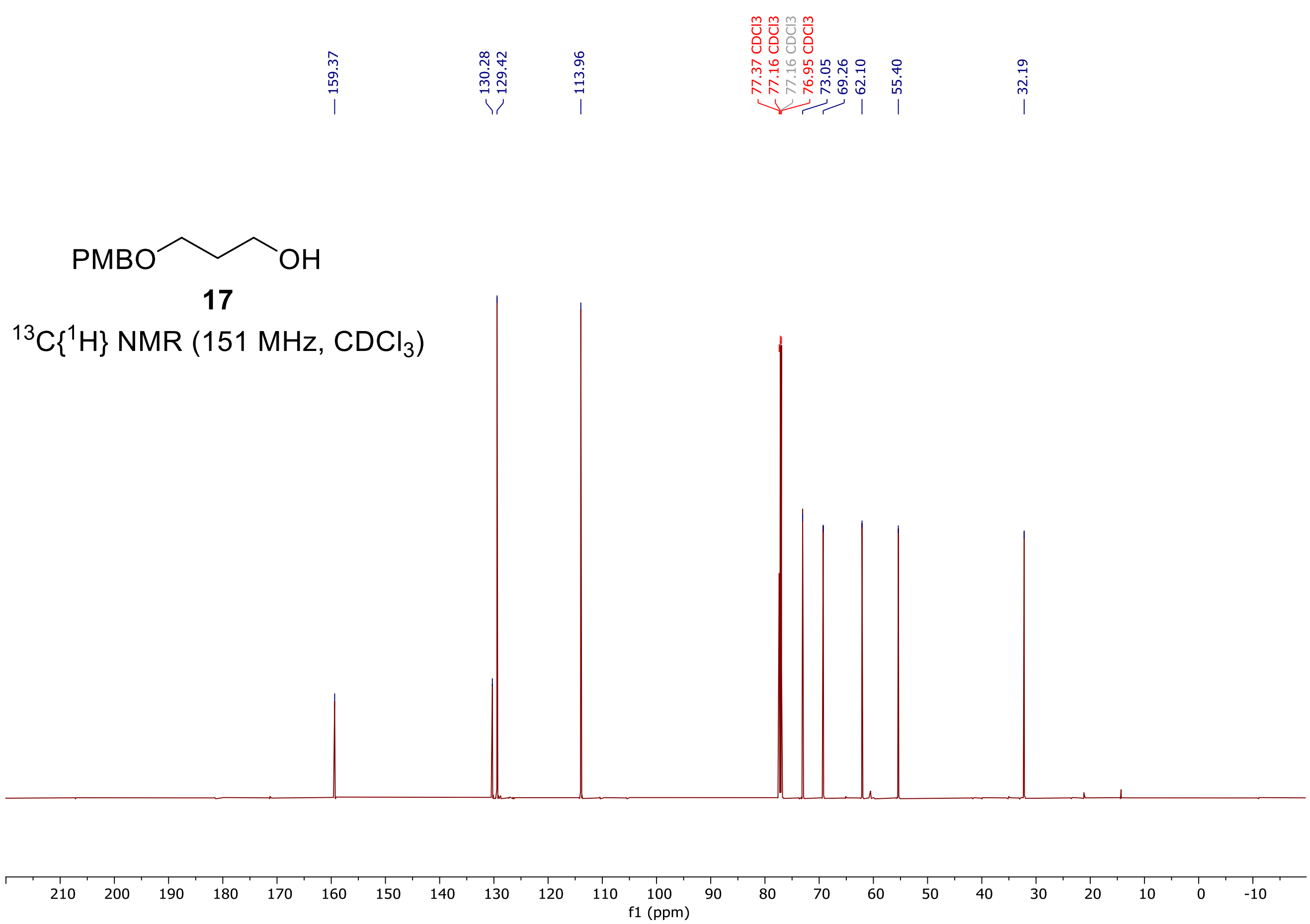

159.37
130.28
129.42
113.96
77.37 CDCl3
77.16 CDCl3
77.16 CDCl3
76.95 CDCl3
73.05
69.26
62.10
55.40
32.19
PMBO OH
17
13C{1H} NMR (151 MHz, CDCl3)
210 200 190 180 170 160 150 140 130 120 110 100 90 80 70 60 50 40 30 20 10 0 -10
f1 (ppm)

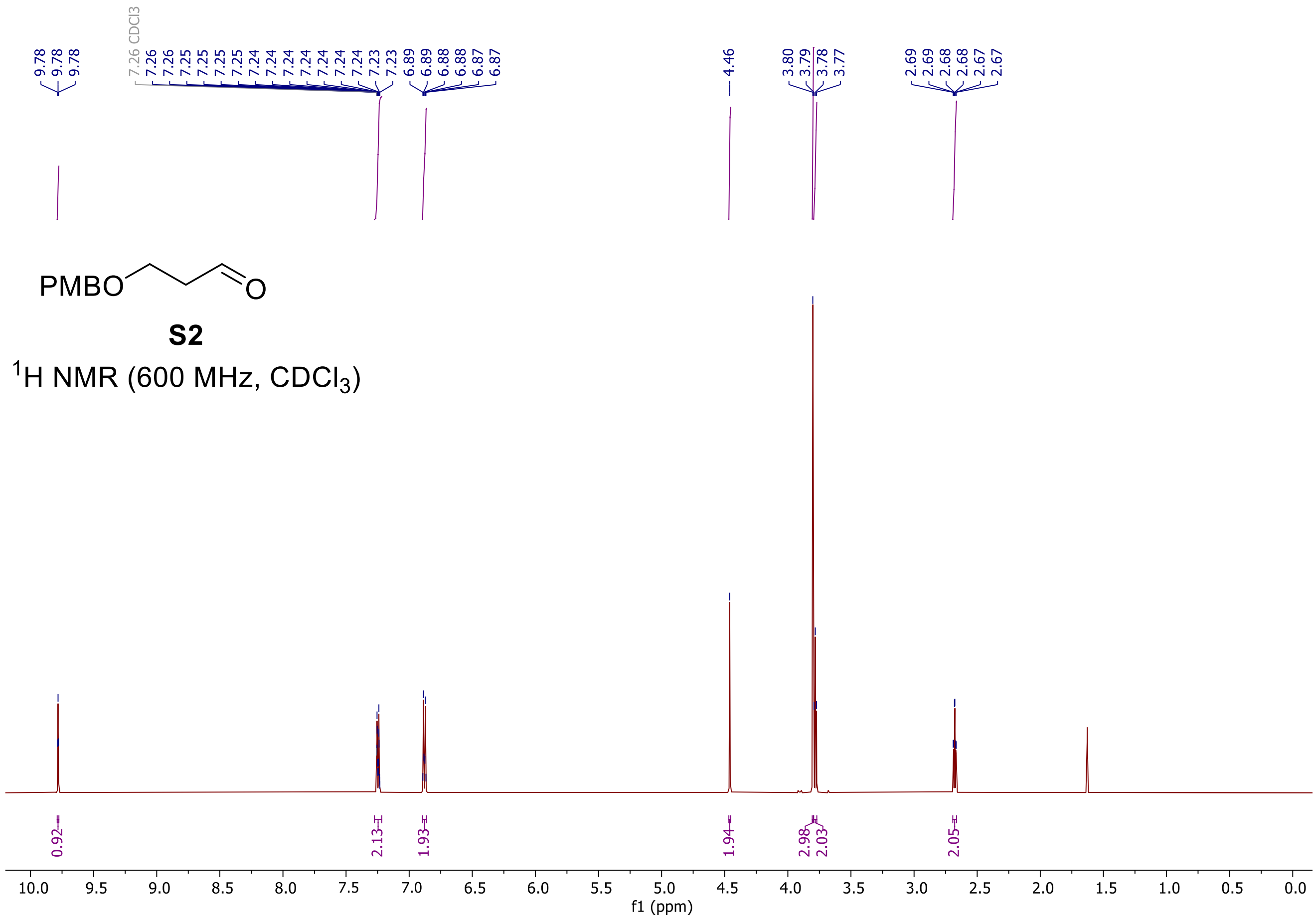
9.78
9.78
9.78
7.26 CDCl3
7.26
7.26
7.25
7.25
7.25
7.25
7.24
7.24
7.24
7.24
7.24
7.24
7.24
7.23
7.23
6.89
6.89
6.88
6.88
6.87
6.87
4.46
3.80
3.79
3.78
3.77
2.69
2.69
2.68
2.68
2.67
2.67
PMBO
O
S2
1H NMR (600 MHz, CDCl3)
0.92
2.13
1.93
1.94
2.98
2.03
2.05
f1 (ppm)

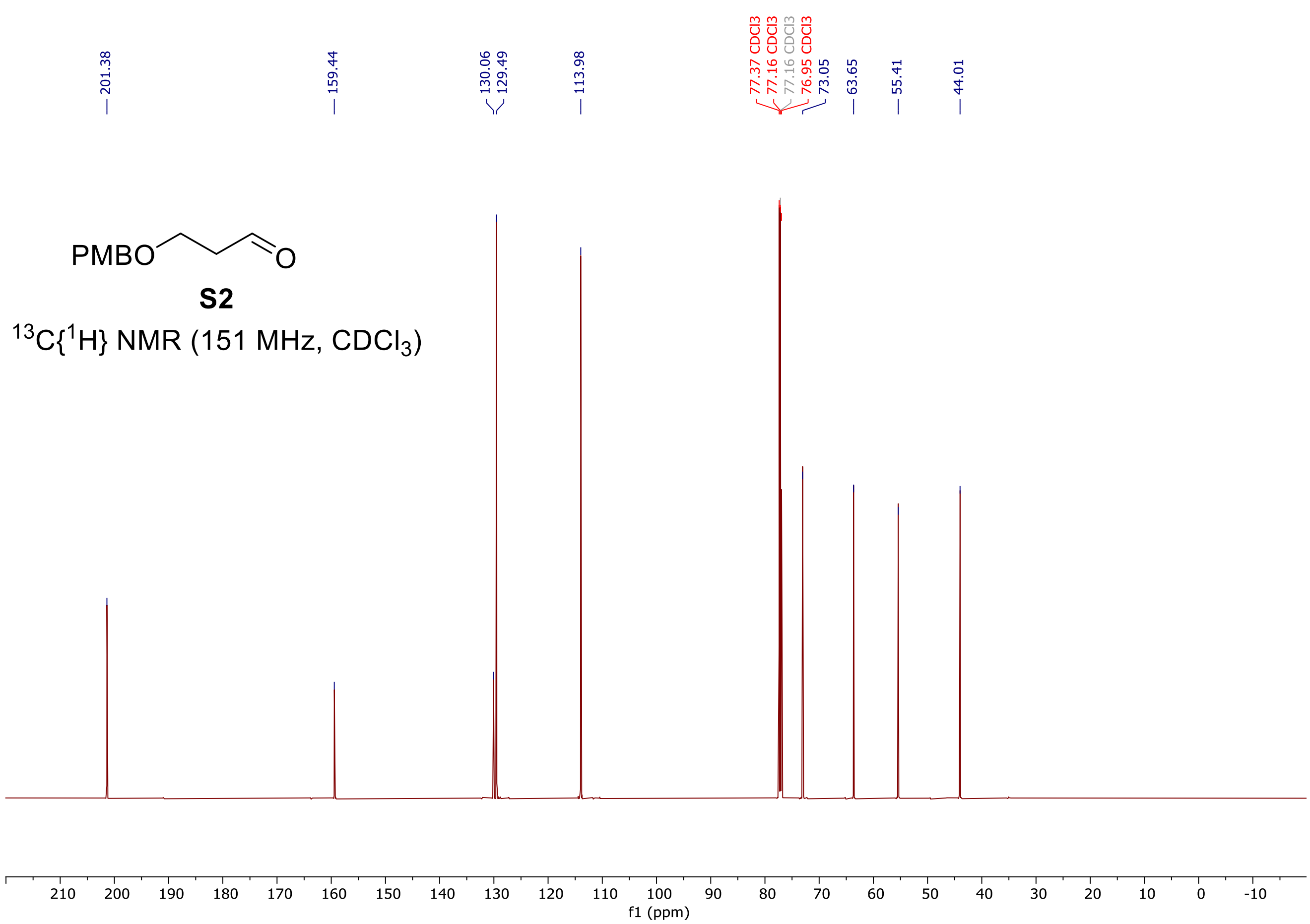
201.38
159.44
130.06
129.49
113.98
77.37 CDCl3
77.16 CDCl3
77.16 CDCl3
76.95 CDCl3
73.05
63.65
55.41
44.01
PMBO
O
S2
13C{1H} NMR (151 MHz, CDCl3)
f1 (ppm)

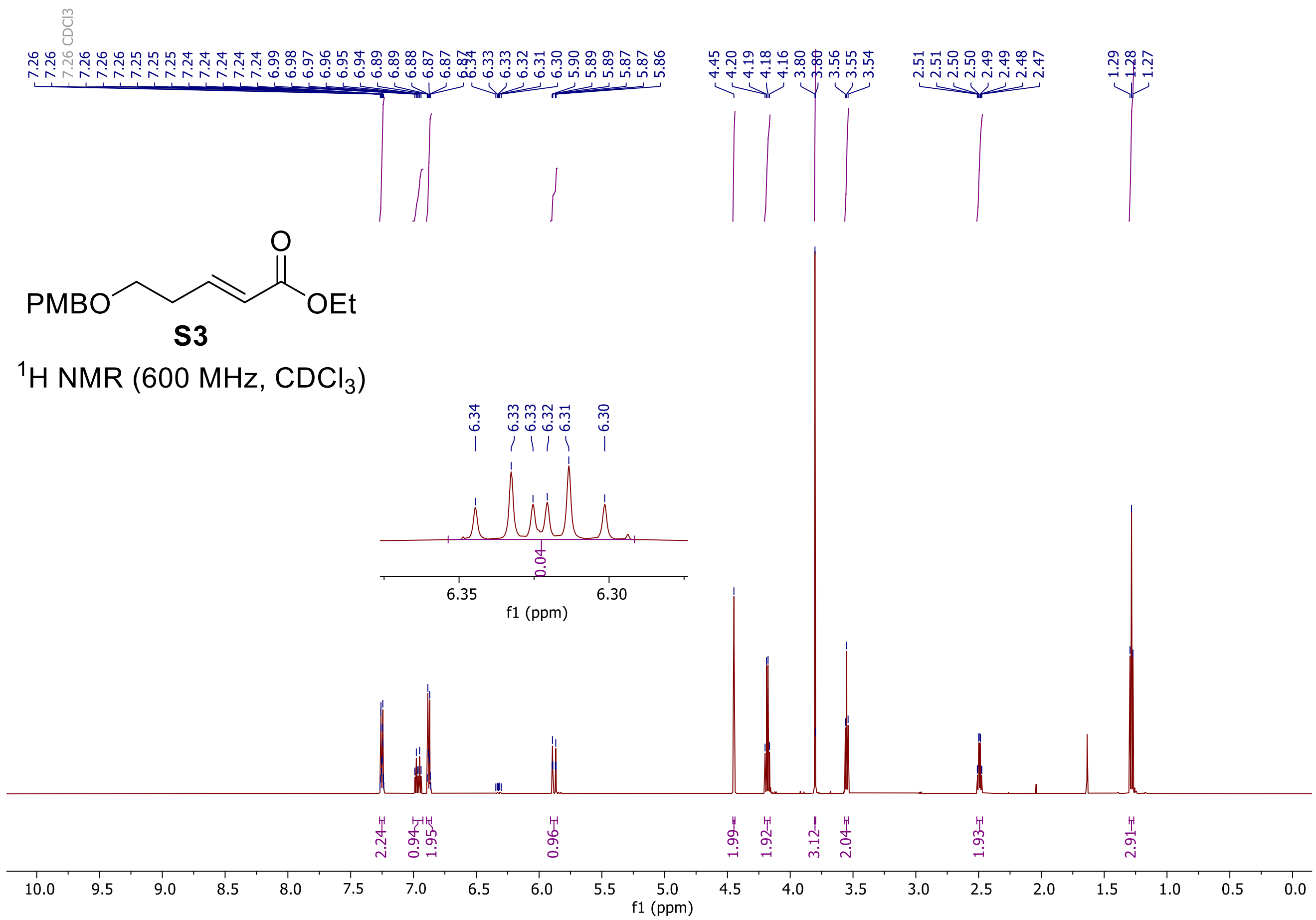

PMBO OEt
O
S3
1H NMR (600 MHz, CDCl3)
7.26 7.26 7.26 CDCl3 7.26 7.26 7.25 7.25 7.25 7.24 7.24 7.24 7.24 7.24 6.99 6.98 6.97 6.96 6.95 6.94 6.89 6.89 6.88 6.87 6.87 6.87 6.34 6.33 6.33 6.32 6.31 6.30 5.90 5.89 5.89 5.87 5.87 5.86
4.45 4.20 4.19 4.18 4.16 3.80 3.80 3.56 3.55 3.54
2.51 2.51 2.50 2.50 2.49 2.49 2.48 2.47
1.29 1.28 1.27
6.34 6.33 6.33 6.32 6.31 6.30
0.04
6.35 6.30
f1 (ppm)
2.24 0.94 1.95 0.96 1.99 1.92 3.12 2.04 1.93 2.91
10.0 9.5 9.0 8.5 8.0 7.5 7.0 6.5 6.0 5.5 5.0 4.5 4.0 3.5 3.0 2.5 2.0 1.5 1.0 0.5 0.0
f1 (ppm)

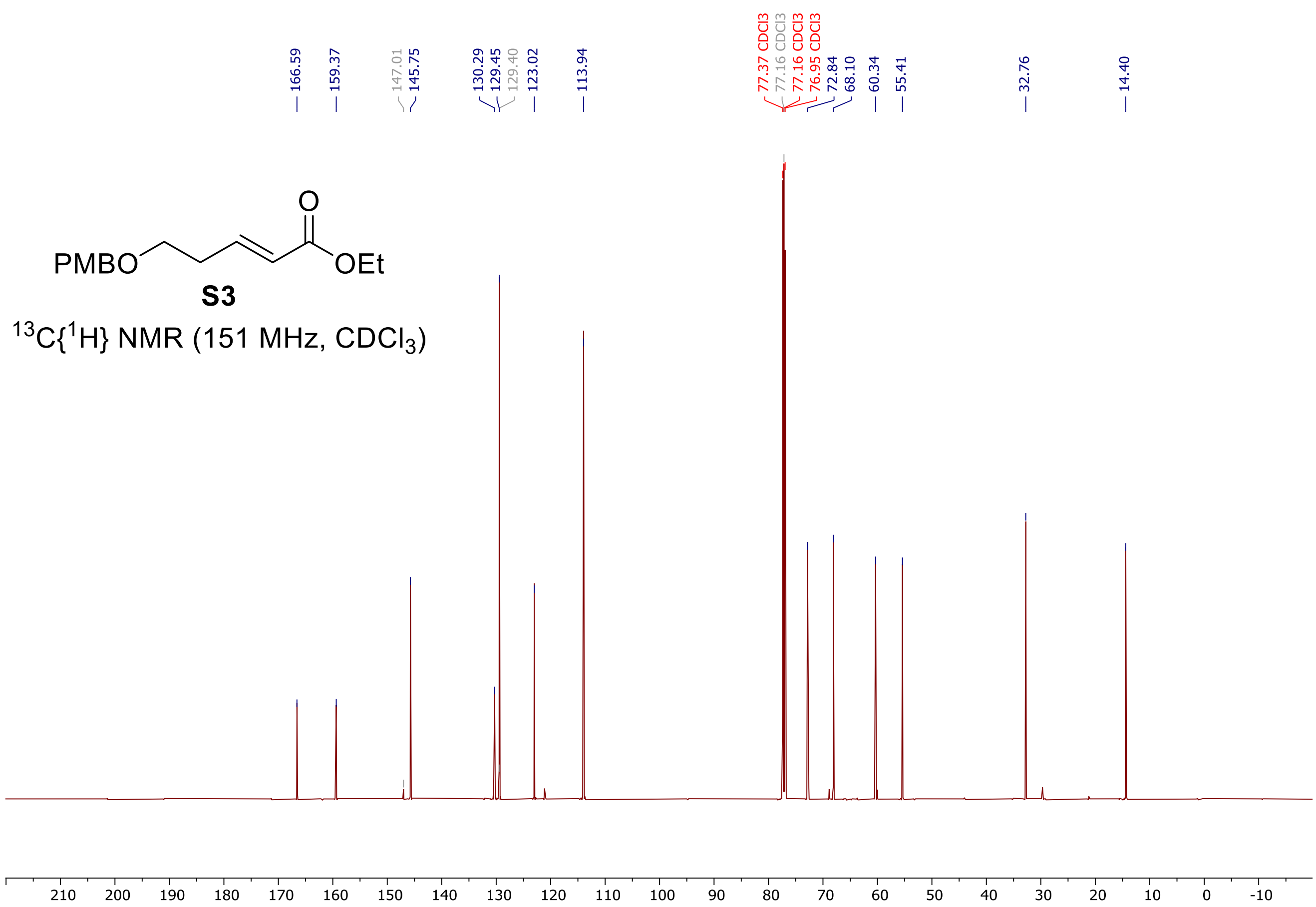

PMBO OEt
O
S3
13C{1H} NMR (151 MHz, CDCl3)
166.59 159.37 147.01 145.75 130.29 129.45 129.40 123.02 113.94
77.37 CDCl3 77.16 CDCl3 77.16 CDCl3 76.95 CDCl3 72.84 68.10 60.34 55.41 32.76 14.40
210 200 190 180 170 160 150 140 130 120 110 100 90 80 70 60 50 40 30 20 10 0 -10
f1 (ppm)

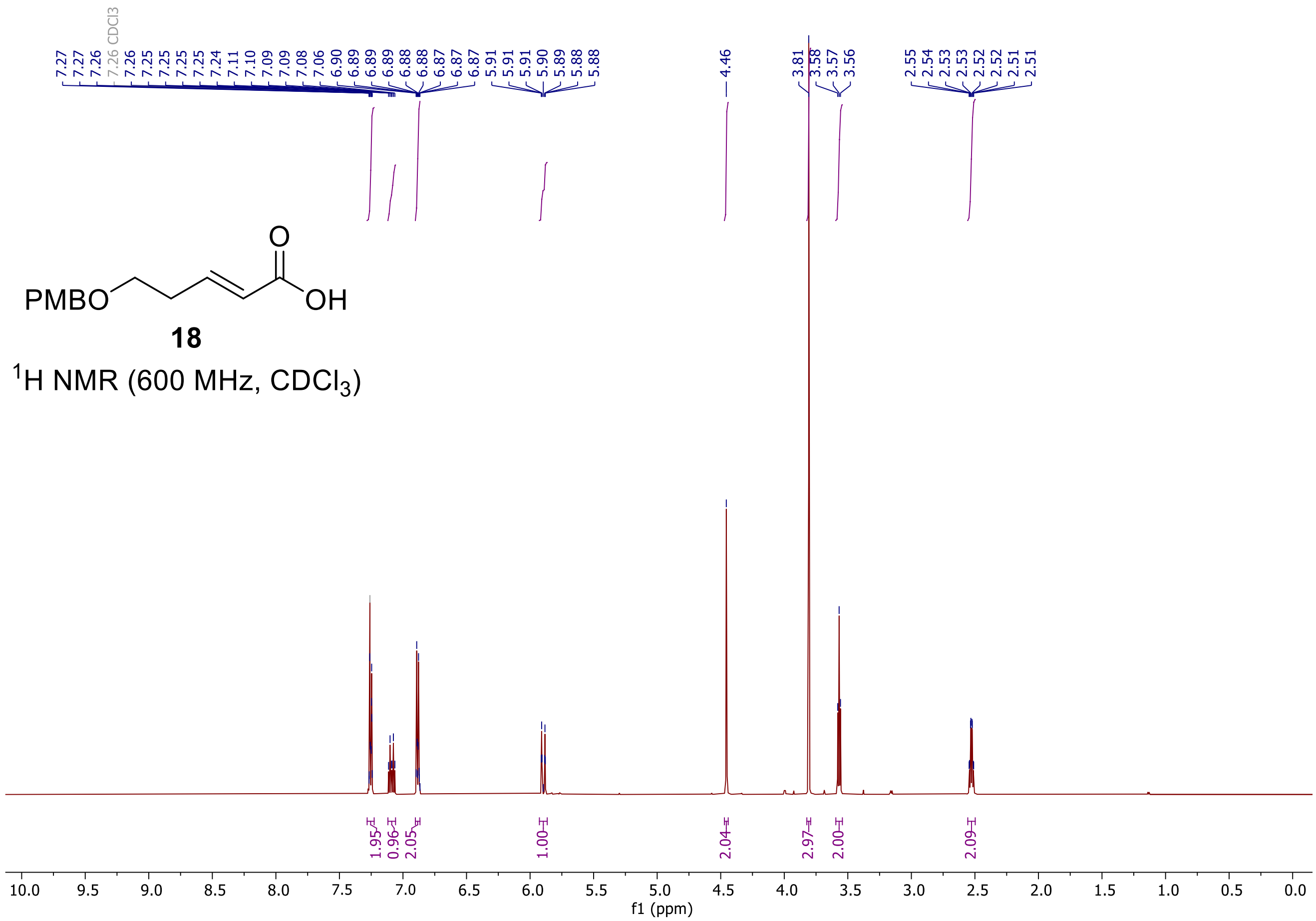

PMBO
OH
O
18
1H NMR (600 MHz, CDCl3)
7.27 7.27 7.26 7.26 CDCl3 7.26 7.25 7.25 7.25 7.25 7.24 7.11 7.10 7.09 7.09 7.08 7.06 6.90 6.89 6.89 6.89 6.88 6.88 6.87 6.87 6.87 5.91 5.91 5.91 5.90 5.89 5.88 5.88
4.46
3.81 3.58 3.57 3.56
2.55 2.54 2.53 2.53 2.52 2.52 2.51 2.51
1.95 0.96 2.05 1.00 2.04 2.97 2.00 2.09
f1 (ppm)

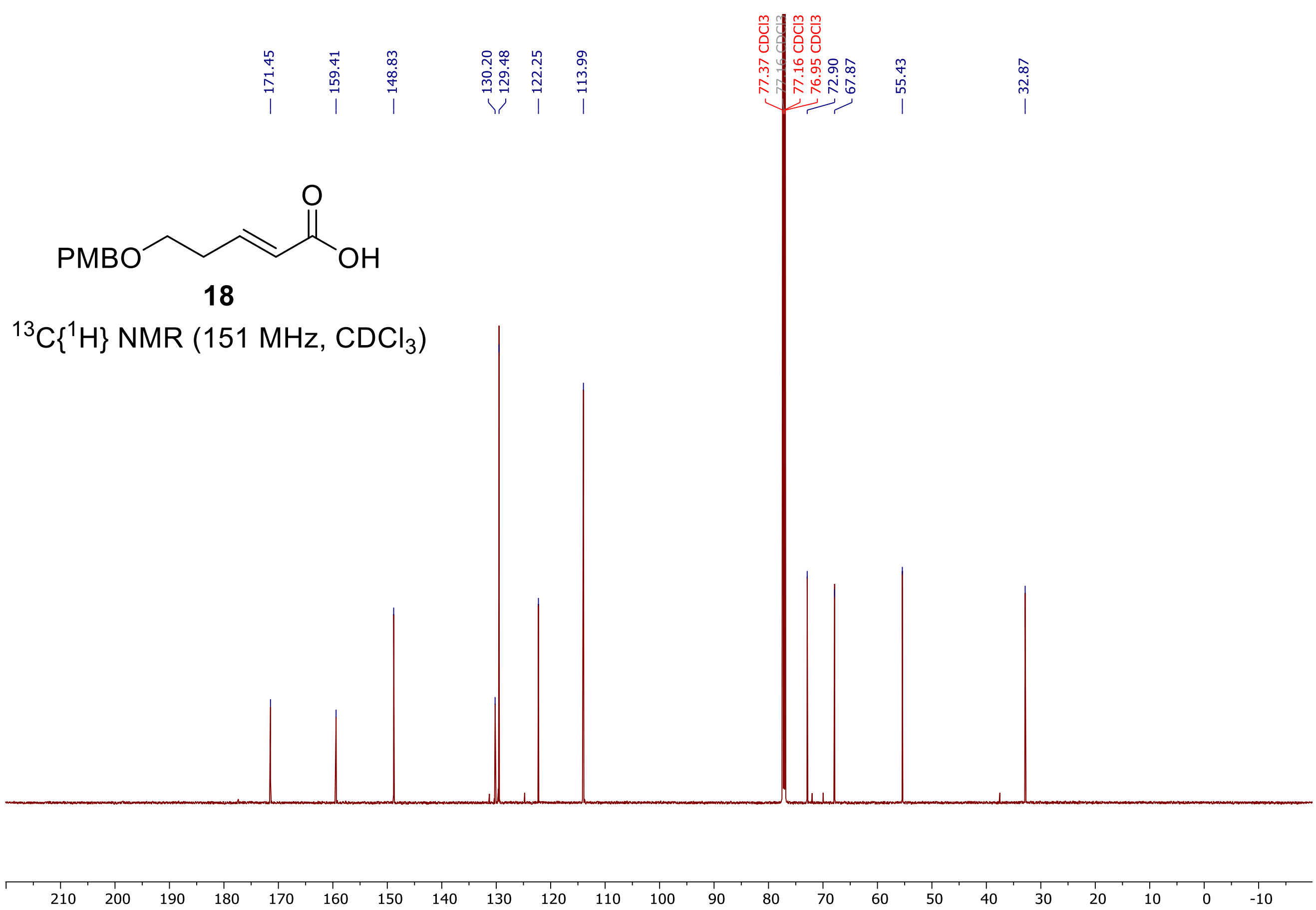

PMBO
OH
O
18
13C{1H} NMR (151 MHz, CDCl3)
171.45
159.41
148.83
130.20 129.48
122.25
113.99
77.37 CDCl3 77.16 CDCl3 77.16 CDCl3 76.95 CDCl3
72.90 67.87
55.43
32.87
f1 (ppm)

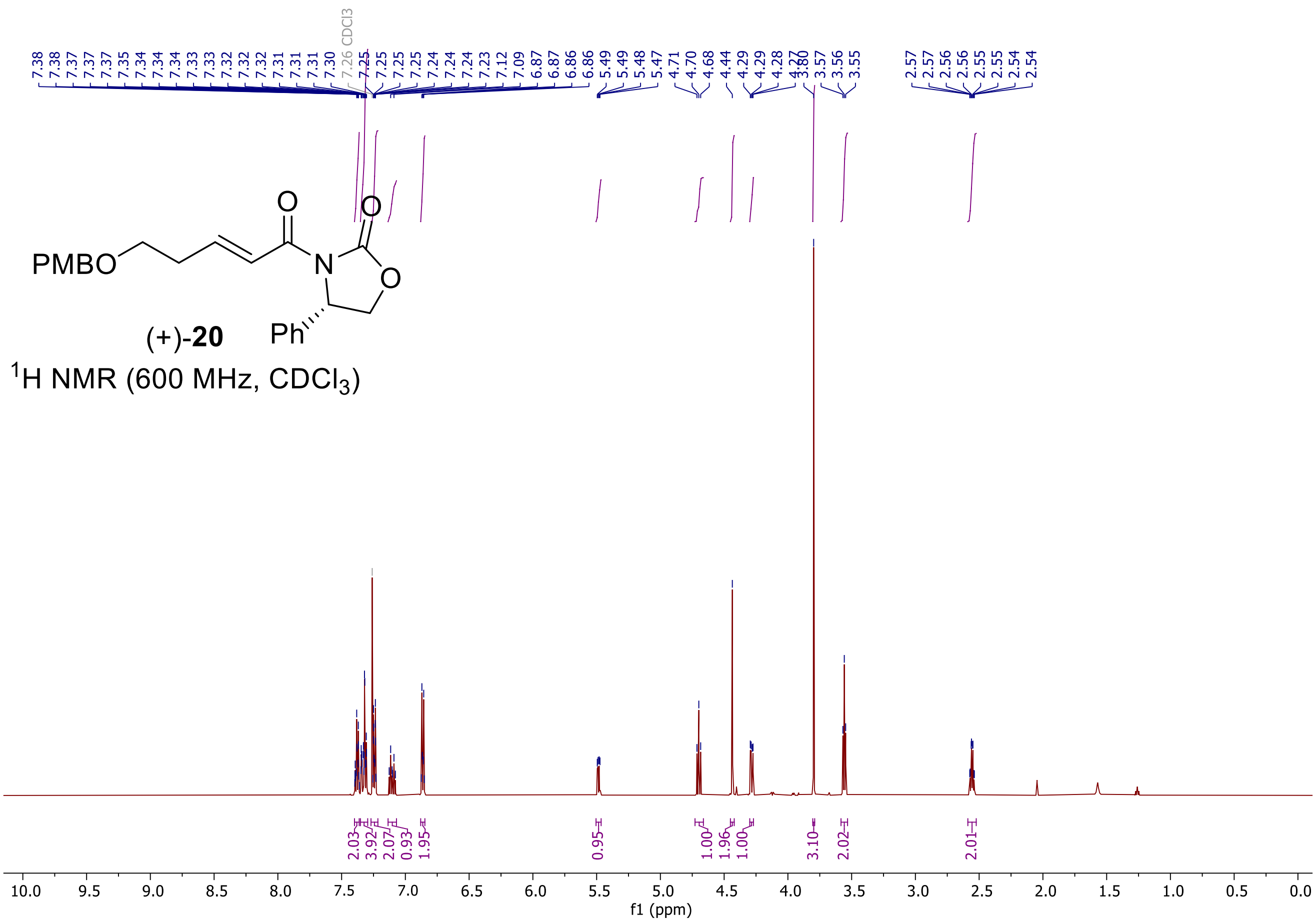
PMBO
N
O
O
O
Ph
(+)-20
^{1}H NMR (600 MHz, $CDCl_3$)
f1 (ppm)

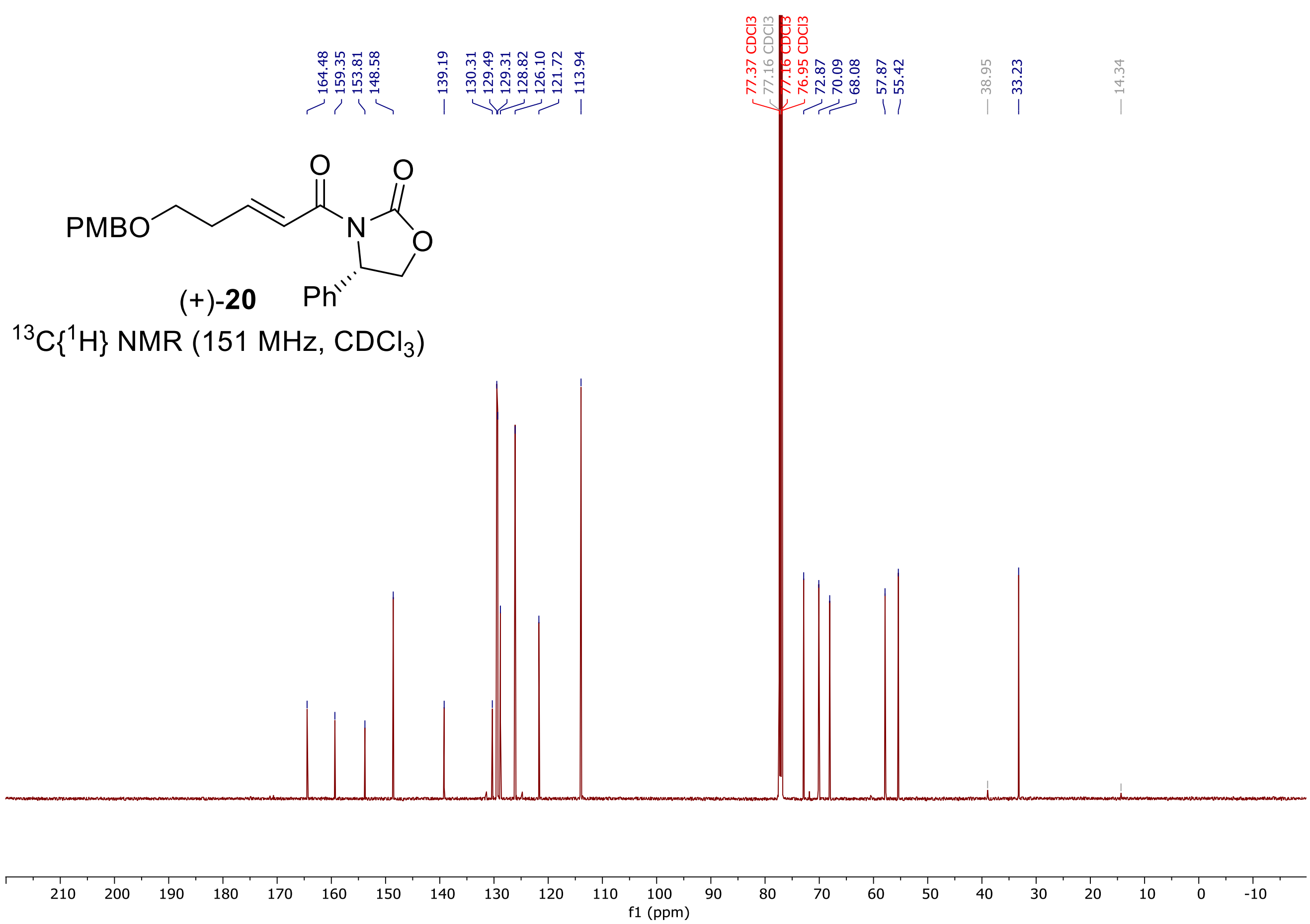
PMBO
N
O
O
O
Ph
(+)-20
^{13}C{^{1}H} NMR (151 MHz, $CDCl_3$)
f1 (ppm)

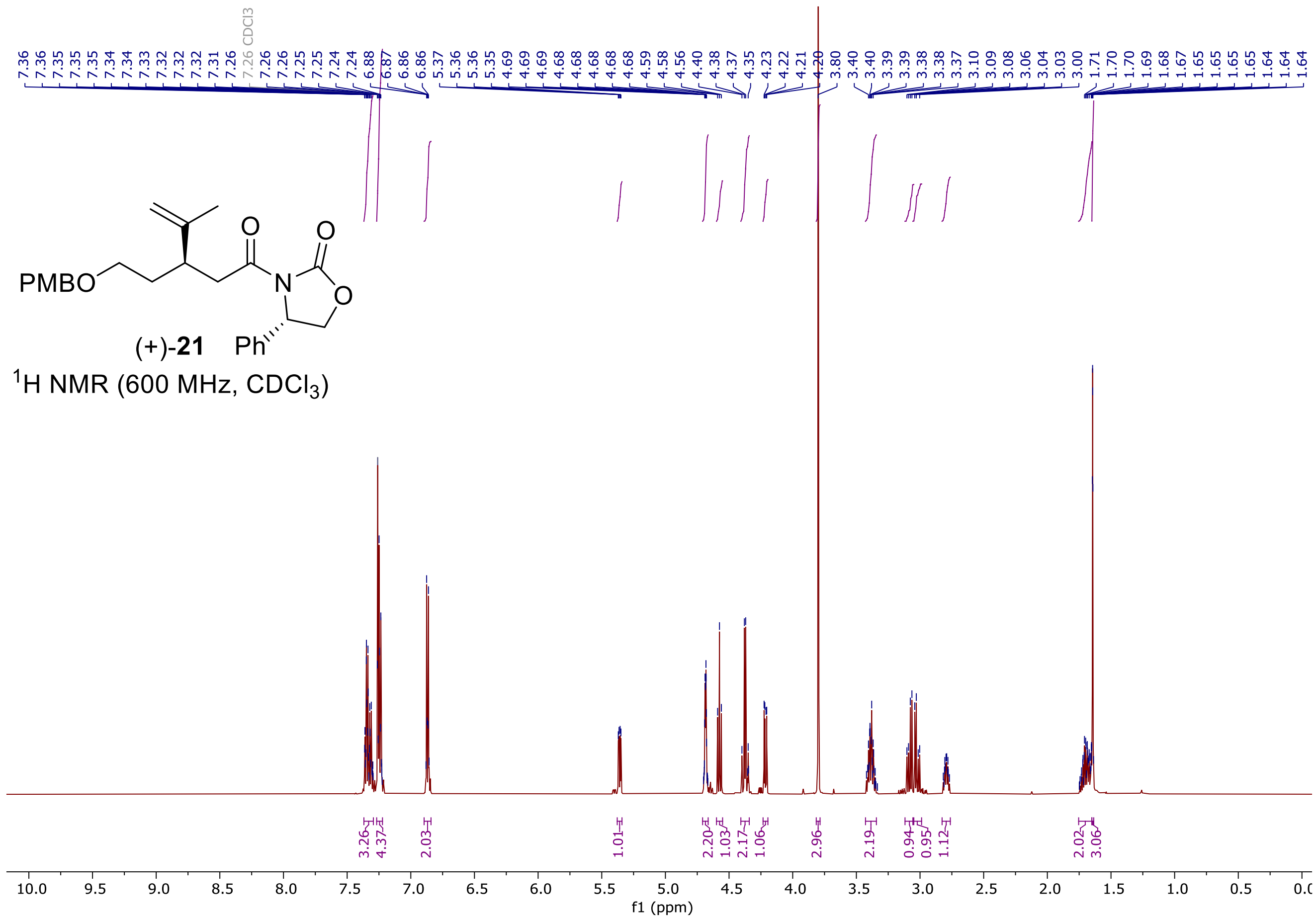

(+)-**21**

^{1}H NMR (600 MHz, $CDCl_3$)

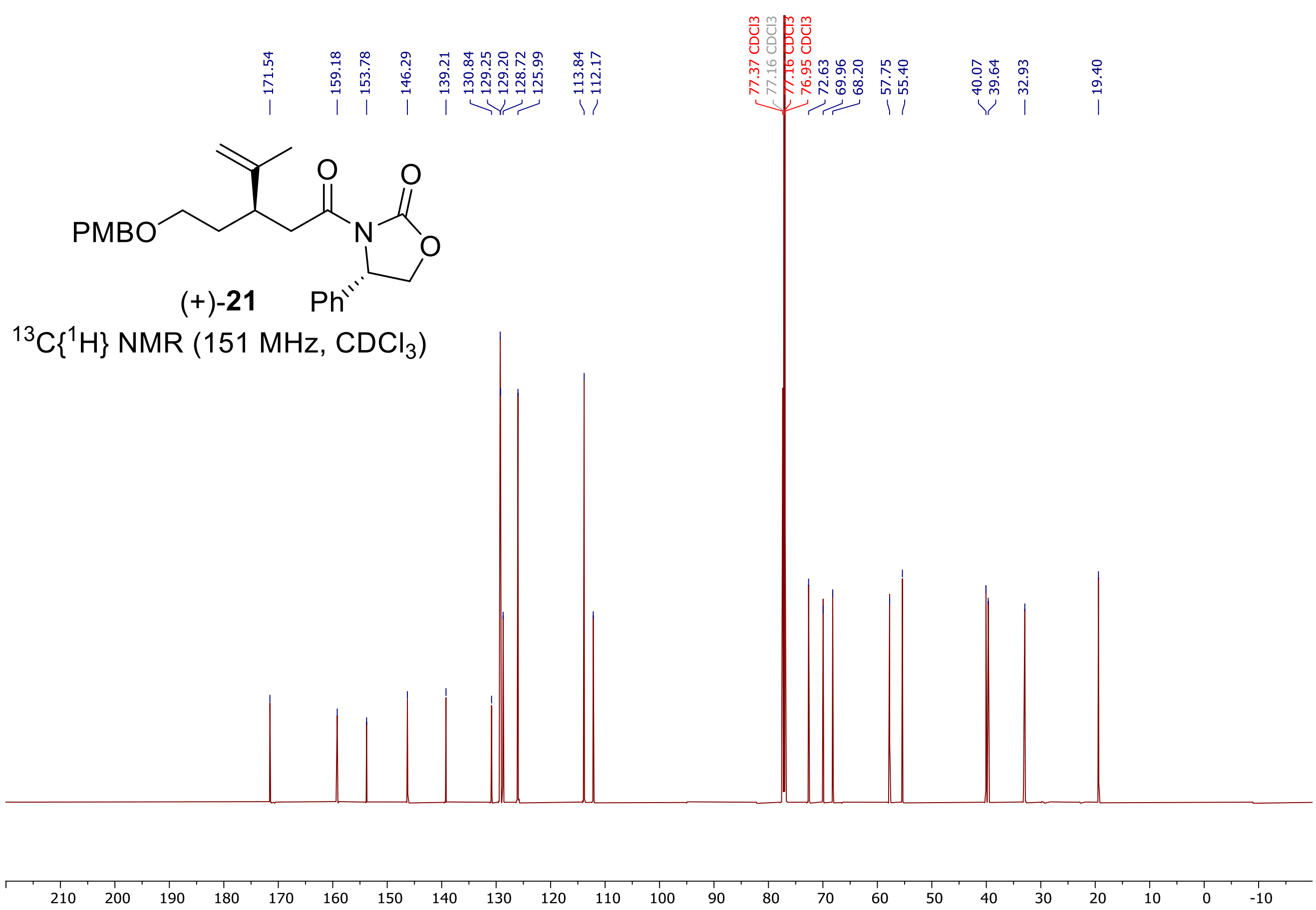

(+)-**21**

$^{13}C\{^{1}H\}$ NMR (151 MHz, $CDCl_3$)

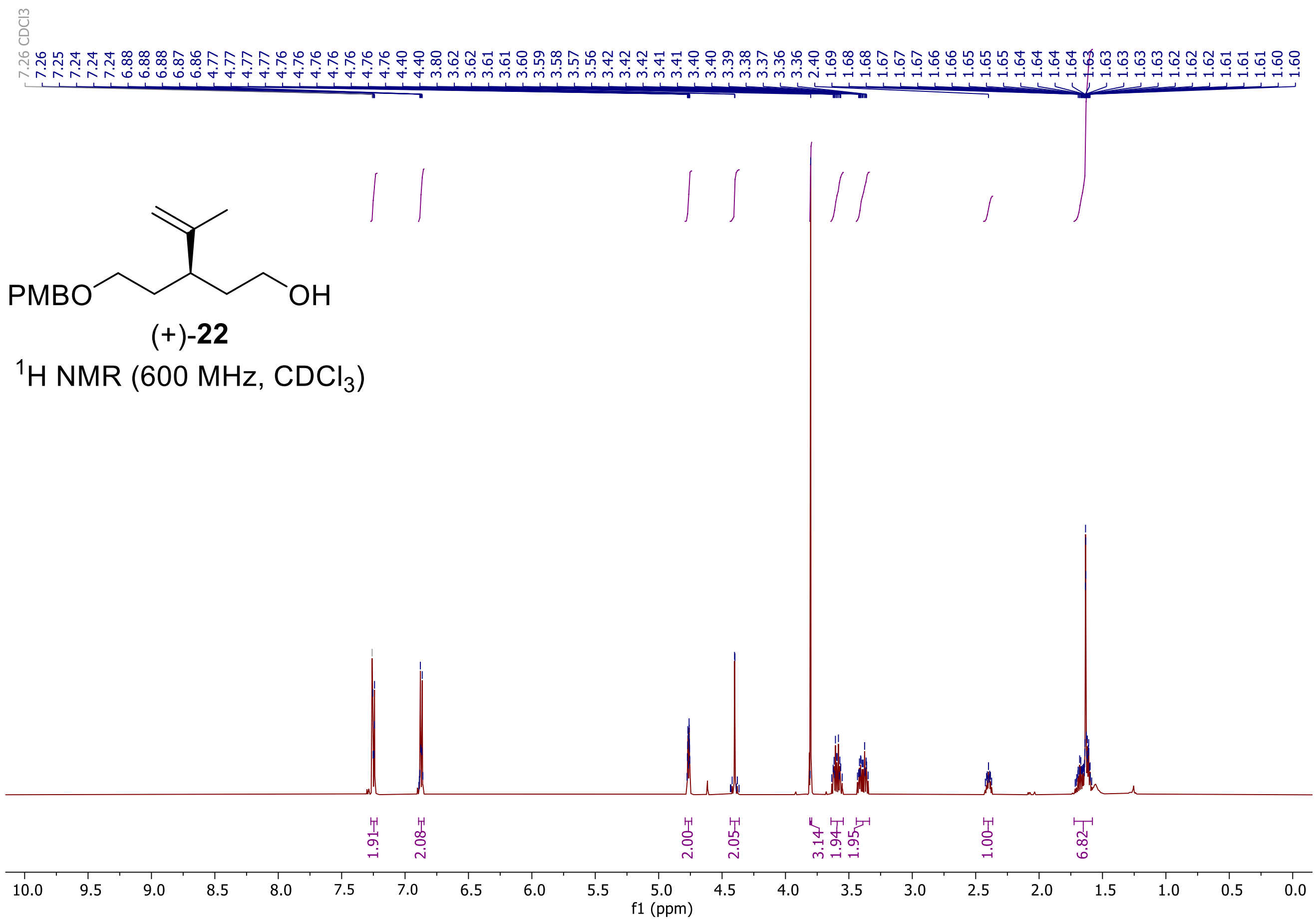

(+)-**22**

^{1}H NMR (600 MHz, $CDCl_3$)

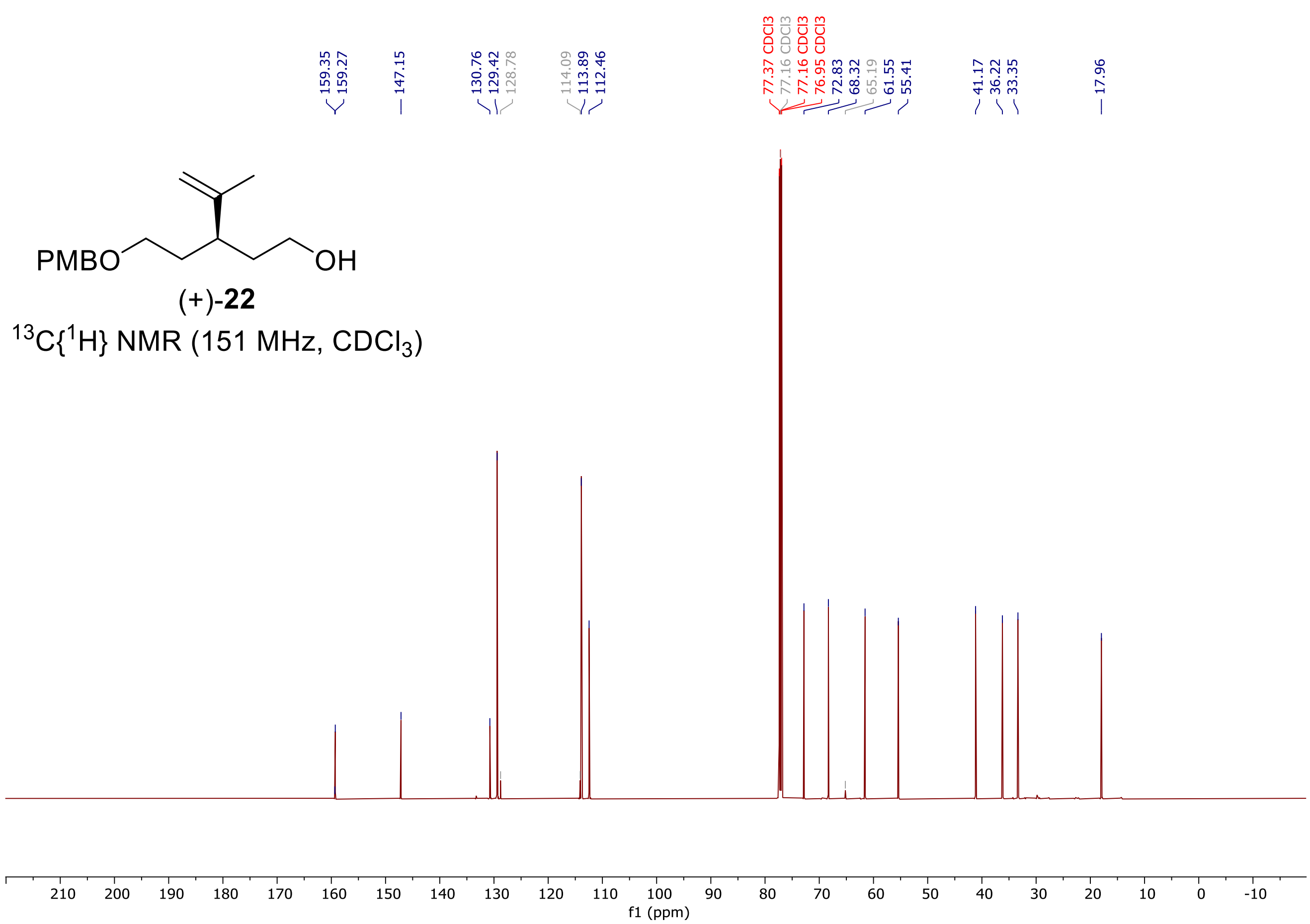

(+)-**22**

$^{13}C\{^{1}H\}$ NMR (151 MHz, $CDCl_3$)

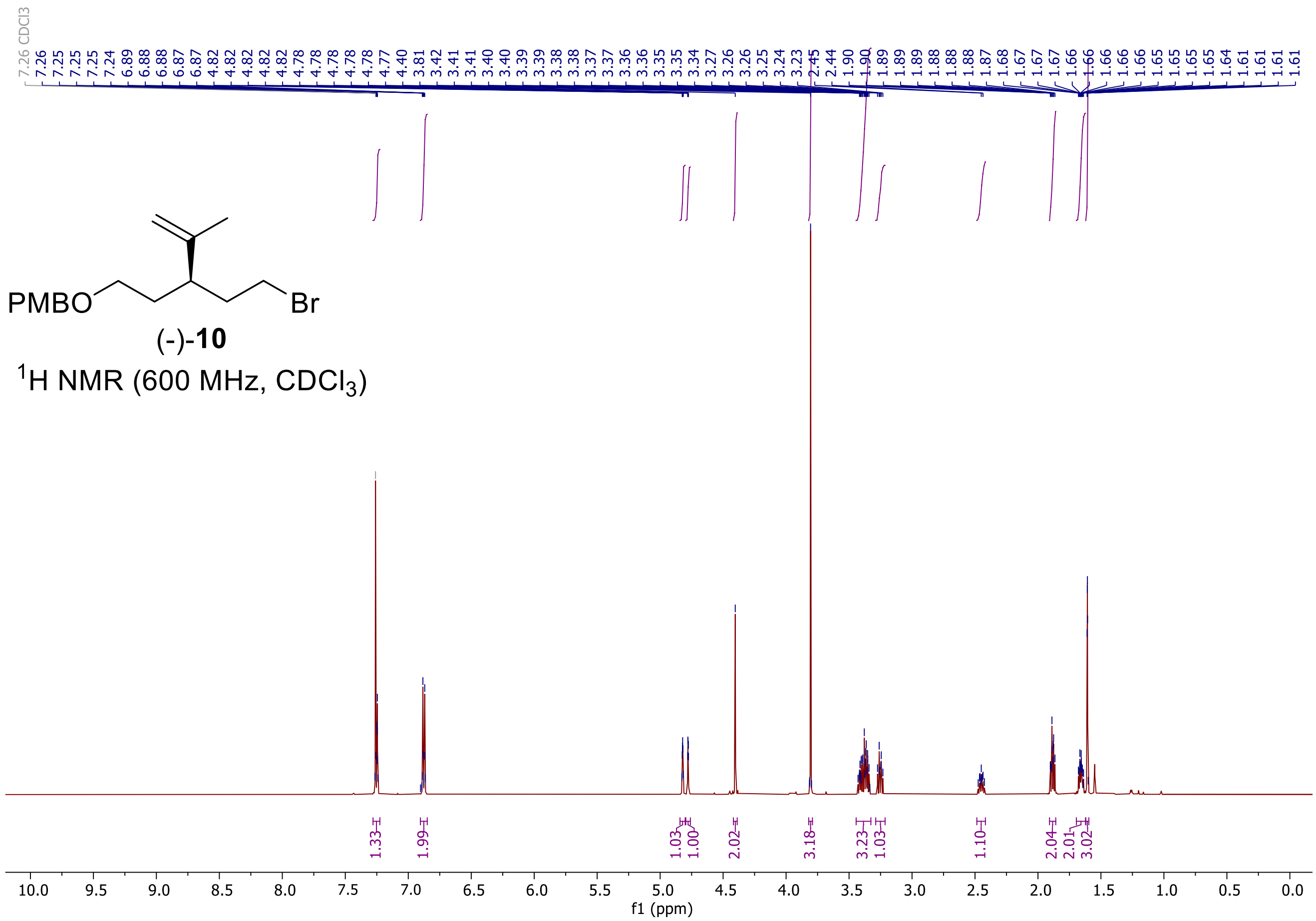
7.26 CDCl3
7.26 7.25 7.25 7.25 7.24 6.89 6.88 6.88 6.87 6.87 4.82 4.82 4.82 4.82 4.82 4.78 4.78 4.78 4.78 4.78 4.77 4.40 3.81 3.42 3.41 3.41 3.40 3.40 3.39 3.39 3.38 3.38 3.37 3.37 3.36 3.36 3.35 3.35 3.34 3.27 3.26 3.26 3.25 3.24 3.23 2.45 2.44 1.90 1.90 1.89 1.89 1.89 1.88 1.88 1.88 1.87 1.68 1.67 1.67 1.67 1.66 1.66 1.66 1.66 1.66 1.65 1.65 1.65 1.65 1.64 1.61 1.61 1.61 1.61
PMBO
Br
(-)-10
^{1}H NMR (600 MHz, $CDCl_3$)
1.33 1.99 1.03 1.00 2.02 3.18 3.23 1.03 1.10 2.04 2.01 3.02
10.0 9.5 9.0 8.5 8.0 7.5 7.0 6.5 6.0 5.5 5.0 4.5 4.0 3.5 3.0 2.5 2.0 1.5 1.0 0.5 0.0
f1 (ppm)

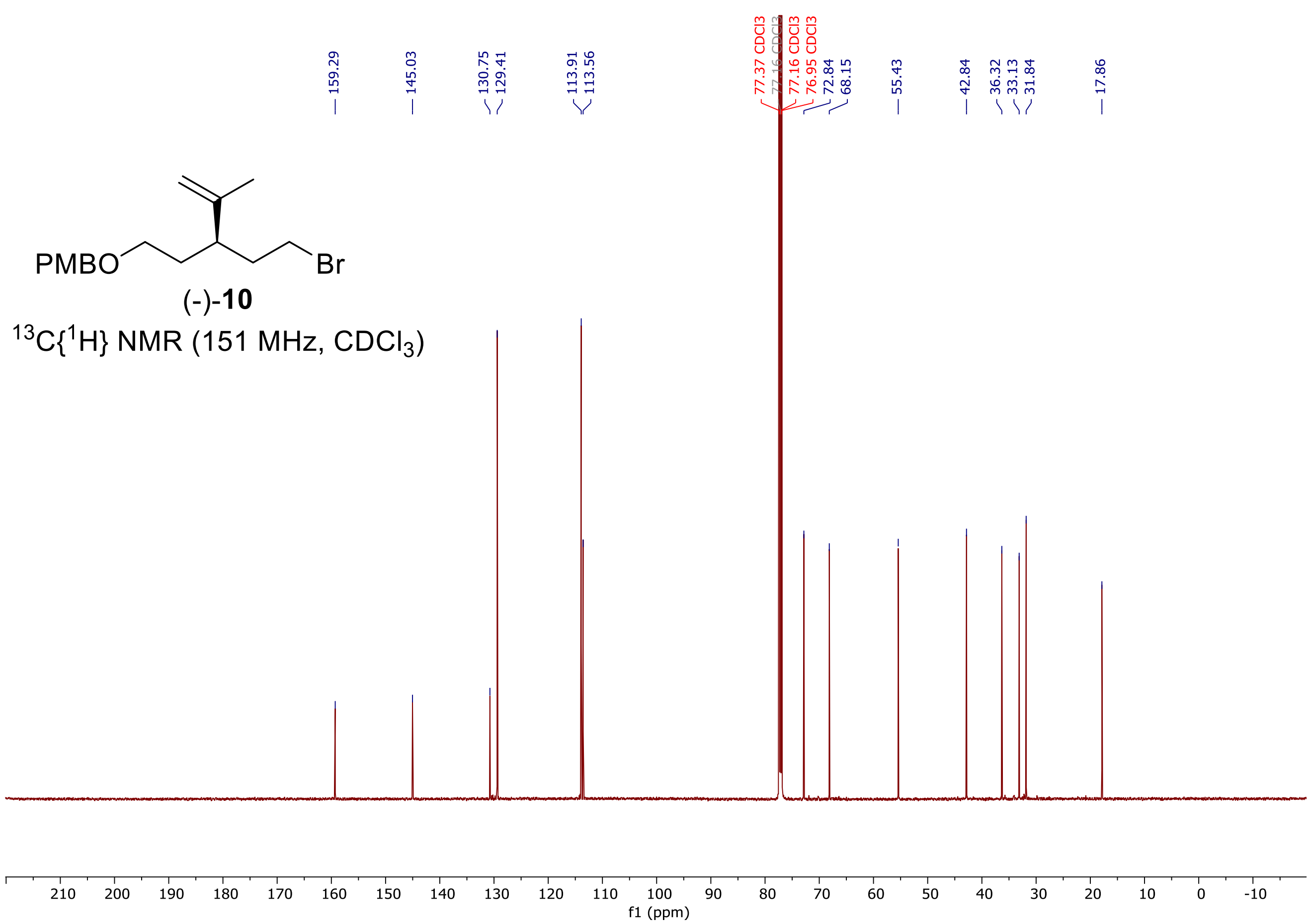
159.29
145.03
130.75 129.41
113.91 113.56
77.37 CDCl3
77.16 CDCl3
77.16 CDCl3
76.95 CDCl3
72.84
68.15
55.43
42.84
36.32
33.13
31.84
17.86
PMBO
Br
(-)-10
$^{13}C\{^{1}H\}$ NMR (151 MHz, $CDCl_3$)
210 200 190 180 170 160 150 140 130 120 110 100 90 80 70 60 50 40 30 20 10 0 -10
f1 (ppm)

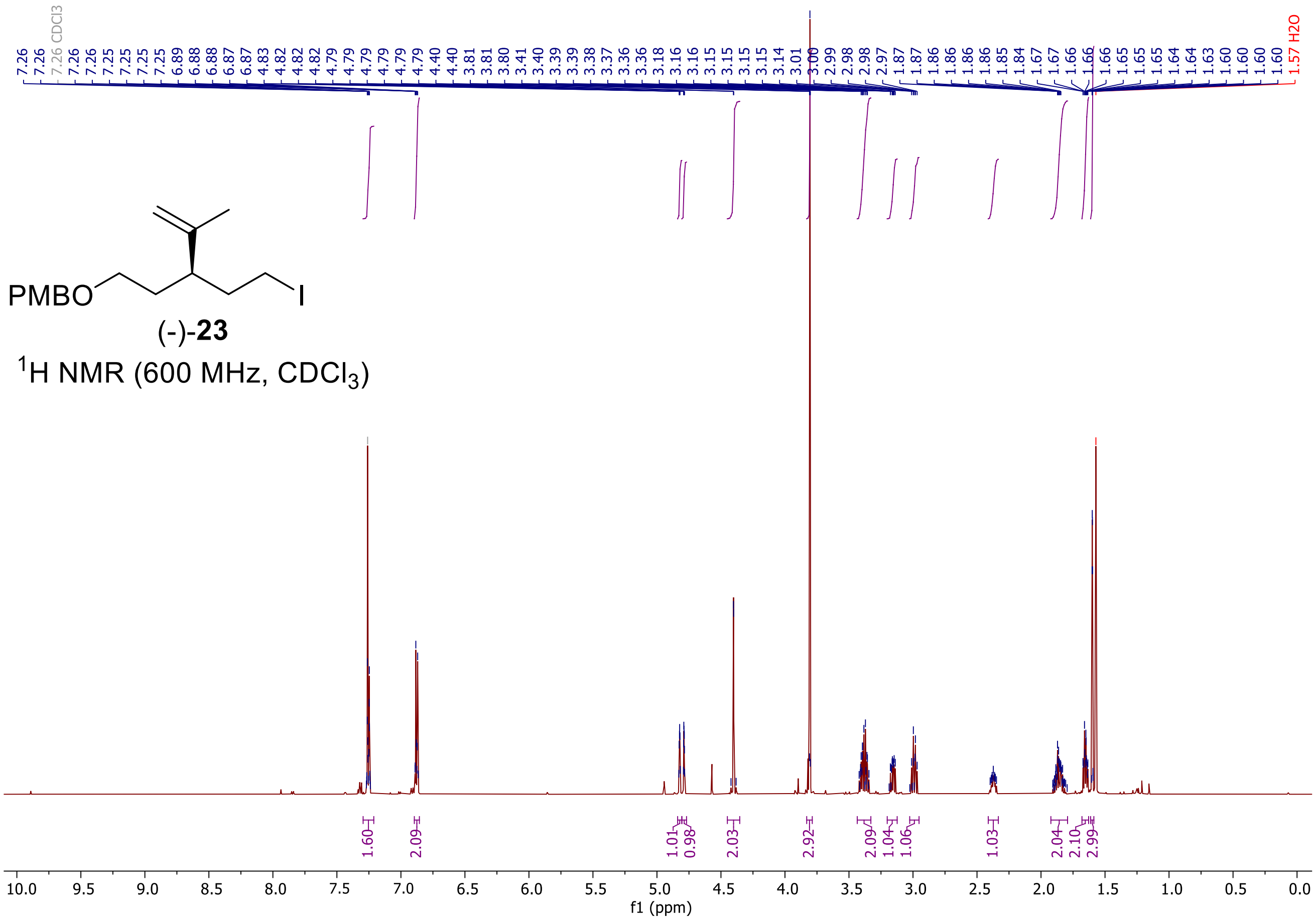
7.26 7.26 7.26 CDCl3 7.26 7.25 7.25 7.25 7.25 6.89 6.88 6.88 6.87 6.87 4.83 4.82 4.82 4.82 4.79 4.79 4.79 4.79 4.79 4.40 4.40 3.81 3.81 3.80 3.41 3.40 3.39 3.39 3.38 3.37 3.36 3.36 3.18 3.16 3.16 3.15 3.15 3.15 3.15 3.14 3.01 3.00 2.99 2.98 2.98 2.97 1.87 1.87 1.86 1.86 1.86 1.85 1.84 1.67 1.67 1.66 1.66 1.65 1.65 1.65 1.64 1.63 1.60 1.60 1.60 1.60 1.57 H2O
PMBO
I
(-)-**23**
^{1}H NMR (600 MHz, $CDCl_3$)
1.60 2.09 1.01 0.98 2.03 2.92 2.09 1.04 1.06 1.03 2.04 2.10 2.99
f1 (ppm)

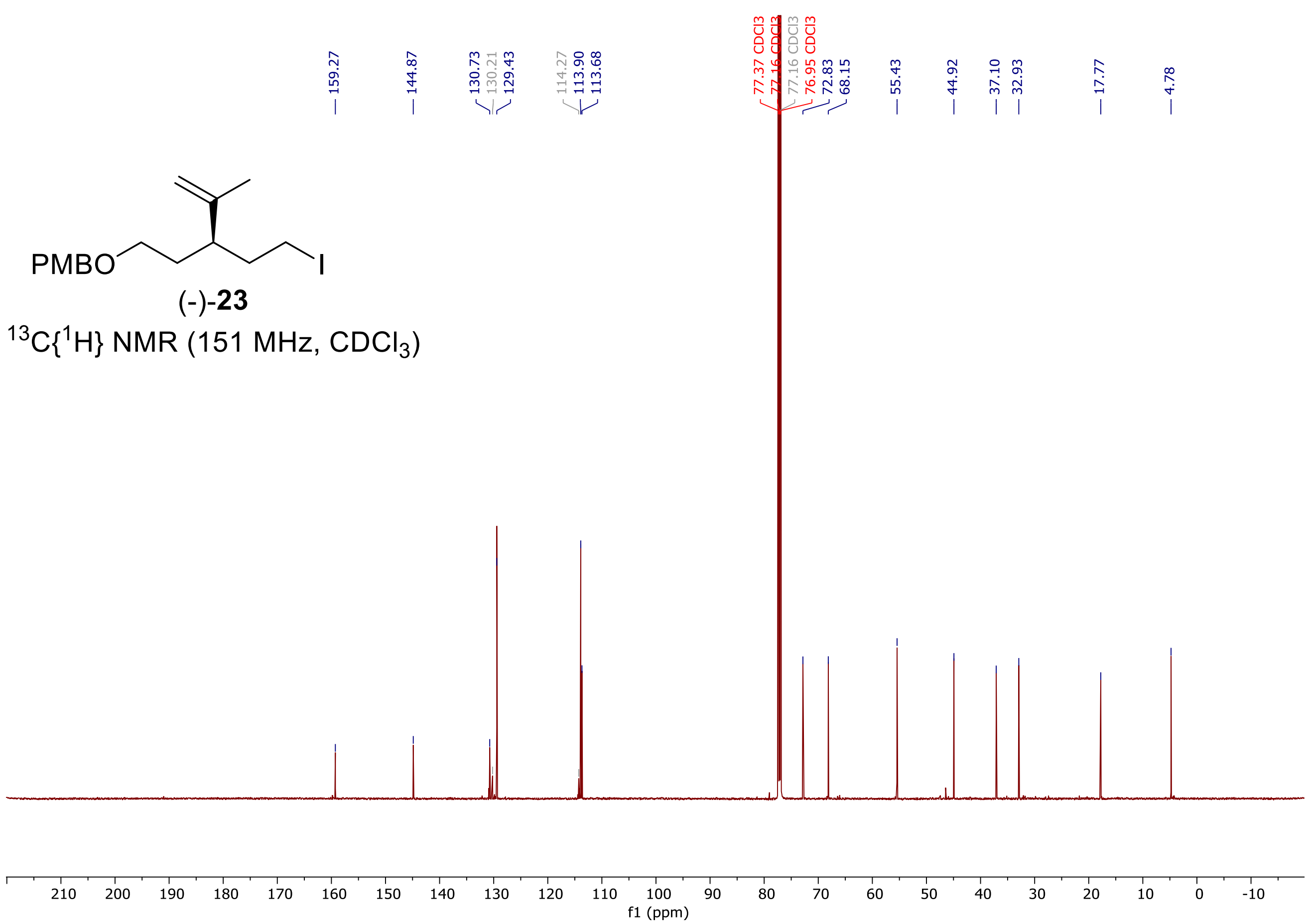
159.27 144.87 130.73 130.21 129.43 114.27 113.90 113.68 77.37 CDCl3 77.16 CDCl3 77.16 CDCl3 76.95 CDCl3 72.83 68.15 55.43 44.92 37.10 32.93 17.77 4.78
PMBO
I
(-)-**23**
^{13}C{^{1}H} NMR (151 MHz, $CDCl_3$)
f1 (ppm)

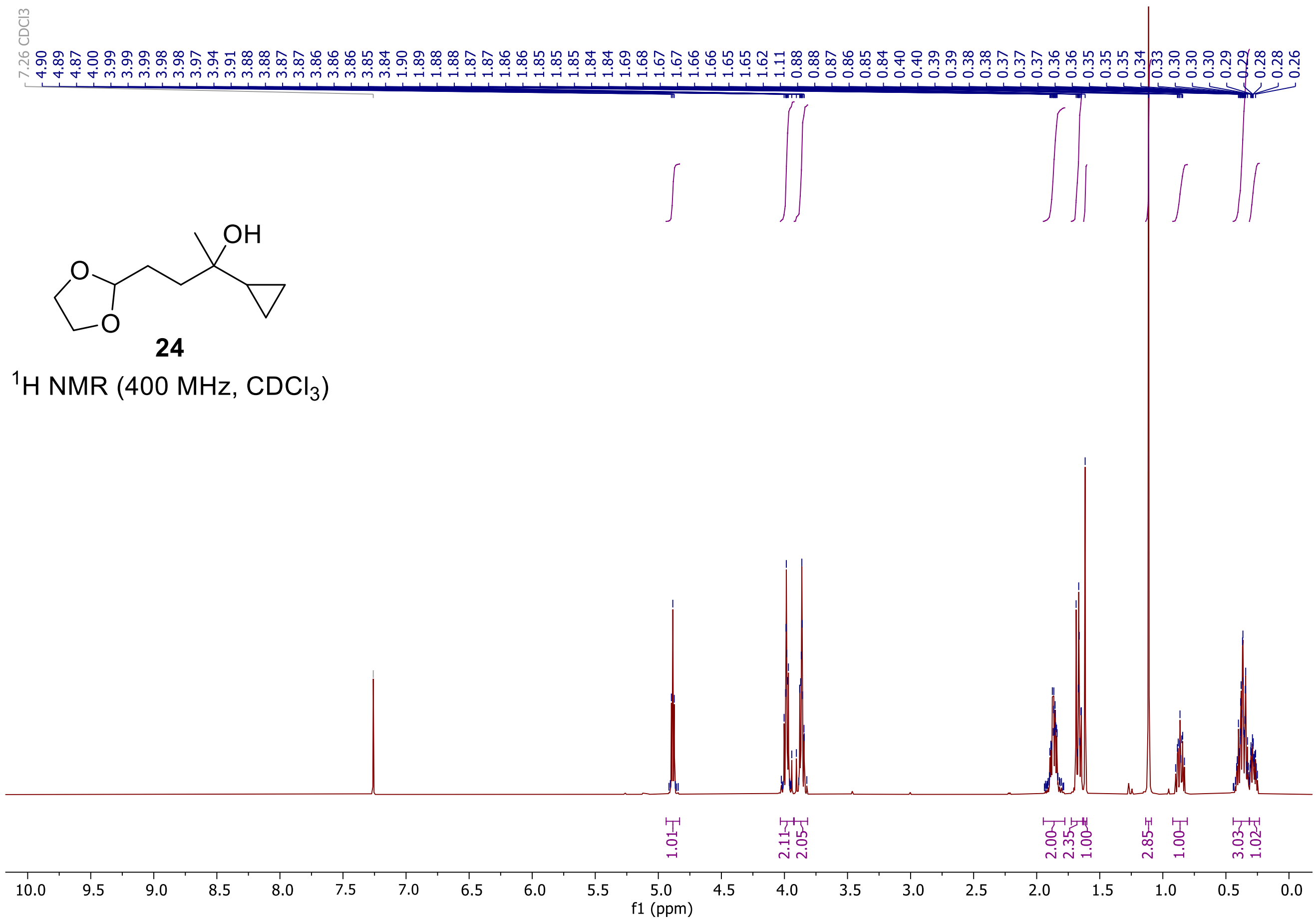
24
^{1}H NMR (400 MHz, $CDCl_3$)
f1 (ppm)

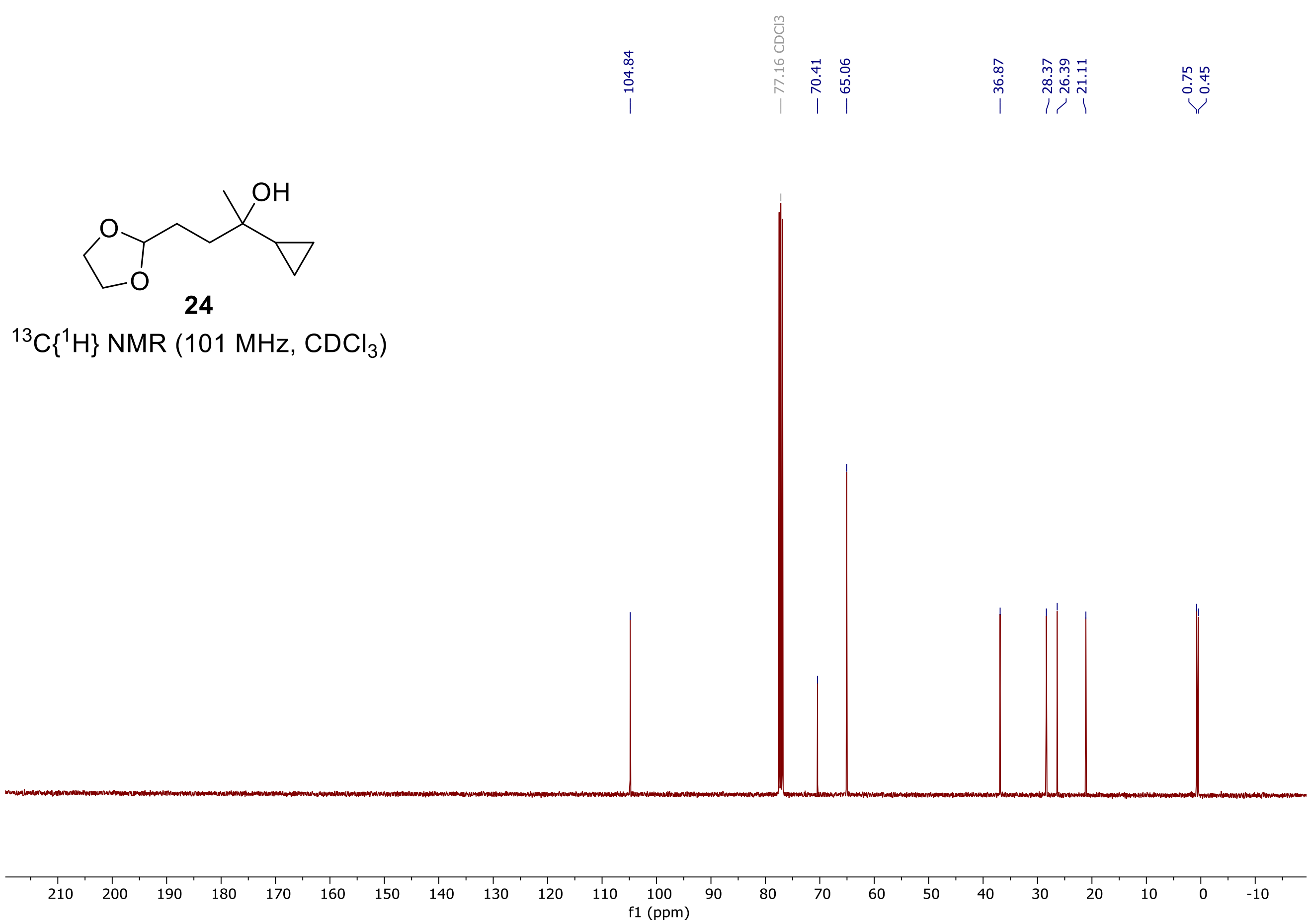
24
$^{13}C\{^{1}H\}$ NMR (101 MHz, $CDCl_3$)
104.84
77.16 CDCl3
70.41
65.06
36.87
28.37
26.39
21.11
0.75
0.45
f1 (ppm)

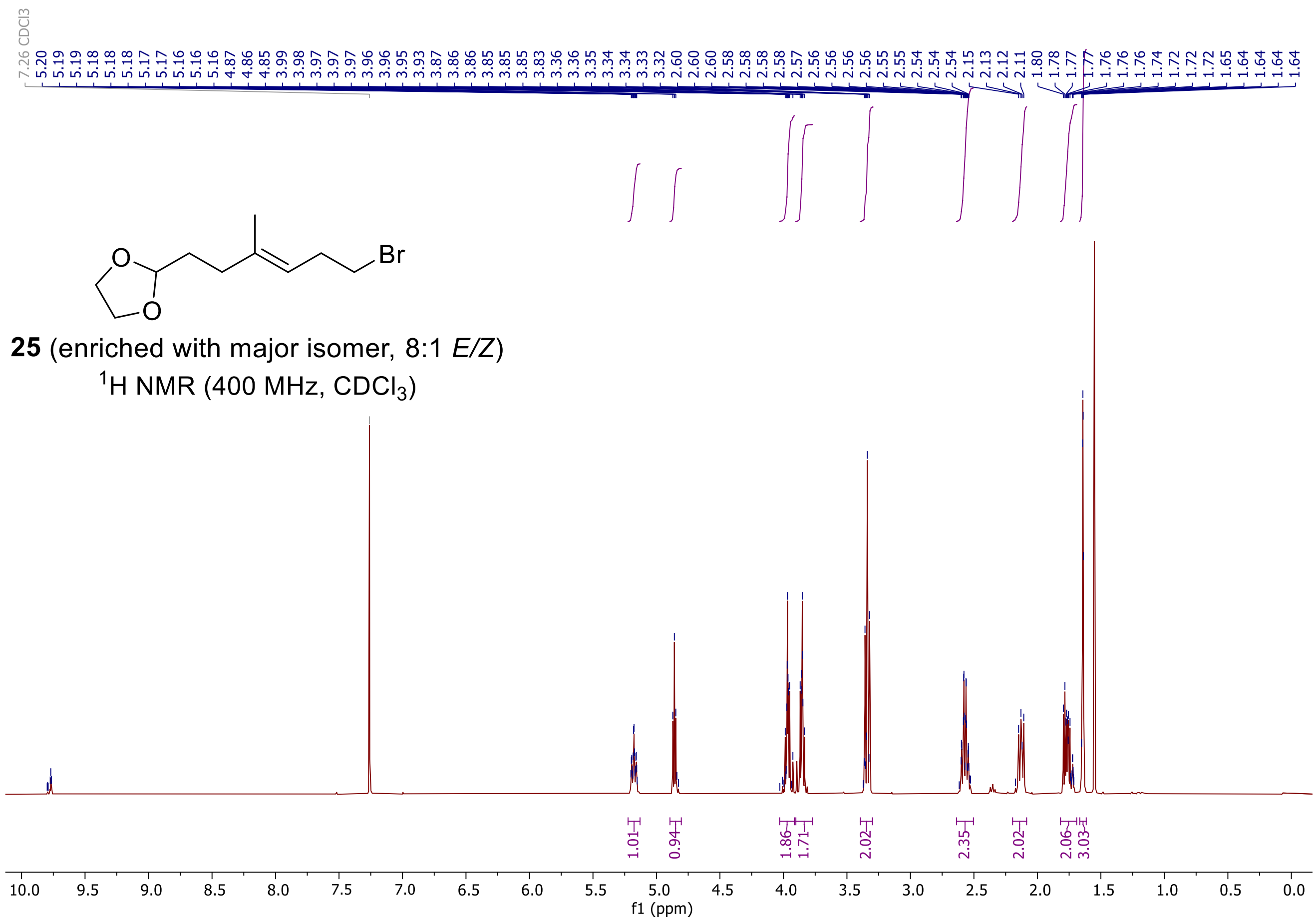

25 (enriched with major isomer, 8:1 *E/Z*)

^{1}H NMR (400 MHz, $CDCl_3$)

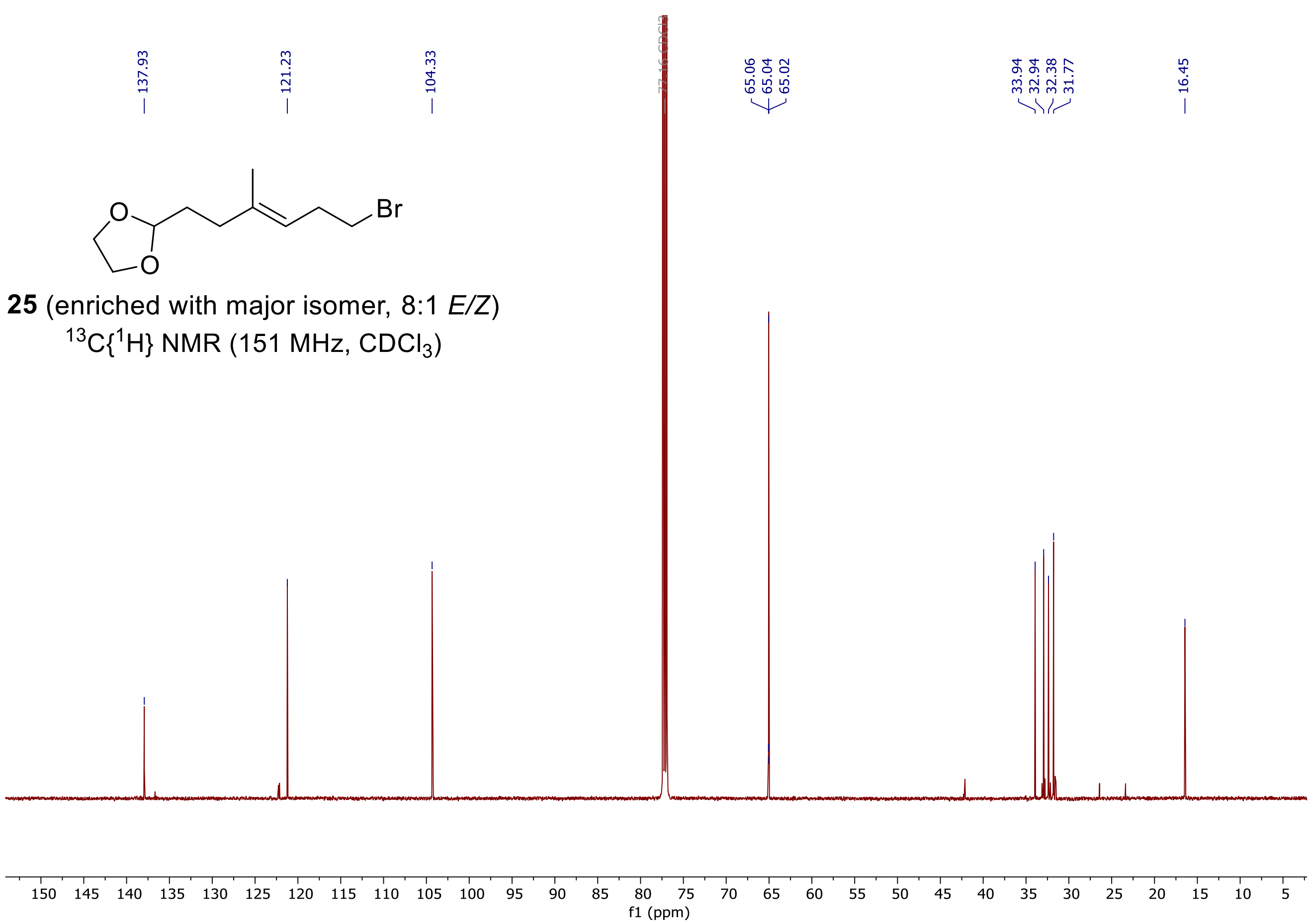

25 (enriched with major isomer, 8:1 *E/Z*)

^{13}C{^{1}H} NMR (151 MHz, $CDCl_3$)

25 (enriched with major isomer, 8:1 *E/Z*)

^{1}H NMR (600 MHz, $CDCl_3$)

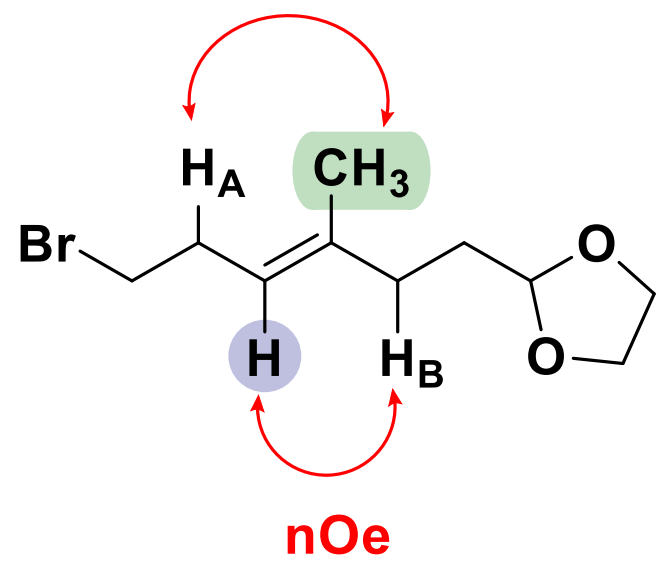

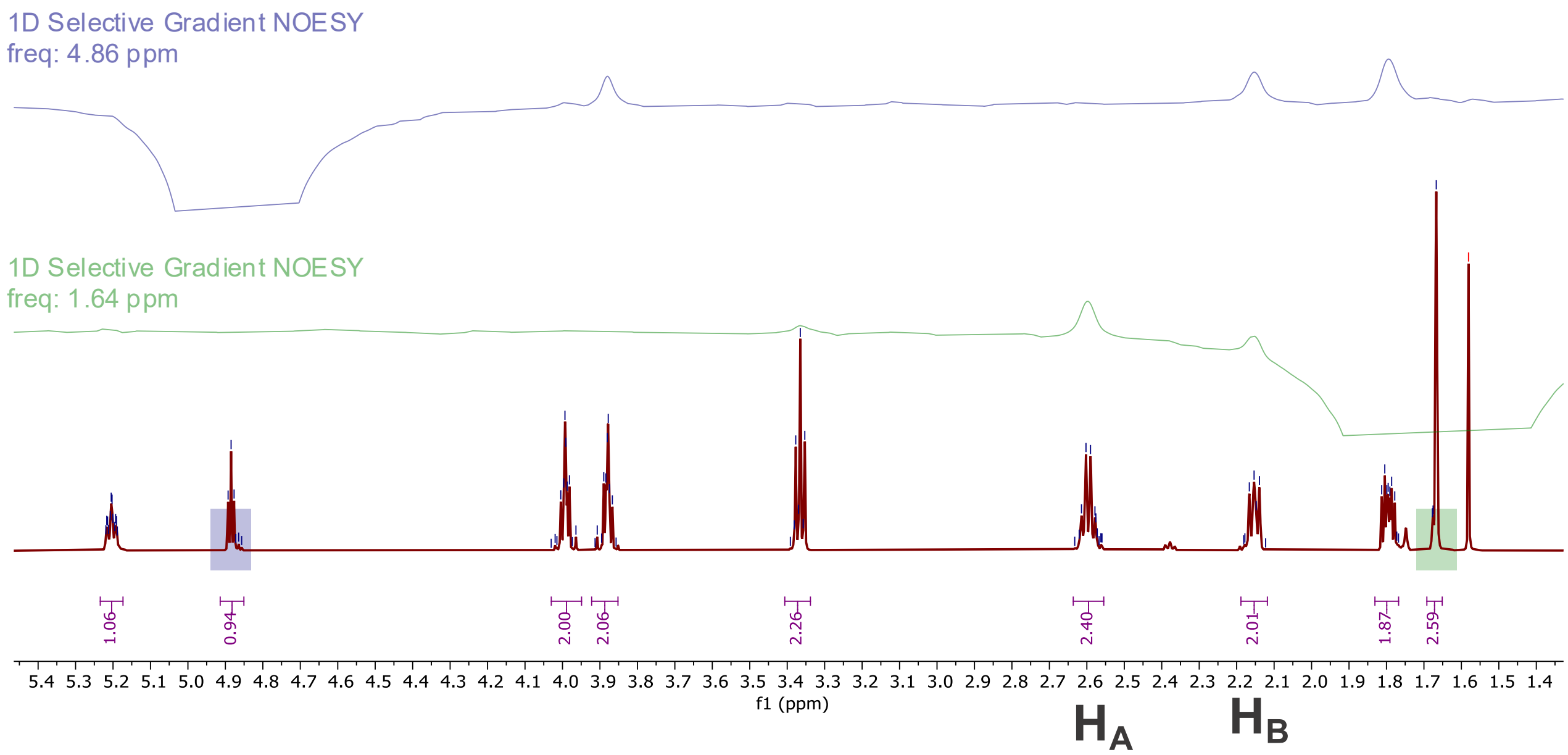

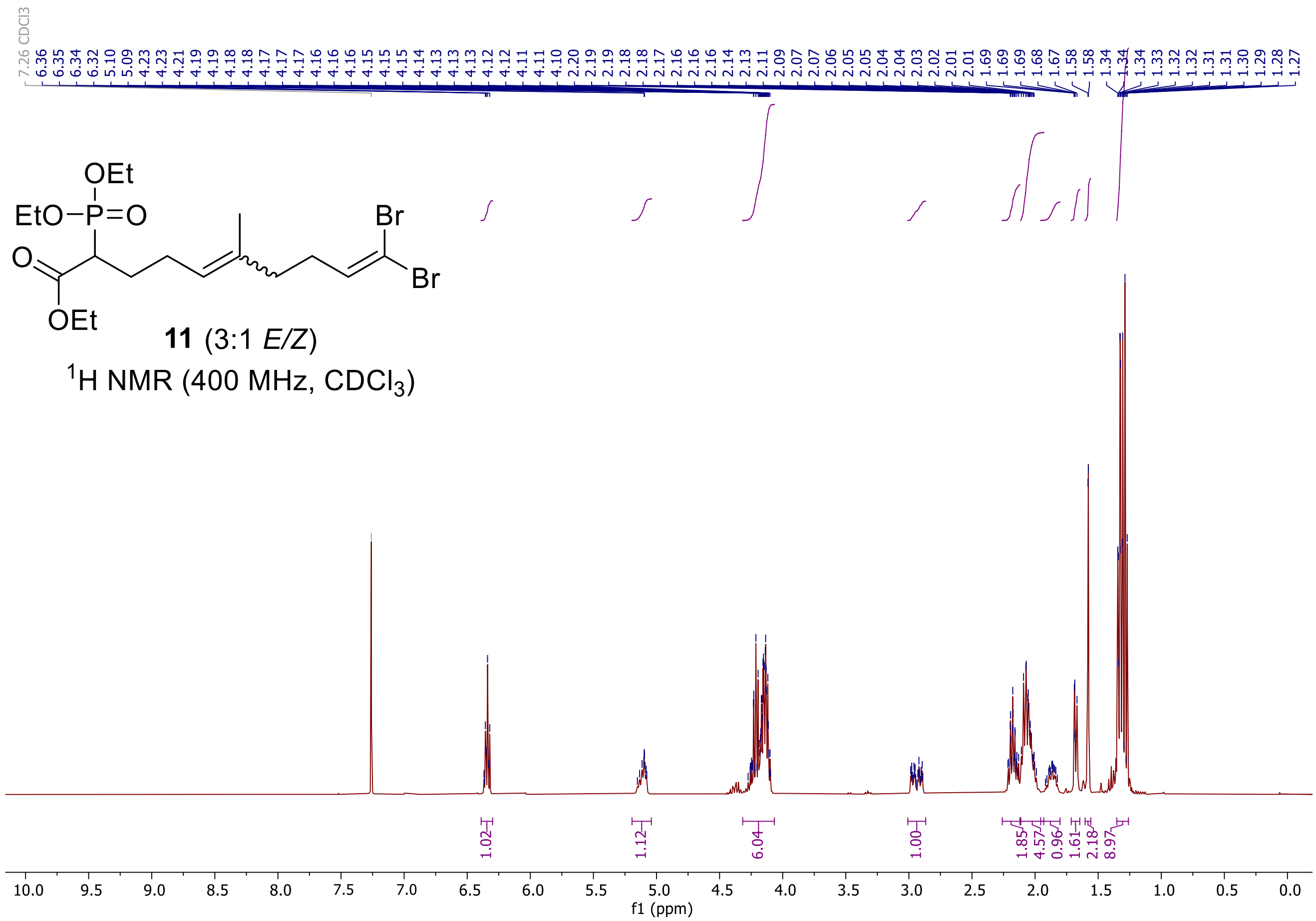

7.26 CDCl3
6.36 6.35 6.34 6.32 5.10 5.09 4.23 4.23 4.21 4.19 4.19 4.18 4.18 4.17 4.17 4.17 4.16 4.16 4.16 4.15 4.15 4.15 4.14 4.13 4.13 4.13 4.12 4.12 4.11 4.11 4.10 2.20 2.19 2.19 2.18 2.18 2.17 2.16 2.16 2.16 2.14 2.13 2.11 2.09 2.07 2.07 2.06 2.05 2.05 2.04 2.04 2.03 2.02 2.01 2.01 1.69 1.69 1.69 1.68 1.67 1.58 1.58 1.34 1.34 1.34 1.33 1.32 1.32 1.31 1.31 1.30 1.29 1.28 1.27
OEt
EtO
P
O
O
OEt
Br
Br
11 (3:1 E/Z)
1H NMR (400 MHz, CDCl3)
1.02
1.12
6.04
1.00
1.85
4.57
0.96
1.61
2.18
8.97
f1 (ppm)

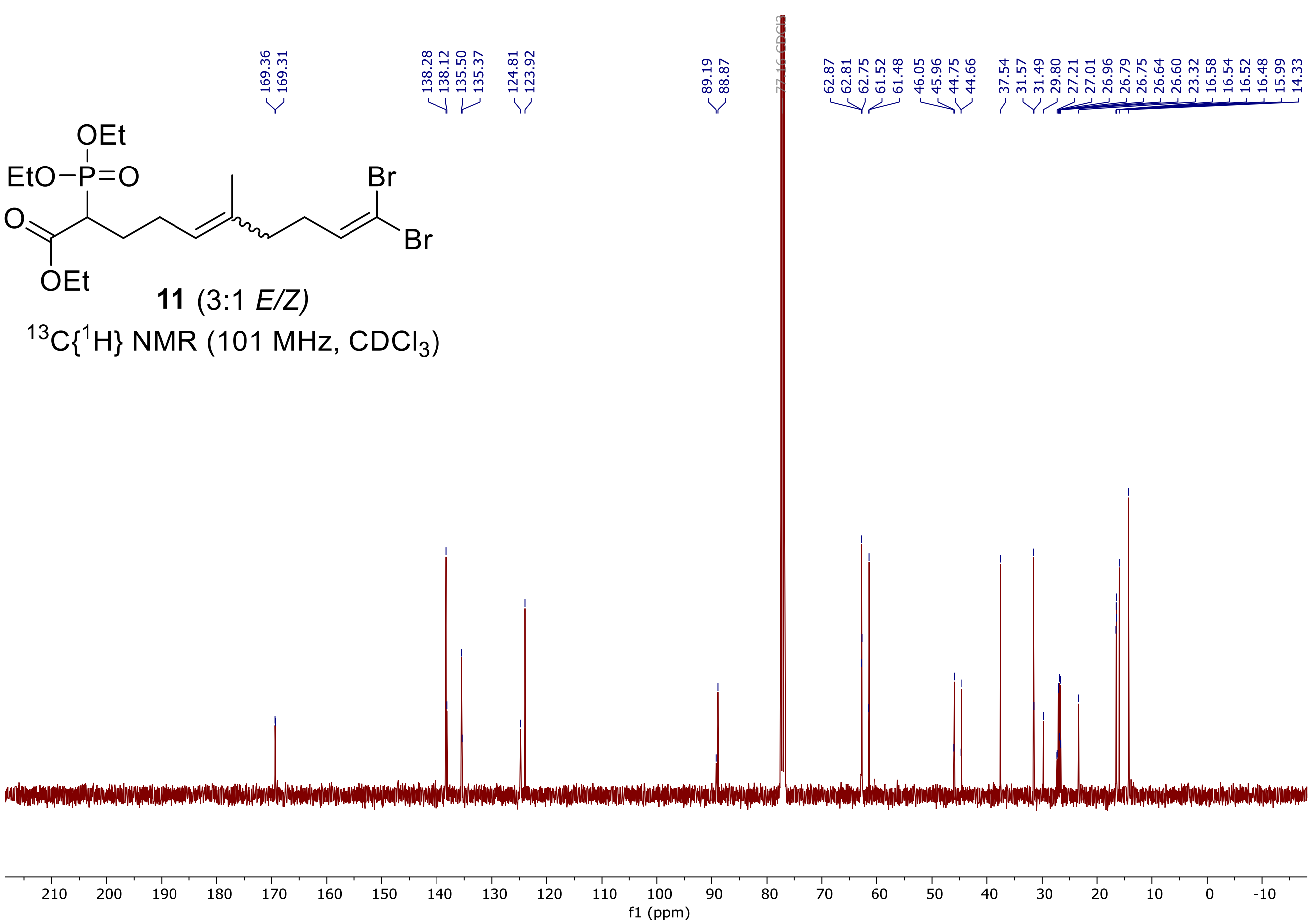

169.36 169.31
138.28 138.12 135.50 135.37
124.81 123.92
89.19 88.87
77.16 CDCl3
62.87 62.81 62.75 61.52 61.48 46.05 45.96 44.75 44.66 37.54 31.57 31.49 29.80 27.21 27.01 26.96 26.79 26.75 26.64 26.60 23.32 16.58 16.54 16.52 16.48 15.99 14.33
OEt
EtO
P
O
O
OEt
Br
Br
11 (3:1 E/Z)
13C{1H} NMR (101 MHz, CDCl3)
f1 (ppm)

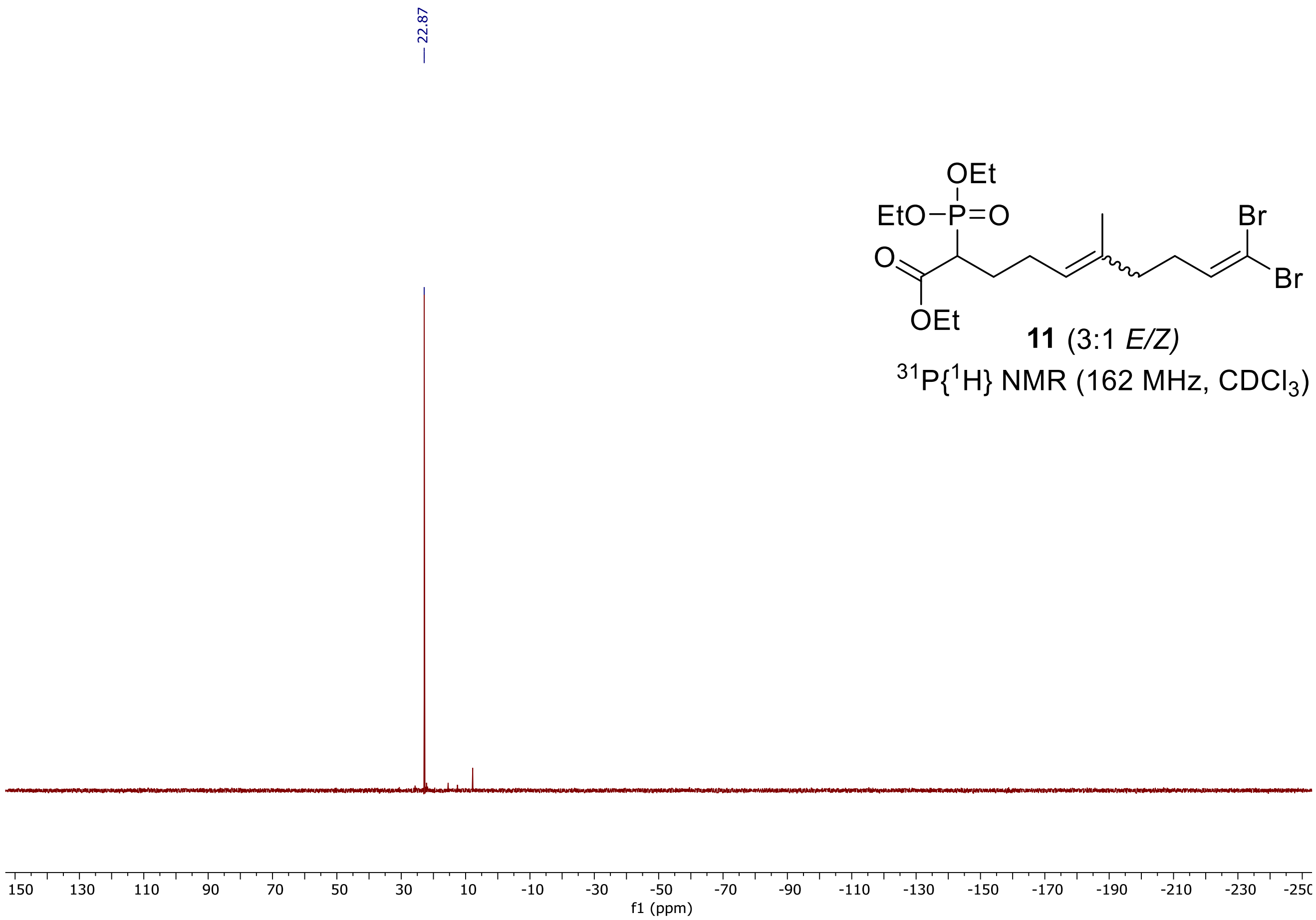

11 (3:1 *E/Z*)

$^{31}P\{^{1}H\}$ NMR (162 MHz, $CDCl_3$)

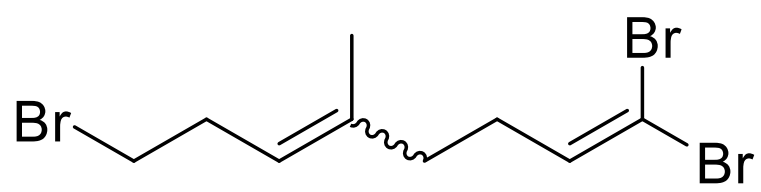

29 (3:1 *E/Z*)
^{1}H NMR (400 MHz, $CDCl_3$)

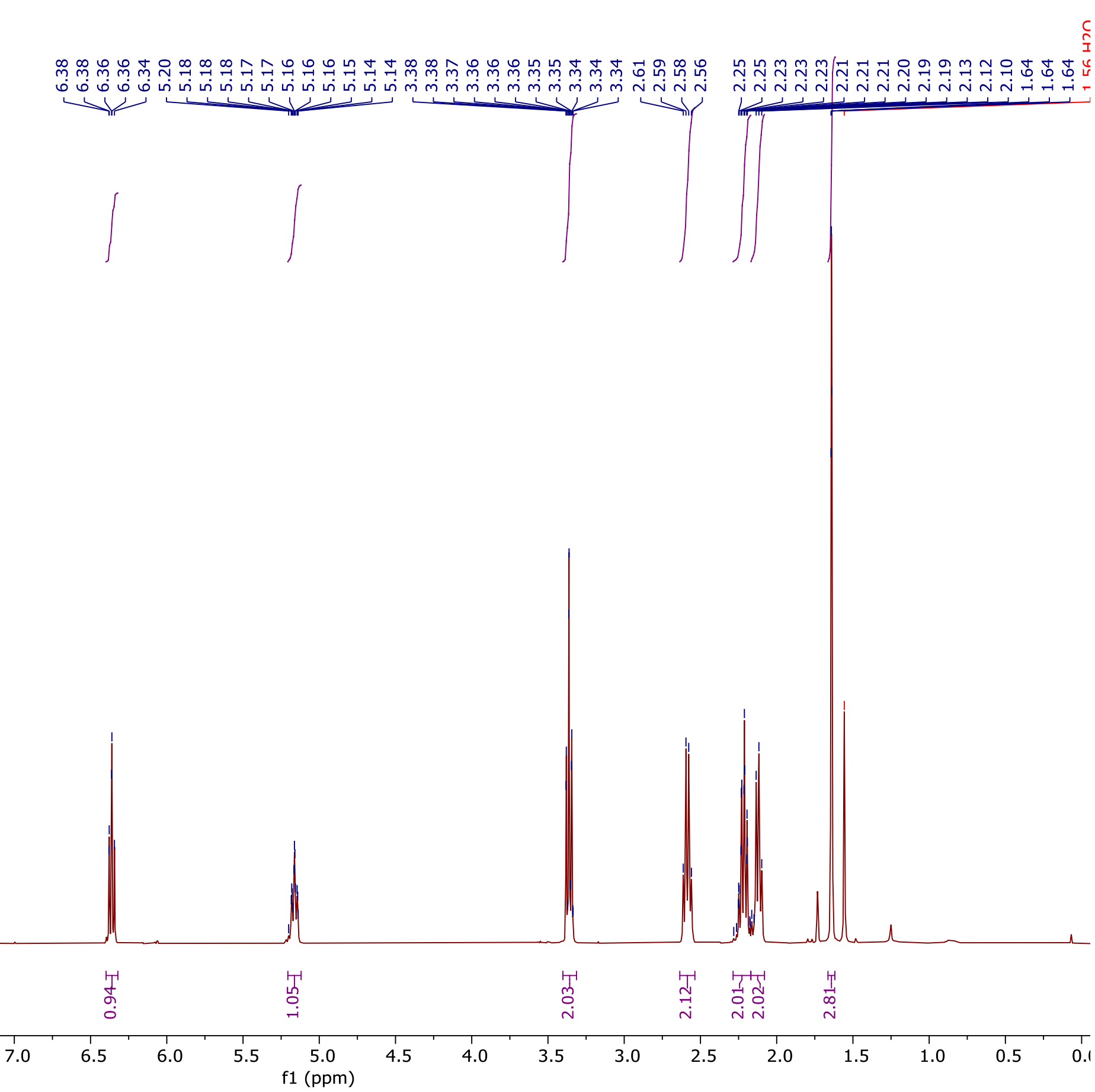

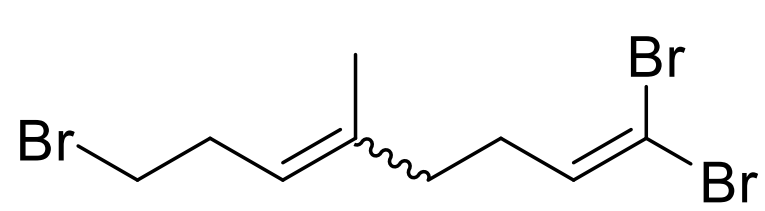

29 (3:1 *E/Z*)
^{13}C{^{1}H} NMR (101 MHz, $CDCl_3$)

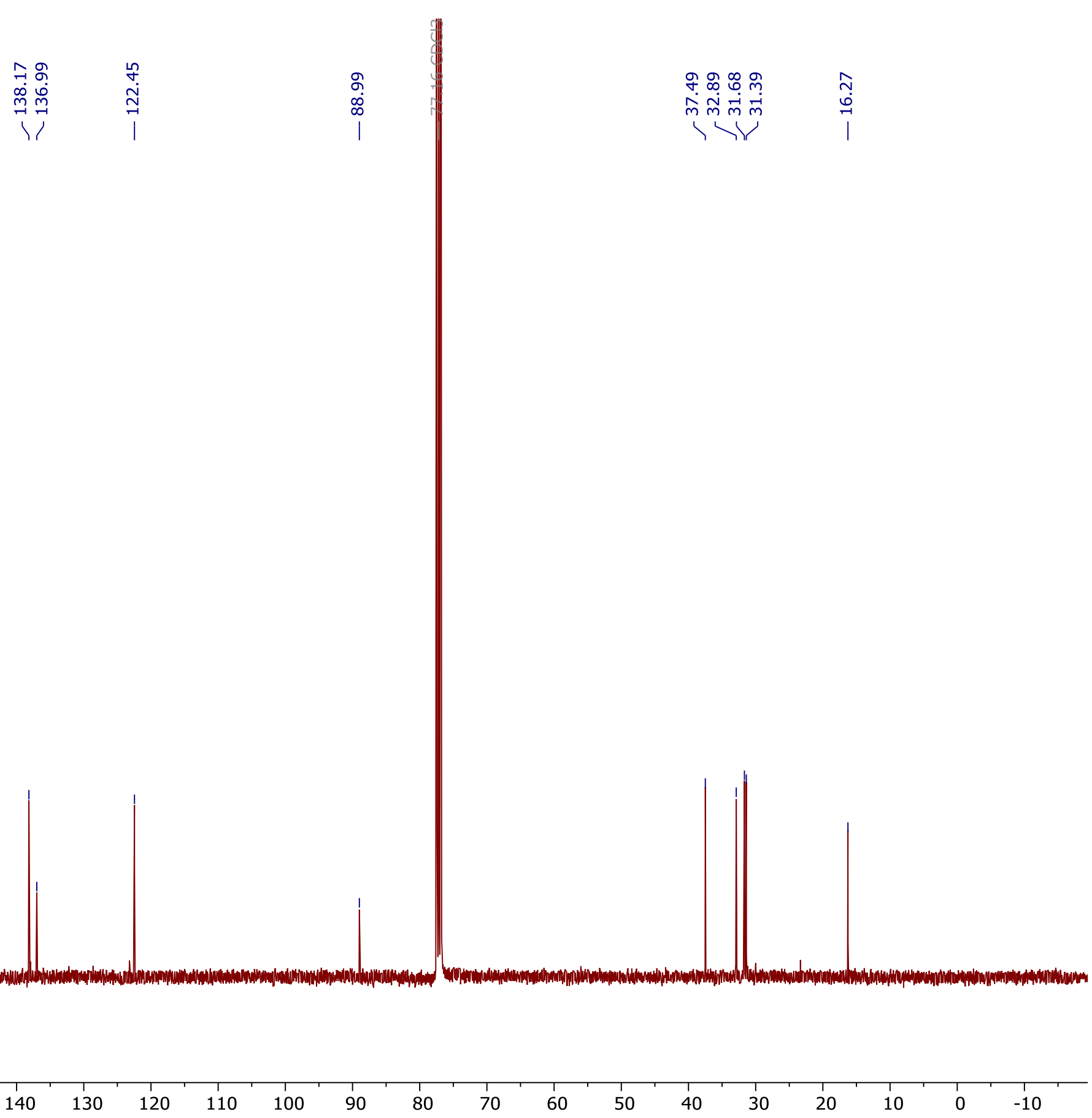

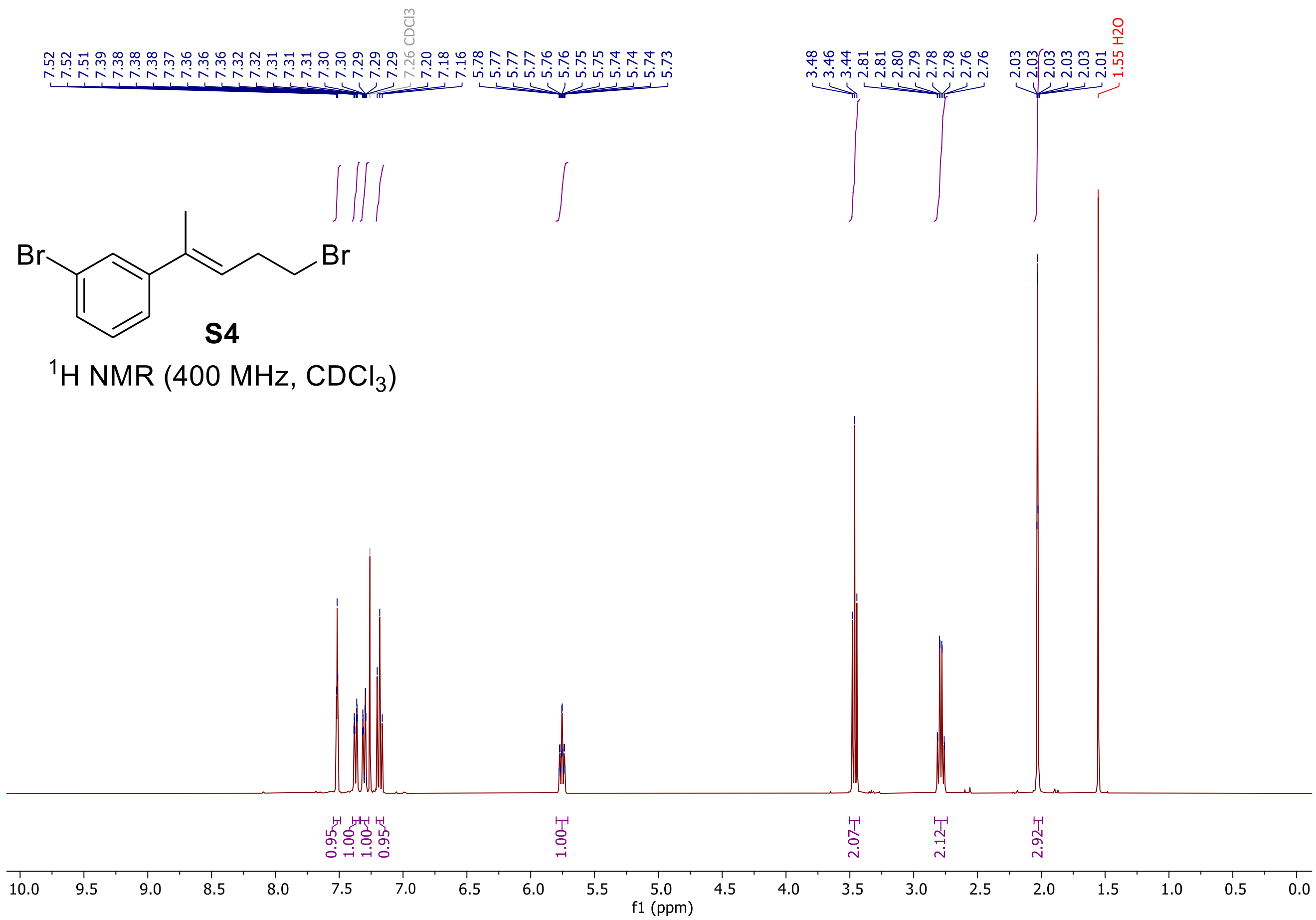
S4
1H NMR (400 MHz, CDCl3)

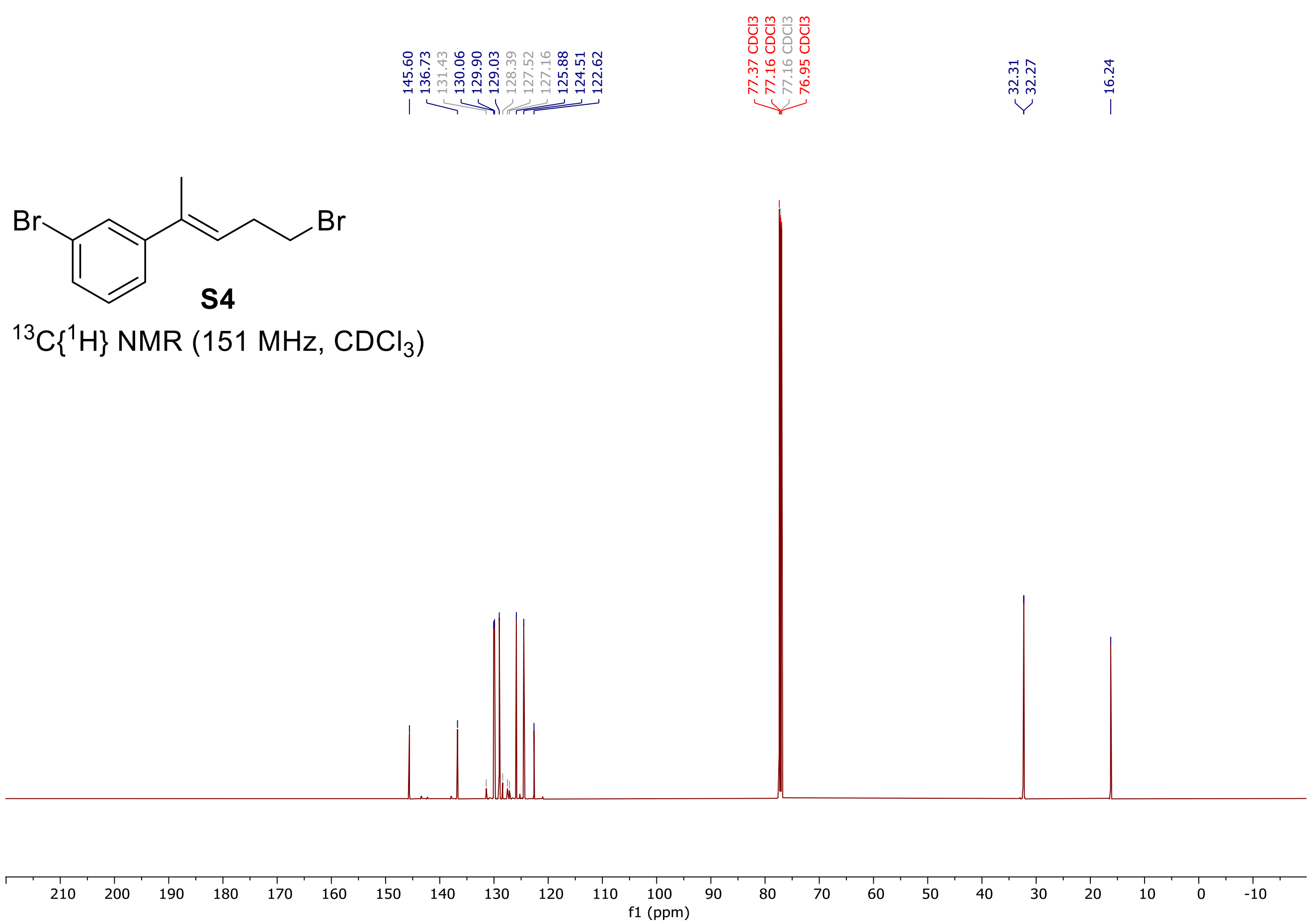
S4
13C{1H} NMR (151 MHz, CDCl3)

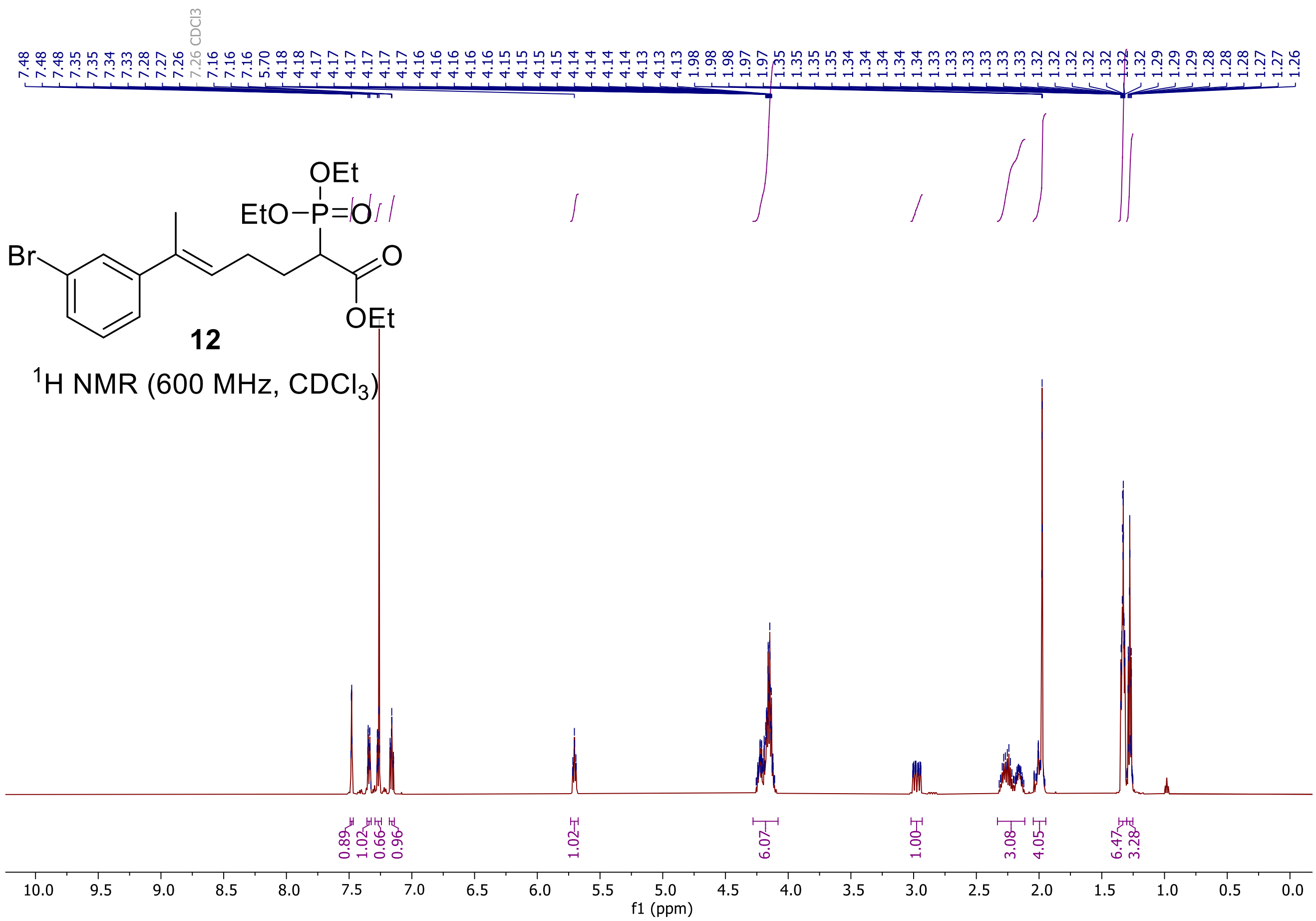
OEt
EtO–P=O
Br
O
OEt
12
^{1}H NMR (600 MHz, $CDCl_3$)
f1 (ppm)

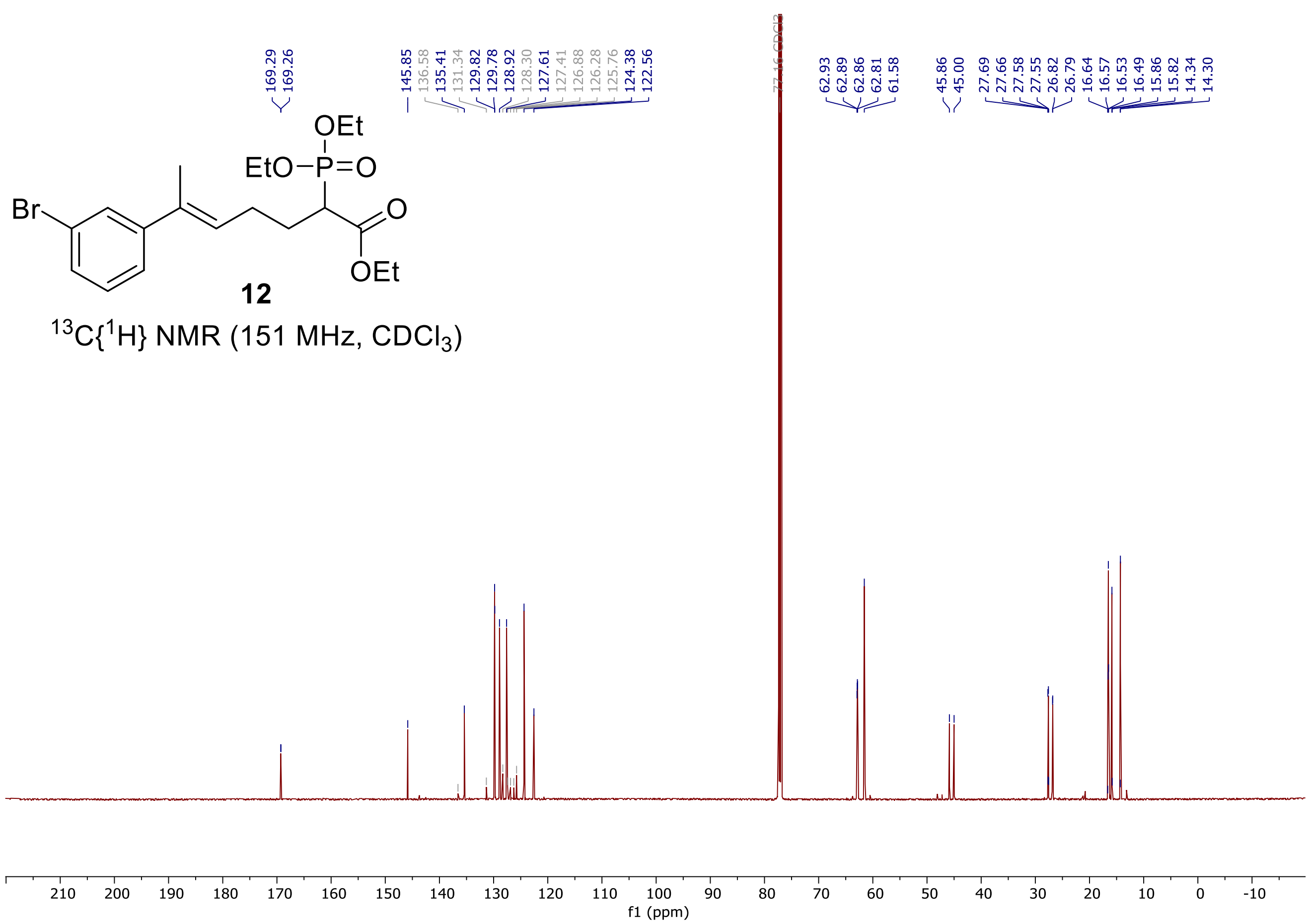
OEt
EtO–P=O
Br
O
OEt
12
^{13}C{^{1}H} NMR (151 MHz, $CDCl_3$)
f1 (ppm)

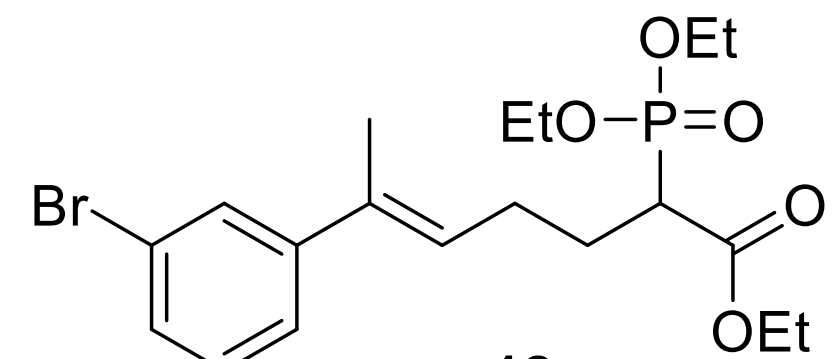

12

$^{31}P\{^{1}H\}$ NMR (243 MHz, $CDCl_3$)

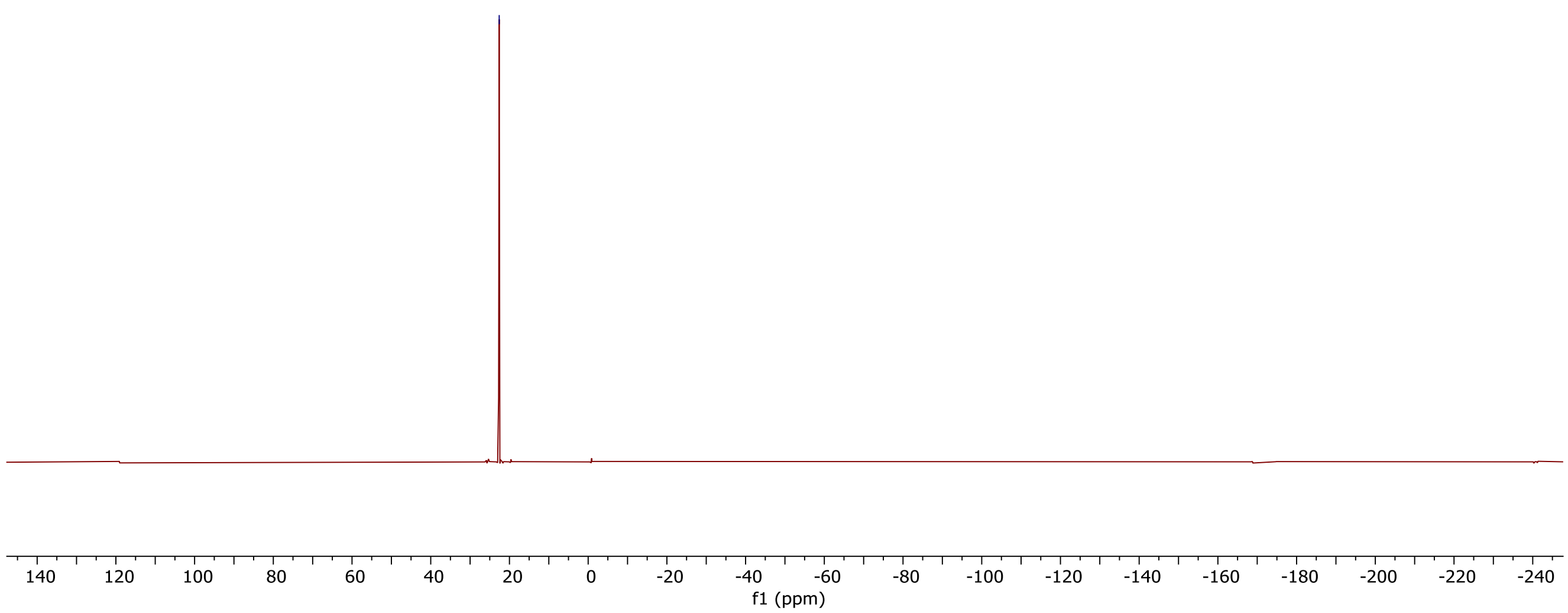

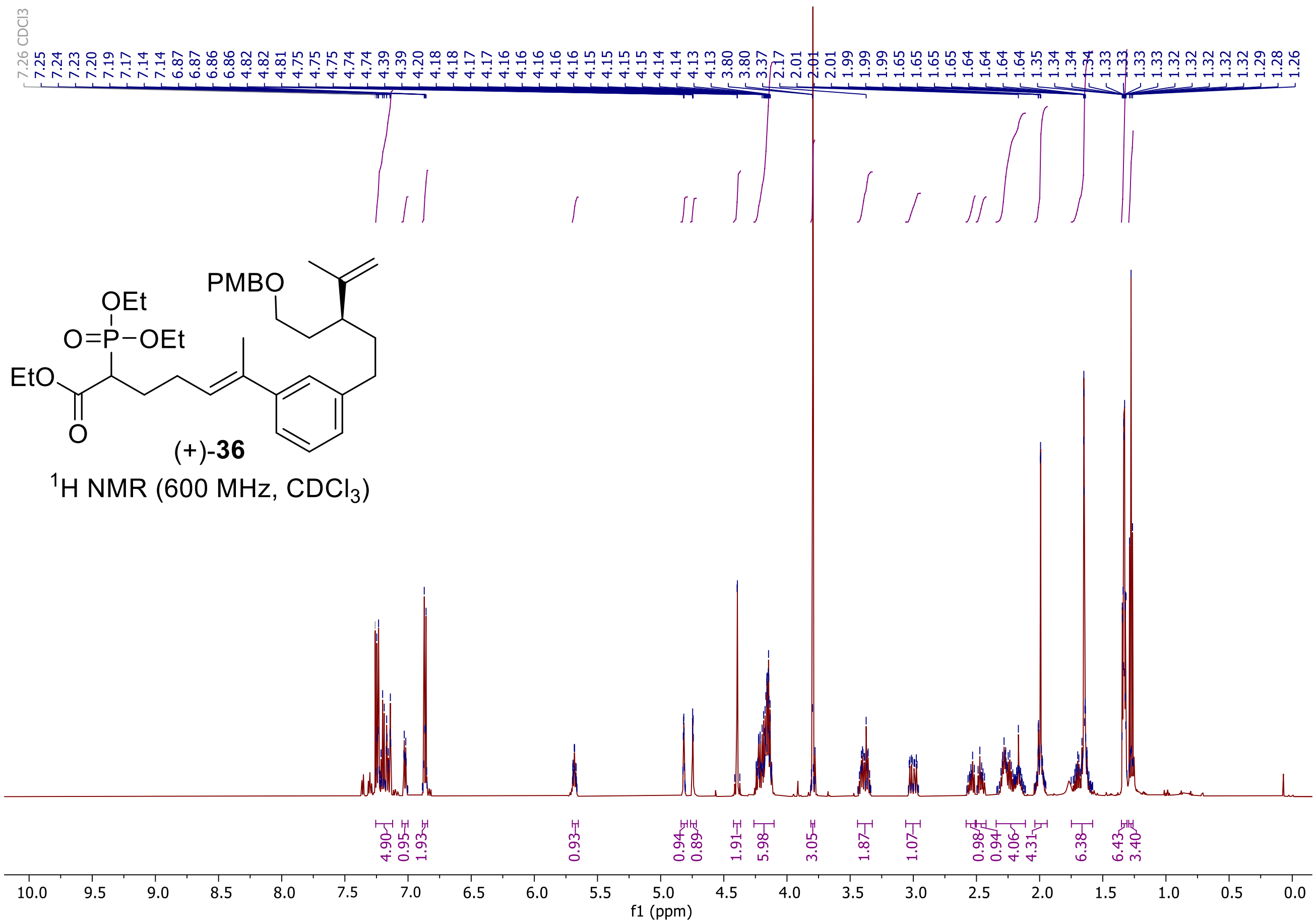
7.26 CDCl3
7.25 7.24 7.23 7.20 7.19 7.17 7.14 7.14 6.87 6.87 6.86 6.86 4.82 4.82 4.81 4.75 4.75 4.75 4.74 4.74 4.39 4.39 4.20 4.18 4.18 4.17 4.17 4.16 4.16 4.16 4.16 4.16 4.15 4.15 4.15 4.15 4.14 4.14 4.13 4.13 3.80 3.80 3.37 2.17 2.01 2.01 2.01 1.99 1.99 1.99 1.65 1.65 1.65 1.64 1.64 1.64 1.64 1.35 1.34 1.34 1.34 1.33 1.33 1.33 1.33 1.32 1.32 1.32 1.32 1.32 1.29 1.28 1.26
PMBO
OEt
O=P−OEt
EtO
O
(+)-36
1H NMR (600 MHz, CDCl3)
4.90 0.95 1.93 0.93 0.94 0.89 1.91 5.98 3.05 1.87 1.07 0.98 0.94 4.06 4.31 6.38 6.43 3.40
10.0 9.5 9.0 8.5 8.0 7.5 7.0 6.5 6.0 5.5 5.0 4.5 4.0 3.5 3.0 2.5 2.0 1.5 1.0 0.5 0.0
f1 (ppm)

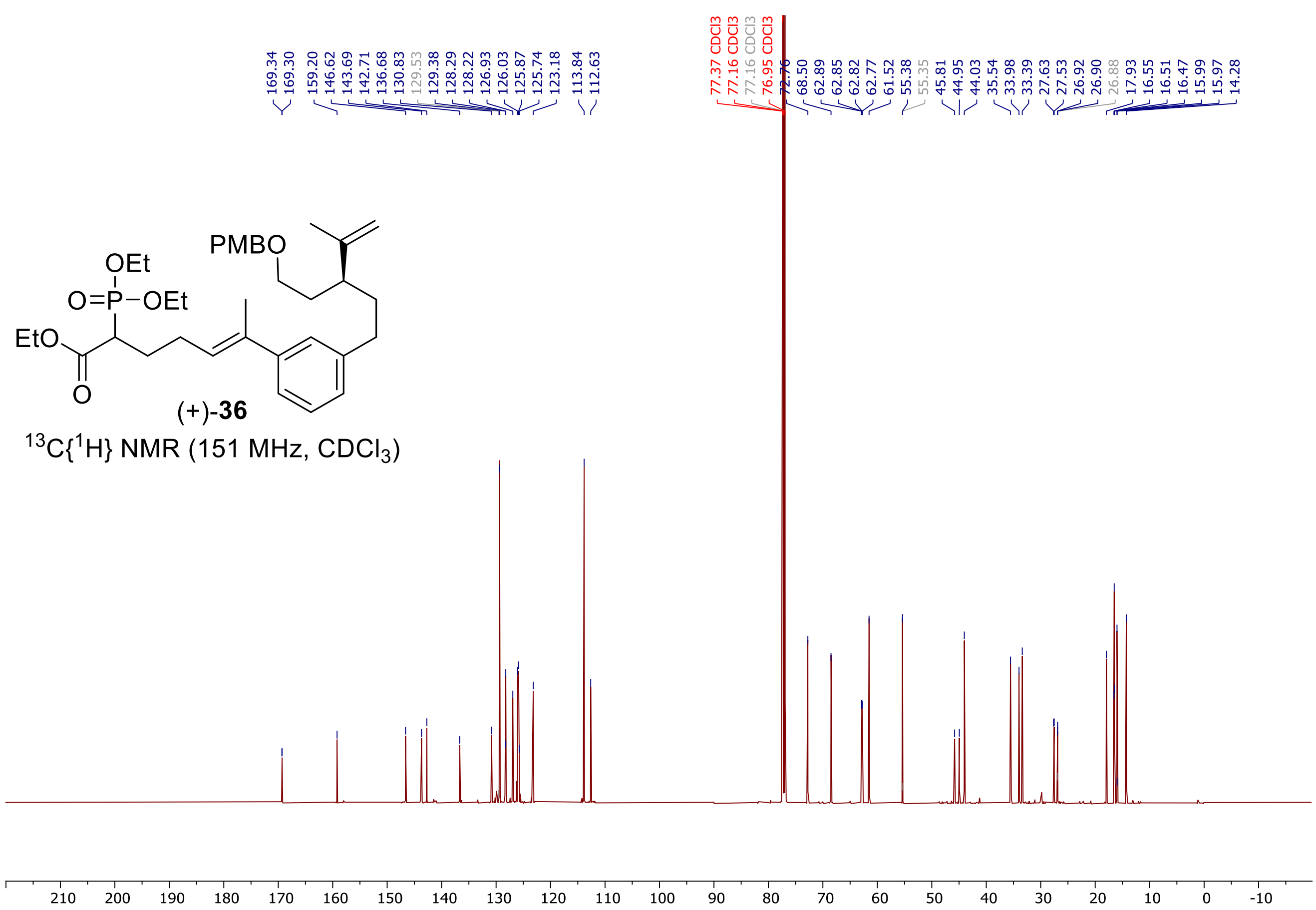
169.34 169.30 159.20 146.62 143.69 142.71 136.68 130.83 129.53 129.38 128.29 128.22 126.93 126.03 125.87 125.74 123.18 113.84 112.63
77.37 CDCl3 77.16 CDCl3 77.16 CDCl3 76.95 CDCl3
72.76 68.50 62.89 62.85 62.82 62.77 61.52 55.38 55.35 45.81 44.95 44.03 35.54 33.98 33.39 27.63 27.53 26.92 26.90 26.88 17.93 16.55 16.51 16.47 15.99 15.97 14.28
PMBO
OEt
O=P−OEt
EtO
O
(+)-36
13C{1H} NMR (151 MHz, CDCl3)
210 200 190 180 170 160 150 140 130 120 110 100 90 80 70 60 50 40 30 20 10 0 -10
f1 (ppm)

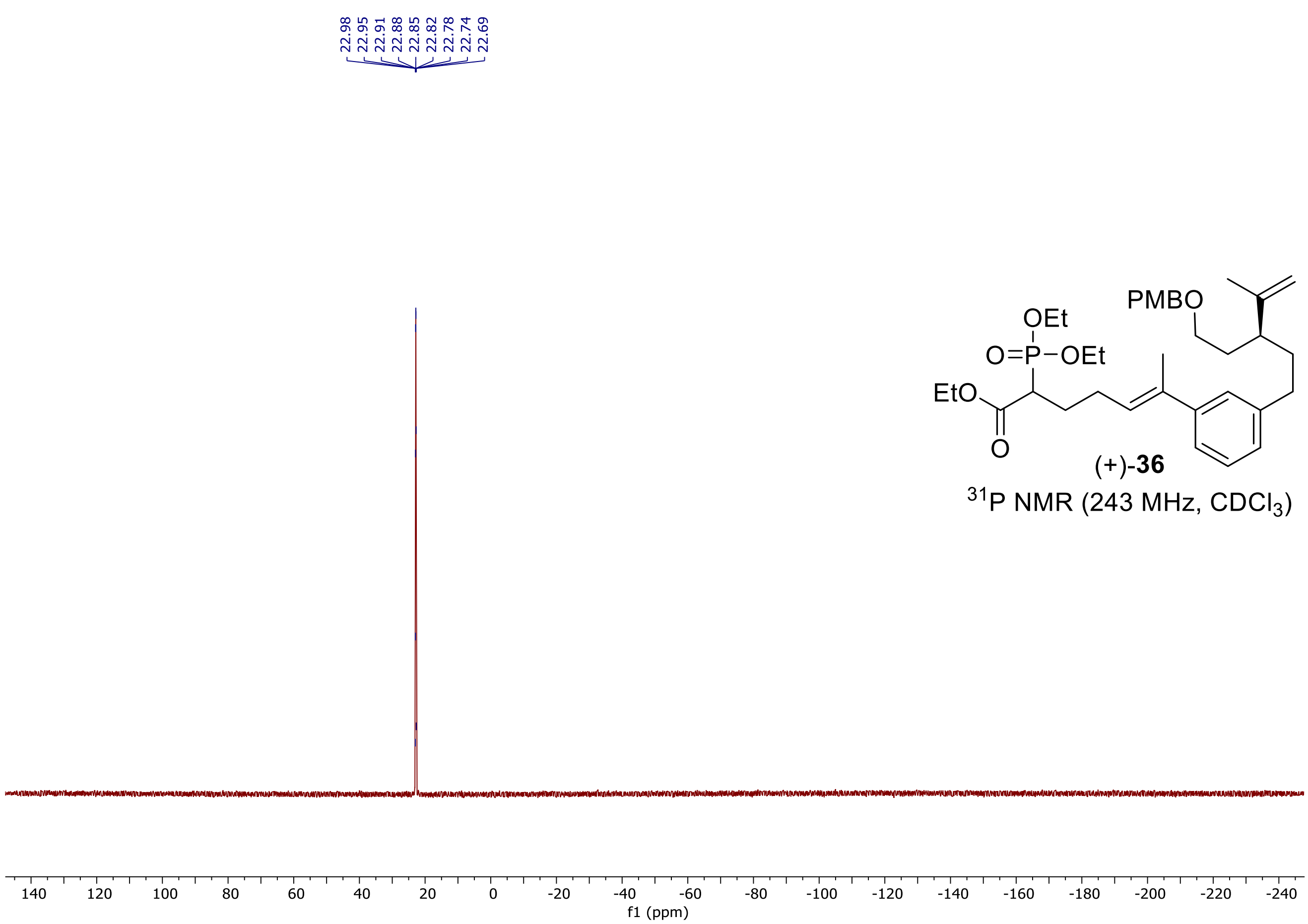

(+)-**36**

^{31}P NMR (243 MHz, $CDCl_3$)

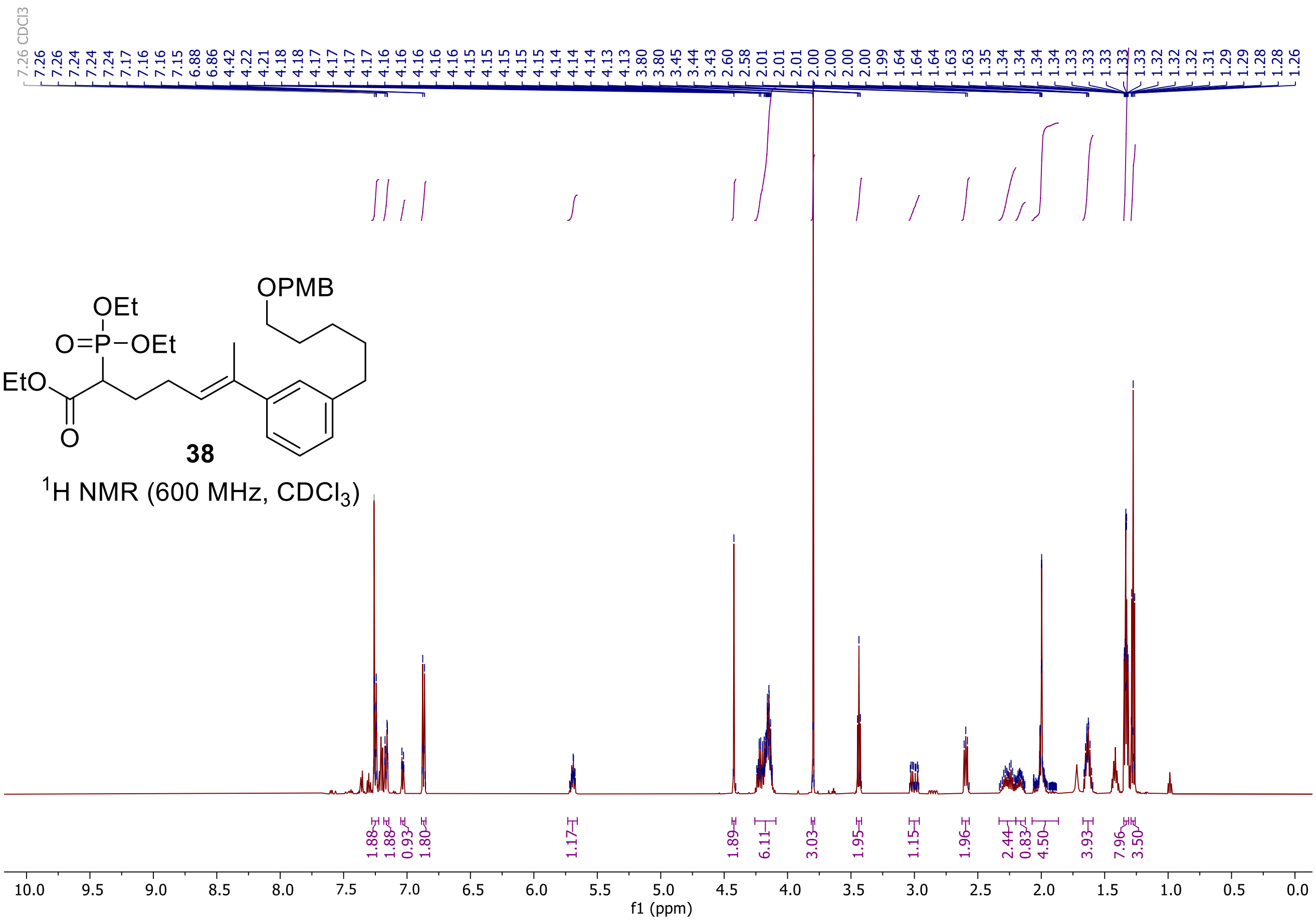
OPMB
OEt
O=P–OEt
EtO
O
38
1H NMR (600 MHz, CDCl3)
f1 (ppm)

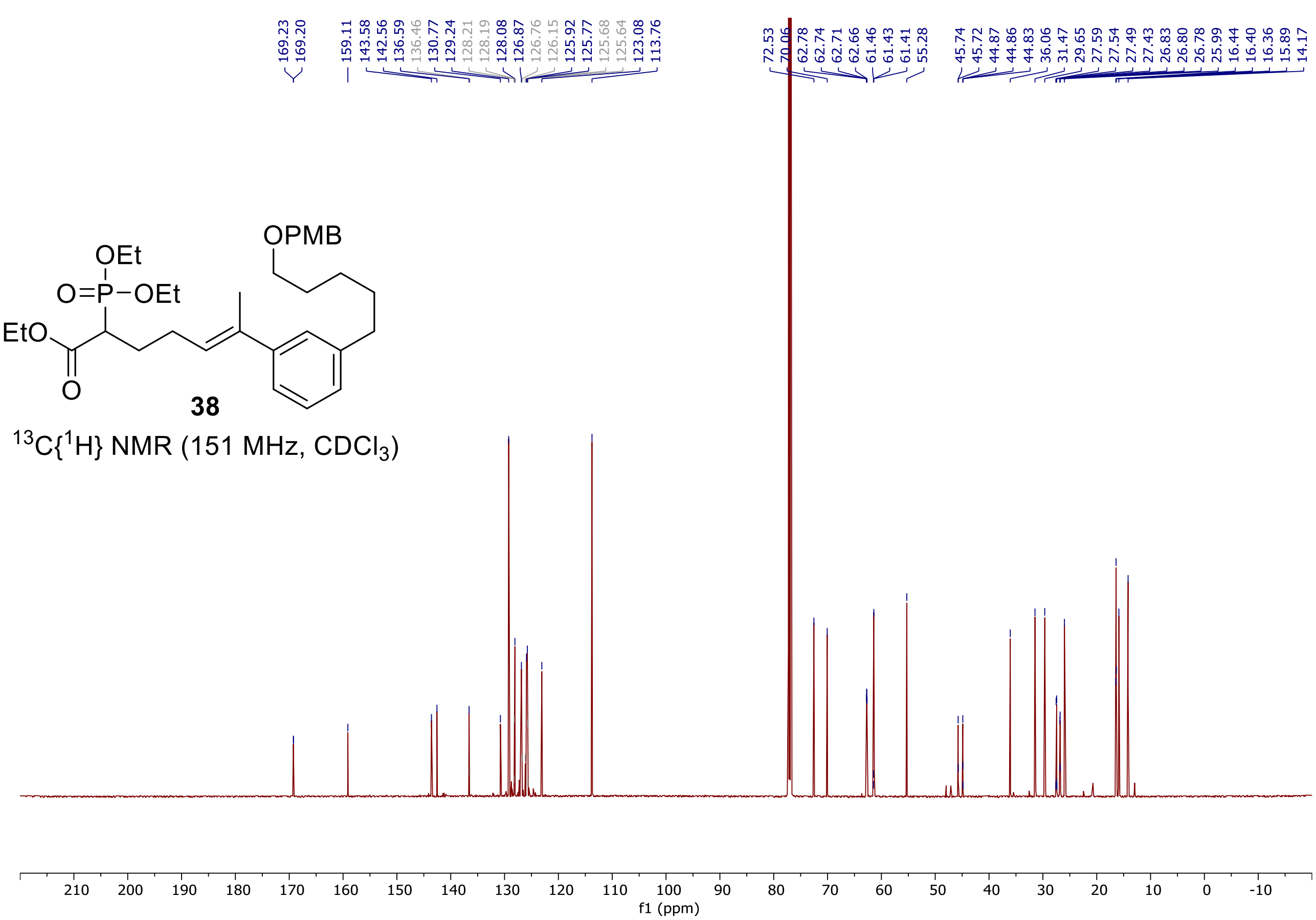
OPMB
OEt
O=P–OEt
EtO
O
38
13C{1H} NMR (151 MHz, CDCl3)
f1 (ppm)

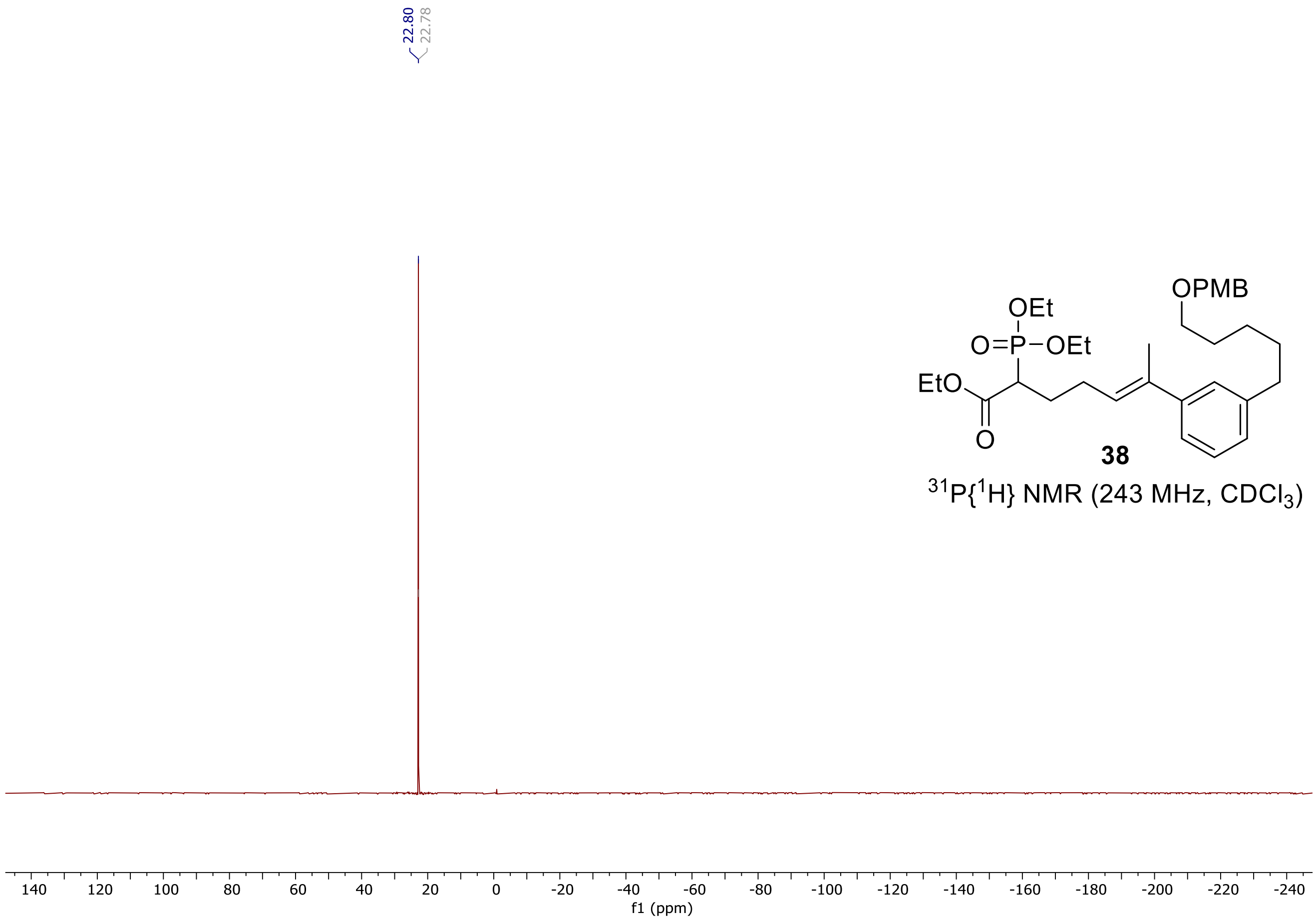

38

$^{31}P\{^{1}H\}$ NMR (243 MHz, $CDCl_3$)

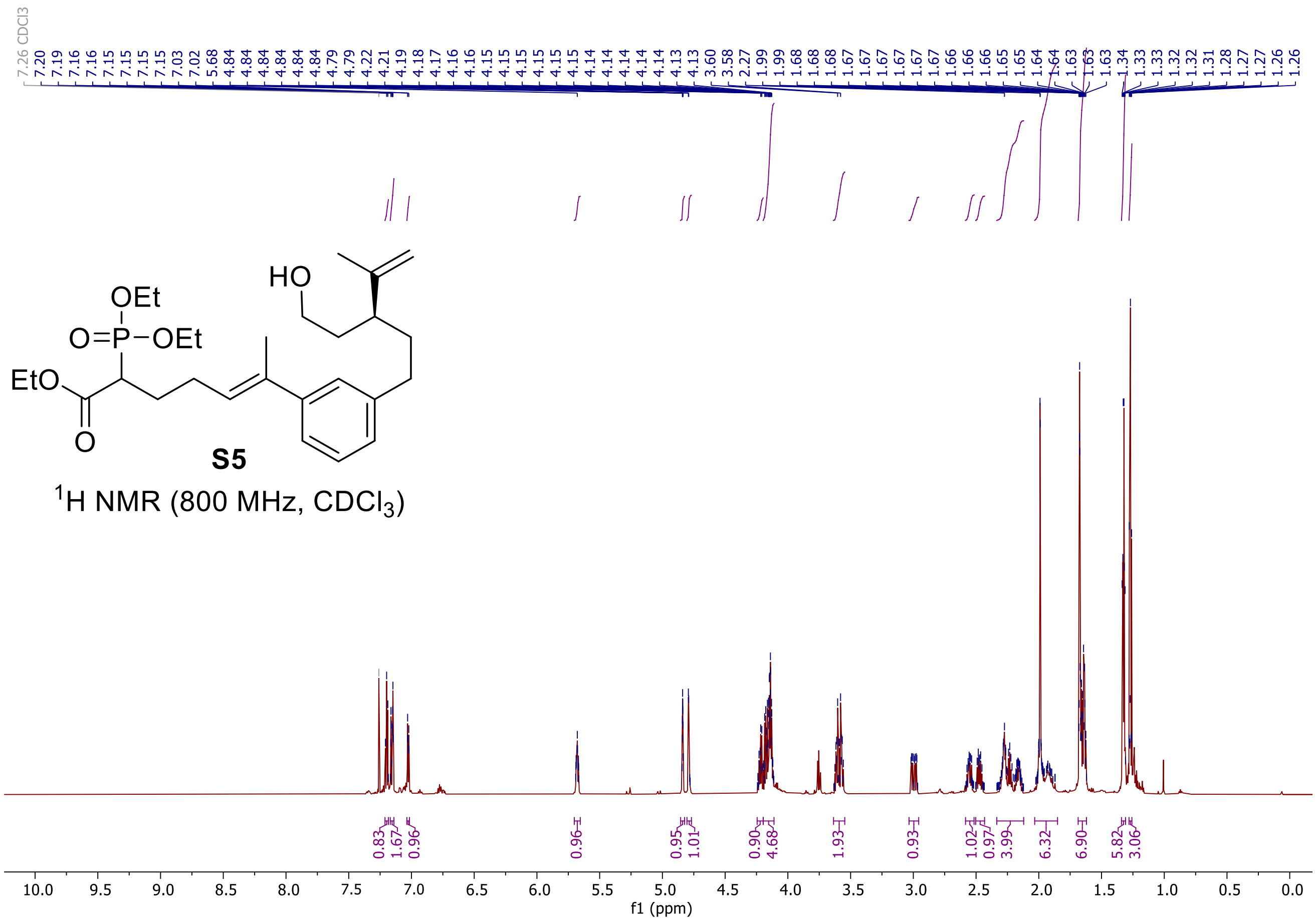
7.26 CDCl3
7.20 7.19 7.16 7.16 7.15 7.15 7.15 7.15 7.03 7.02 5.68 4.84 4.84 4.84 4.84 4.84 4.84 4.79 4.79 4.22 4.21 4.19 4.18 4.17 4.16 4.16 4.15 4.15 4.15 4.15 4.15 4.15 4.14 4.14 4.14 4.14 4.14 4.14 4.13 4.13 3.60 3.58 2.27 1.99 1.99 1.68 1.68 1.68 1.67 1.67 1.67 1.67 1.67 1.67 1.66 1.66 1.66 1.65 1.65 1.64 1.64 1.63 1.63 1.63 1.34 1.33 1.33 1.32 1.32 1.31 1.28 1.27 1.26 1.26
HO
OEt
O=P−OEt
EtO
O
S5
1H NMR (800 MHz, CDCl3)
0.83 1.67 0.96 0.96 0.95 1.01 0.90 4.68 1.93 0.93 1.02 0.97 3.99 6.32 6.90 5.82 3.06
10.0 9.5 9.0 8.5 8.0 7.5 7.0 6.5 6.0 5.5 5.0 4.5 4.0 3.5 3.0 2.5 2.0 1.5 1.0 0.5 0.0
f1 (ppm)

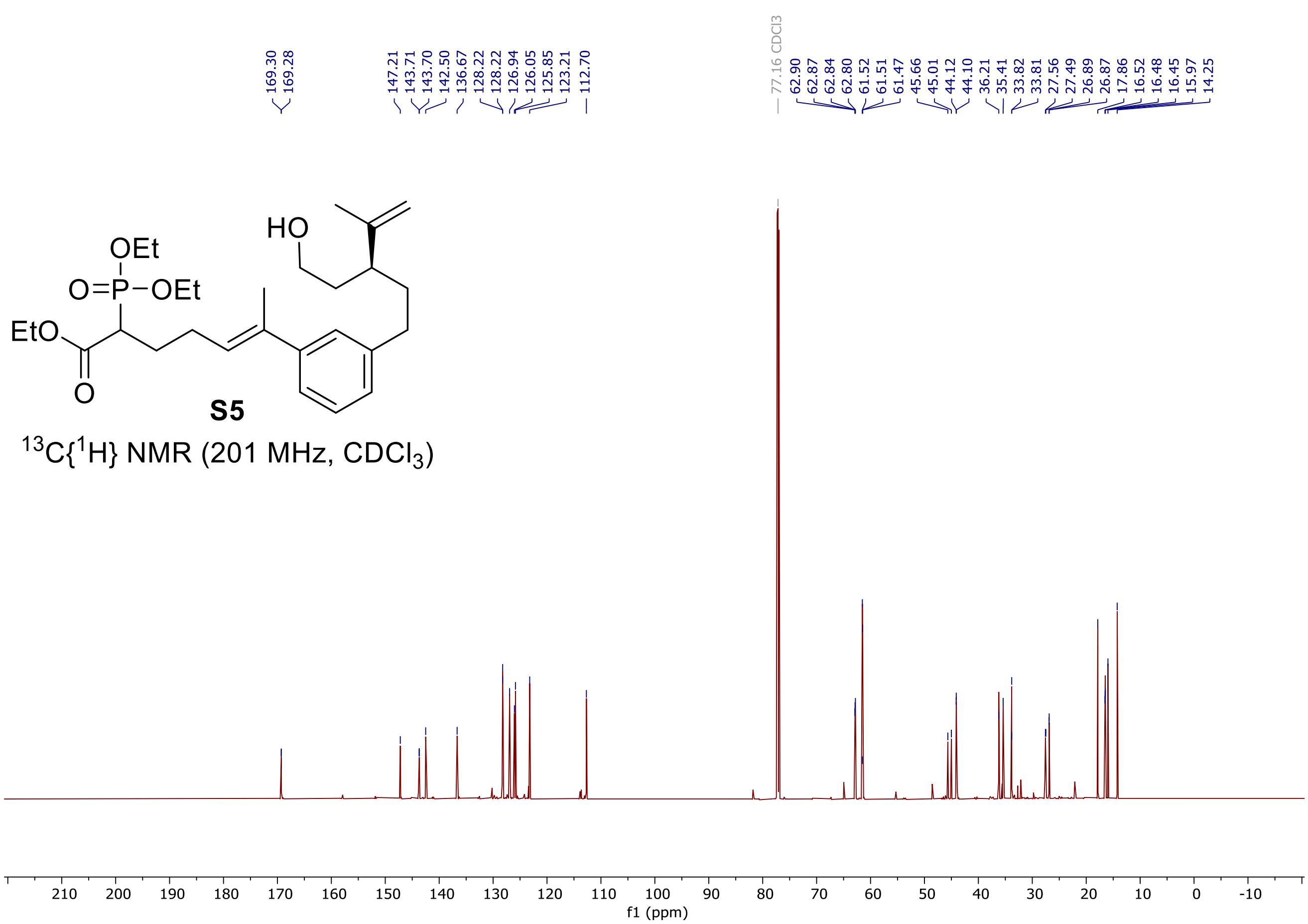
169.30 169.28
147.21 143.71 143.70 142.50 136.67 128.22 128.22 126.94 126.05 125.85 123.21 112.70
77.16 CDCl3
62.90 62.87 62.84 62.80 61.52 61.51 61.47 45.66 45.01 44.12 44.10 36.21 35.41 33.82 33.81 27.56 27.49 26.89 26.87 17.86 16.52 16.48 16.45 15.97 14.25
HO
OEt
O=P−OEt
EtO
O
S5
13C{1H} NMR (201 MHz, CDCl3)
210 200 190 180 170 160 150 140 130 120 110 100 90 80 70 60 50 40 30 20 10 0 -10
f1 (ppm)

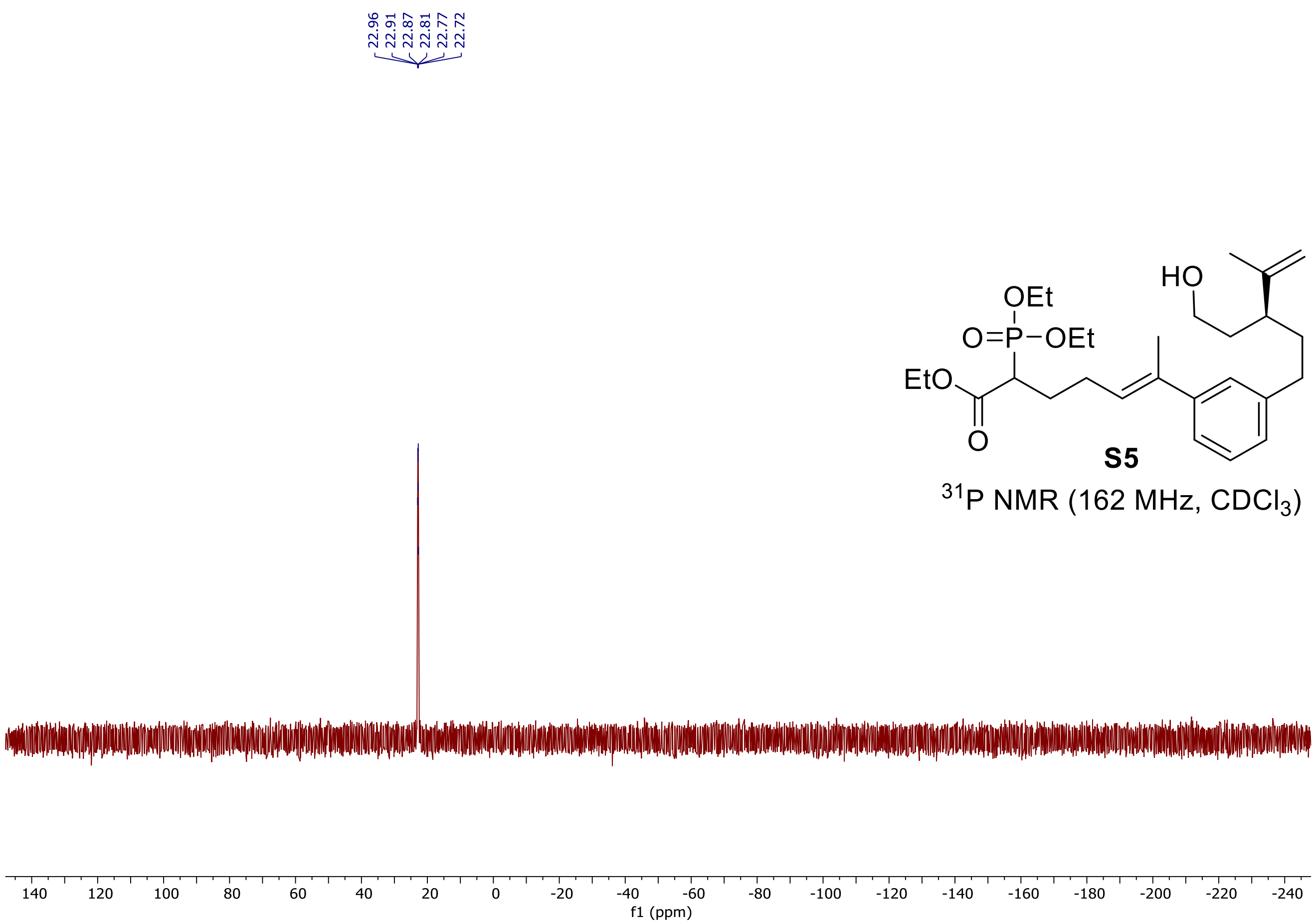

22.96
22.91
22.87
22.81
22.77
22.72
OEt
O=P−OEt
EtO
O
HO
S5
³¹P NMR (162 MHz, CDCl₃)
140
120
100
80
60
40
20
0
-20
-40
-60
-80
-100
-120
-140
-160
-180
-200
-220
-240
f1 (ppm)

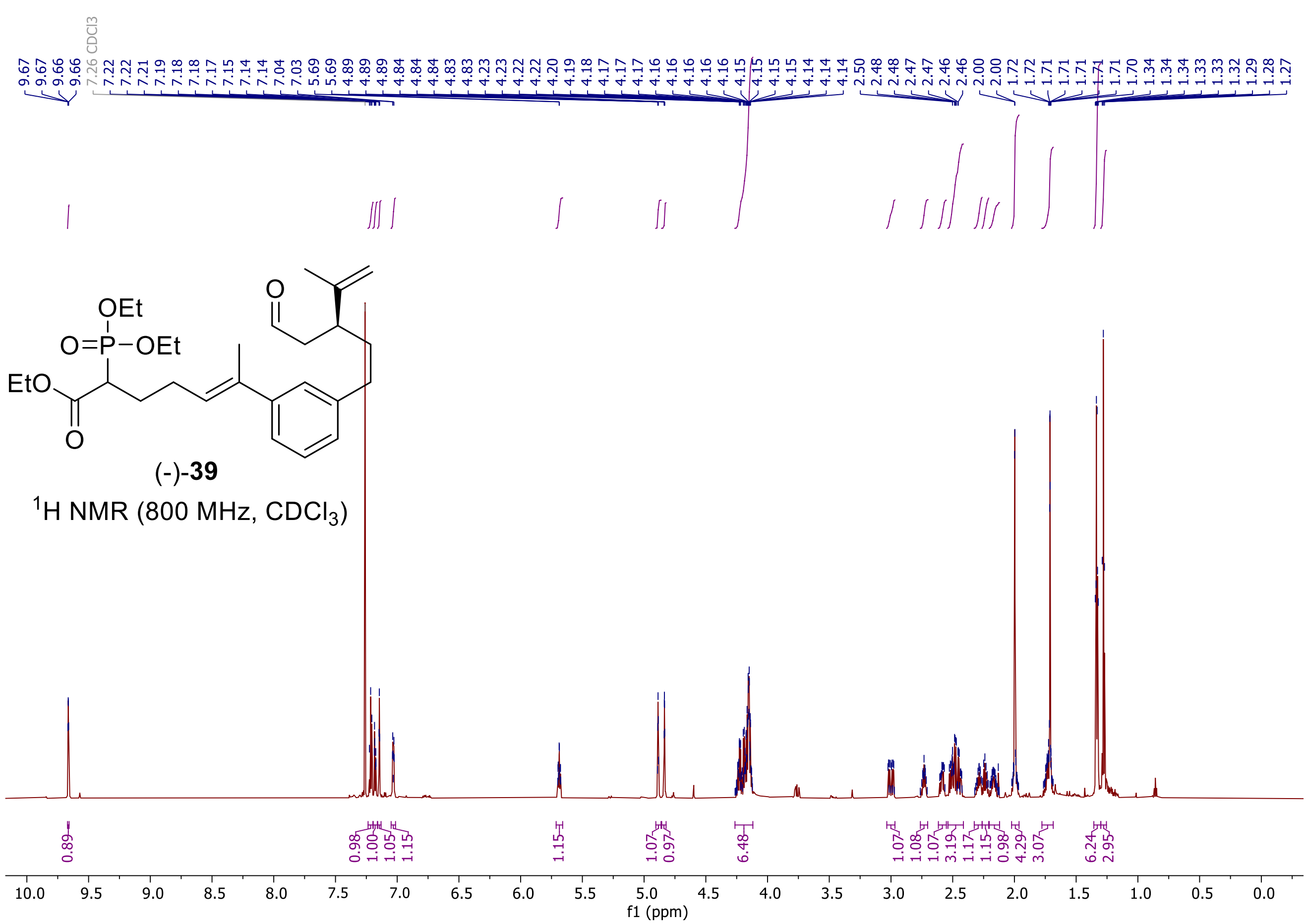

9.67 9.67 9.66 9.66 7.26 CDCl3 7.22 7.22 7.21 7.19 7.18 7.18 7.17 7.15 7.14 7.14 7.04 7.03 5.69 5.69 4.89 4.89 4.89 4.84 4.84 4.84 4.83 4.83 4.23 4.23 4.22 4.22 4.20 4.19 4.18 4.17 4.17 4.17 4.16 4.16 4.16 4.16 4.15 4.15 4.15 4.15 4.14 4.14 4.14 2.50 2.48 2.48 2.47 2.47 2.46 2.46 2.00 2.00 1.72 1.72 1.71 1.71 1.71 1.71 1.71 1.70 1.34 1.34 1.34 1.33 1.33 1.32 1.29 1.28 1.27
OEt
O=P−OEt
EtO
O
(-)-39
^{1}H NMR (800 MHz, CDCl$_3$)
0.89 0.98 1.00 1.05 1.15 1.15 1.07 0.97 6.48 1.07 1.08 1.07 3.19 1.17 1.15 0.98 4.29 3.07 6.24 2.95
10.0 9.5 9.0 8.5 8.0 7.5 7.0 6.5 6.0 5.5 5.0 4.5 4.0 3.5 3.0 2.5 2.0 1.5 1.0 0.5 0.0
f1 (ppm)

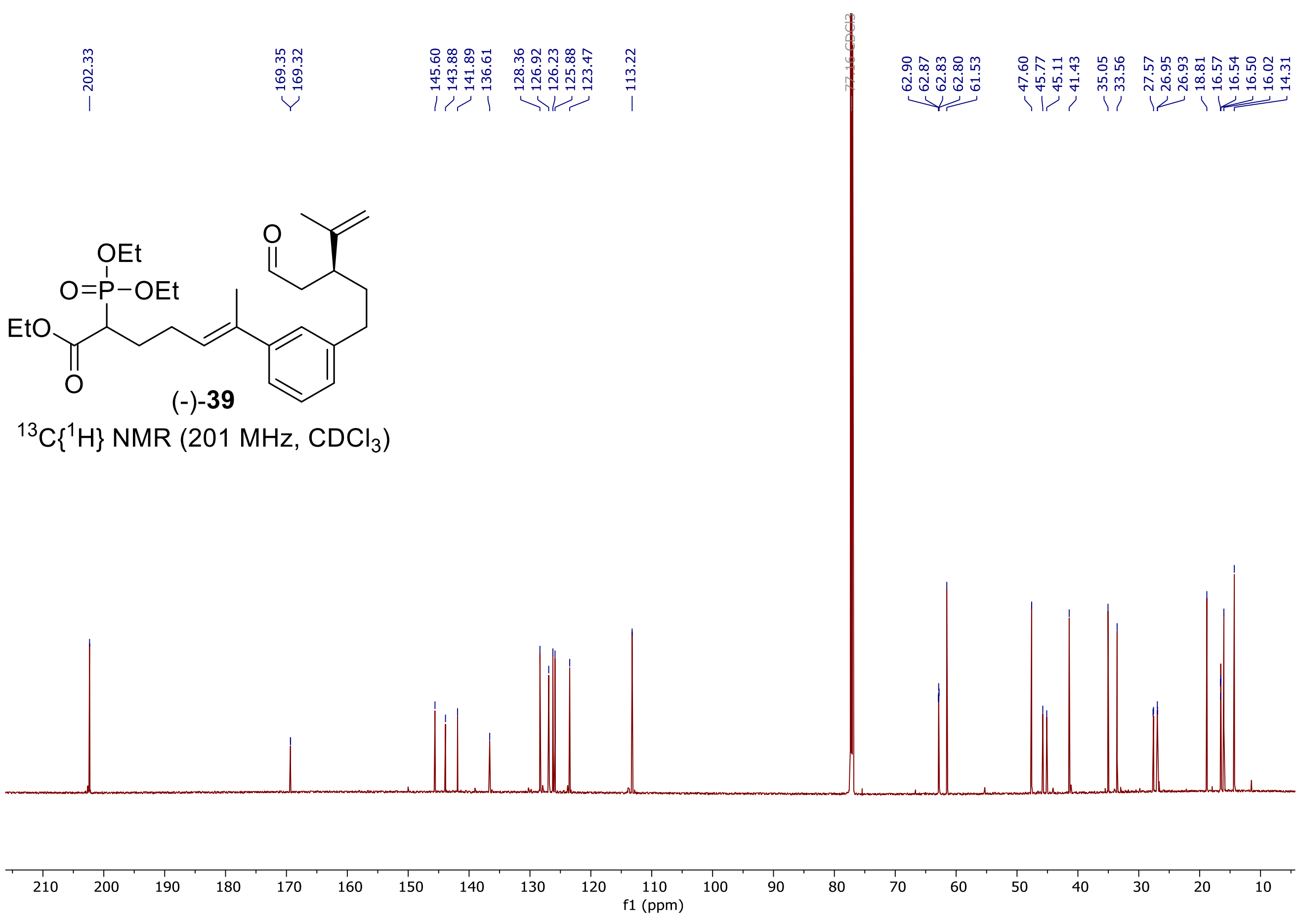

202.33 169.35 169.32 145.60 143.88 141.89 136.61 128.36 126.92 126.23 125.88 123.47 113.22 77.16 CDCl3 62.90 62.87 62.83 62.80 61.53 47.60 45.77 45.11 41.43 35.05 33.56 27.57 26.95 26.93 18.81 16.57 16.54 16.50 16.02 14.31
OEt
O=P−OEt
EtO
O
(-)-39
^{13}C{^{1}H} NMR (201 MHz, CDCl$_3$)
210 200 190 180 170 160 150 140 130 120 110 100 90 80 70 60 50 40 30 20 10
f1 (ppm)

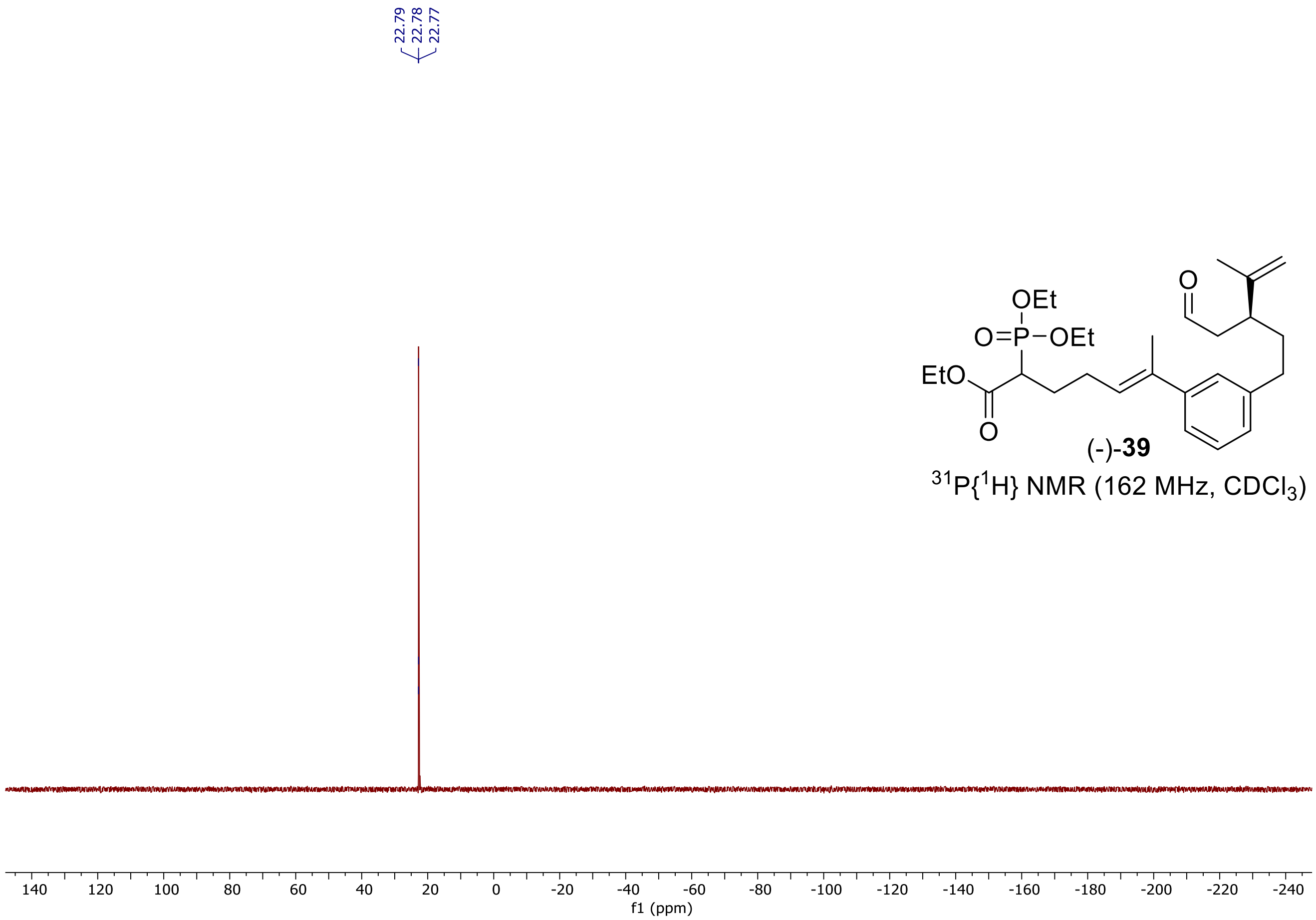

22.79
22.78
22.77
OEt
O=P−OEt
EtO
O
O
(-)-39
$^{31}P\{^{1}H\}$ NMR (162 MHz, $CDCl_3$)
140 120 100 80 60 40 20 0 -20 -40 -60 -80 -100 -120 -140 -160 -180 -200 -220 -240
f1 (ppm)

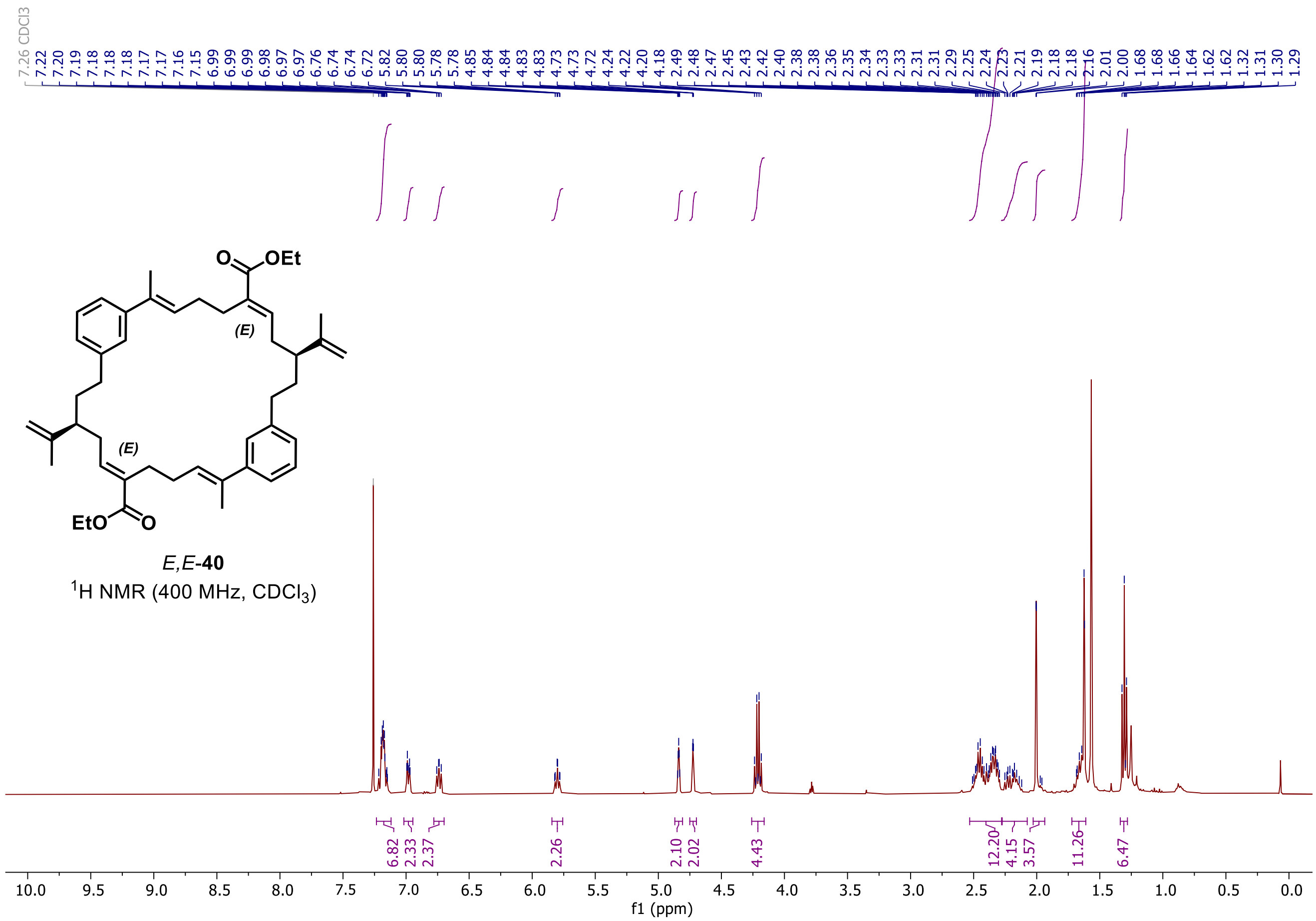

E,E-**40**

^{1}H NMR (400 MHz, $CDCl_3$)

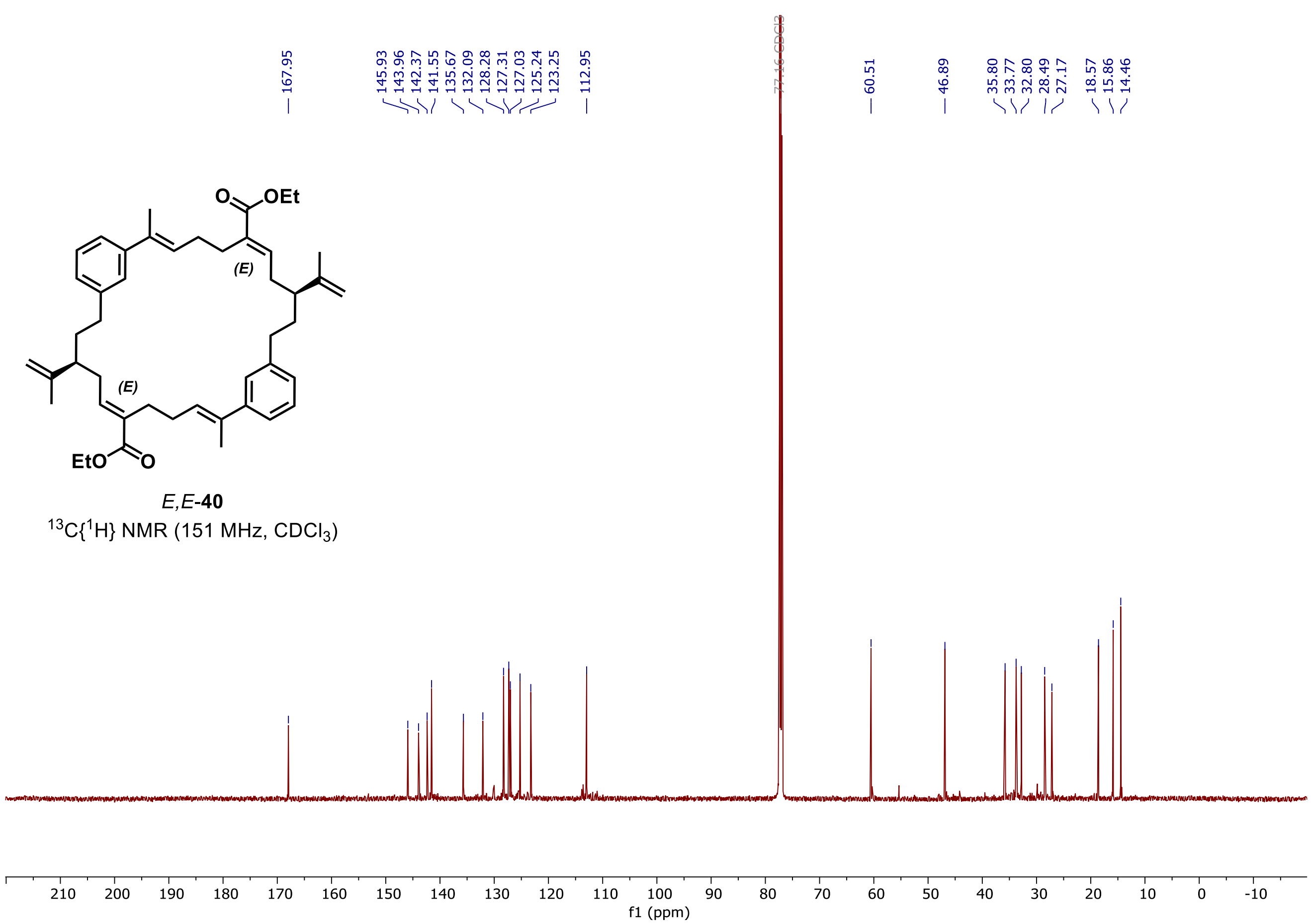

E,E-**40**

$^{13}C\{^1H\}$ NMR (151 MHz, $CDCl_3$)

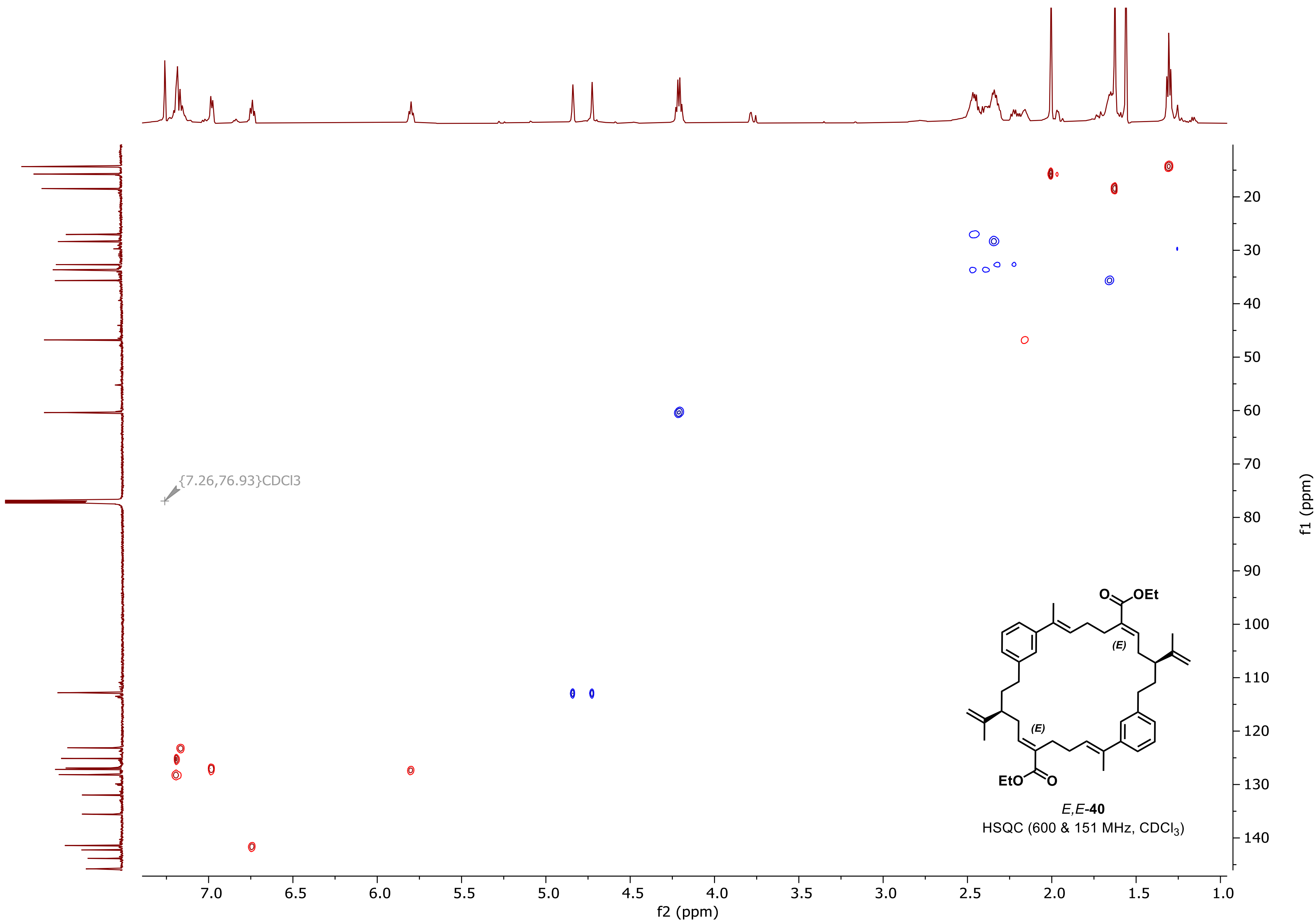

E,*E*-**40**
HSQC (600 & 151 MHz, $CDCl_3$)

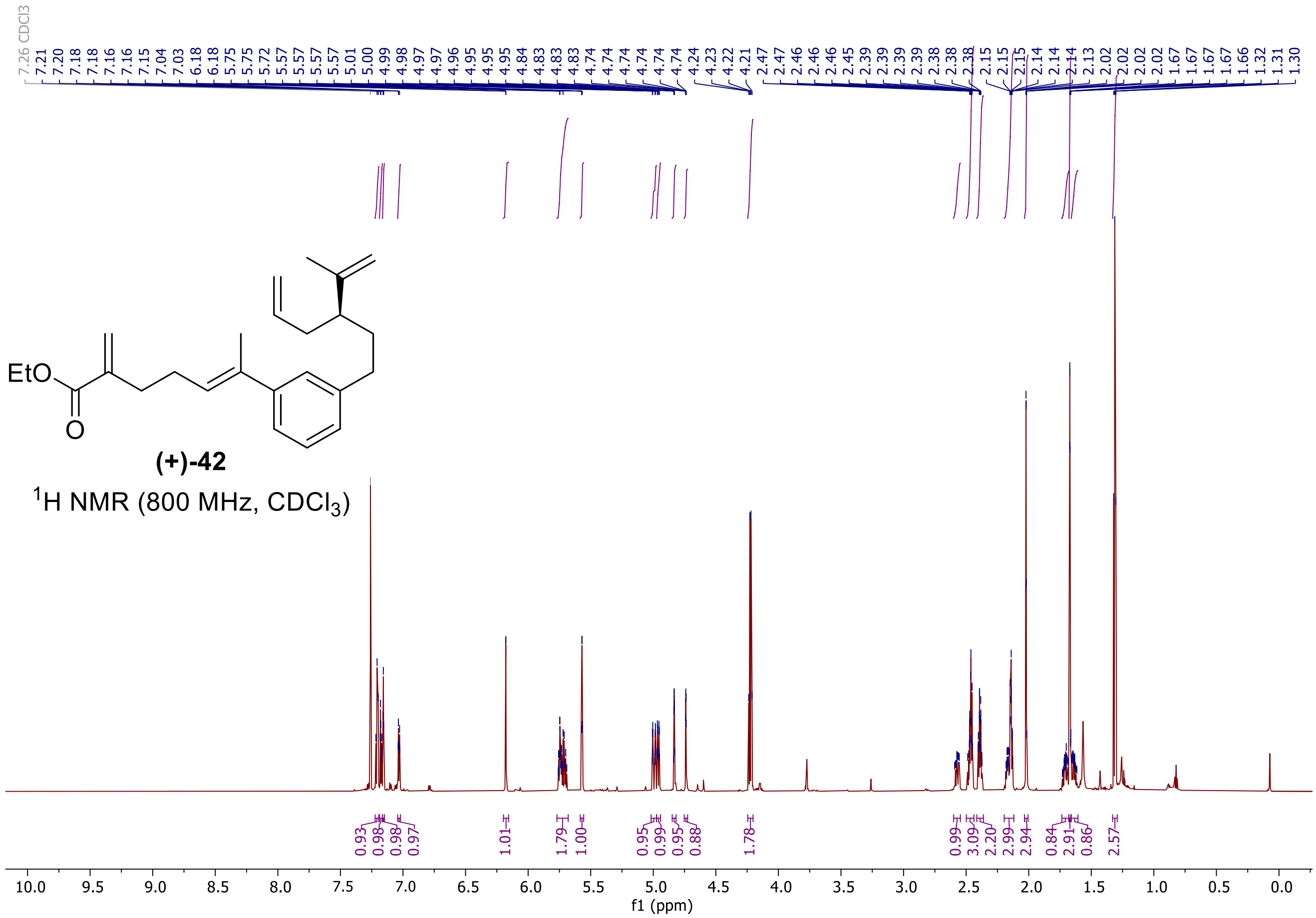
7.26 CDCl3
7.21 7.20 7.18 7.16 7.16 7.15 7.04 7.03 6.18 6.18 5.75 5.75 5.72 5.57 5.57 5.57 5.57 5.01 5.00 4.99 4.98 4.97 4.97 4.96 4.95 4.95 4.95 4.84 4.83 4.83 4.83 4.74 4.74 4.74 4.74 4.74 4.74 4.24 4.23 4.22 4.21 2.47 2.47 2.46 2.46 2.45 2.39 2.39 2.39 2.39 2.38 2.38 2.38 2.15 2.15 2.15 2.14 2.14 2.14 2.13 2.02 2.02 2.02 2.02 1.67 1.67 1.67 1.67 1.66 1.32 1.31 1.30
EtO
O
(+)-42
^{1}H NMR (800 MHz, $CDCl_3$)
0.93 0.98 0.98 0.97 1.01 1.79 1.00 0.95 0.99 0.95 0.88 1.78 0.99 3.09 2.20 2.99 2.94 0.84 2.91 0.86 2.57
10.0 9.5 9.0 8.5 8.0 7.5 7.0 6.5 6.0 5.5 5.0 4.5 4.0 3.5 3.0 2.5 2.0 1.5 1.0 0.5 0.0
f1 (ppm)

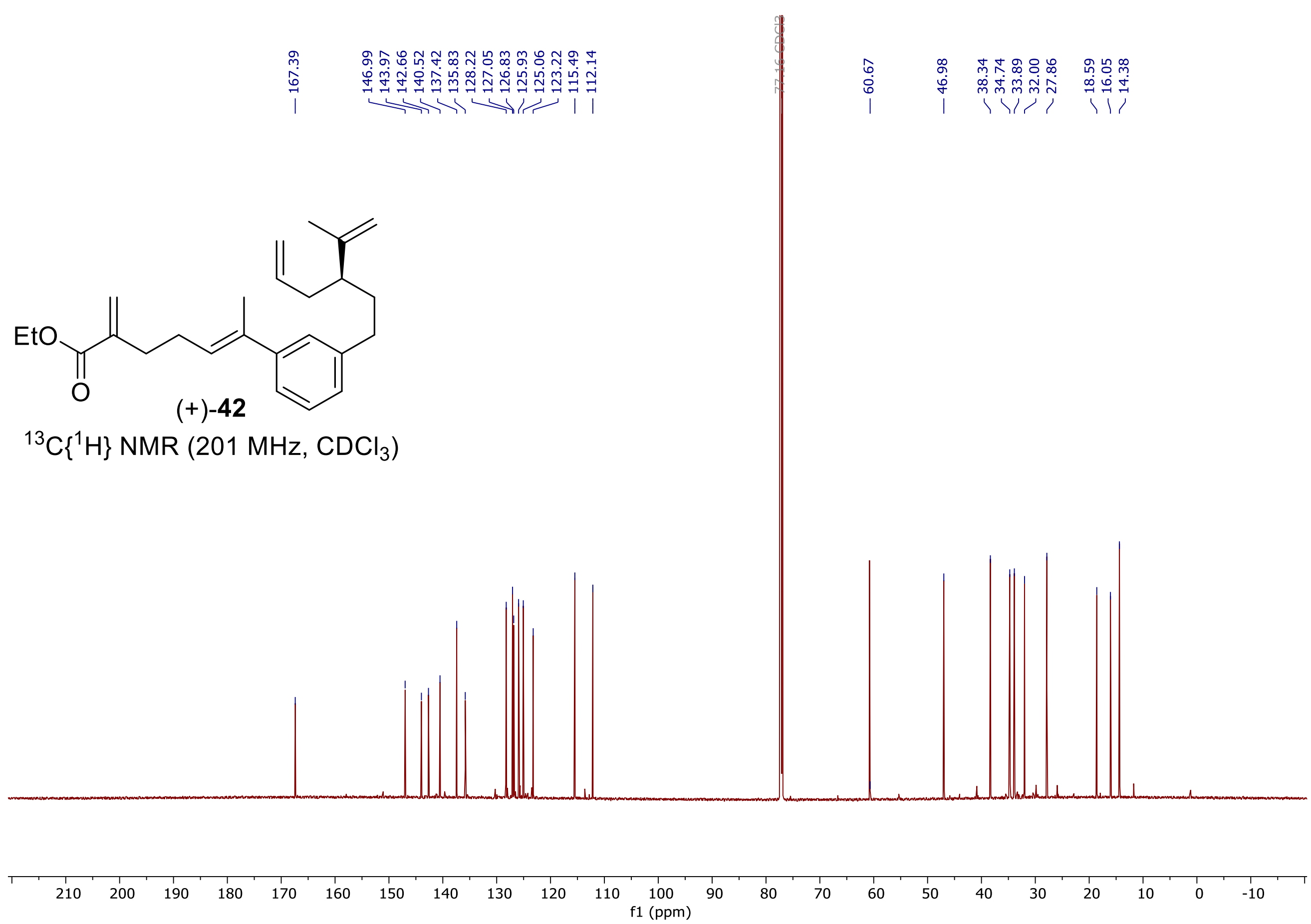
167.39
146.99 143.97 142.66 140.52 137.42 135.83 128.22 127.05 126.83 125.93 125.06 123.22 115.49 112.14
77.16 CDCl3
60.67
46.98
38.34 34.74 33.89 32.00 27.86
18.59 16.05 14.38
EtO
O
(+)-42
$^{13}C\{^{1}H\}$ NMR (201 MHz, $CDCl_3$)
210 200 190 180 170 160 150 140 130 120 110 100 90 80 70 60 50 40 30 20 10 0 -10
f1 (ppm)

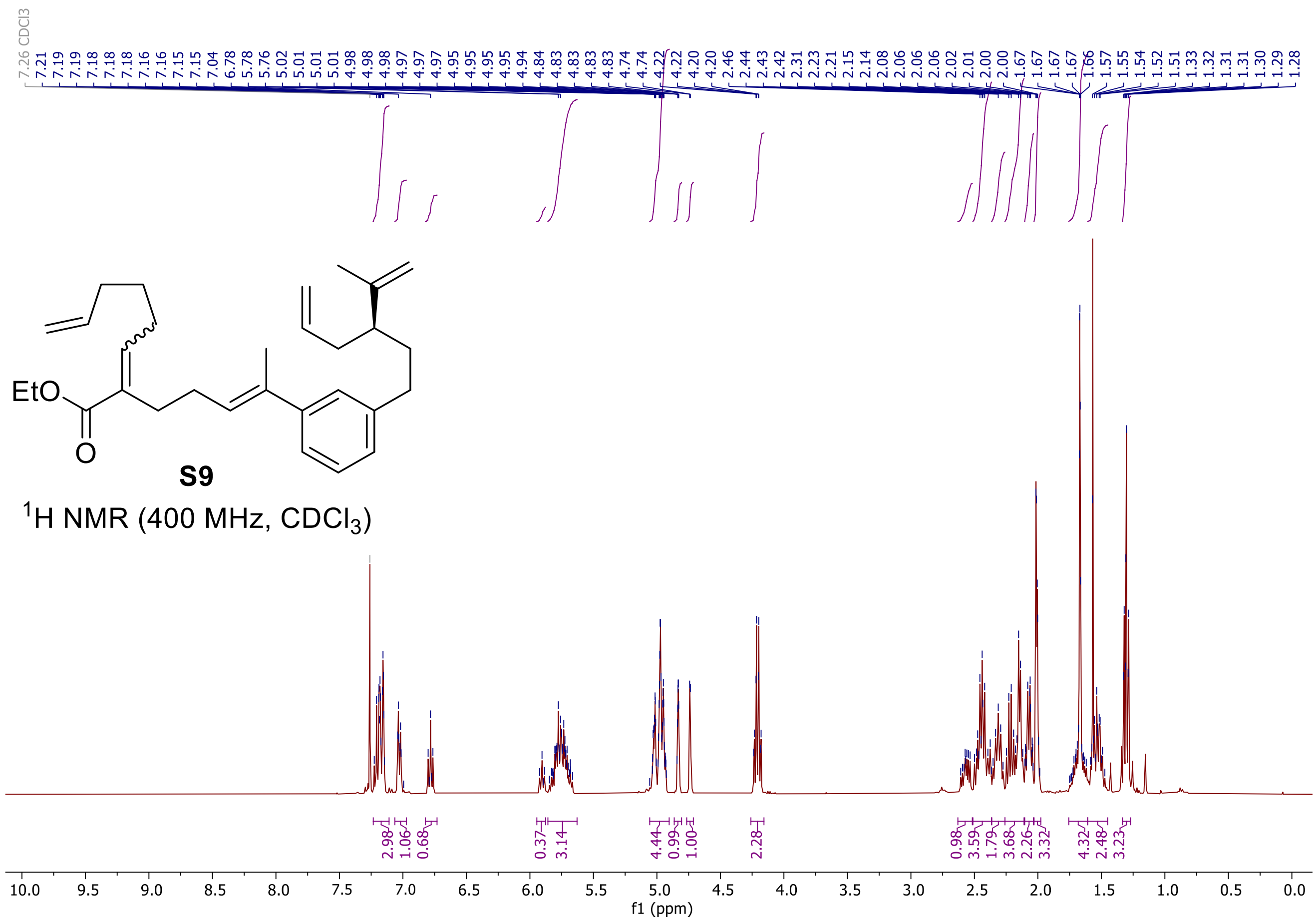
7.26 CDCl3
7.21 7.19 7.19 7.18 7.18 7.18 7.16 7.16 7.15 7.15 7.04 6.78 5.78 5.76 5.02 5.01 5.01 4.98 4.98 4.97 4.97 4.97 4.95 4.95 4.95 4.95 4.94 4.84 4.83 4.83 4.83 4.83 4.74 4.74 4.22 4.22 4.20 4.20 2.46 2.44 2.43 2.42 2.31 2.23 2.21 2.15 2.14 2.08 2.06 2.06 2.06 2.06 2.02 2.01 2.00 2.00 1.67 1.67 1.67 1.67 1.66 1.57 1.55 1.54 1.52 1.51 1.33 1.32 1.31 1.31 1.30 1.29 1.28
EtO
O
S9
^{1}H NMR (400 MHz, $CDCl_3$)
2.98 1.06 0.68 0.37 3.14 4.44 0.99 1.00 2.28 0.98 3.59 1.79 3.68 2.26 3.32 4.32 2.48 3.23
10.0 9.5 9.0 8.5 8.0 7.5 7.0 6.5 6.0 5.5 5.0 4.5 4.0 3.5 3.0 2.5 2.0 1.5 1.0 0.5 0.0
f1 (ppm)

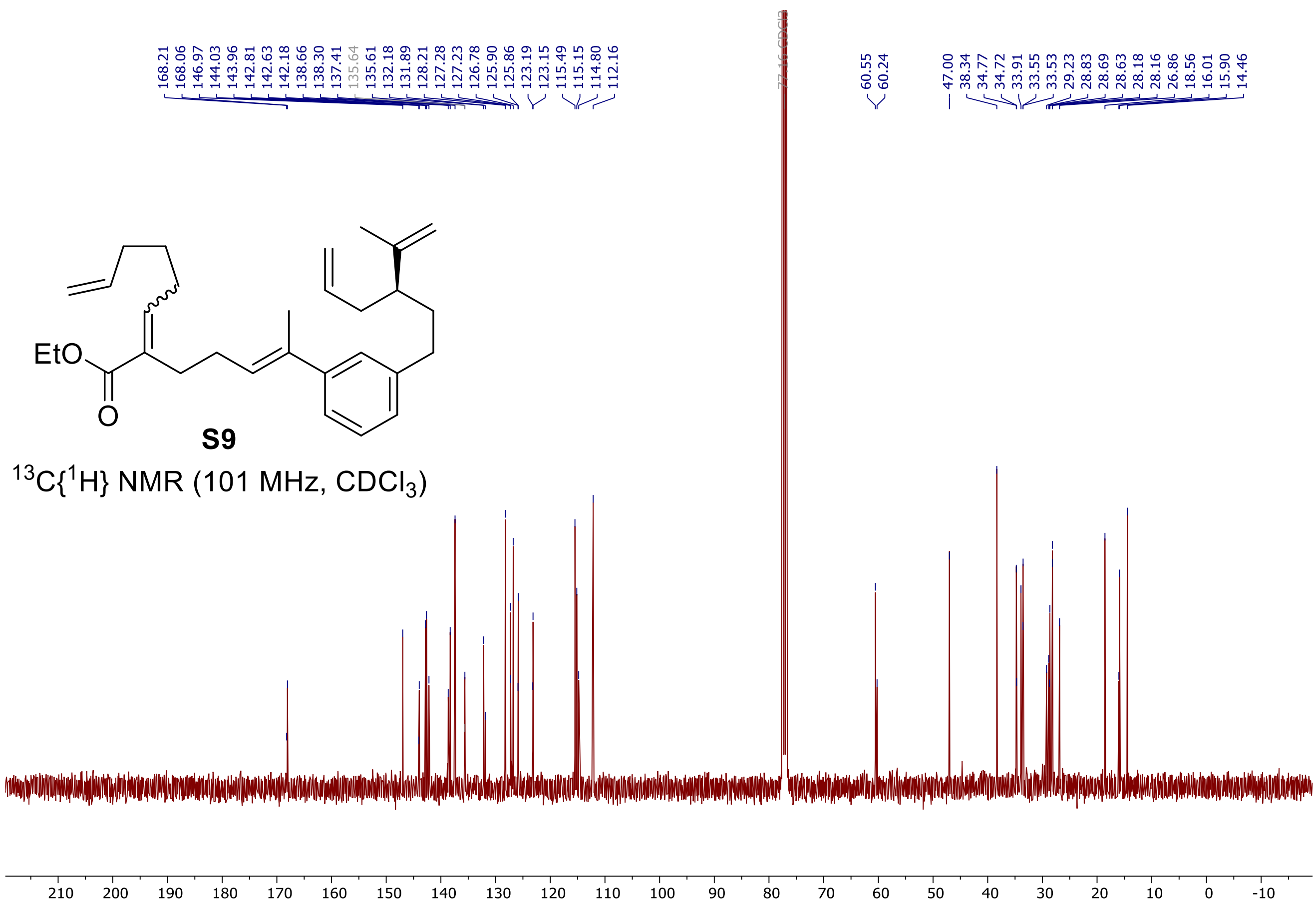
168.21 168.06 146.97 144.03 143.96 142.81 142.63 142.18 138.66 138.30 137.41 135.64 135.61 132.18 131.89 128.21 127.28 127.23 126.78 125.90 125.86 123.19 123.15 115.49 115.15 114.80 112.16
77.16 CDCl3
60.55 60.24 47.00 38.34 34.77 34.72 33.91 33.55 33.53 29.23 28.83 28.69 28.63 28.18 26.86 18.56 16.01 15.90 14.46
EtO
O
S9
$^{13}C\{^{1}H\}$ NMR (101 MHz, $CDCl_3$)
210 200 190 180 170 160 150 140 130 120 110 100 90 80 70 60 50 40 30 20 10 0 -10
f1 (ppm)

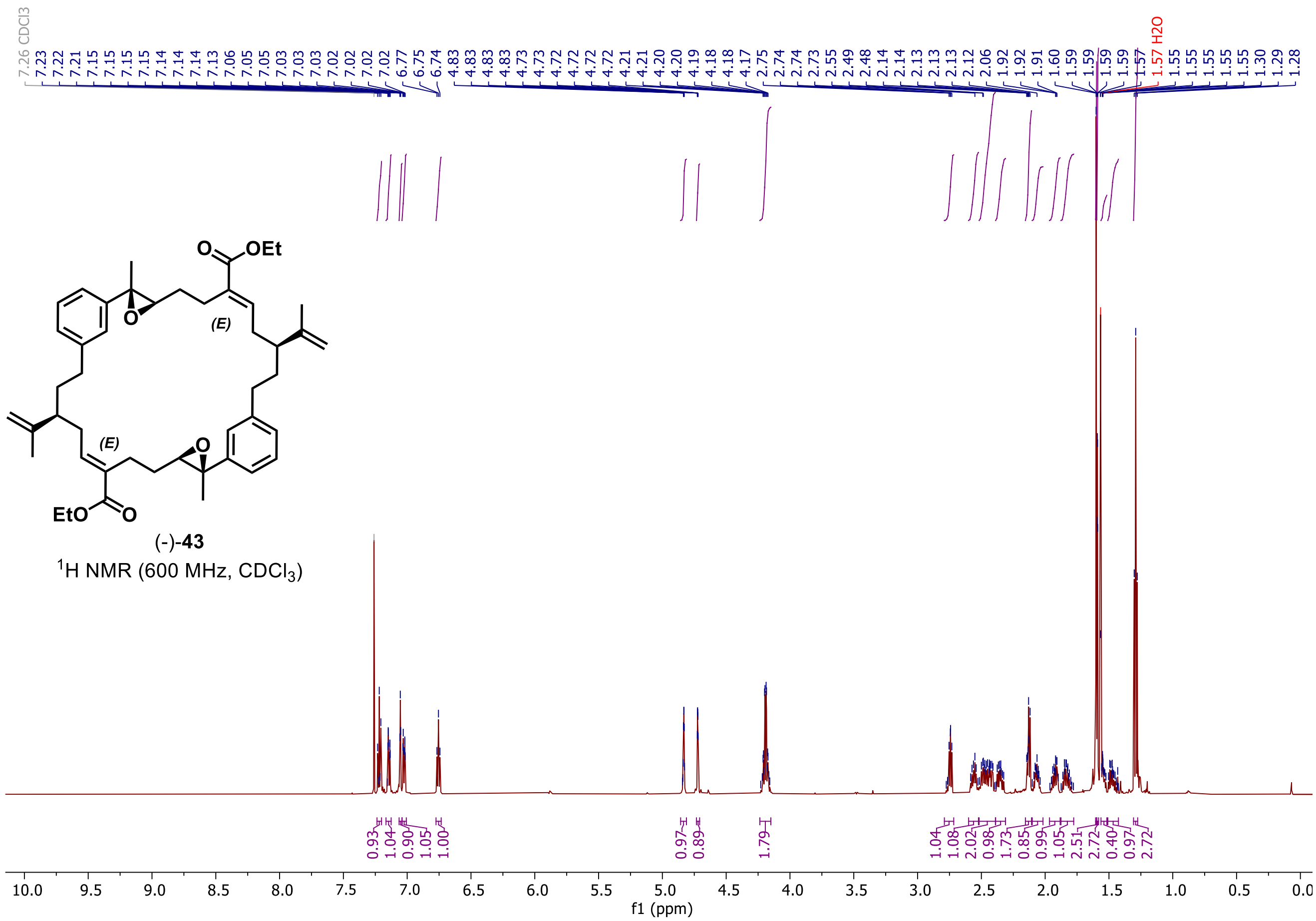
(-)-43
^{1}H NMR (600 MHz, $CDCl_3$)
f1 (ppm)

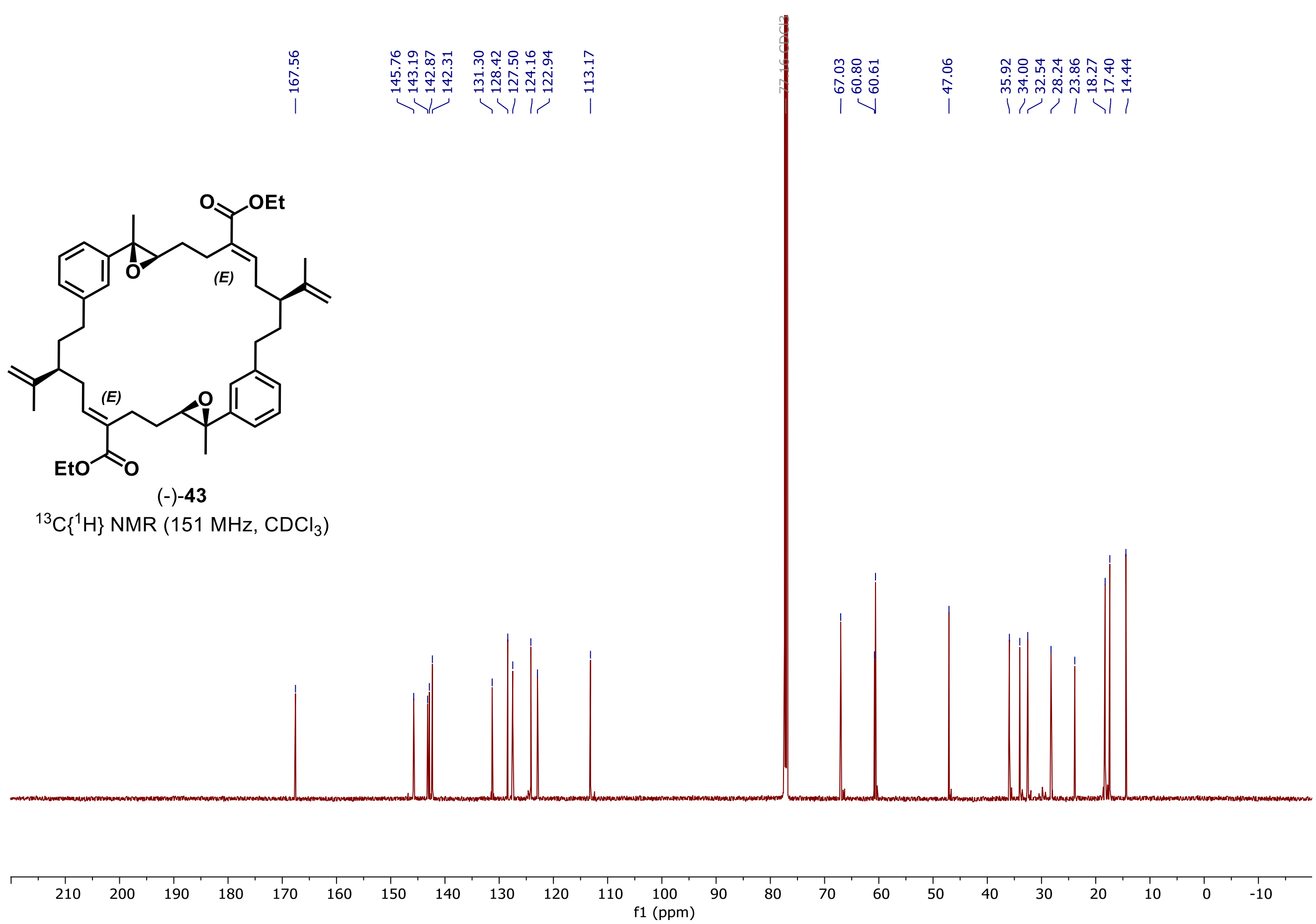
(-)-43
$^{13}C\{^{1}H\}$ NMR (151 MHz, $CDCl_3$)
f1 (ppm)

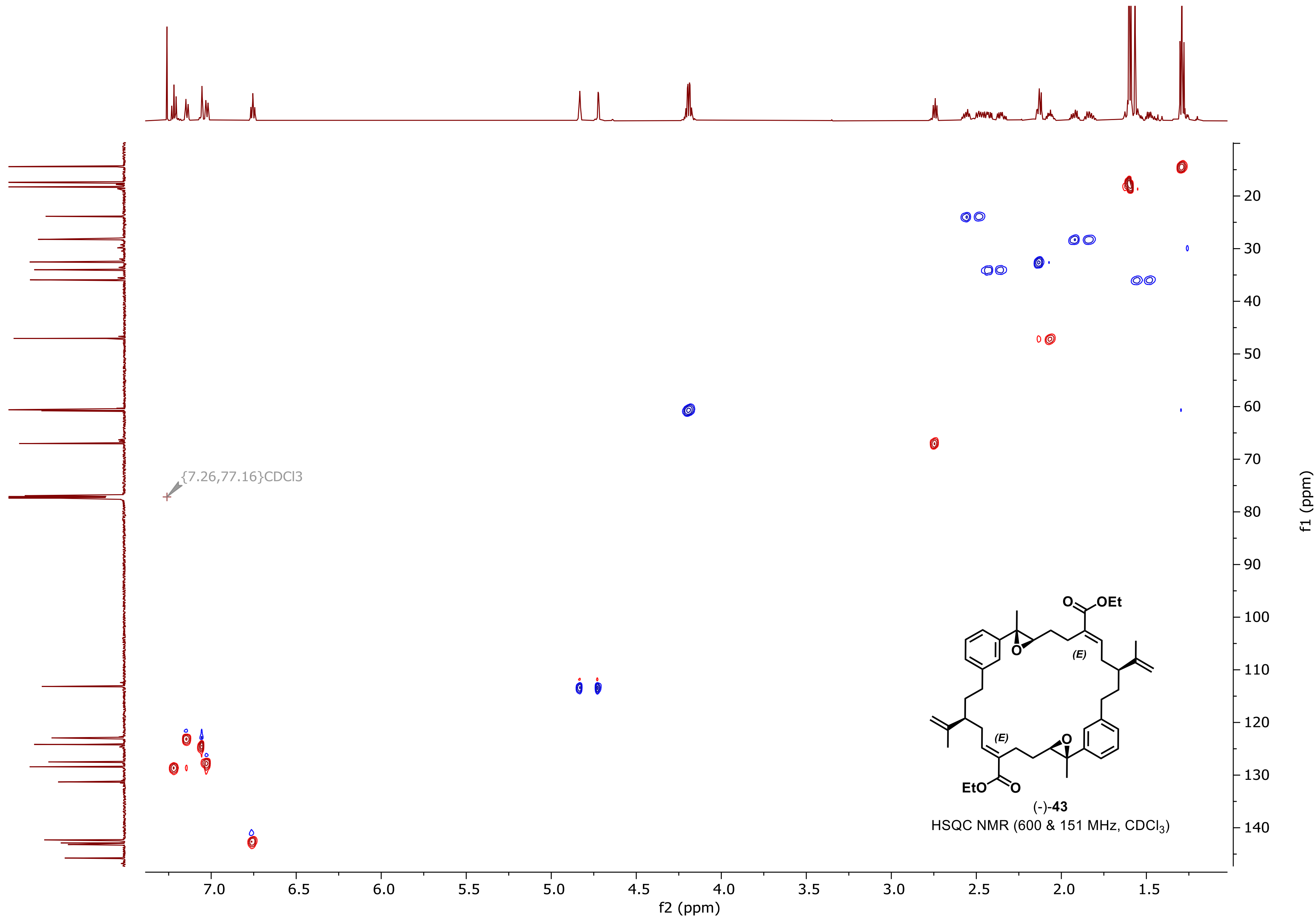

(-)-**43**
HSQC NMR (600 & 151 MHz, $CDCl_3$)
